# tRNA gene content, structure, and organization in the flowering plant lineage

**DOI:** 10.3389/fpls.2024.1486612

**Published:** 2024-12-23

**Authors:** Kim Carlo Monloy, Jose Planta

**Affiliations:** National Institute of Molecular Biology and Biotechnology, College of Science, University of the Philippines Diliman, Quezon City, Philippines

**Keywords:** tRNA genes, tDNA, tRNA gene content, tRNA gene organization, tRNA gene structure

## Abstract

Transfer RNAs (tRNAs) are noncoding RNAs involved in protein biosynthesis and have noncanonical roles in cellular metabolism, such as RNA silencing and the generation of transposable elements. Extensive tRNA gene duplications, modifications to mature tRNAs, and complex secondary and tertiary structures impede tRNA sequencing. As such, a comparative genomic analysis of complete tRNA sets is an alternative to understanding the evolutionary processes that gave rise to the extant tRNA sets. Although the tRNA gene (tDNA) structure and distribution in prokaryotes and eukaryotes, specifically in vertebrates, yeasts, and flies, are well understood, there is little information regarding plants. A detailed and comprehensive analysis and annotation of tDNAs from the genomes of 44 eudicots, 20 monocots, and five other non-eudicot and non-monocot species belonging to the Ceratophyllaceae and the ANA (Amborellales, Nymphaeales, and Austrobaileyales) clade will provide a global picture of plant tDNA structure and organization. Plant genomes exhibit varying numbers of nuclear tDNAs, with only the monocots showing a strong correlation between nuclear tDNA numbers and genome sizes. In contrast, organellar tDNA numbers varied little among the different lineages. A high degree of tDNA duplication in eudicots was detected, whereby most eudicot nuclear genomes (91%) and only a modest percentage of monocot (65%) and ANA nuclear genomes (25%) contained at least one tDNA cluster. Clusters of tRNA^Tyr^–tRNA^Ser^ and tRNA^Ile^ genes were found in eudicot and monocot genomes, respectively, while both eudicot and monocot genomes showed clusters of tRNA^Pro^ genes. All plant genomes had intron-containing tRNA^eMet^ and tRNA^Tyr^ genes with modest sequence conservation and a strictly conserved tRNA^Ala-AGC^ species. Regulatory elements found upstream (TATA-box and CAA motifs) and downstream (poly(T) signals) of the tDNAs were present in only a fraction of the detected tDNAs. A and B boxes within the tDNA coding region show varying consensus sequences depending on the tRNA isotype and lineage. The chloroplast genomes, but not the mitogenomes, possess relatively conserved tRNA gene organization. These findings reveal differences and patterns acquired by plant genomes throughout evolution and can serve as a foundation for further studies on plant tRNA gene function and regulation.

## 1 Introduction

Transfer RNAs (tRNAs) are short, noncoding molecules acting as intermediaries between the genetic information in nucleic acids and protein sequences. Although the mechanistic roles of tRNAs in ribosomal protein biosynthesis are well understood, they have noncanonical functions in several aspects of cellular metabolism. Plant tRNAs have been implicated in tetrapyrrole and cytokinin biosynthesis (Chery and Drouard, 2022), plant cell growth and immunity (Soprano et al., 2018), and regulation of auxin response in *Arabidopsis* (Leitner et al., 2015). Increased attention has also been given to tRNA-derived fragments (tRFs), a class of small RNAs produced from the enzymatic cleavage of tRNAs. Initially thought as mere tRNA degradation byproducts, tRFs have been linked to gene regulation, ribosome biogenesis, plant–pathogen interactions, and stress response in plants (Park and Kim, 2018; Alves and Nogueira, 2021; Wang et al., 2023; Panstruga and Spanu, 2024). tRFs have been reported to be involved in the RNA silencing pathway and are the major source of the transposable element SINEs (short interspersed nuclear elements; Bermudez-Santana et al., 2010; Phizicky and Hopper, 2010; Soprano et al., 2018). All tRNA genes are postulated to be derived from an ancestral “proto-tRNA” (Eigen et al., 1989), and during evolution, a tRNA repertoire was generated from gene duplication and numerous mutational events. These processes gave rise to the core and dispensable sets of tRNA genes.

Despite the growing knowledge and interest in plant tRNA biology, studies on how tRNAs are structured and organized on a genome-wide scale in plants still number too few. A survey of the content, distribution, and clustering of tRNA genes and pseudogenes in many eukaryotes, including nine genomes from the green lineage, has been reported (Bermudez-Santana et al., 2010). More recent studies have also reported the evolution of tRNA gene content in the three domains of life, involving 13 plant genomes (Santos and Del-Bem, 2023), as well as the tRNA anticodon frequency of 128 plant genomes (Mohanta et al., 2020). Databases of tRNA gene sets from hundreds of plant nuclear and organellar genomes, covering diverse families of plants, have also been developed (e.g., Cognat et al., 2022; Mokhtar and Allali, 2022), whose curators were able to provide a general survey of the tRNA gene populations of 51 and 256 plant species, respectively. However, these mostly only provided insights on the tRNA gene content of these plants, and separate studies fully utilizing the information from these databases are yet to be found. To date, the first comprehensive study that focused on tRNA gene content, structure, and distribution in plants covered both the nuclear and organellar genomes of only five angiosperms—consisting of three eudicots and two monocots—and one green alga (Michaud et al., 2011). However, given the species diversity within the flowering plants, a more comprehensive and systematic comparative study is needed to provide a global landscape of plant tRNA structure and organization. The increased availability of plant genomes will provide common patterns and taxon-specific particularities of plant tRNAs.

Compared to other eukaryotic genomes, plant genomes possess a smaller variation in the number of tRNA genes and a varying abundance of tRNA gene clusters (Bermudez-Santana et al., 2010). The following tRNA gene organization has been reported among flowering plant genomes: a predominantly A-/T-rich region spanning 50 nucleotides upstream of the tRNA gene, an upstream CAA motif and a downstream poly(T) termination signal found in most tRNAs, and intron-containing tRNA^Met^ and tRNA^Tyr^ genes (Michaud et al., 2011). Except for *Arabidopsis*, a similar chromosomal distribution of tRNA genes in terms of the numbers of tRNA genes per megabase of the chromosome was also reported within angiosperms, which hinted at the possibility of excessive tRNA gene duplications in some plant genomes (Michaud et al., 2011). Although a significant correlation between genome size and number of tRNA genes have been reported among 74 eukaryotic genomes (Bermudez-Santana et al., 2010), five plant genomes (Michaud et al., 2011), and eight monocot genomes (Planta et al., 2022), a more recent regression analysis involving a higher coverage of plant genomes (128 genomes) instead reported a weak correlation (Mohanta et al., 2020).

In the case of organellar genomes, previous studies also reported the lack of certain tRNA isoacceptors in some plant plastomes and mitogenomes (Michaud et al., 2011; Mohanta et al., 2020). Although possessing significantly fewer tRNA genes than the nuclear genome, the organellar genomes from mitochondria and chloroplasts can also encode their tRNAs. The chloroplast genome is assumed to encode all the tRNA species required for protein synthesis, and unlike the mitochondria, chloroplasts do not import cytosolic tRNAs (Maréchal-Drouard et al., 1993). A relaxed wobble rule might also explain the small number of organellar tRNAs that can read all codons of the universal genetic code (Crick, 1966; Percudani, 2001).

Several different sequencing-based approaches have been developed to quantify highly modified tRNAs. However, modifications on tRNAs can impair cDNA synthesis by premature reverse transcriptase (RT) stops (Pinkard et al., 2020; Padhiar et al., 2024). These methods [e.g., ARM-seq (Cozen et al., 2015), DM-TGIRT-seq (Zheng et al., 2015), YAMAT-seq (Shigematsu et al., 2017), Nano-tRNAseq (Lucas et al., 2024); see Padhiar et al. (2024) for a comprehensive review] incorporate pre-treatment of RNA before library construction and the use of modified adapters; pre-treatment of RNA produces less complex secondary structures and fewer modifications that can lead to premature RT stops (Padhiar et al., 2024). Plant tRNA expression and post-transcriptional modifications have been characterized in *Arabidopsis thaliana* by modifying RNA-seq to involve a demethylating enzyme and using a tRNA-specific adapter (Shigematsu et al., 2017; Warren et al., 2021). While these are promising advancements in direct tRNA sequencing, at its current state, computationally predicting tRNA genes from whole-genome sequencing data is still the preferred method in most tRNA gene studies (Chan et al., 2021).

This study compared and analyzed the tRNA gene content, structure, and organization of 69 nuclear plant genomes—including available chloroplast and mitochondrial genome counterparts (**Supplementary Table 1**). Included in our analyses are 44 genomes from the eudicot lineage, 20 from the monocot lineage, four from the ANA clade (Amborellales, Nymphaeales, and Austrobaileyales), and one from Ceratophyllaceae, the sister clade to eudicots. The eudicot and monocot genomes were chosen to cover as much family in the flowering plant lineage; the chosen plant genomes span 32 families—two from the ANA clade (Amborellaceae and Nymphaeaceae), Ceratophyllaceae, nine from monocots, and 20 from dicots (**Supplementary Figure 1**). Having these lineages within the scope of this study should provide a better and more inclusive analysis of tRNA genes in plants. Using the widely adopted tool tRNAscan-SE (Chan et al., 2021), tRNA genes from these genomes were computationally predicted and then filtered for a “high-confidence” set of tRNA genes discarding pseudogenes. To characterize these “high-confidence” tRNA genes, we also screened the tDNAs for regulatory sequences commonly associated with the RNA polymerase III-transcribed plant tRNA genes: the upstream TATA-box and CAA motifs (Choisne et al., 1998; Yukawa et al., 2000; Dieci et al., 2006; Michaud et al., 2011), the intragenic A and B boxes (Choisne et al., 1998; Dieci et al., 2006), and the downstream poly(T) stretches (Yukawa et al., 2000; Braglia et al., 2005; Arimbasseri and Maraia, 2015).

Comparative genomics analyses revealed that the number of nuclear tRNA genes varied mainly among the plant genomes studied, even among genomes of the same lineage. In contrast, the number of organellar tRNA genes had slight variation and was consistent regardless of plant lineage. Moreover, gene duplications in tRNA gene clusters appeared more prevalent in eudicots. All nuclear genomes were found to have a strictly conserved tRNA^Ala-AGC^ species and intron-containing tRNA^eMet^ and tRNA^Tyr^ genes that exhibited modest sequence conservation. Regulatory sequences found in the nuclear tRNA genes include the upstream TATA-box and CAA motifs (found upstream of 22%–32% and 78%–82% of tRNA genes detected, respectively), the intragenic A and B boxes (found in all tRNA genes detected) with general lineage- and isotype-specific motifs, and the downstream poly(T) termination signals (found downstream of 67%–72% of tRNA genes detected). Overall, this study revealed differences and patterns acquired by plant genomes throughout evolution and can serve as a foundation for further studies on plant tRNA gene function and regulation.

## 2 Materials and methods

### 2.1 Phylogenetic tree construction

Nuclear and organellar genomes from 69 flowering plant species encompassing the ANA, Ceratophyllaceae, eudicot, and monocot lineages used in this study are listed in **Supplementary Table 1** and were obtained either from Phytozome (Goodstein et al., 2012) or the NCBI database (Sayers et al., 2021). Our analyses focused on the basal angiosperms—the Amborellaceae and Nymphaceae families—20 eudicot families, Ceratophyllaceae, and nine monocot families (see **Supplementary Figure 1**; https://www.plabipd.de/pubplant_cladogram1.html). The nuclear genomes in our dataset also have at least an available organellar genome (chloroplast, mitochondrial, or both). To enhance our tRNA gene clustering analysis, we incorporated genomes with chromosome-scale assemblies from the ANA, eudicot, and monocot lineages.

A phylogenetic tree was constructed from concatenated *matK* and *rbcL* sequences of each genome (**Supplementary Table 2**) obtained from the NCBI database (Sayers et al., 2021). Alignment and trimming were performed with MAFFT ver. 7.453 (default parameters; Katoh and Toh, 2008) and trimAI ver. 3-2021.11 (with “-strictplus” option; Capella-Gutiérrez et al., 2009), respectively, and the tree was generated using the IQ-TREE web server (Trifinopoulos et al., 2016). Default parameters were used for the IQ-TREE run. The constructed tree was viewed and edited using TreeGraph ver. 2.15.0-887 (Stöver and Müller, 2010) and FigTree ver. 1.4.4 (Rambaut, 2024.).

### 2.2 tRNA gene detection in plant genomes and alignment of tRNA genes and introns

For nuclear genomes, tRNAscan-SE ver. 2.0.9 (with “-Hy” option) was used for the detection of tRNA genes, or tDNAs, and the primary results were parsed with the post-filtering tool EukHighConfidenceFilter (with “-r” option) of the tRNAscan-SE package listing the high-confidence sets of tDNAs most likely to be involved in ribosomal translation (Chan et al., 2021). To ensure only nuclear tDNAs are detected, we checked each nuclear genome FASTA file and manually removed chloroplast and mitochondrial sequences that were found. The number of high-confidence, intron-containing, and unique tDNA sequences were tabulated for each tRNA isoacceptor of each genome. The “-O” and “-Hy” options were used to detect tRNA genes from chloroplast and mitochondrial genomes. To visualize the overall tRNA gene content in our dataset, heatmaps were generated using the superheat R package (Barter and Yu, 2017). Linear regression analyses were also performed using the built-in lm function in R (R Core Team, 2021; ver. 4.4.2), which was based on the works of Chambers (1992) and Wilkinson and Rogers (1973). We considered p-values lower than 0.05 to be statistically significant.

All the nuclear genomes used for tRNA gene detection were found to have at least one intron-containing tRNA^eMet^ and tRNA^Tyr^ gene. Intronic sequences of these tRNA isoacceptors (extracted using an in-house Perl script) were separately aligned for each of the eudicot, monocot, and ANA lineages to identify conserved nucleotide bases as well as similarities and differences between the consensus intronic sequences of each lineage. Alignment was performed using Multalin ver. 5.4.1 (Corpet, 1988) with the following parameters: “symbol comparison table—DNA-5-0,” “gap penalty at extremities—both,” and “one iteration only—no.” Alignments were then manually modified, if necessary, using AliView ver. 1.21 (Larsson, 2014). Sequence logo plots for the ANA, eudicot, and monocot tRNA^eMet^ and tRNA^Tyr^ intronic sequences were then separately generated using WebLogo 3 (Crooks et al., 2004).

### 2.3 Analysis of tRNA gene regulatory elements and conservation of tRNA species

Sequences 50 and 300 bases immediately upstream and 50 bases immediately downstream of each tDNA sequence were extracted from each genome with the toolkit TBTools (Chen et al., 2020). PlantCARE (Lescot et al., 2002), a database for cis-acting plant regulatory elements database, was utilized to search for TATA-box motifs in tDNA upstream sequences. Other regulatory elements, such as the upstream CAA triplet and the downstream poly(T) signals, were searched through command-line text manipulation. On the other hand, intragenic regulatory elements (A and B boxes) were manually extracted from the alignment of tRNA genes for each isoacceptor and lineage. Sequence logo plots showing upstream A/T content and intragenic A/B box motifs were generated using WebLogo 3 (Crooks et al., 2004).

Command-line BLASTn was used with default settings to compare the high-confidence tRNA gene set of *Amborella trichopoda* with the high-confidence tRNA gene sets of the rest of the nuclear genomes following the procedure of Tang et al. (2009). From this search, one tRNA^Ala-AGC^ species from *A. trichopoda* was found to be identical in the other 68 nuclear genomes, and the secondary sequence of this tDNA was visualized using the RNAfold web server (Institute for Theoretical Chemistry RNAfold web server). This discovery prompted us to investigate the secondary structure conservation of all nuclear tRNA^Ala-AGC^ sequences further using structural alignment and single covariation analysis. Consensus tRNAAla-AGC secondary structures for each lineage were generated using RNAalifold (Bernhart et al., 2008).

Following Tourasse and Darfeuille’s (2020) procedure, structural alignment was performed with MAFFT ver. 7.511 (Katoh and Toh, 2008) in the X-INS-i mode. These structural alignments were then analyzed by single covariation analysis through the web-based version of R-chie (Lai et al., 2012). Before single covariation analysis, a reference secondary structure was generated for tRNA^Ala-AGC^ by uploading the tRNA^Ala-AGC^ sequence into the Mfold web server (Zuker, 2003). For eudicots, monocots, and ANA, the reference secondary structures are from *A. thaliana*, *O. sativa*, and *N. colorata*, respectively. With these reference secondary structures, a single covariation analysis was performed in R-chie by mapping the structures onto the alignments (Tourasse and Darfeuille, 2020). Results were visualized with arc diagrams (with colors representing the various covariation scores) superimposed on the corresponding multiple sequence alignments allowing for the simultaneous comparison of secondary structures and sequences (Lai et al., 2012).

### 2.4 Analysis of tRNA gene clustering

We considered tDNAs to be clustered if at least three tDNAs are within 1 kb of each other (a density of ≥3 tDNAs/kb). The “merge” function of BEDTools was used to obtain a list of clustered tDNAs (Quinlan and Hall, 2010). The BED files for each nuclear genome were created from their respective GFF3 files, which were generated by converting each EukHighConfidenceFilter output file to GFF3 format using an in-house Perl script. Long tDNA clusters with more than 10 repeated tRNA gene units were visualized using the ChromoMap R package (Anand and Rodriguez Lopez, 2022).

### 2.5 Inferring tRNA gene duplication and loss events

To infer and gain insights into what duplication or loss events may have transpired in certain tRNA isoacceptors throughout the evolution of flowering plants, Notung ver. 2.9.1.5 (Chen et al., 2000; Zmasek and Eddy, 2001; Durand et al., 2006; Vernot et al., 2007; Stolzer et al., 2012; Darby et al., 2017) was used. This inference was made in Notung by reconciling the manually prepared gene and species trees.

A separate gene tree was created for tRNA^Pro^, tRNA^Ile^, and tRNA^Ala-AGC^. All tDNA sequences of the specific isoacceptor were aligned using the Clustal Omega server to create a gene tree (Madeira et al., 2022). After converting the generated ClustalW files into the MEGA format, a maximum likelihood tree was generated using the MEGA11 software (Tamura et al., 2021) with the following parameters: “test of phylogeny—bootstrap method,” “no. of bootstrap replications—100,” “model/method—Jukes–Cantor model,” “rates among sites—uniform rates,” “gaps/missing data treatment—partial deletion,” “site coverage cutoff (%)—95,” “ML heuristic method—Nearest-Neighbor-Interchange (NNI),” “initial tree for ML—make initial tree automatically,” and “branch swap filter—very strong.” These parameters were based on the protocol of Mohanta and Bae (2017). The species tree, on the other hand, was based on the phylogenetic tree made by Janssens et al. (2020). Plant genomes in this study that were missing in the said tree were manually added, the placements of which were based on the cladogram found in the Published Plant Genomes website (https://www.plabipd.de/plant_genomes_pa.ep; Usadel Lab Published plant genomes). These trees were labeled and rerooted via the phylogenetic tree viewer FigTree ver. 1.4.4 (Rambaut, 2024).

### 2.6 Analysis of organellar tRNA genes

To visualize the tRNA gene organization in chloroplast and mitochondrial genomes, gene maps were created using the online tool MG2C ver. 2.1 (Chao et al., 2021). The BED file outputs of tRNAscan-SE were used to determine the tRNA gene locations in the respective organellar genome.

## 3 Results

Plants with sequenced chloroplast, mitochondrial, or nuclear genomes (**Supplementary Table 1**) were used for the comparative analysis of tRNA gene content, structure, and organization. *Aquilegia coerulea* and *Acorus americanus* were included in the analysis as these are members of the basal-most eudicot clade and the sister lineage to all other monocots (Filiault et al., 2018; Givnish et al., 2018), respectively. *Amborella trichopoda*, *Nymphaea colorata*, *Nymphaea thermarum*, and *Euryale ferox* under the ANA (Amborellales, Nymphaeales, and Austrobaileyales) clade are sisters to all other angiosperms. *Ceratophyllum demersum* belongs to the species-poor lineage of Ceratophyllales and is sister to eudicots (Yang et al., 2020). Given the phylogenetic positions of these species (**Supplementary Figure 1**), including these sequences will facilitate better comparative analysis of the tRNA gene arrangement and structure in flowering plants.

### 3.1 Nuclear tDNA content

**Figure 1:**
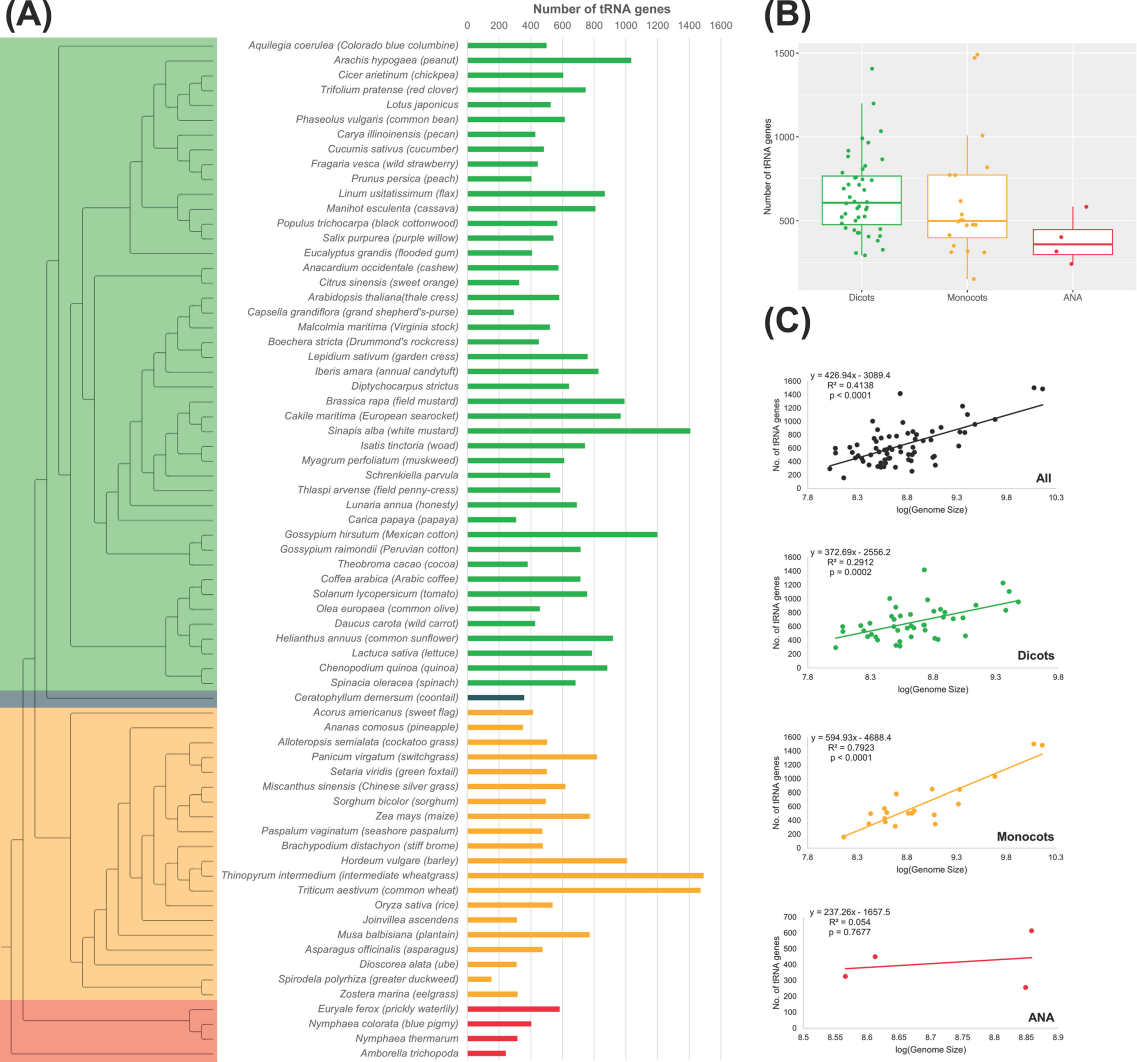
tRNA gene counts in plant nuclear genomes. **(A)** The phylogenetic tree on the left illustrates the evolutionary relationship among the 69 plant genomes examined. In the tree, eudicots are represented in green, monocots in orange, ANA (Amborellales, Nymphaeales, and Austrobaileyales) in red, and *Ceratophyllum* in blue. Adjacent to the tree, a bar graph shows the number of high-confidence tRNA genes found in each species. **(B)** Distribution of tRNA gene counts across different lineages. **(C)** Correlation between genome size and the number of tRNA genes is presented for all genomes as well as for each lineage.

There is a wide variation in the number of tRNA genes, or tDNAs, among the plant genomes studied, even within the same lineage (**Figure 1**). Among these lineages, monocots have the largest range in tDNAs (152–1,491 tDNAs; **Figure 1A**). Compared to the more ancestral ANA clade, several eudicot and monocot genomes have evolved to have a greater number of tDNAs, with some even exceeding 1,400 tDNAs, as in the eudicot *Sinapis alba* (n = 1,407) and the monocots *Thinopyrum intermedium* (n = 1,491) and *Triticum aestivum* (n = 1,472). On the other hand, *E. ferox* had the highest tDNA count of 583 among the ANA species studied (**Figures 1A, B**). *Spirodela polyrhiza* had the smallest number of tDNAs at 152 between the eudicots and monocots. Regarding the number of tDNAs, no general pattern was observed within the eudicots and monocots suggesting that lineage does not influence the number of tDNAs. Genome sizes are also not correlated with the number of tDNAs (**Figure 1C**), as there is a low correlation between genome size and tRNA gene count in our angiosperm dataset (R^2^ = 0.41, p-value <0.0001). Grouping the plants into their respective lineage showed that eudicots have the least correlation (R^2^ = 0.29, p-value = 0.0002), while the monocots showed a relatively high correlation (R^2^ = 0.79, p-value <0.0001). At least for the monocot lineage, one can expect an increased number of tDNAs with a larger genome size. On the other hand, since the linear regression for ANA has a very high p-value (0.7677; likely due to having only four data points), we cannot make conclusions regarding the correlation between genome size and tRNA gene count in the ANA lineage.

**Figure 2:**
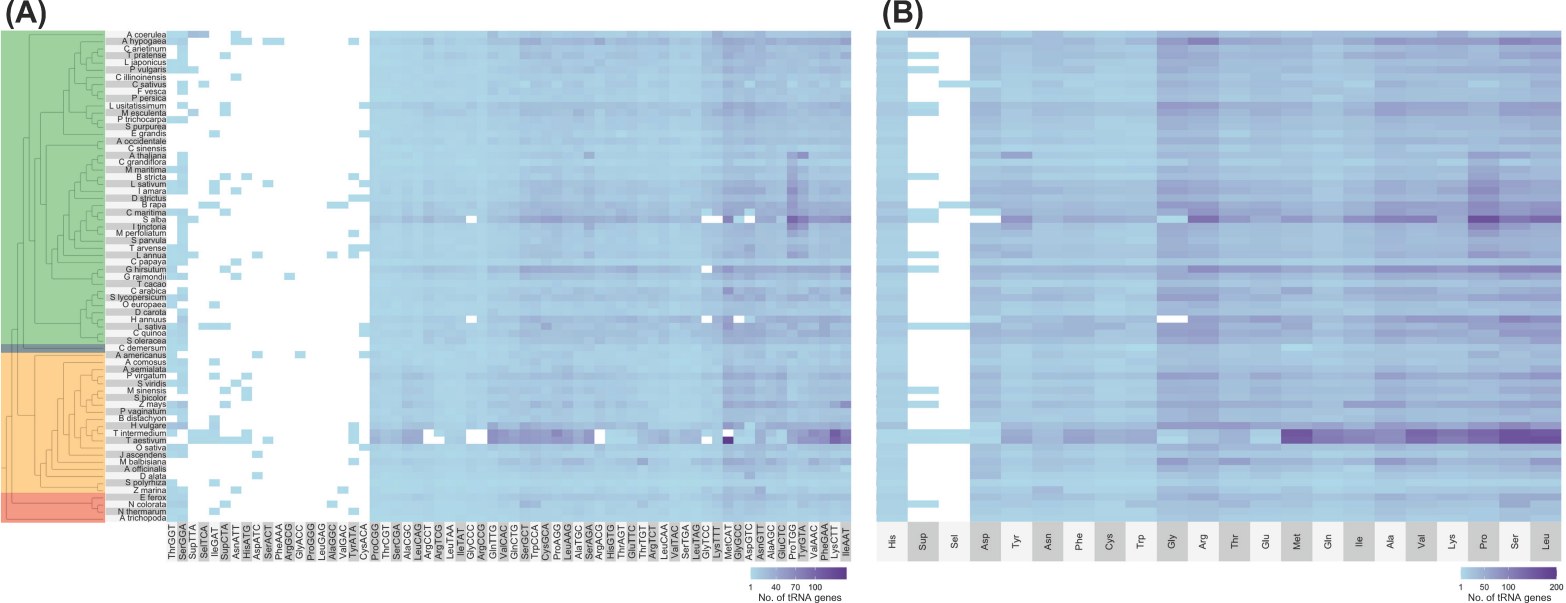
Number of tRNA isoacceptor genes found in plant nuclear genomes. Alongside the heatmap, which displays the number of tRNA genes categorized by **(A)** isoacceptors and **(B)** isotypes, is the exact phylogenetic tree shown in **Figure 1A**. The color coding indicates different groups: green represents eudicots, orange denotes monocots, red signifies ANA (Amborellales, Nymphaeales, and Austrobaileyales), and blue corresponds to *Ceratophyllum*. In the heatmap, white shows that no tRNA gene was found. Refer to **Supplementary File 1** for the tRNA gene counts of all plant genomes examined.

No distinct patterns can also be observed between lineages regarding tRNA isoacceptor content (**Figure 2** and **Supplementary File 1**). The most abundant tRNA isotypes include tRNA^Ala^, tRNA^Pro^, tRNA^Ser^, tRNA^Arg^, and tRNA^Leu^. All genomes, however, lacked tRNA^Pro-GGG^ and tRNA^Leu-GAG^ tDNAs, while tRNA^Gly-ACC^, tRNA^Arg-GCG^, and tRNA^Phe-AAA^ tDNAs were each found in only one genome (*A. americanus*, *Gossypium raimondii*, and *Arachis hypogaea*, respectively; **Figure 2A**). Out of the six tRNA isoacceptors for tRNA^Arg^, *T. aestivum* only contained tRNA^Arg-TCT^ (**Figure 2A**). At the same time, *Helianthus annuus* and *S. alba* completely lacked a nuclear tRNA^Gly^ and tRNA^Asp^, respectively (**Figure 2B**).

**Figure 3:**
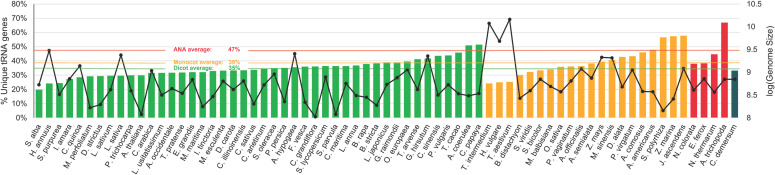
Percentage of unique tRNA gene sequences identified in the nuclear genomes of various plants. Each bar represents the proportion of unique tRNA gene sequences relative to the total number of tRNA genes within each genome. The bars are color coded according to plant lineages: green for eudicots, orange for monocots, red for ANA (Amborellales, Nymphaeales, and Austrobaileyales), and blue for *Ceratophyllum*. Additionally, a second y-axis displaying genome sizes is indicated by solid black lines. A horizontal line representing the average percentage for each major lineage is also included for reference.

On average, less than half of all tRNA genes of each lineage are unique (**Figure 3**). Specifically, 35%, 39%, and 47% of the total tDNAs are unique in the eudicot, monocot, and ANA genomes, respectively. The more ancestral ANA clade had higher percentages of unique tDNA sequences in general, with *A. trichopoda* having the highest at 67%. The more recent lineages, eudicots and monocots, showed a general decrease in tRNA gene uniqueness suggesting a higher prevalence of tRNA gene duplications in these lineages.

All the plant genomes analyzed in this study have intron-containing tRNA^eMet^ and tRNA^Tyr^ (**Supplementary Figures 2–3**). The mean length of these introns is similar for all lineages (**Table 1**), though there are extreme outliers. Five monocot tRNA^eMet^ introns had lengths ranging from 59 to 86 bp, three of which are in the *T. intermedium* genome (**Supplementary Table 3**). On the other hand, two long tRNA^Tyr^ introns were found in the *Miscanthus sinensis* genome (172 and 64 bp in size, respectively), while two identical 85-bp tRNA^Tyr^ introns were each found in the *G. hirsutum* and *G. raimondii* genomes (**Supplementary Table 3**). Aligning all tRNA^eMet^ and tRNA^Tyr^ introns reveals a modest conservation in the former and a relatively lesser conservation in the latter. For tRNA^eMet^, a GCT motif at the start of the intron and a GAGT motif near the end appear to be conserved in angiosperms (**Supplementary Figure 2**). For tRNA^Tyr^, a CAG motif around the middle of the intron appears to be the only relatively conserved residue (**Supplementary Figure 3**). Although rare, introns were also found in non-Met and non-Tyr tDNAs (**Table 2**). While most tRNA isotypes had at least one intron-containing tDNA, no intron-containing tRNA^Ala^, tRNA^Asp^, and tRNA^His^ were found in any plant nuclear genomes studied.

**Table 1:** Mean intron lengths of tDNA^eMet^ and tDNA^Tyr^ in plant nuclear genomes.

Lineage	Mean intron length (bp)	
	tDNA^eMet^	tDNA^Tyr^
ANA	12.16	17.24
Dicot	11.49	14.54
Monocot	14.44	14.11

**Table 2:** Detected non-Met and non-Tyr intron-containing tDNAs in plant nuclear genomes.

Isotype	Genome(s) (no. of intron-containing tDNAs found)
tRNA^Ala^	None
tRNA^Gly^	*M. balbisiana* (1)
tRNA^Pro^	*P. virgatum* (2)
tRNA^Thr^	*L. annua* (1), *H. vulgare* (1), *T. intermedium* (3)
tRNA^Val^	*I. tinctoria* (1), *T. aestivum* (1), *T. intermedium* (1), *Z. mays* (1)
tRNA^Ser^	*C. grandiflora* (1), *P. vaginatum* (2)
tRNA^Arg^	*H. vulgare* (1), *A. trichopoda* (1)
tRNA^Leu^	*G. hirsutum* (1), *A. semialata* (1), *T. aestivum* (4), *T. intermedium* (3)
tRNA^Phe^	*G. hirsutum* (9), *G. raimondii* (3), *P. virgatum* (1), *T. intermedium* (1)
tRNA^Asn^	*I. amara* (2), *L. usitatissimum* (1)
tRNA^Lys^	*B. rapa* (1), *I. tinctoria* (2), *L. usitatissimum* (1), *T. aestivum* (3), *T. intermedium* (1)
tRNA^Asp^	None
tRNA^Glu^	*P. virgatum* (3)
tRNA^His^	None
tRNA^Gln^	*P. virgatum* (1)
tRNA^Ile^	*P. virgatum* (1)
tRNA^Cys^	*P. virgatum* (1), *A. trichopoda* (1)
tRNA^Trp^	*H. vulgare* (1)
tRNA^Sup^	*B. stricta* (1), *L. annua* (1), *M. sinensis* (1)

tRNA^Sup^ refers to the suppressor tRNA.

### 3.2 Nuclear tDNA regulatory regions

Previous analyses of plant tDNA sequences reveal the prevalence of several regulatory elements implicated in the proper recruitment of RNA polymerase III and its efficiency in transcribing nuclear plant tDNAs: an A-/T-rich upstream region (Choisne et al., 1998; Yukawa et al., 2000, 2013; Michaud et al., 2011), upstream TATA-box and CAA motifs (Choisne et al., 1998; Dieci et al., 2006; Michaud et al., 2011; Yukawa et al., 2011, 2013; Soprano et al., 2018), intragenic A and B box promoters (Yukawa et al., 2000, 2013; Michaud et al., 2011; Mitra et al., 2015; Soprano et al., 2018), and downstream stretches of Ts for transcription termination (Yukawa et al., 2000; Arimbasseri and Maraia, 2015; Soprano et al., 2018). In our dataset, the 50 nucleotide sequences immediately upstream of the tDNAs are predominantly A-/T-rich (**Supplementary Figure 4**; **Supplementary Table 4**), and this A-/T-rich upstream region of tDNAs is not dictated by the A/T content of the genome (**Supplementary Figures 4F–I**). This A-/T-rich feature does not extend past the 50 nucleotides upstream of the tDNAs (**Supplementary Figure 5**).

Looking for regulatory elements in the sequences 50 bases upstream of the detected tDNAs revealed a modest percentage of tDNAs, at approximately 22%–32%, having at least one TATA-box motif, and a high percentage, at approximately 78%–82%, having at least one CAA motif (**Table 3**). Narrowing down on the first 10 nucleotides upstream of tDNAs, where CAA triplets usually are found to act as transcription initiation sites in *Arabidopsis* (Yukawa et al., 2011), reduces the percentages to approximately 36%–45% (**Table 3**). On the other hand, sequences 50 nucleotides downstream of all tDNAs revealed a high percentage, at approximately 67%–72%, of having at least one stretch of T residues at least four bases long (**Table 4**). Many of these tDNAs (39%–44%) also contain a “backup” stretch of T residues shortly after the first poly(T) stretch, a common characteristic found in eukaryotic tRNA genes (Braglia et al., 2005; Padilla-Mejía et al., 2009). The lengths of the poly(T) stretches are variable, the longest being 19, 26, and 23 bp for ANA, eudicots, and monocots, respectively (**Supplementary Figure 6**).

**Table 3:** Percentage of tRNA genes possessing upstream TATA-box and CAA motifs.

Lineage	tDNAs (%) with upstream TATA motifs				tDNAs (%) with upstream CAA motifs	
	=1 TATA	=2 TATA	>2 TATA	Total (≥1 TATA)	50 Bases upstream	10 Bases upstream
ANA	15.83 ± 4.95%	4.65 ± 1.08%	1.60 ± 0.87%	22.08 ± 6.13%	78.71 ± 1.76%	36.15 ± 2.21%
Dicot	20.36 ± 2.65%	6.97 ± 1.79%	4.56 ± 1.78%	31.89 ± 5.03%	82.29 ± 3.20%	45.40 ± 4.49%
Monocot	17.66 ± 4.31%	6.22 ± 3.08%	4.16 ± 3.38%	28.04 ± 9.74%	79.97 ± 4.98%	38.08 ± 5.70%

Sequences 50 bases upstream of tDNAs were searched for TATA-box motifs using PlantCARE (Lescot et al., 2002).

**Table 4:** Poly(T) termination signals found downstream of tRNA genes.

	ANA	Dicots	Monocots
Number of tDNAs	1,635	29,463	12,698
%tDNAs with poly(T)s	67.03%	72.77%	69.20%
%tDNAs with backup poly(T)s	39.14%	44.60%	44.32%
Mean length (bp)	5.02	5.20	5.44
Length (bp)	4–19	4–26	4–23

A tDNA contains a backup poly(T) signal if it has at least one additional stretch of T residues downstream of the first determined poly(T) signal.

All tDNAs in the study contained A and B boxes within their coding regions, with varying consensus sequences depending on the tRNA isotype and lineage (**Supplementary Files 2-3**). For A boxes, there are generally conserved T and GG residues at the 5′ and 3′ positions, respectively. In contrast, for B boxes, there are generally conserved GG and CC residues at the 5′ and 3′ positions, respectively. Each tRNA isotype had varying internal A and B box sequences, but the internal sequences were generally conserved among lineages for each isotype. However, some A and B boxes had sequences vastly different from the consensus and are listed separately in **Supplementary Table 5**.

### 3.3 A single conserved tRNA^Ala-AGC^ species

**Figure 4:**
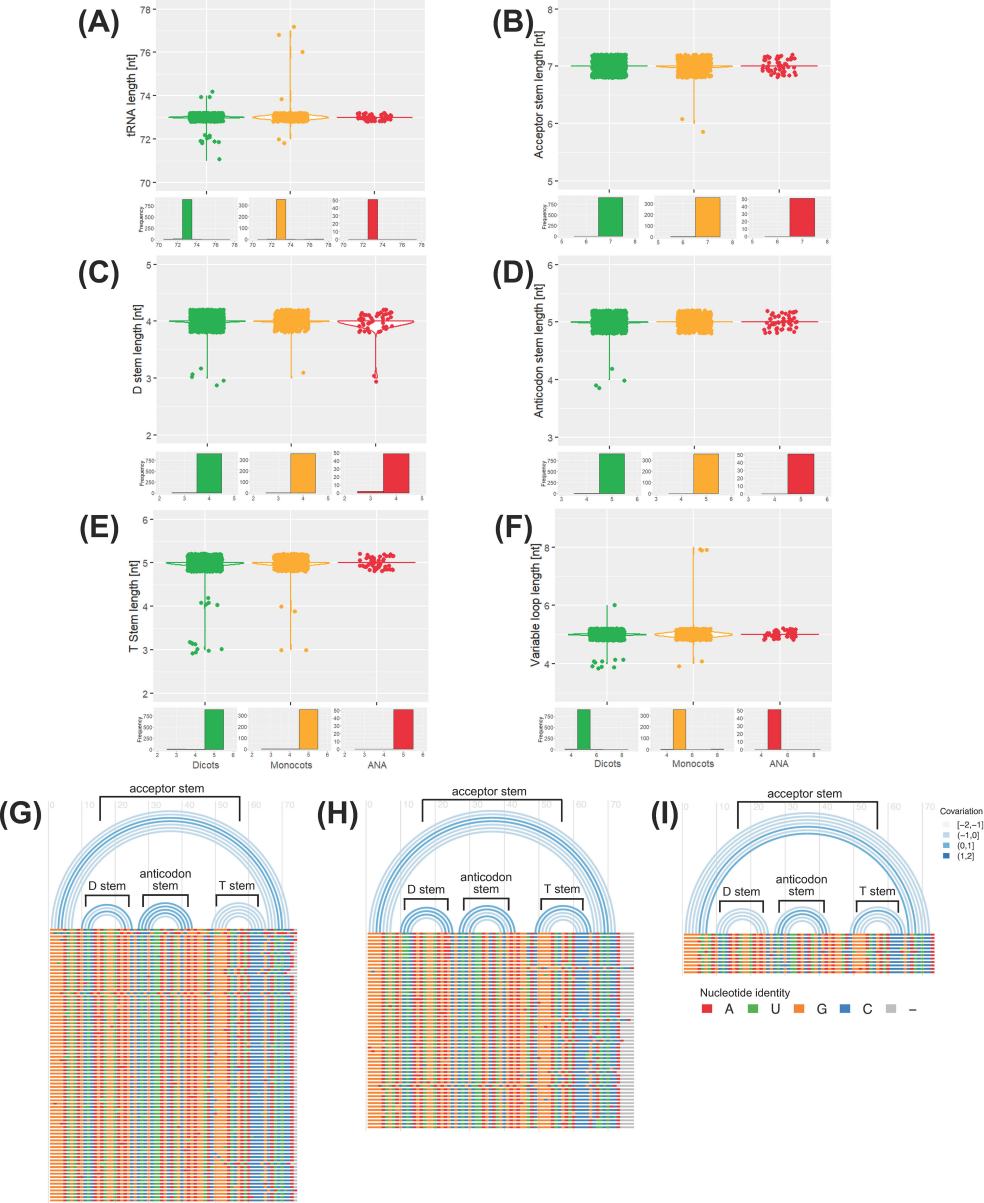
Conservation of the tRNA^Ala-AGC^ secondary structure. The distribution of lengths for various elements of the tRNA^Ala-AGC^ genes across different lineages is displayed: **(A)** tRNA, **(B)** acceptor stem, **(C)** D stem, **(D)** anticodon stem, **(E)** T stem, and **(F)** variable loop lengths for each lineage (green for eudicots, yellow for monocots, red for ANA). Structural representation of tRNA^Ala-AGC^ is also illustrated through arc diagrams for **(G)** eudicots, **(H)** monocots, and **(I)** ANA (Amborellales, Nymphaeales, and Austrobaileyales) generated using R-chie. Horizontal bars below the arcs (colored by nucleotide identity, bottom legend: A is red, U is green, G is orange, C is blue, and gray is a gap) represent the multiple sequence alignment of all unique tRNA^Ala-AGC^ genes of each lineage. Significant arcs corresponding to the different tRNA cloverleaf stems are labeled accordingly. The top legend for **(G)** to **(I)** indicates the covariation of the base pairing between the arches, where a negative and positive covariations indicates no conservation and conservation of base pairings, respectively.

A conserved tRNA^Ala-AGC^ species was detected in our genomic dataset (**Supplementary Figure 7A**; see **Supplementary Figure 8** for consensus structures of other tRNA^Ala^ isoacceptors). Polymorphic tRNA^Ala-AGC^ sequences were also detected (**Supplementary Figure 7B**); thus, we also analyzed the evolution and structural conservation of all detected tRNA^Ala-AGC^ genes. Gene tree and species tree reconciliation via Notung (Chen et al., 2000; Zmasek and Eddy, 2001; Durand et al., 2006; Vernot et al., 2007; Stolzer et al., 2012; Darby et al., 2017) reveals that the evolution of tRNA^Ala-AGC^ in angiosperms is characterized by more gene losses than duplications (253 inferred gene duplications and 586 inferred gene losses; **Supplementary File 4**). The tRNA, cloverleaf stem, and variable loop lengths are generally conserved in the nuclear tRNA^Ala-AGC^ genes in plants (**Figures 4A-F**). Sequence covariation analysis reveals that the base pairing within each cloverleaf stem is not well conserved in tRNA^Ala-AGC^ (**Figures 4G-I**). In general, for all lineages, base pairs (represented by single arcs) show negative covariation, where should a base mutate in one of the stems, its paired base will not likely mutate to preserve the base pairing. An exception is the D-stem of monocot tRNA^Ala-AGC^ genes, whose base pairs or arcs exhibit positive covariation.

### 3.4 Nuclear tDNA clusters

**Figure 5:**
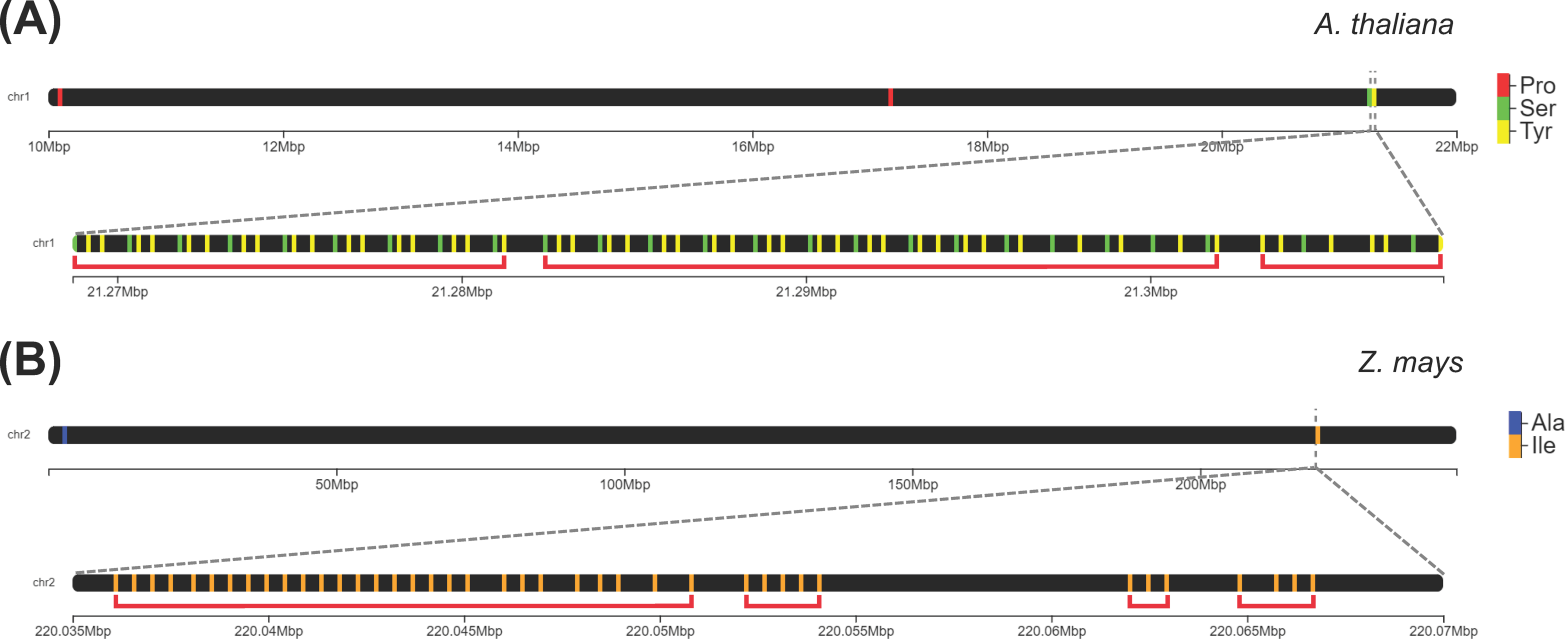
Extensive tRNA gene clusters identified in the genomes of eudicots and monocots. **(A)** In the genome of *Arabidopsis thaliana*, one cluster on Chromosome 1 consists of alternating tRNA^Tyr^ and tRNA^Ser^ genes. **(B)** In *Zea mays*, there are clusters on Chromosome 2 that are composed of tandem repeats of tRNA^Ile^ genes. Each red bracket indicates a distinct gene cluster.

We classified a group of tDNAs as a cluster if they have a density of at least three tDNAs per kilobase of a genomic region. The majority of eudicot genomes (40 out of 44) and only a modest percentage of monocot (13 out of 20) and ANA genomes (1 out of 4) contained at least one tDNA cluster using this criterion. The proportion of tRNA genes that are clustered is generally deficient among angiosperms (5% and 3% in eudicots and monocots, respectively), the highest being 20% in *Musa balbisiana*, followed by *A. thaliana* and *Isatis tinctoria* (19% and 16% clustered tDNAs, respectively). In the eudicot, monocot, and ANA lineages, 324, 103, and 2 tDNA clusters were identified, respectively. The following tDNA clusters were detected in our analysis: stretches of at least three tRNA^Pro^ (to as many as 10) found in *Ceratophyllum*, eudicots, and monocots; stretches of alternating tRNA^Tyr^ and tRNA^Ser^ found only in eudicots (**Figure 5A**); and a stretch of 28 tRNA^Ile^ found only in the monocot *Zea mays* (**Figure 5B**). Since these clusters may be linked to tRNA gene duplication, gene duplication events of tRNA^Pro^ and tRNA^Ile^ were inferred using Notung. Reconciliation of each tRNA gene tree with the species tree reveals that the tRNA^Pro^ and tRNA^Ile^ genes underwent 592 and 479 gene duplication events, respectively (**Supplementary Files 5–6**).

### 3.5 Organellar tDNA content, organization, and structure

**Figure 6:**
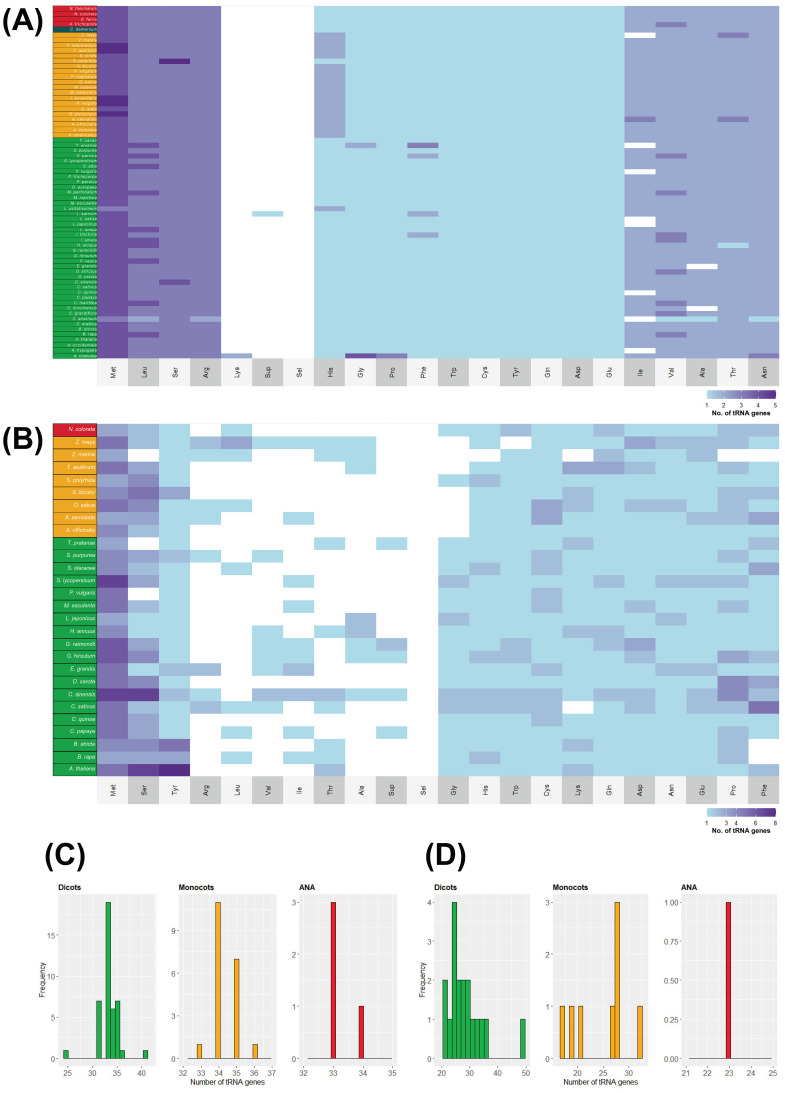
tRNA isotypes and gene numbers in plant organellar genomes. The heatmap illustrates the number of tRNA isotypes found in **(A)** chloroplast and **(B)** mitochondrial genomes of plants. Species names are color coded according to their lineage: green represents eudicots, orange denotes monocots, red indicates ANA (Amborellales, Nymphaeales, and Austrobaileyales), and blue signifies *Ceratophyllum*. Additionally, the distribution of tRNA gene counts is displayed for **(C)** chloroplast and **(D)** mitochondrial genomes.

In contrast to their nuclear counterparts, chloroplast and mitochondrial genomes show slight variation in their tDNA numbers. The tRNA isotype content of plastomes and mitogenomes also shows slight variation among the different plant lineages (**Figure 6**). The relative abundance of each isotype is almost uniform in all the surveyed chloroplast genomes, while it varies in all the surveyed mitogenomes. Apart from *A. coerulea*, all the surveyed plastomes lack a tRNA^Lys^ gene. Plastomes typically have 31–36 tDNAs regardless of lineage (except for *Cicer arietinum* and *A. coerulea*, with 25 and 41 chloroplast tDNAs, respectively). On the other hand, mitogenomes typically have 17–36 tDNAs and more variable tDNA content than the plastomes. The eudicot *Citrus sinensis* has 49 mitochondrial tDNAs.

Although *H. annuus* lacked nuclear tRNA^Gly^ genes (**Figure 2B**), one tRNA^Gly-GCC^ sequence was detected in its chloroplast and mitochondrial genomes. *S. alba*, which lacked a nuclear tRNA^Asp^ (**Figure 2B**), also had one detected tRNA^Asp-GTC^ in its chloroplast genome. While *S. alba* currently does not have an available mitochondrial genome, the closely related *Brassica rapa* (**Supplementary Figure 1**) also has one tRNA^Asp-GTC^ gene in its mitogenome.

The tRNA gene organization in the plastomes and mitogenomes reflects the evolutionary conservation of these organellar genomes. Plastomes of flowering plants show a relatively conserved tRNA gene organization, with some rearrangements in some species (**Supplementary Files 7–9**). Their mitogenomes, on the other hand, show little conservation in their tRNA gene organization (**Supplementary Files 10–12**).

Unlike their nuclear counterparts, sequences immediately upstream of organellar tDNAs do not exhibit a distinct, consistent pattern. Though chloroplast tDNAs still have predominantly A-/T-rich upstream sequences (**Supplementary Figures 9** and **10**), the same cannot be said about mitochondrial tDNAs, which exhibit much less conservation than chloroplast tDNAs (**Supplementary Figures 11 and 12**).

## 4 Discussion

A narrow range of nuclear tDNA numbers in angiosperms (500–600 tDNAs between five angiosperm genomes) had been previously reported (Michaud et al., 2011), and extending the coverage to 69 angiosperm genomes resulted in a broader range in the number of nuclear tDNAs that were detected (approximately 150–1,500 tDNAs; **Figure 1A**). This tDNA range is comparable to that reported by Bermudez-Santana et al. (2010), although their range also included tRNA pseudogenes (432–1,290 tDNAs between seven land plant genomes). In addition, the green algae *Volvox carteri* and *Chlamydomonas reinhardtii* were reported to have 1,051 (including tRNA pseudogenes) and 256 tDNAs, respectively (Bermudez-Santana et al., 2010; Michaud et al., 2011). Therefore, nuclear genomes from the green lineage can have tDNAs as few as 150 or as many as 1,500. This variation in plant nuclear tDNA numbers is relatively small compared to other eukaryotes. Tetraodontiformes have approximately 700 tDNAs, while the related zebrafish, *Danio rerio*, has approximately 20,000 (Bermudez-Santana et al., 2010). Concurrently, in mammals, old-world monkeys and apes had 496–736 tDNAs, while cows and rats exceeded 100,000 tDNAs (Bermudez-Santana et al., 2010); a reannotation of the cow tRNAs showed that the majority of these putative tDNAs include tRNA-like sequences (Theologis et al., 2000; Tang et al., 2009). In nuclear eukaryotic genomes, the number of tDNAs can vary even within species of the same lineage or clade. Indeed, ANA, eudicot, and monocot genomes have varying numbers of nuclear tDNAs, and no lineage-specific pattern could be observed (**Figure 1A**).

The varying genome sizes in eukaryotes, including plants, could explain this variation in the number of tDNAs. While earlier studies suggested a strong correlation among plants, with *Arabidopsis* being an outlier (Bermudez-Santana et al., 2010; Michaud et al., 2011), our data showed a weak overall correlation in the 69 angiosperm genomes studied (R^2^ = 0.41, p-value <0.0001; **Figure 1C**), especially for the eudicot lineage, with an R-squared value of 0.29 (p-value = 0.0002). More recent studies have similarly reported a weak correlation among plants (Mohanta et al., 2020; Santos and Del-Bem, 2023). However, this was not the case for the monocot lineage, which exhibited a strong correlation (R^2^ = 0.79, p-value <0.0001; **Figure 1C**). A strong correlation between the monocot genome sizes and the number of tDNAs had been previously reported (Planta et al., 2022).

At least for the eudicot genomes, a likely explanation is related to the unique case of *Arabidopsis* (Michaud et al., 2011). A weak correlation between the number of tDNAs and genome size was initially shown in *A. thaliana*, with an R-squared value of 0.16. This correlation contrasted with the other analyzed plant genomes, which all had moderate to high R-squared values. Compared to four other angiosperms (*Medicago truncatula*, *Populus trichocarpa*, *Oryza sativa*, and *Brachypodium distachyon*) and one green alga (*C. reinhardtii*), *A. thaliana* had a higher number of tDNAs in each chromosome (Michaud et al., 2011). Except for *A. thaliana*, the other genomes had at most only two tDNAs per Mb of chromosome. Chromosomes 2–5 of *A. thaliana* had approximately four tDNAs per Mb, while Chromosome 1 had eight tDNAs per Mb of chromosome (Michaud et al., 2011). This unusually high number of tDNAs in Chromosome 1 of *A. thaliana* is largely due to the existence of two large tDNA clusters in this chromosome: tandem repeats of 27 tRNA^Pro^ and tandem repeats of 27 tRNA^Tyr^–tRNA^Tyr^–tRNA^Ser^ (Theologis et al., 2000). These clusters, indicative of gene duplications (Theologis et al., 2000; Bermudez-Santana et al., 2010), are likely the cause of the weak correlation between the tDNA number and genome size of *A. thaliana*. Indeed, removing the tRNA isotypes involved in the two identified clusters (tRNA^Pro^, tRNA^Ser^, and tRNA^Tyr^) increased the R-squared value in *A. thaliana* from 0.16 to 0.70 (Michaud et al., 2011).

Similarly, the weak overall correlation found in the angiosperm genomes in this study might be explained by the prevalence of gene duplication events. This is likely the case, given that generally less than half of all tDNAs of each lineage were found to be unique (**Figure 3**). This may also explain the observation that plants, alongside vertebrates, appear to have higher tDNA count and redundancy compared to other organisms (Santos and Del-Bem, 2023). However, this does not explain why the monocots showed a strong correlation between tDNA number and genome size (R^2^ = 0.79; **Figure 1C**), as opposed to weaker correlation observed in eudicot genomes (R^2^ = 0.29). The key difference may lie in the existence of tDNA clusters, like the ones found in *A. thaliana*.

We considered tRNA genes to be clustered if at least three tDNAs were within 1 kb of each other. Using this criterion, 324 (in 40/44 genomes), 103 (in 13/20 genomes), and 2 (in 1/4 genomes) tDNA clusters were identified in eudicots, monocots, and ANA, respectively. Eudicots thus appear to have a stronger tendency toward gene duplication in the form of tDNA clustering compared to the other plant lineages, and this should explain the weaker correlation between tDNA numbers and genome sizes in eudicots compared to those in monocots. While ANA genomes appear to have a weak correlation like eudicots (**Figure 1C**), they had very few tDNA clusters. It is very likely that the linear regression model did not properly represent the correlation between ANA genome size and tRNA gene count due to the high p-value (0.7677). This may also be a result of our stricter criteria for tDNA clustering compared to other tRNA studies (Bermudez-Santana et al., 2010; Morgado and Vicente, 2019), which considered clusters as having at least two tDNAs within 1 kb of each other.

We identified tDNA clusters in Chromosome 1 of *A. thaliana*, similar to the two large clusters that were previously reported (Michaud et al., 2011) as follows: (i) consecutive tRNA^Pro^ clusters, adding up to 25 tandem repeats of tRNA^Pro^, and (ii) consecutive tRNA^Tyr^-tRNA^Ser^ clusters, comprising a long stretch of alternating tRNA^Tyr^ and tRNA^Ser^ genes. Unlike previously reported, these stretches of tRNA^Tyr^ and tRNA^Ser^ genes were not strictly tandem repeats of the triplet tRNA^Tyr^–tRNA^Tyr^–tRNA^Ser^. The difference in the size and order of these clusters compared to those found by Theologis et al. (2000) is likely due to the updated genome assembly for *A. thaliana*. These tRNA^Pro^ and tRNA^Tyr^–tRNA^Ser^ clusters were also found in other plant genomes. Most eudicots (34 out of 44 genomes, including *A. thaliana*), a few monocots (6 out of 20 genomes), and *C. demersum* were also found to have stretches of tRNA^Pro^ genes. On the other hand, a long stretch of alternating tRNA^Tyr^ and tRNA^Ser^ genes was also found in eight other eudicot genomes (*Boechera stricta*, *Diptychocarpus strictus*, *Iberis amara*, *I. tinctoria*, *Lunaria annua*, *Lepidium sativum*, *Malcolmia maritima*, and *Myagrum perfoliatum*). This tRNA^Tyr^–tRNA^Ser^ tDNA cluster was not found in any other monocot or ANA genome. Another tDNA cluster detected is a tandem repeat of 28 tRNA^Ile^ found exclusively in Chromosome 2 of *Z. mays*. Among the clusters found in this study, this is the longest in size. Interestingly, this cluster is followed by three more clusters consisting purely of tRNA^Ile^ (5x tRNA^Ile^, 3x tRNA^Ile^, then 4x tRNA^Ile^) within the same chromosome.

It remains to be seen whether these tDNA clusters serve any biological purpose. tDNA clusters are implicated in genome breakage resulting in genome rearrangement (Rienzi et al., 2009). They are also found to be involved in mobile genetic elements and horizontal gene transfer (Morgado and Vicente, 2019). tDNA clusters are likely dynamic and fragile genomic regions, and this inherent instability might be the reason for the evolution and prevalence of these tDNA clusters rather than being products of positive selection. Moreover, a study on the tDNA clusters of *Arabidopsis* shows that these clusters are predominantly methylated and transcriptionally repressed (Hummel et al., 2020). However, the case of tRNA^Pro^ clusters is intriguing given its frequency among the plant genomes studied.

Proline is found to have diverse roles in plants. They are involved in cell wall and plant growth (Kishor et al., 2015), but the more well-documented function of proline is related to plant stress. In response to different environmental stresses, e.g., drought or water loss, salt, metal, and pathogen attack, plants accumulate proline (Kishor et al., 2005; Verslues and Sharma, 2010; Patriarca et al., 2021; Vujanovic et al., 2022). Being an osmolyte, proline can maintain cellular metabolism and even reduce plant growth in stressful conditions (Maggio et al., 2002; Vujanovic et al., 2022). This physiological response of proline accumulation would involve tRNA^Pro^ activity and could thus be a reason behind the prevalence of tRNA^Pro^ clusters and duplications (**Supplementary File 8**). While these clusters might be initially repressed by methylation (Hummel et al., 2020), the plant stress response could induce the removal of these epigenetic marks, thereby increasing global tRNA^Pro^ transcription levels. To confirm this link, future studies are encouraged to look into the expression profile of these clustered tDNAs in plants. The potential biological functions of these tDNA clusters themselves may also be investigated further by future studies.

Another interesting observation is the apparent lack of certain tRNA isotypes in the nuclear genome of *H. annuus* and *S. alba*, even though their organellar counterparts are present. After further investigation, we found that prior to filtering via EukHighConfidenceFilter, *H. annuus* and *S. alba* had 117 tRNA^Gly^ and 82 tRNA^Asp^ predicted genes, respectively. None of these first-pass tRNA genes had an isotype score that met the cutoff for EukHighConfidenceFilter, which was 95 by default for these two isotypes. The tRNAscan-SE developers emphasized to only change the cutoff values with great caution, as they have already been tested on different large eukaryotic genomes (Chan and Lowe, 2019); thus, throughout our analysis, we opted to keep all default cutoff values unchanged. However, the fact that some of the first-pass tRNA^Gly^ and tRNA^Asp^ genes had scores that were very close to the cutoff value (as close as 94.5) indicates the need to reevaluate these score cutoffs.

To transcribe plant tRNAs, RNA polymerase III (Pol III) is recruited. One of the requirements for its recruitment is a TATA-binding protein (TBP), and the presence of TATA-box motifs upstream of plant tRNA genes is implicated in the efficiency of tRNA transcription (Dieci et al., 2006; Michaud et al., 2011). However, the proportion of angiosperm tDNAs containing such a motif is strikingly low (**Table 3**). Previous studies have similarly reported the lack of TATA-box motifs upstream of many eukaryotic tDNAs (Hamada et al., 2001; Giuliodori et al., 2003; Dieci et al., 2006) as well as the little effect caused by the removal of TATA-box motifs in the transcription of plant tRNA^Leu^ genes (Choisne et al., 1998). For many Pol III-transcribed genes, TBP can be recruited without a specific TATA-like sequence. For these TATA-less genes, recruiting Pol III is instead facilitated by TFIIIC, which binds the DNA via the A and B boxes and recruits TFIIIB, which has a TBP as one of its subunits. TFIIIB recruits Pol III (Choisne et al., 1998; Yukawa et al., 2000; Dieci et al., 2006). This suggests that while some plants prefer the TATA-mediated recruitment of TBP [e.g., *A. thaliana* (Choisne et al., 1998; Hamada et al., 2001)], it may not be preferred or deemed necessary by other organisms that lack conserved TATA-box motifs. Dieci et al. (2006) hinted that the difference between a TATA-box-dependent and a TATA-box-independent organism might be found in their respective transcription machinery. Notably, the intragenic A and B boxes bound by TFIIIC were found in all detected nuclear tRNA genes (**Supplementary Files 2** and **3**). However, this can mainly be explained by the fact that the tRNA D- and T-loops are encoded within these boxes (Galli et al., 1981; Hofstetter et al., 1981; Turowski and Tollervey, 2016) and that the tRNAscan-SE program detects tRNA genes based on the presence of A and B box sequences (Lowe and Eddy, 1997).

The CAA motifs, on the other hand, were found in most angiosperm tDNAs between positions −1 and −50 bp (**Table 3**). Removal of these motifs upstream of plant tDNAs decreased *in vitro* expression levels of these tRNAs (Choisne et al., 1998; Yukawa et al., 2000). While previous studies reported functional CAA motifs to be between −1 and −10 bp in plant tDNAs (Yukawa et al., 2000, 2011; Michaud et al., 2011), more CAA motifs were found when the scope was extended up to −50 bp (**Table 3**). This suggests that transcription start sites (TSS) for many plant tDNAs may be further upstream than others.

The majority of angiosperm tDNAs contained at least one downstream stretch of T residues (**Table 4**), which is expected as it is considered an essential signal used by Pol III for transcription termination (Braglia et al., 2005; Arimbasseri and Maraia, 2015). In eukaryotic tRNAs, this poly(T) signal is commonly found to be approximately four to five bases long (Braglia et al., 2005). Aside from stretches of four to five T residues, there is also an abundance of poly(T) stretches that are 6 to 10 bases long, and those with extreme lengths—19, 26, and 23 bases—were found in the ANA, eudicot, and monocot tDNAs, respectively. While a significant percentage of angiosperm tDNAs do not contain a downstream poly(T) signal (**Table 4**), it is possible that increasing the coverage to 100 or more nucleotides downstream (instead of only 50) will locate more poly(T) signals, backup poly(T) signals, and other poly(T) signals of extreme and variable lengths.

Our results provide a comprehensive overview of the tRNA gene content, structure, and organization of nuclear and organellar angiosperm genomes, utilizing the recent abundance of genomic data enabled by next-generation sequencing technologies. This study can thus supplement further studies on plant tRNA gene function and regulation. The specific function of these tRNA gene clusters and an explanation for the differences in the abundance of several regulatory motifs [e.g., TATA-boxes, CAA motifs, and poly(T) stretches] are some points that may be explored in the future.

## Data Availability

The datasets presented in this study can be found in online repositories. The names of the repository/repositories and accession number(s) can be found in the article/**Supplementary Material**.
