# Supplementary material for: tRNA gene content, structure, and organization in the flowering plant lineage
Source: Front Plant Sci. 2024 Dec 23;15:1486612. doi: 10.3389/fpls.2024.1486612 (PMC11700998; doi:10.3389/fpls.2024.1486612)
Supplement: Supplementary file 1 [file DataSheet1.pdf]

Supplementary File 1. Number of tRNA isoacceptors per genome.

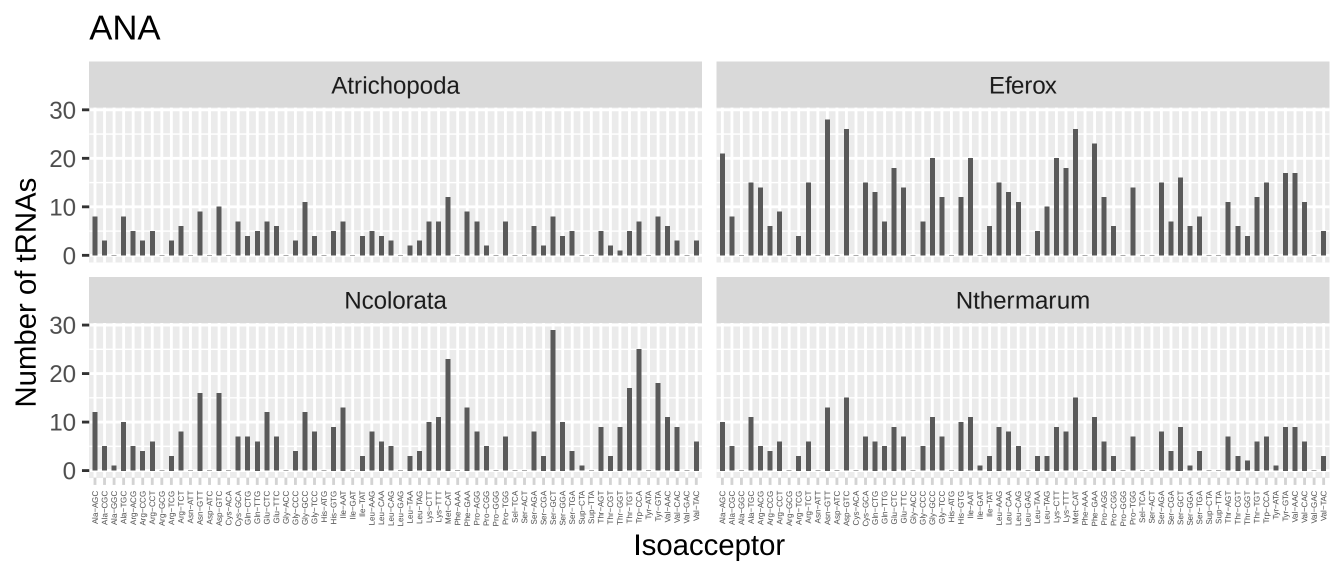

ANA
Atrichopoda
Eferox
Ncolorata
Nthermarum
Number of tRNAs
Isoacceptor

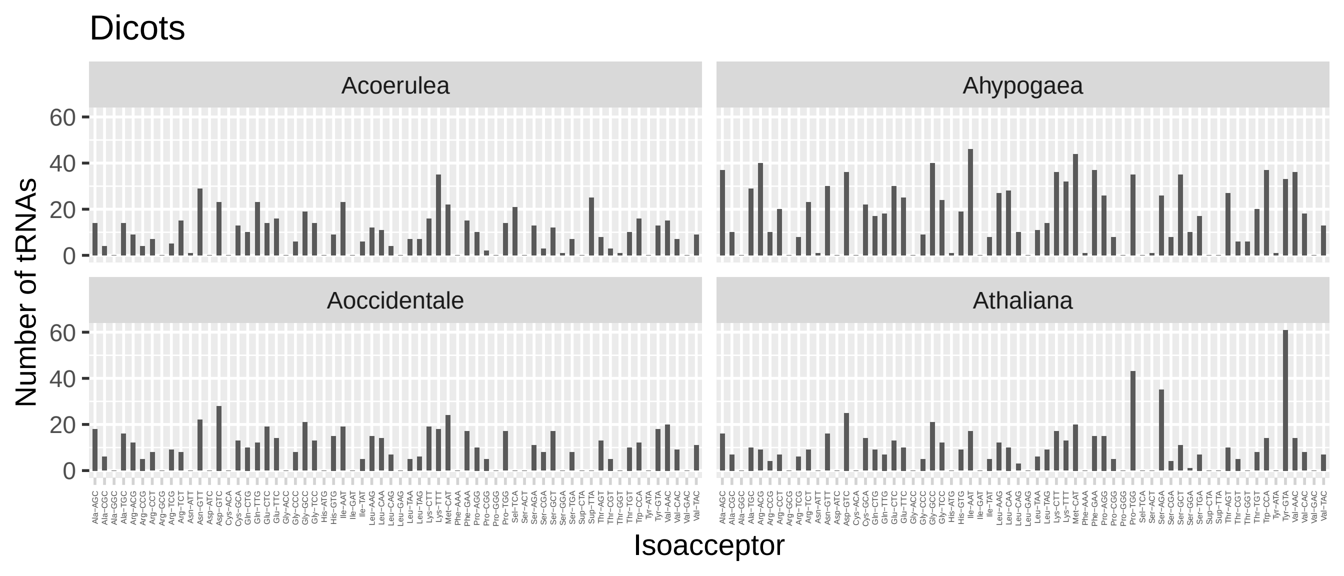

Dicots
Acoerulea
Ahypogaea
Aoccidentale
Athaliana
Number of tRNAs
Isoacceptor
0
20
40
60

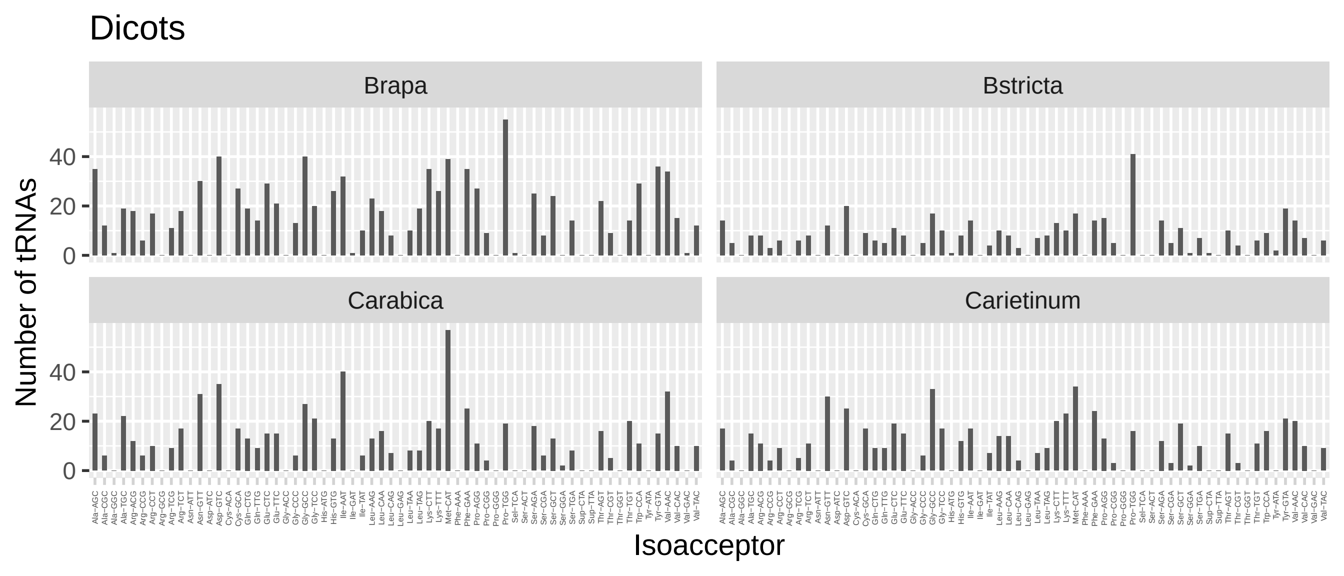
Dicots
Brapa
Bstricta
Carabica
Carietinum
Number of tRNAs
0
20
40
Isoacceptor
Ala-AGC
Ala-CGC
Ala-GGC
Ala-TGC
Arg-ACG
Arg-CCG
Arg-CCT
Arg-GCG
Arg-TCG
Arg-TCT
Asn-ATT
Asn-GTT
Asp-ATC
Asp-GTC
Cys-ACA
Cys-GCA
Gln-CTG
Gln-TTG
Glu-CTC
Glu-TTC
Gly-ACC
Gly-CCC
Gly-GCC
Gly-TCC
His-ATG
His-GTG
Ile-AAT
Ile-GAT
Ile-TAT
Leu-AAG
Leu-CAA
Leu-CAG
Leu-GAG
Leu-TAA
Leu-TAG
Lys-CTT
Lys-TTT
Met-CAT
Phe-AAA
Phe-GAA
Pro-AGG
Pro-CGG
Pro-GGG
Pro-TGG
Sel-TCA
Ser-ACT
Ser-AGA
Ser-CGA
Ser-GCT
Ser-GGA
Ser-TGA
Sup-CTA
Sup-TTA
Thr-AGT
Thr-CGT
Thr-GGT
Thr-TGT
Trp-CCA
Tyr-ATA
Tyr-GTA
Val-AAC
Val-CAC
Val-GAC
Val-TAC

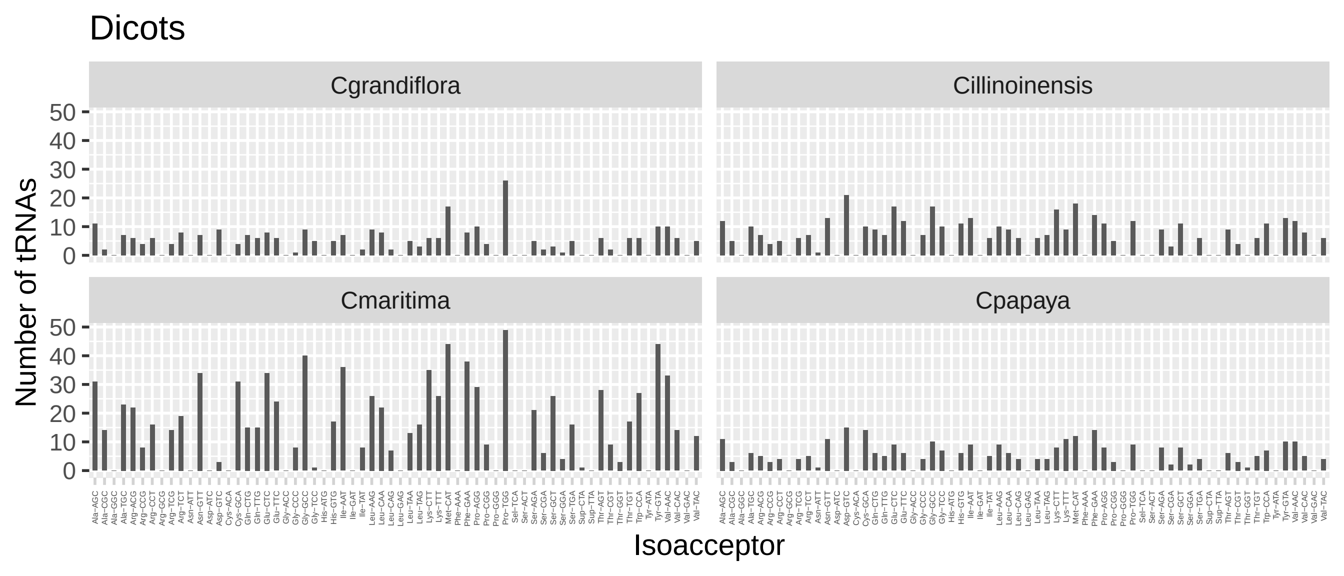

Dicots
Cgrandiflora
Cillinoinensis
Cmaritima
Cpapaya
Number of tRNAs
Isoacceptor

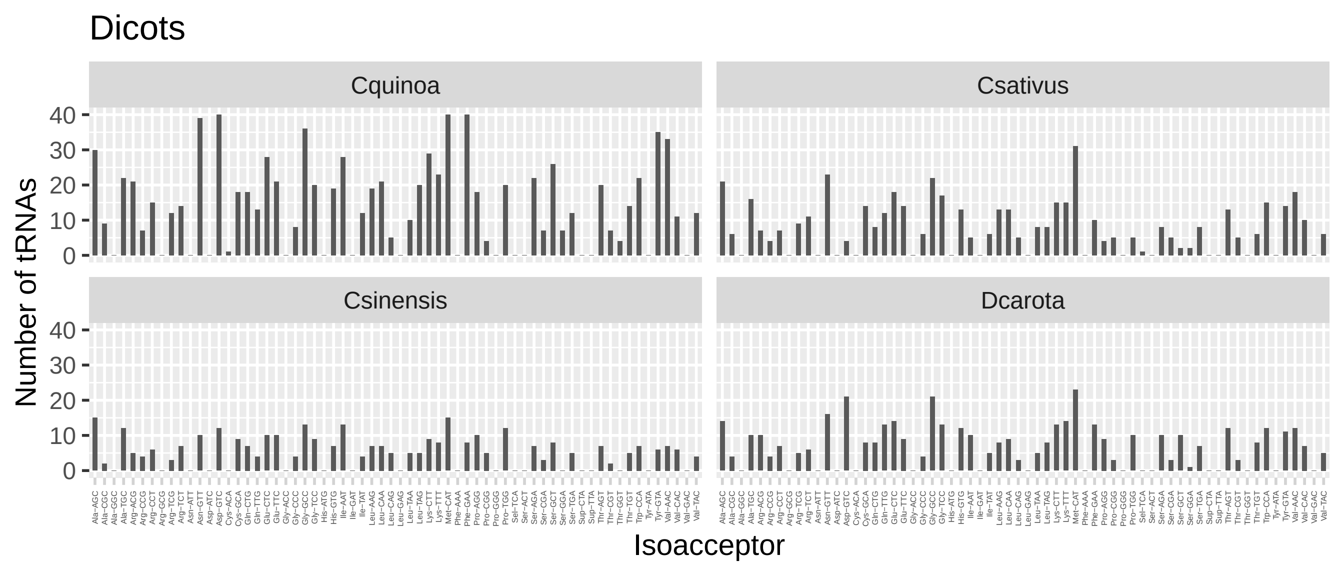
Dicots
Cquinoa
Csativus
Csinensis
Dcarota
Number of tRNAs
Isoacceptor
0
10
20
30
40

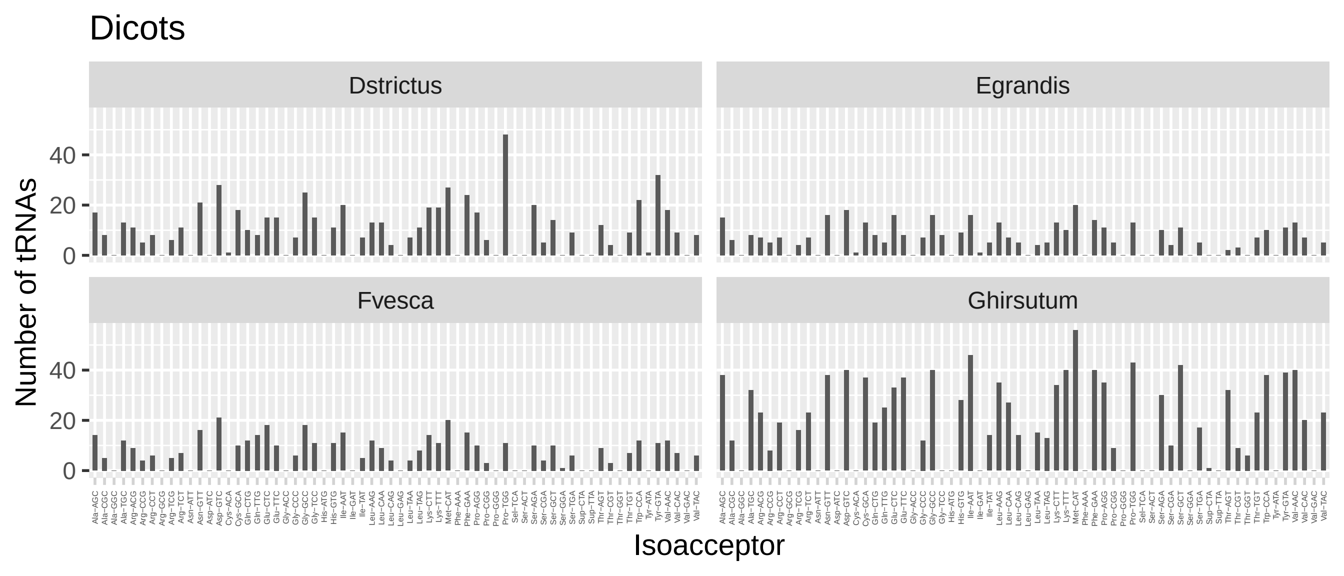
Dicots
Dstrictus
Egrandis
Fvesca
Ghirsutum
Number of tRNAs
0
20
40
Isoacceptor
Ala-AGC
Ala-CGC
Ala-GGC
Ala-TGC
Arg-ACG
Arg-CCG
Arg-CCT
Arg-GCG
Arg-TCG
Arg-TCT
Asn-ATT
Asn-GTT
Asp-ATC
Asp-GTC
Cys-ACA
Cys-GCA
Gln-CTG
Gln-TTG
Glu-CTC
Glu-TTC
Gly-ACC
Gly-CCC
Gly-GCC
Gly-TCC
His-ATG
His-GTG
Ile-AAT
Ile-GAT
Ile-TAT
Leu-AAG
Leu-CAA
Leu-CAG
Leu-GAG
Leu-TAA
Leu-TAG
Lys-CTT
Lys-TTT
Met-CAT
Phe-AAA
Phe-GAA
Pro-AGG
Pro-CGG
Pro-GGG
Pro-TGG
Sel-TCA
Ser-ACT
Ser-AGA
Ser-CGA
Ser-GCT
Ser-GGA
Ser-TGA
Sup-CTA
Sup-TTA
Thr-AGT
Thr-CGT
Thr-GGT
Thr-TGT
Trp-CCA
Tyr-ATA
Tyr-GTA
Val-AAC
Val-CAC
Val-GAC
Val-TAC

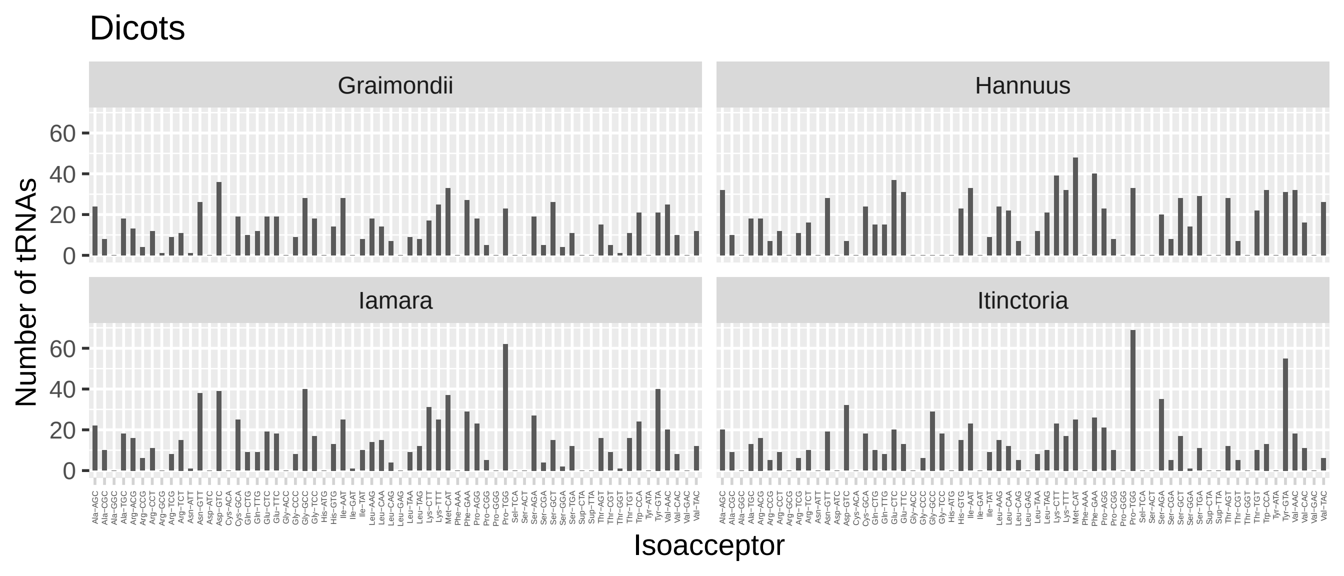

Dicots
Graimondii
Hannuus
Iamara
Itinctoria
Number of tRNAs
Isoacceptor
0
20
40
60

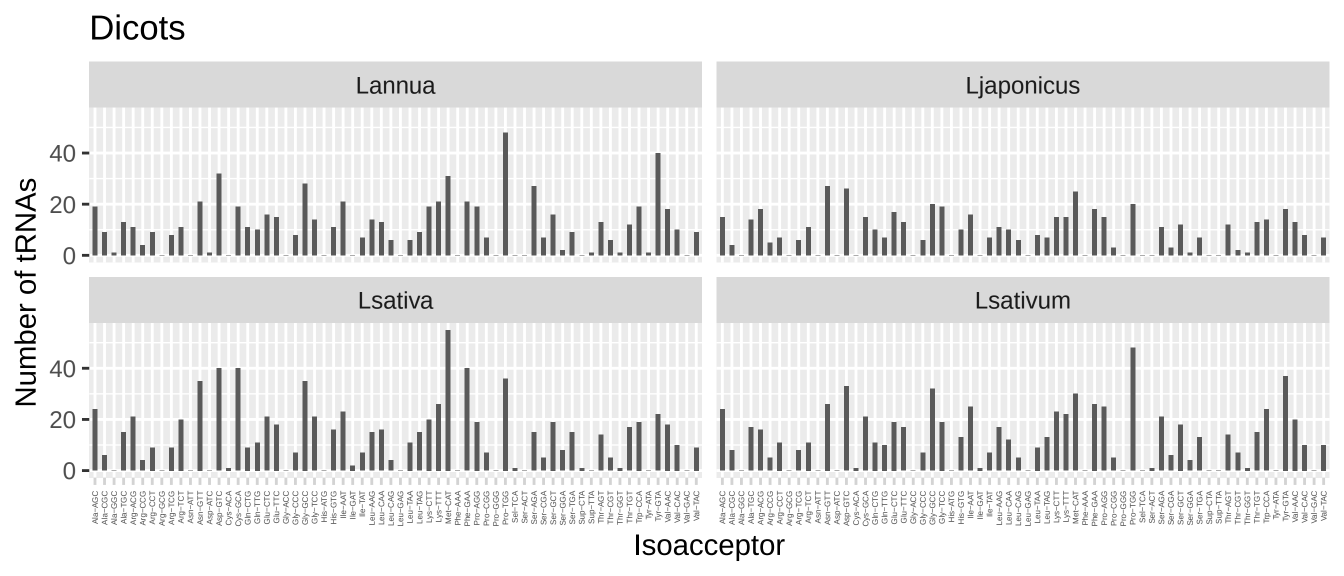
Dicots
Lannua
Ljaponicus
Lsativa
Lsativum
Number of tRNAs
0
20
40
Isoacceptor
Ala–AGC Ala–CGC Ala–GGC Ala–TGC Arg–ACG Arg–CCG Arg–CCT Arg–GCG Arg–TCG Arg–TCT Asn–ATT Asn–GTT Asp–ATC Asp–GTC Cys–ACA Cys–GCA Gln–CTG Gln–TTG Glu–CTC Glu–TTC Gly–ACC Gly–CCC Gly–GCC Gly–TCC His–ATG His–GTG Ile–AAT Ile–GAT Ile–TAT Leu–AAG Leu–CAA Leu–CAG Leu–GAG Leu–TAA Leu–TAG Lys–CTT Lys–TTT Met–CAT Phe–AAA Phe–GAA Pro–AGG Pro–CGG Pro–GGG Pro–TGG Sel–TCA Ser–ACT Ser–AGA Ser–CGA Ser–GCT Ser–GGA Ser–TGA Sup–CTA Sup–TTA Thr–AGT Thr–CGT Thr–GGT Thr–TGT Trp–CCA Tyr–ATA Tyr–GTA Val–AAC Val–CAC Val–GAC Val–TAC

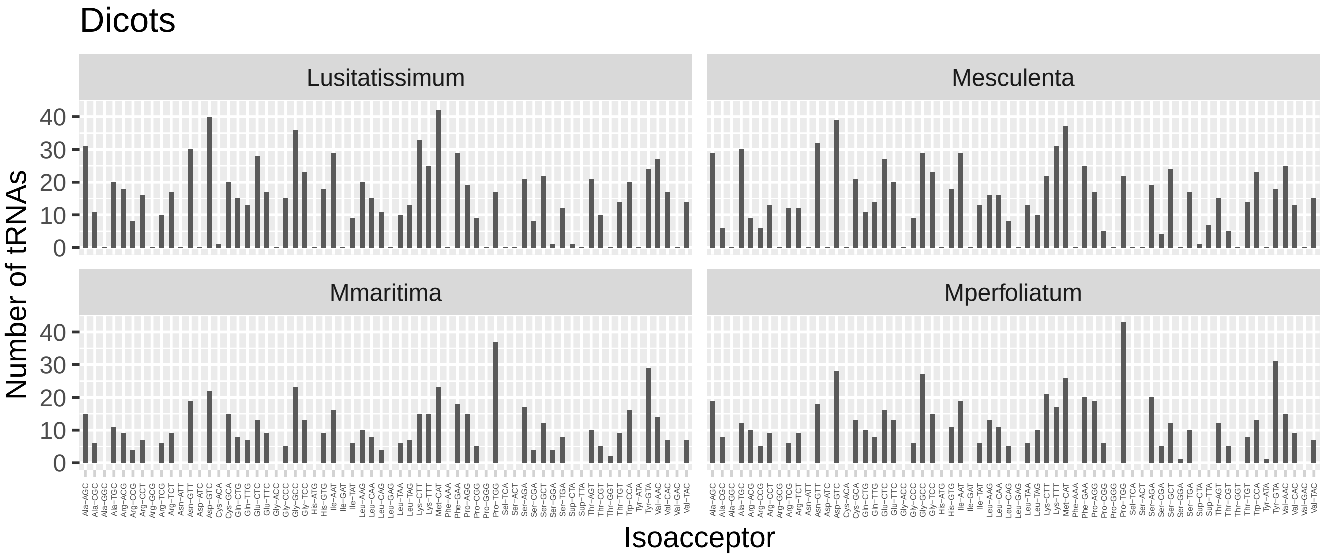
Dicots
Lusitatissimum
Mesculenta
Mmaritima
Mperfoliatum
Number of tRNAs
0
10
20
30
40
Isoacceptor
Ala–AGC Ala–CGC Ala–GGC Ala–TGC Arg–ACG Arg–CCG Arg–CCT Arg–GCG Arg–TCG Arg–TCT Asn–ATT Asn–GTT Asp–ATC Asp–GTC Cys–ACA Cys–GCA Gln–CTG Gln–TTG Glu–CTC Glu–TTC Gly–ACC Gly–CCC Gly–GCC Gly–TCC His–ATG His–GTG Ile–AAT Ile–GAT Ile–TAT Leu–AAG Leu–CAA Leu–CAG Leu–GAG Leu–TAA Leu–TAG Lys–CTT Lys–TTT Met–CAT Phe–AAA Phe–GAA Pro–AGG Pro–CGG Pro–GGG Pro–TGG Sel–TCA Ser–ACT Ser–AGA Ser–CGA Ser–GCT Ser–GGA Ser–TGA Sup–CTA Sup–TTA Thr–AGT Thr–CGT Thr–GGT Thr–TGT Trp–CCA Tyr–ATA Tyr–GTA Val–AAC Val–CAC Val–GAC Val–TAC

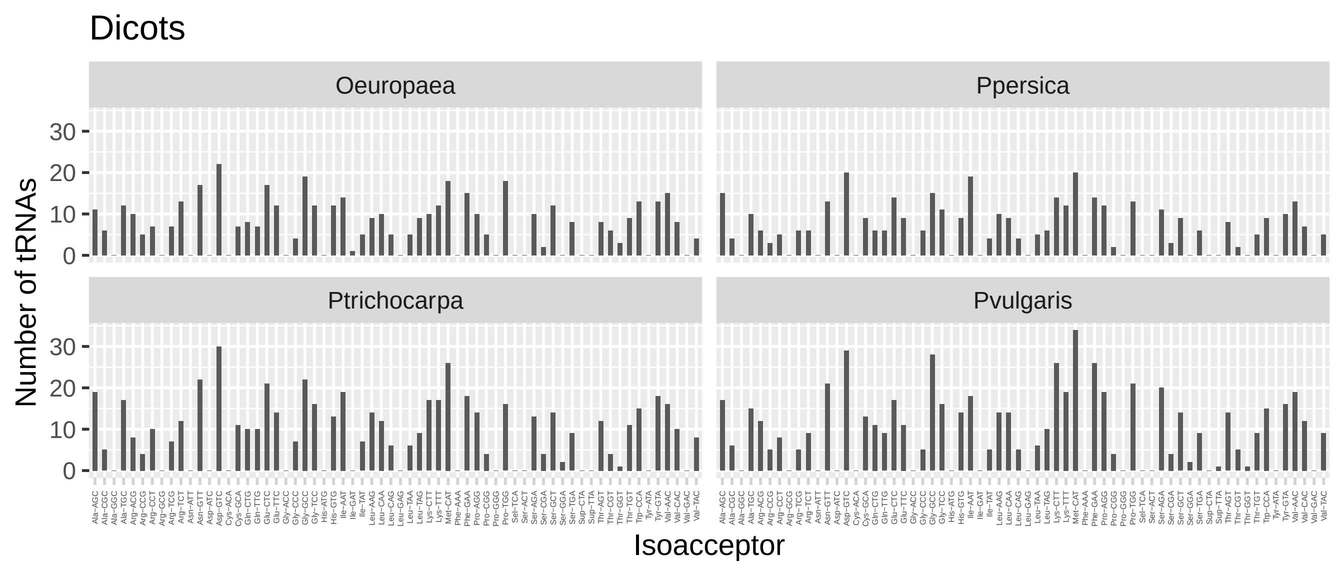

Dicots
Oeuropaea
Ppersica
Ptrichocarpa
Pvulgaris
Number of tRNAs
Isoacceptor
0
10
20
30

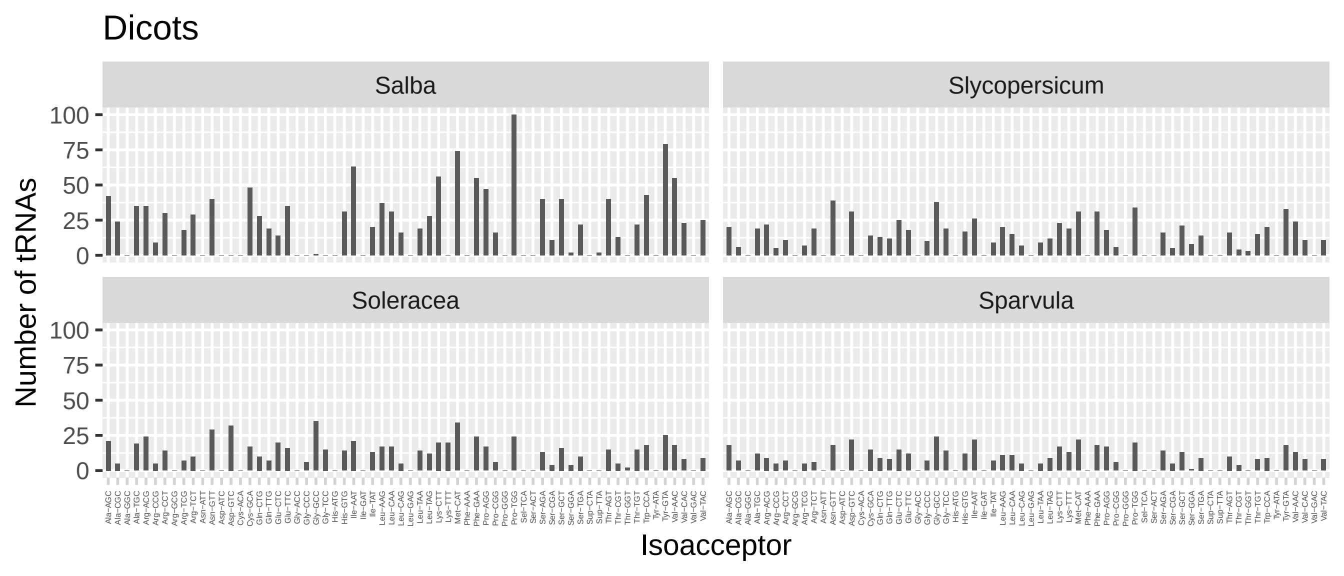

Dicots
Salba
Slycopersicum
Soleracea
Sparvula
Number of tRNAs
Isoacceptor
0
25
50
75
100

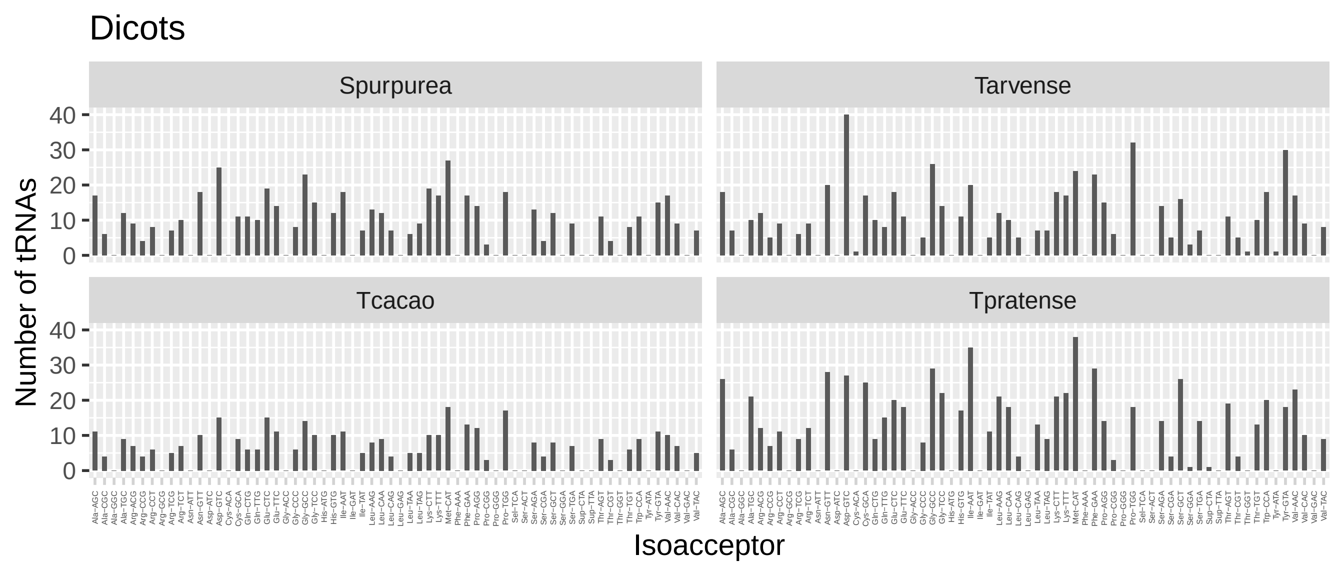
Dicots
Spurpurea
Tarvense
Tcacao
Tpratense
Number of tRNAs
0
10
20
30
40
Isoacceptor
Ala-AGC Ala-CGC Ala-GGC Ala-TGC Arg-ACG Arg-CCG Arg-CCT Arg-GCG Arg-TCG Arg-TCT Asn-ATT Asn-GTT Asp-ATC Asp-GTC Cys-ACA Cys-GCA Gln-CTG Gln-TTG Glu-CTC Glu-TTC Gly-ACC Gly-CCC Gly-GCC Gly-TCC His-ATG His-GTG Ile-AAT Ile-GAT Ile-TAT Leu-AAG Leu-CAA Leu-CAG Leu-GAG Leu-TAA Leu-TAG Lys-CTT Lys-TTT Met-CAT Phe-AAA Phe-GAA Pro-AGG Pro-CGG Pro-TGG Sel-TCA Ser-ACT Ser-AGA Ser-CGA Ser-GCT Ser-GGA Ser-TGA Sup-CTA Sup-TTA Thr-AGT Thr-CGT Thr-GGT Thr-TGT Trp-CCA Tyr-ATA Tyr-GTA Val-AAC Val-CAC Val-GAC Val-TAC

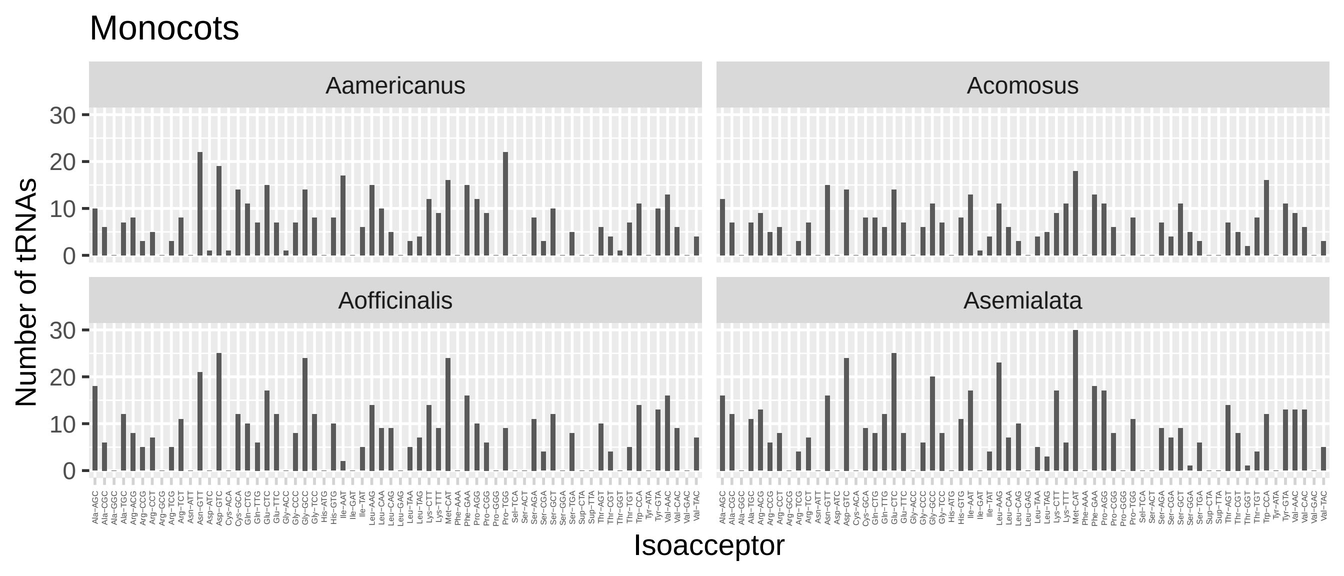

Monocots
Aamericanus
Acomosus
Aofficinalis
Asemialata
Number of tRNAs
Isoacceptor
0
10
20
30

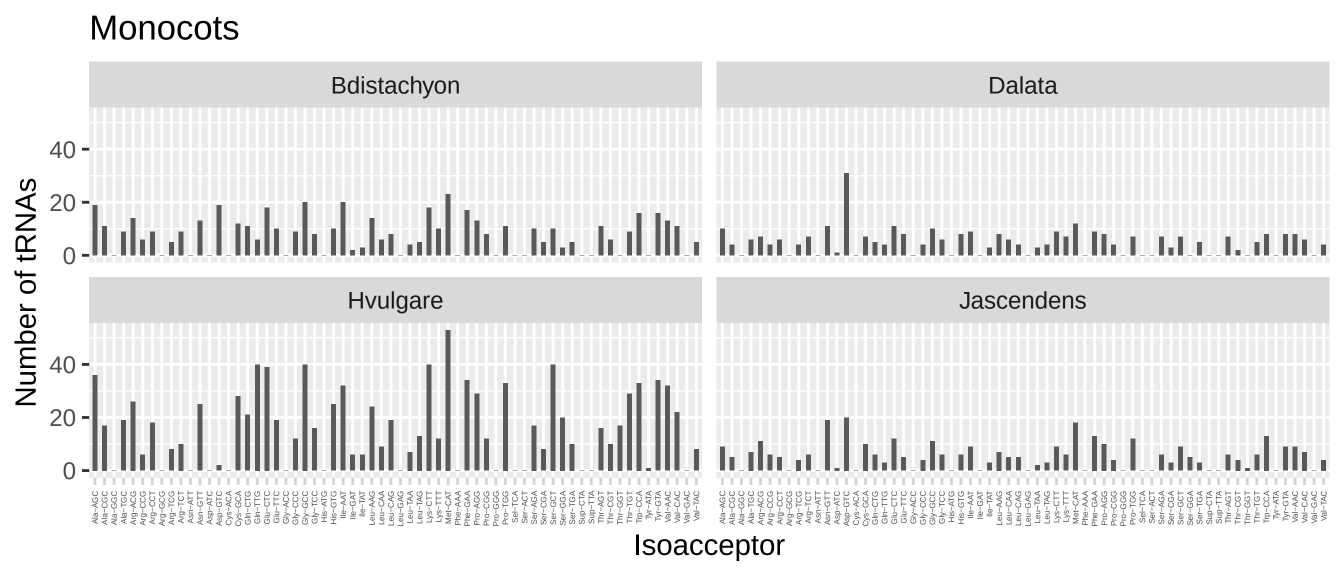
Monocots
Bdistachyon
Dalata
Hvulgare
Jascendens
Number of tRNAs
0
20
40
Isoacceptor
Ala-AGC Ala-CGC Ala-GGC Ala-TGC Arg-ACG Arg-CCG Arg-CCT Arg-GCG Arg-TCG Arg-TCT Asn-ATT Asn-GTT Asp-ATC Asp-GTC Cys-ACA Cys-GCA Gln-CTG Gln-TTG Glu-CTC Glu-TTC Gly-ACC Gly-CCC Gly-GCC Gly-TCC His-ATG His-GTG Ile-AAT Ile-GAT Ile-TAT Leu-AAG Leu-CAA Leu-CAG Leu-GAG Leu-TAA Leu-TAG Lys-CTT Lys-TTT Met-CAT Phe-AAA Phe-GAA Pro-AGG Pro-CGG Pro-GGG Pro-TGG Sel-TCA Ser-ACT Ser-AGA Ser-CGA Ser-GCT Ser-GGA Ser-TGA Sup-CTA Sup-TTA Thr-AGT Thr-CGT Thr-GGT Thr-TGT Trp-CCA Tyr-ATA Tyr-GTA Val-AAC Val-CAC Val-GAC Val-TAC

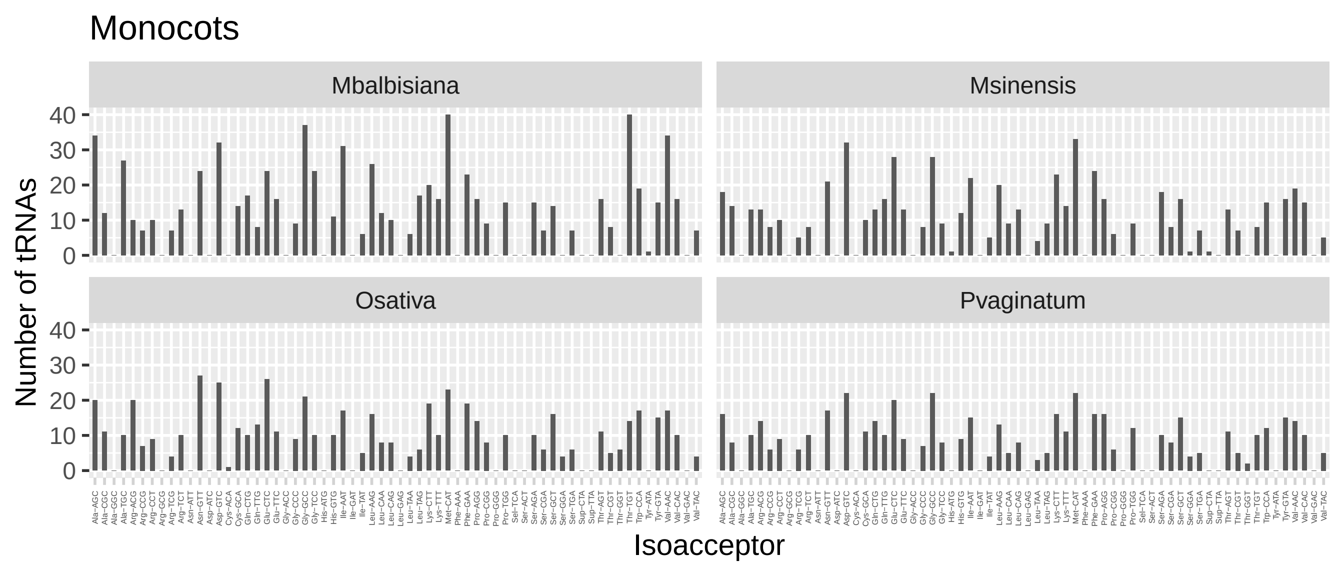

Monocots
Mbalbisiana
Msinensis
Osativa
Pvaginatum
Number of tRNAs
Isoacceptor

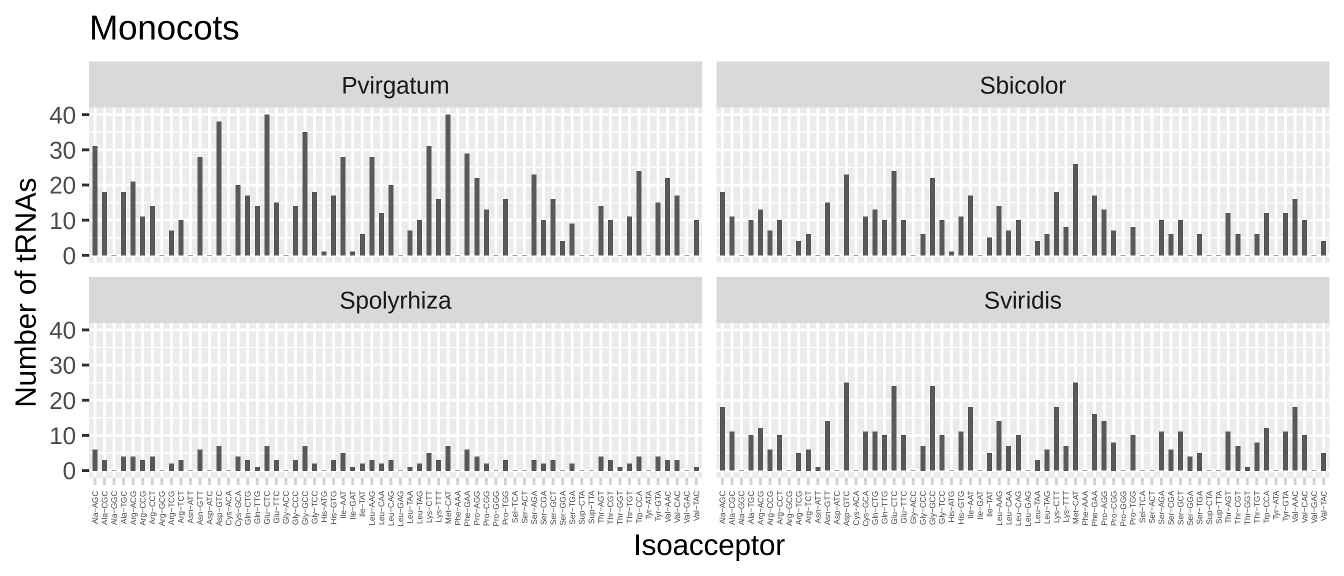
Monocots
Pvirgatum
Sbicolor
Spolyrhiza
Sviridis
Number of tRNAs
Isoacceptor
0
10
20
30
40

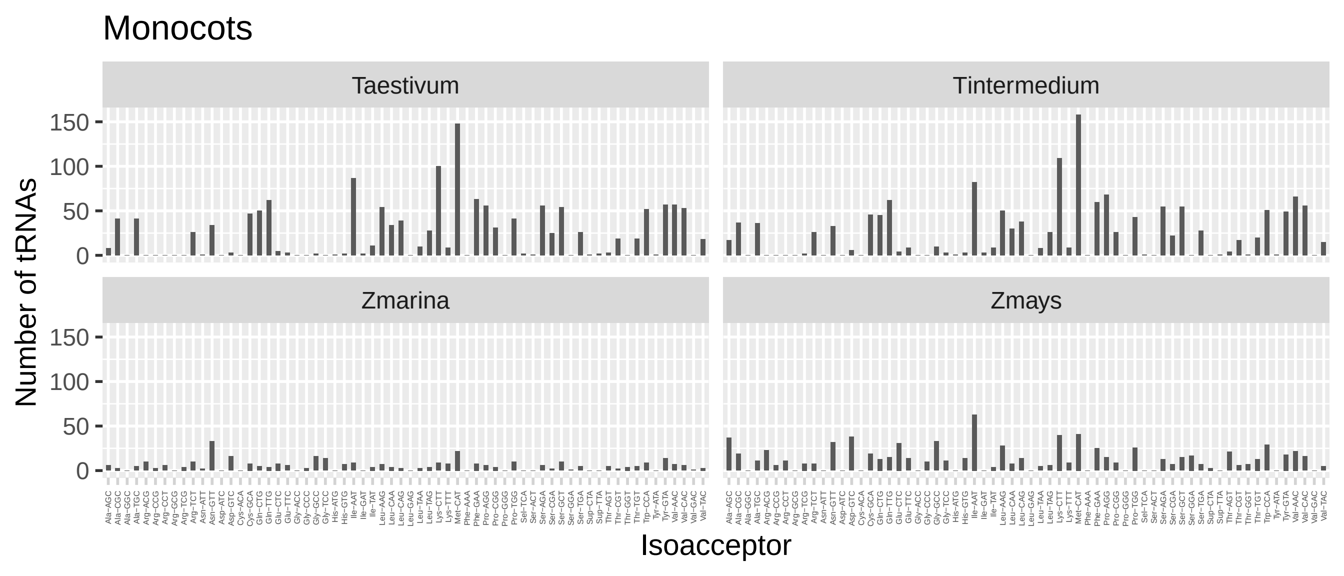
Monocots
Taestivum
Tintermedium
Zmarina
Zmays
Number of tRNAs
150
100
50
0
Isoacceptor
Ala–AGC Ala–CGC Ala–GGC Ala–TGC Arg–ACG Arg–CCG Arg–CCT Arg–GCG Arg–TCG Arg–TCT Asn–ATT Asn–GTT Asp–ATC Asp–GTC Cys–ACA Cys–GCA Gln–CTG Gln–TTG Glu–CTC Glu–TTC Gly–ACC Gly–CCC Gly–GCC Gly–TCC His–ATG His–GTG Ile–AAT Ile–GAT Ile–TAT Leu–AAG Leu–CAA Leu–CAG Leu–GAG Leu–TAA Leu–TAG Lys–CTT Lys–TTT Met–CAT Phe–AAA Phe–GAA Pro–AGG Pro–CGG Pro–GGG Pro–TGG Sel–TCA Ser–ACT Ser–AGA Ser–CGA Ser–GCT Ser–GGA Ser–TGA Sup–CTA Sup–TTA Thr–AGT Thr–CGT Thr–GGT Thr–TGT Trp–CCA Tyr–ATA Tyr–GTA Val–AAC Val–CAC Val–GAC Val–TAC
