# Supplementary material for: tRNA gene content, structure, and organization in the flowering plant lineage
Source: Front Plant Sci. 2024 Dec 23;15:1486612. doi: 10.3389/fpls.2024.1486612 (PMC11700998; doi:10.3389/fpls.2024.1486612)
Supplement: Supplementary file 2 [file DataSheet2.pdf]

Supplementary File 2. Logo plots showing the consensus A box sequences.

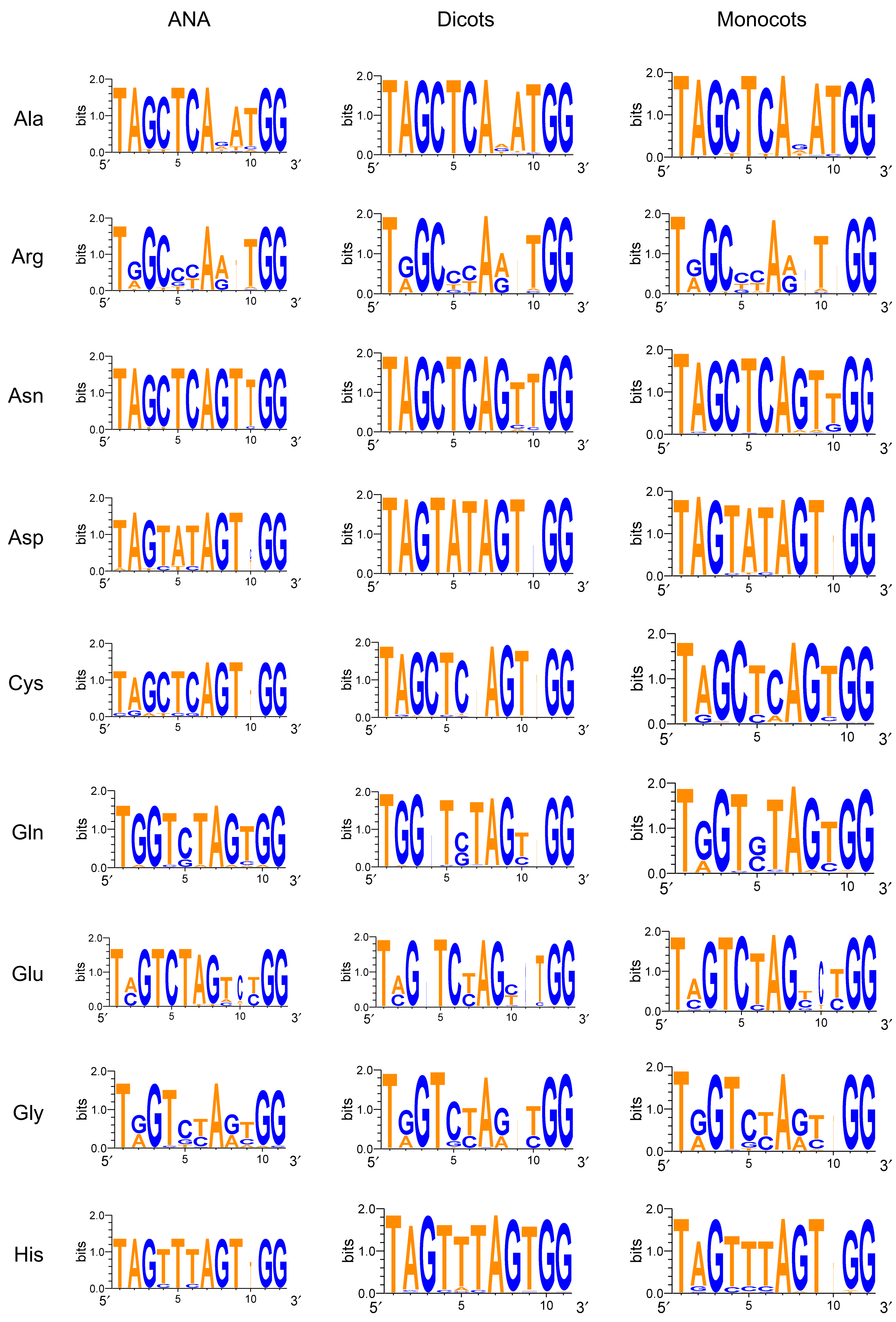
ANA
Dicots
Monocots
Ala
Arg
Asn
Asp
Cys
Gln
Glu
Gly
His
bits
2.0
1.0
0.0
5′
5
10
3′

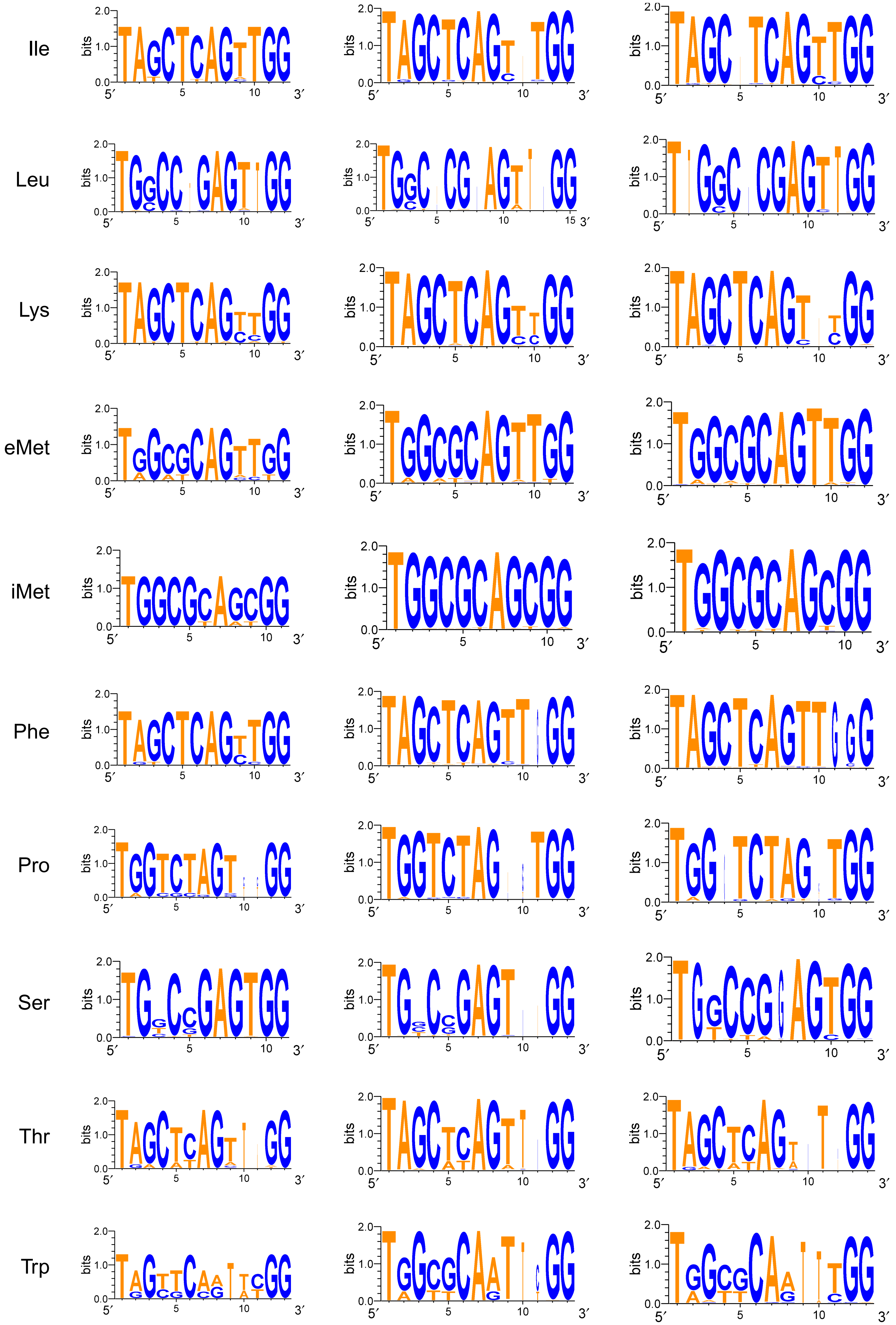
Ile
Leu
Lys
eMet
iMet
Phe
Pro
Ser
Thr
Trp
bits
5′
3′

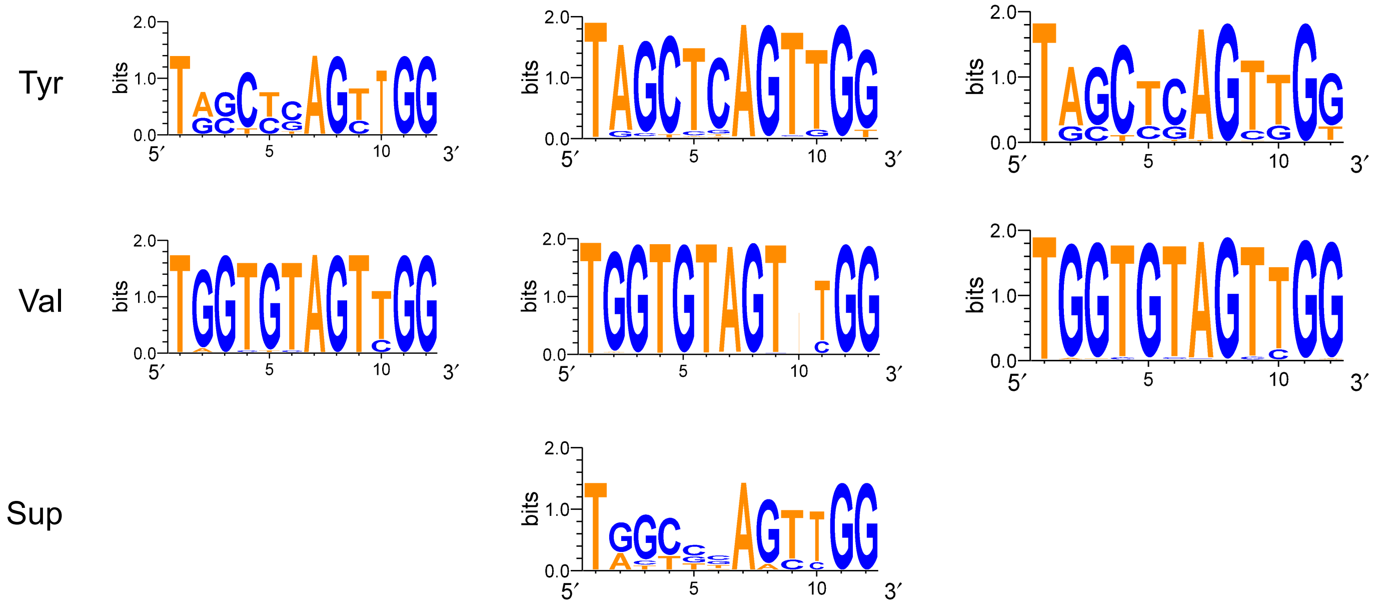
Tyr
Val
Sup
bits
2.0
1.0
0.0
5′
5
10
3′
