# Supplementary material for: tRNA gene content, structure, and organization in the flowering plant lineage
Source: Front Plant Sci. 2024 Dec 23;15:1486612. doi: 10.3389/fpls.2024.1486612 (PMC11700998; doi:10.3389/fpls.2024.1486612)
Supplement: Supplementary file 3 [file DataSheet3.pdf]

Supplementary File 3. Logo plots showing the consensus B box sequences.

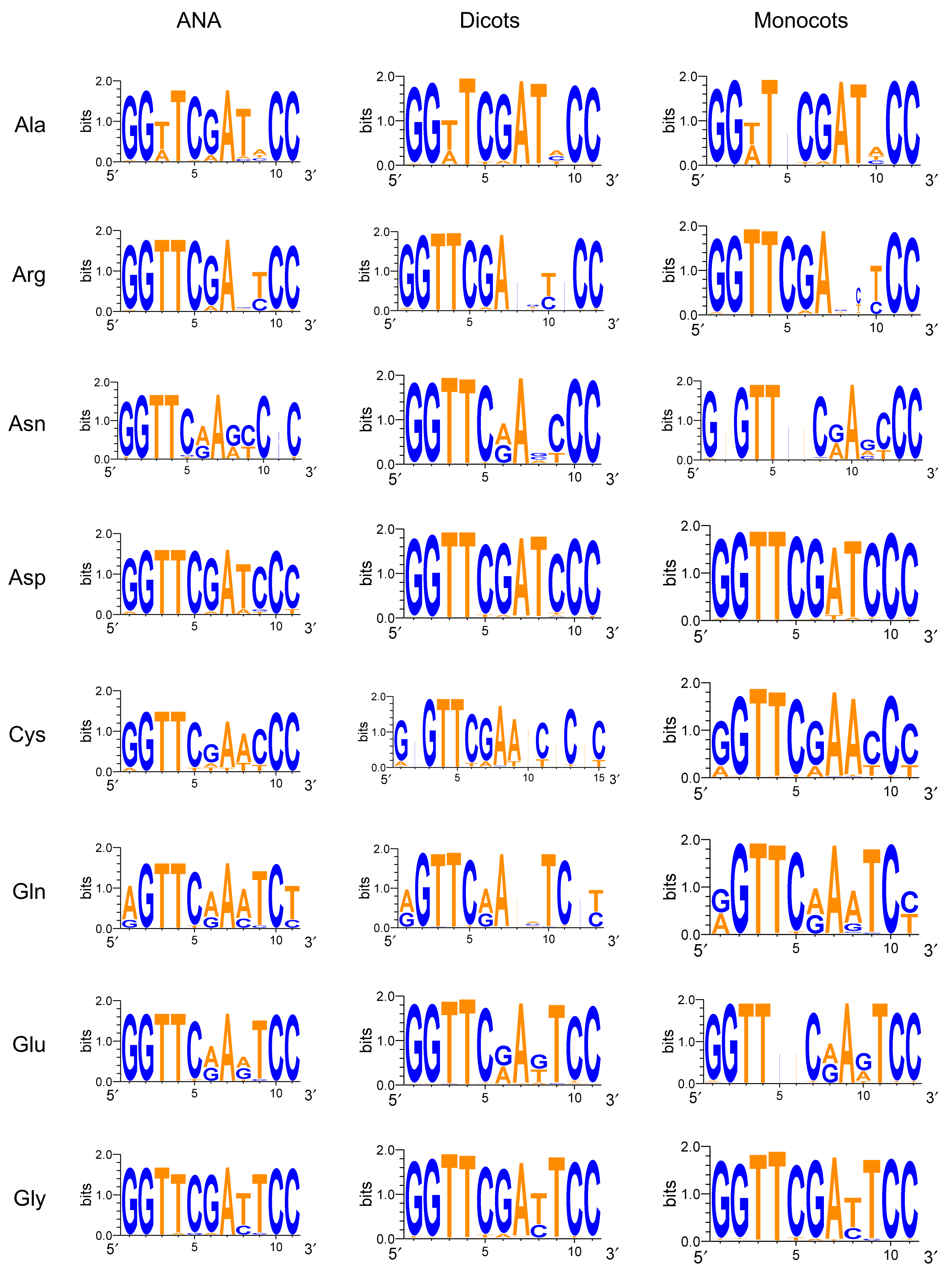
ANA
Dicots
Monocots
Ala
Arg
Asn
Asp
Cys
Gln
Glu
Gly

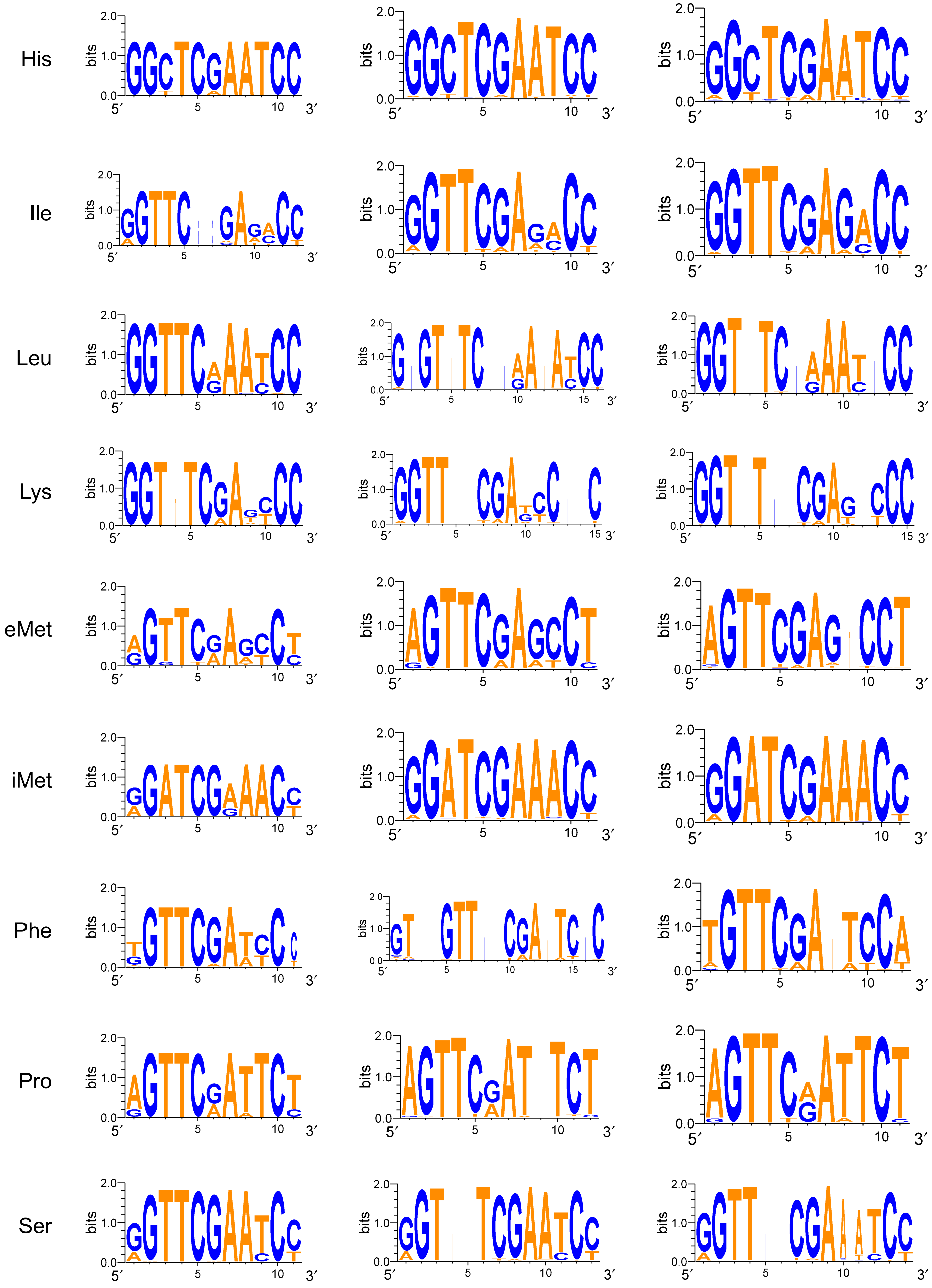
His
Ile
Leu
Lys
eMet
iMet
Phe
Pro
Ser
bits
5′
3′

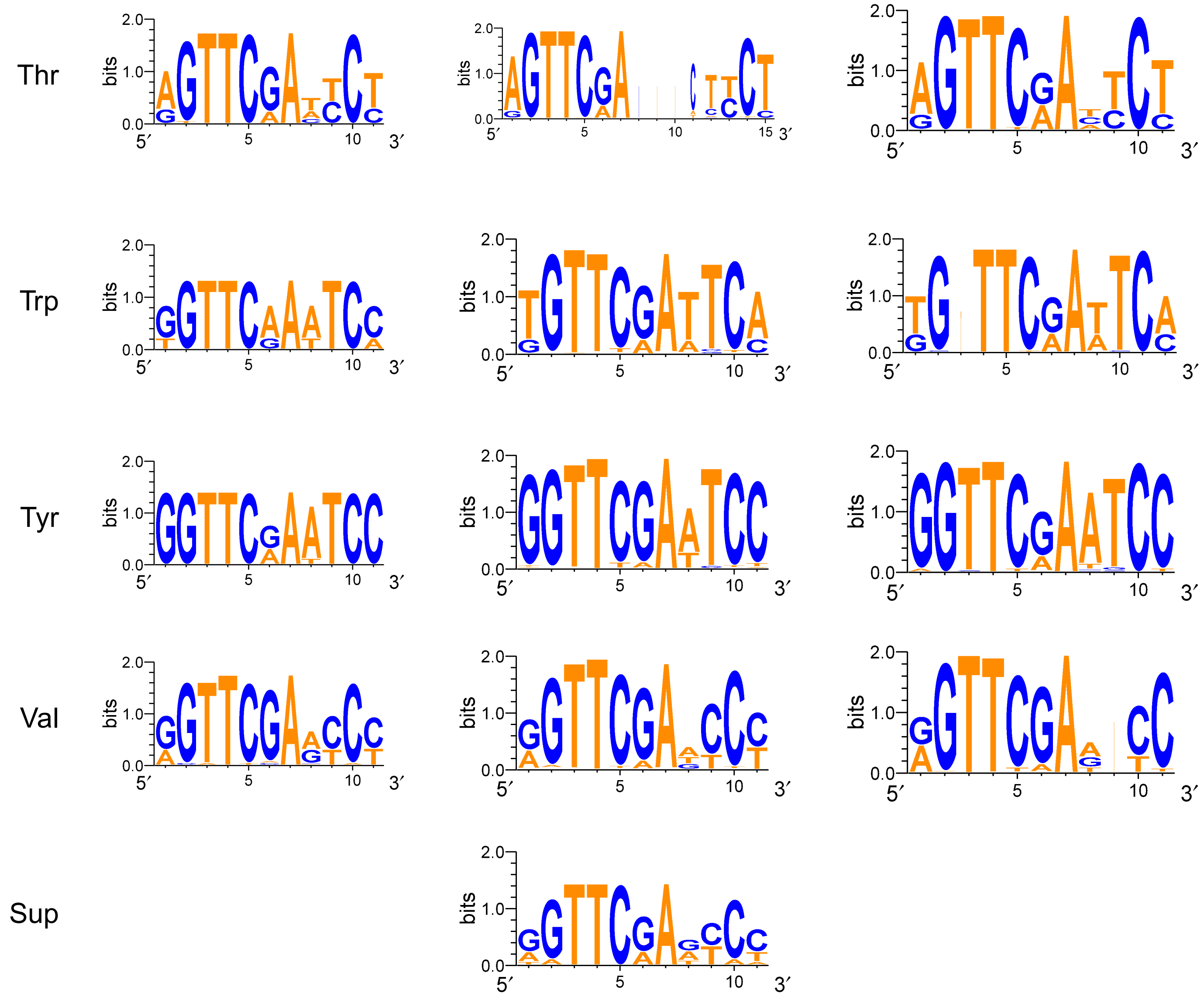
Thr
Trp
Tyr
Val
Sup
bits
5′
3′
