# Supplementary figures and images for: tRNA gene content, structure, and organization in the flowering plant lineage
Source: Front Plant Sci. 2024 Dec 23;15:1486612. doi: 10.3389/fpls.2024.1486612 (PMC11700998; doi:10.3389/fpls.2024.1486612)

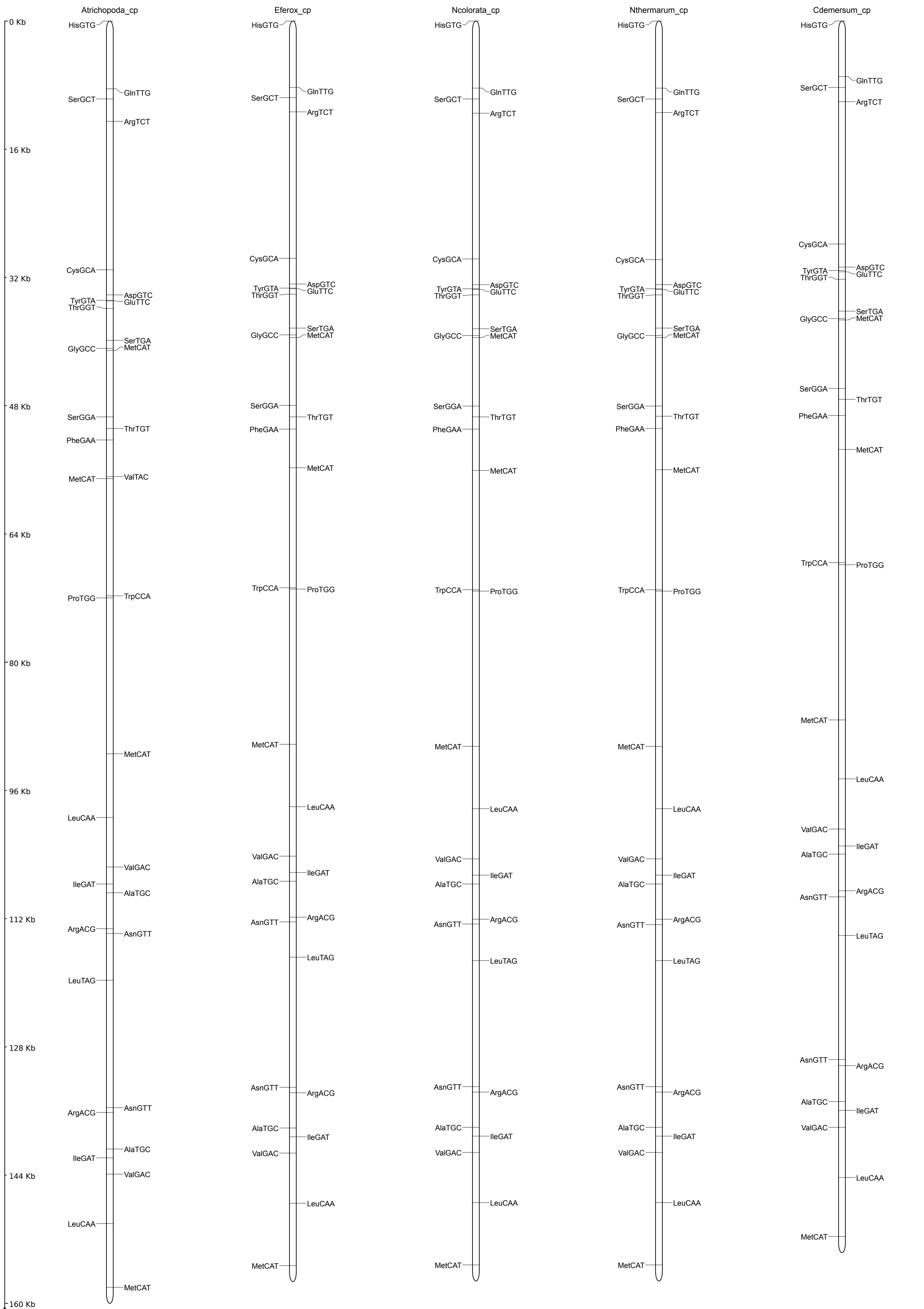

Supplement: Supplementary file 9 [file DataSheet9.pdf]

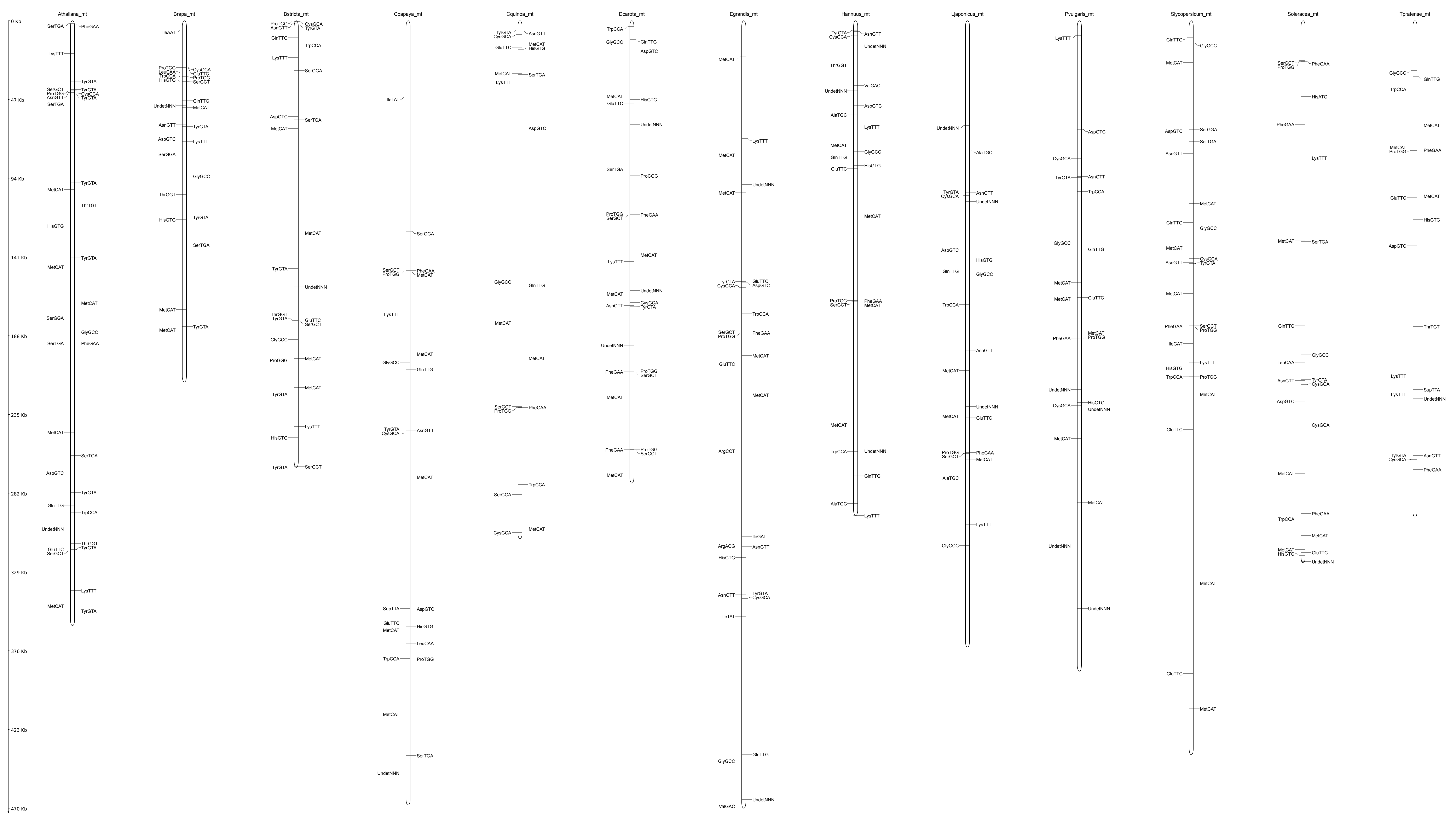

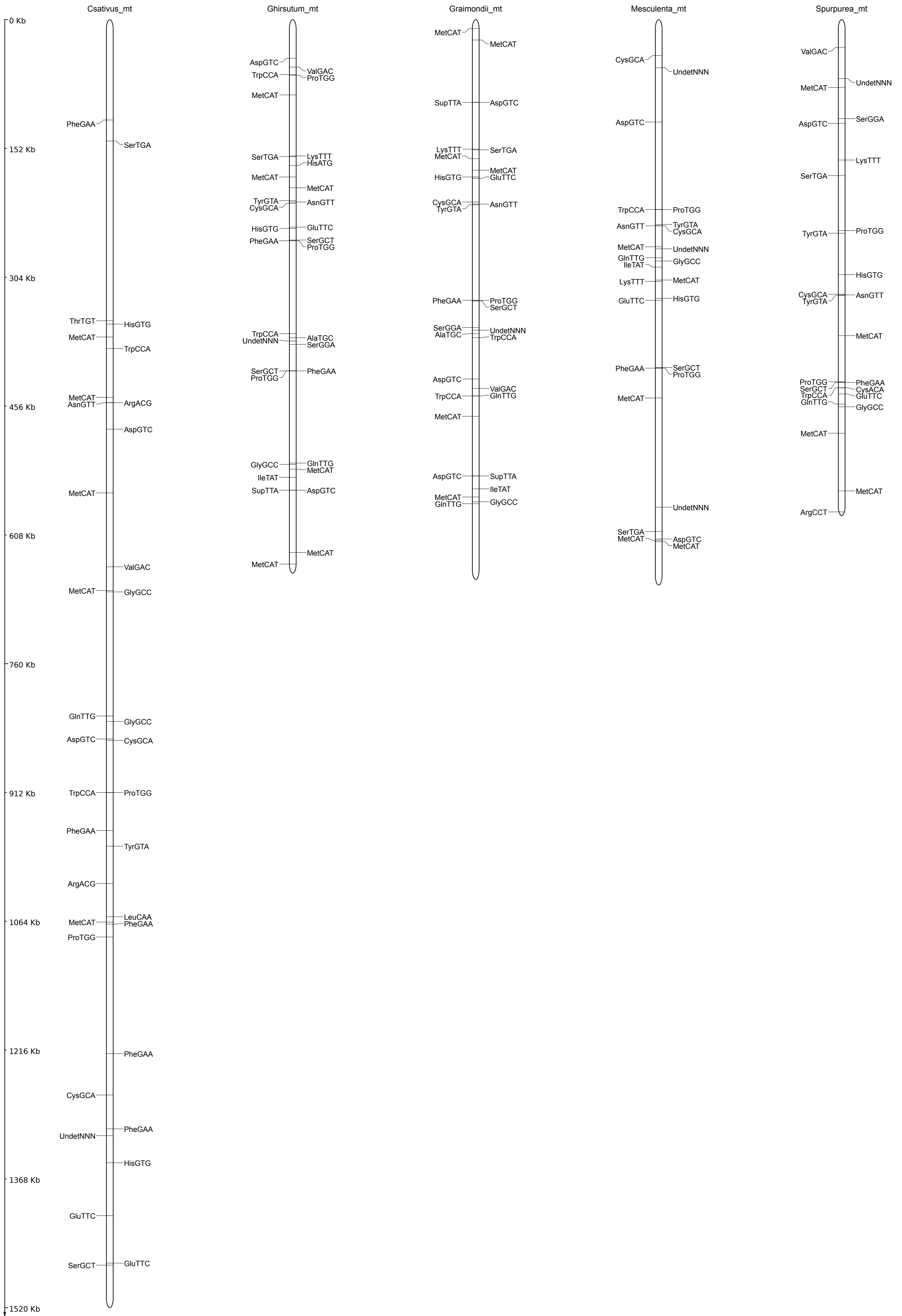

Supplement: Supplementary file 10 [file DataSheet10.pdf]

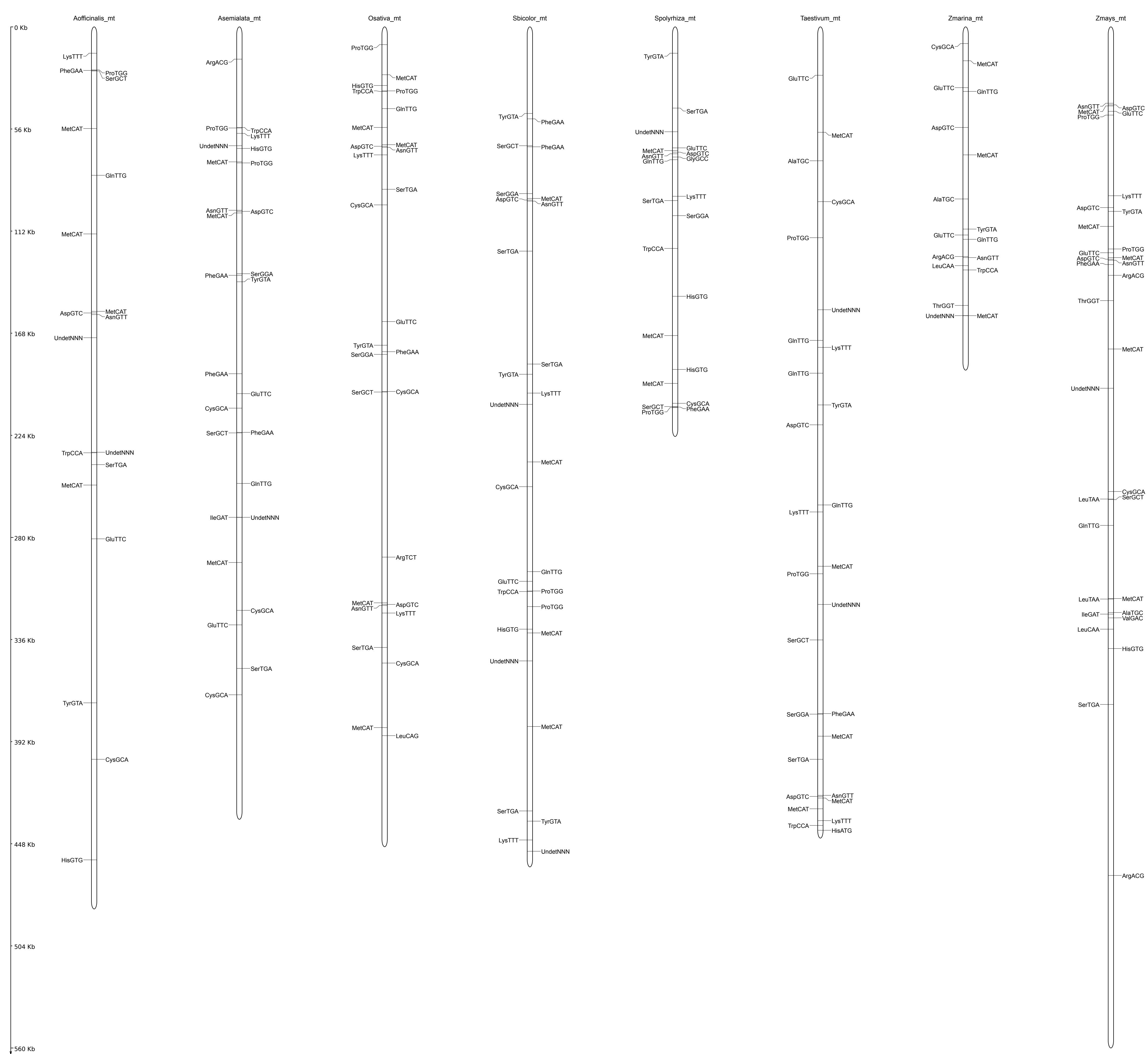

Supplement: Supplementary file 11 [file DataSheet11.pdf]

# Ncolorata\_mt

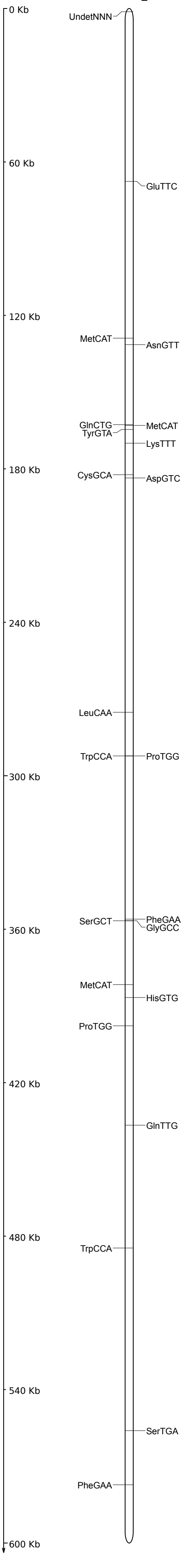

Supplement: Supplementary file 12 [file DataSheet12.pdf]
