# Supplementary material for: tRNA gene content, structure, and organization in the flowering plant lineage
Source: Front Plant Sci. 2024 Dec 23;15:1486612. doi: 10.3389/fpls.2024.1486612 (PMC11700998; doi:10.3389/fpls.2024.1486612)
Supplement: Supplementary file 13 [file Presentation1.pdf]

Supplementary Material

1 Supplementary Figures and Tables

1.1 Supplementary Figures

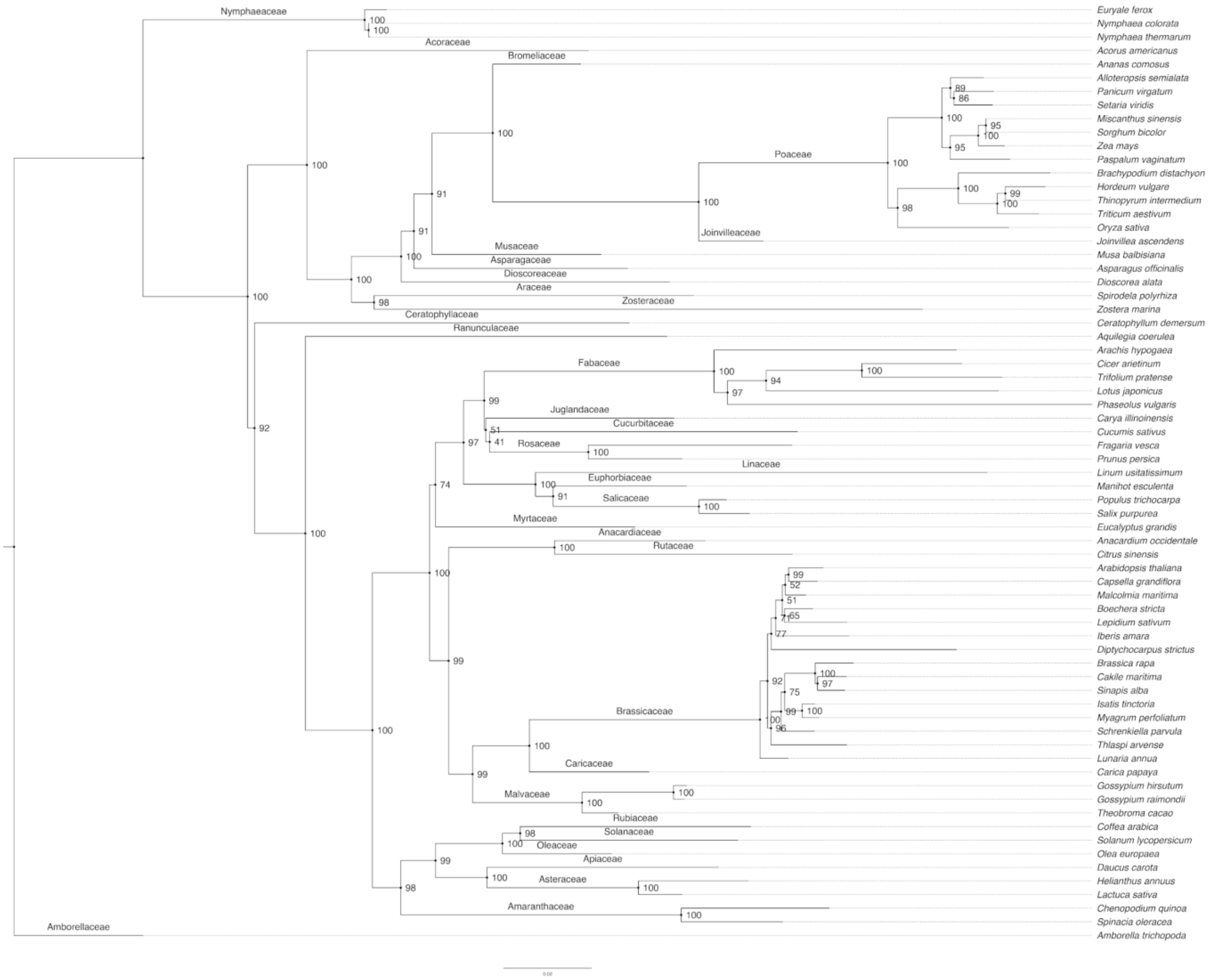

Supplementary Figure 1. A phylogenetic tree of 69 flowering plants used for determining tRNA gene numbers. This gene tree is based on each plant genome's concatenated *matK* and *rbcL* genes. Numbers beside the nodes (dots) indicate bootstrap values.

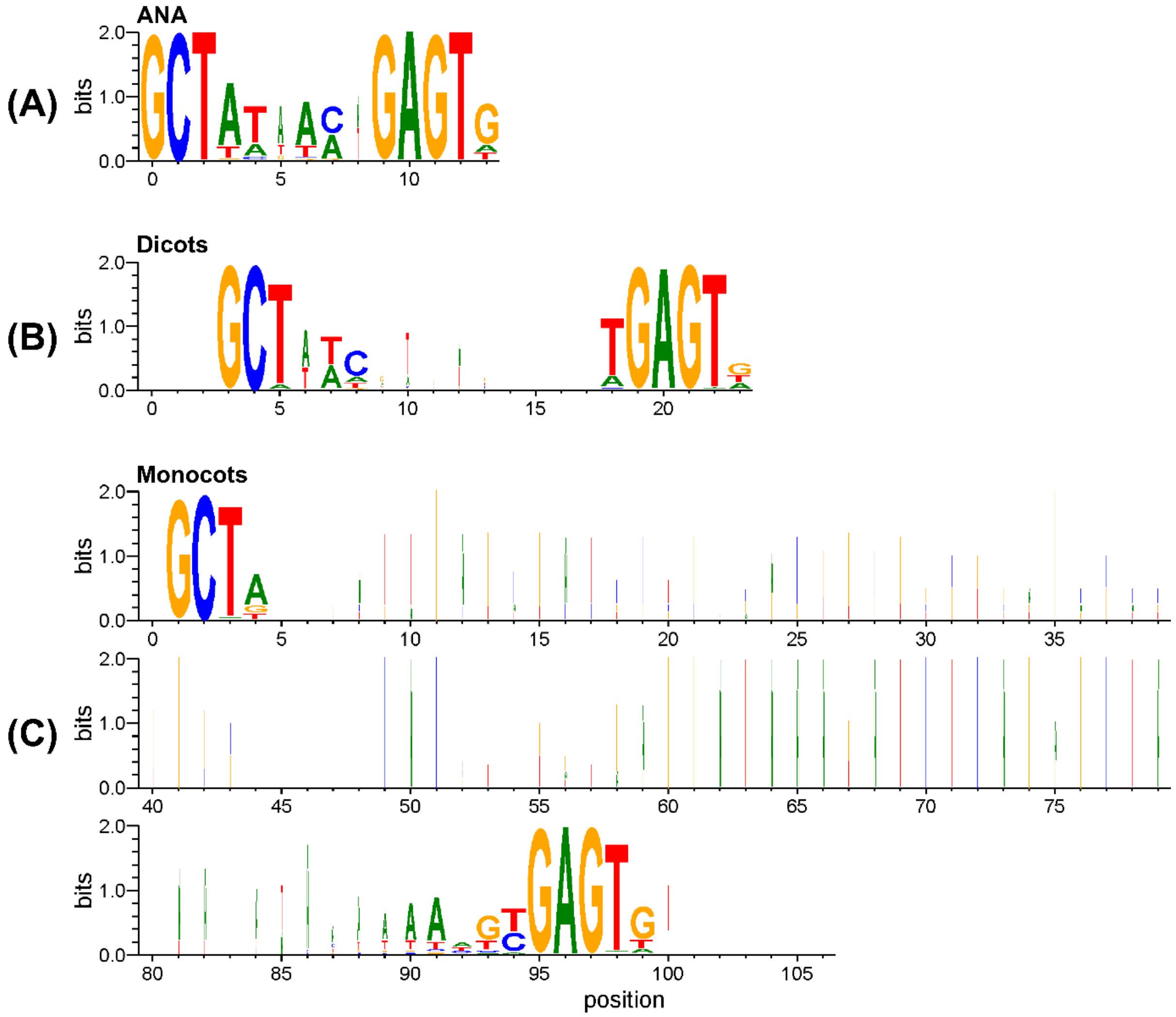

Supplementary Figure 2. tRNA^{eMet} intron conservation. Logo plots representing conserved tRNA^{Met} intronic sequences are shown for (A) ANA, (B) eudicots, and (C) monocots. Columns with empty or narrow stacks represent positions with more gaps than nucleotides.

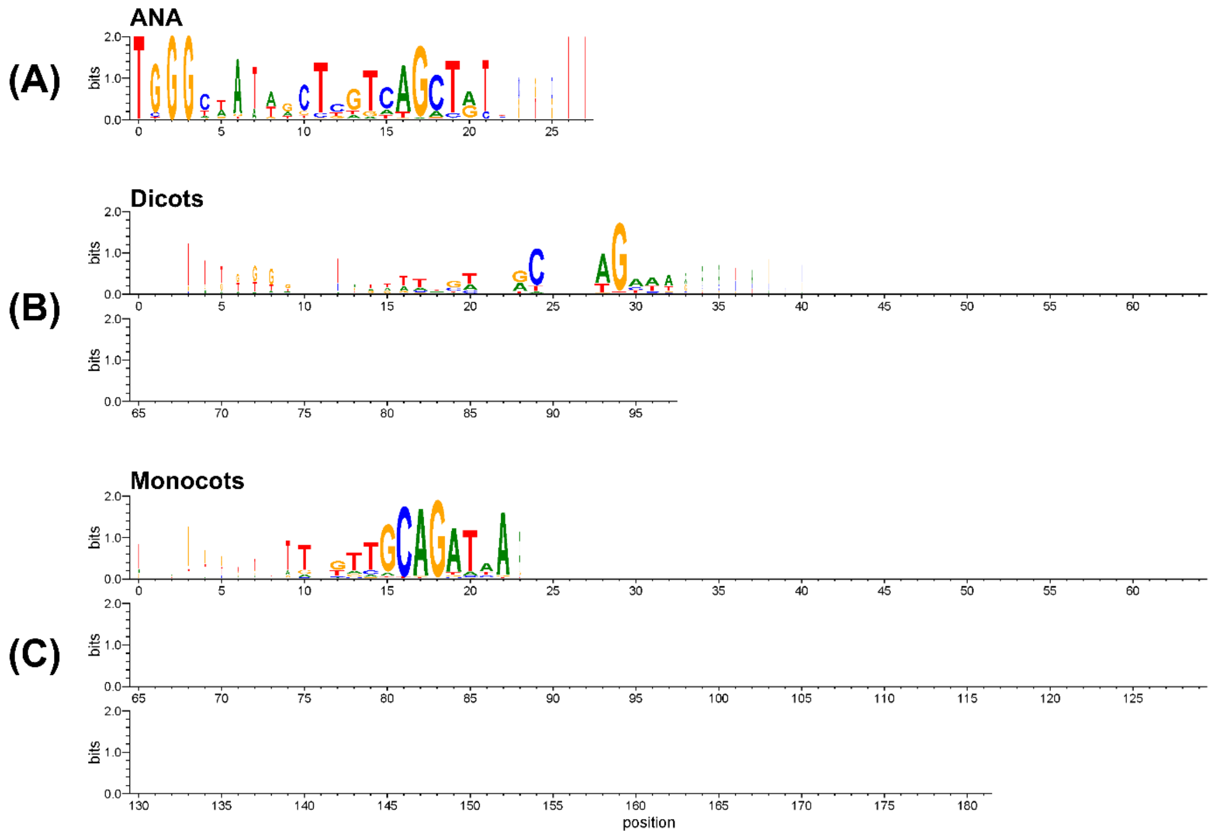

Supplementary Figure 3. tRNA^{Tyr} intron conservation. Logo plots representing conserved tRNA^{Tyr} intronic sequences are shown for (A) ANA, (B) eudicots, and (C) monocots. Columns with empty or narrow stacks represent positions with more gaps than nucleotides.

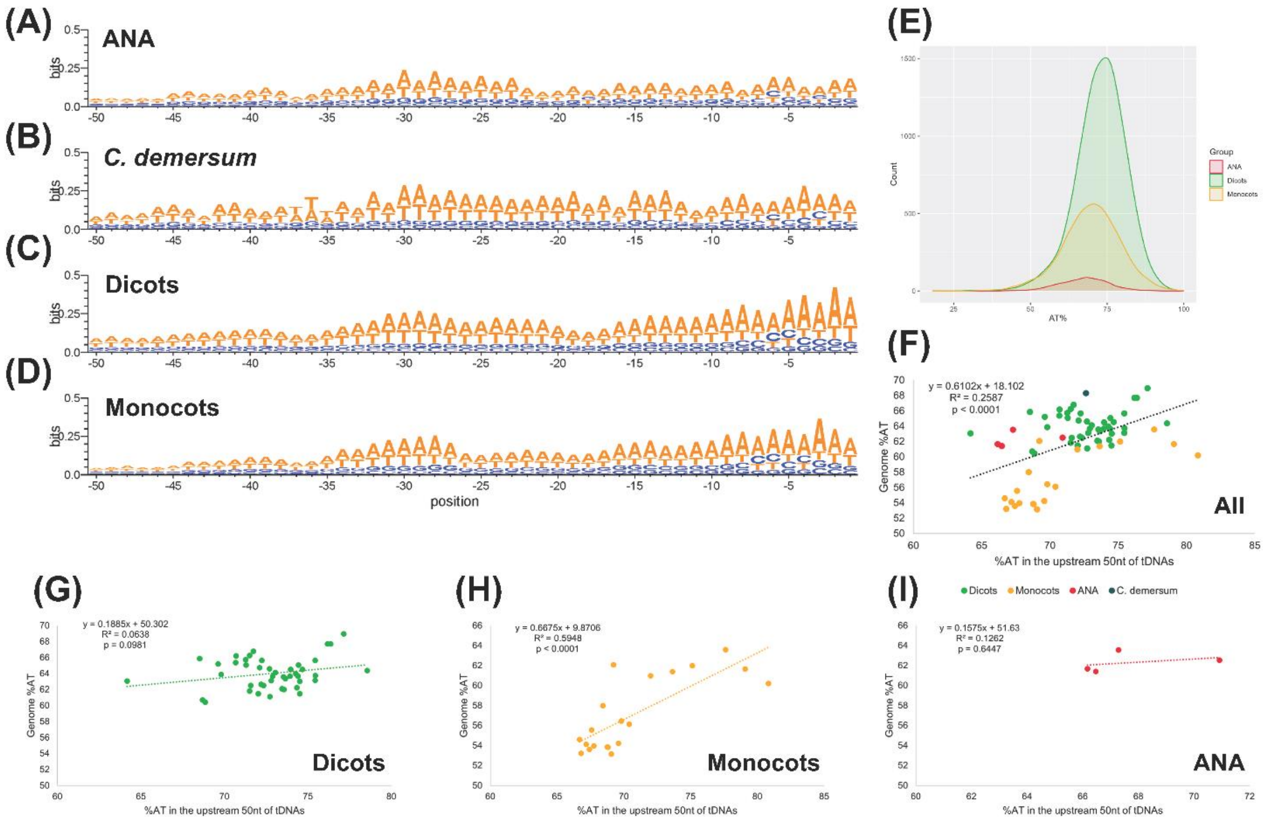

Supplementary Figure 4. Nature of the region 50 nucleotides upstream of nuclear tDNAs. (A-D) Logo plots corresponding to the first 50 nucleotides upstream of tDNAs. (E) The distribution of A/T content (in percentage) among sequences 50 nucleotides upstream of tDNAs (green, eudicots; yellow, monocots; red, ANA). Correlation between the A/T content (in percentage) of tDNA

upstream sequences and that of the whole genome is also shown for all (F) and each lineage: (G) eudicots, (H) monocots, and (I) ANA.

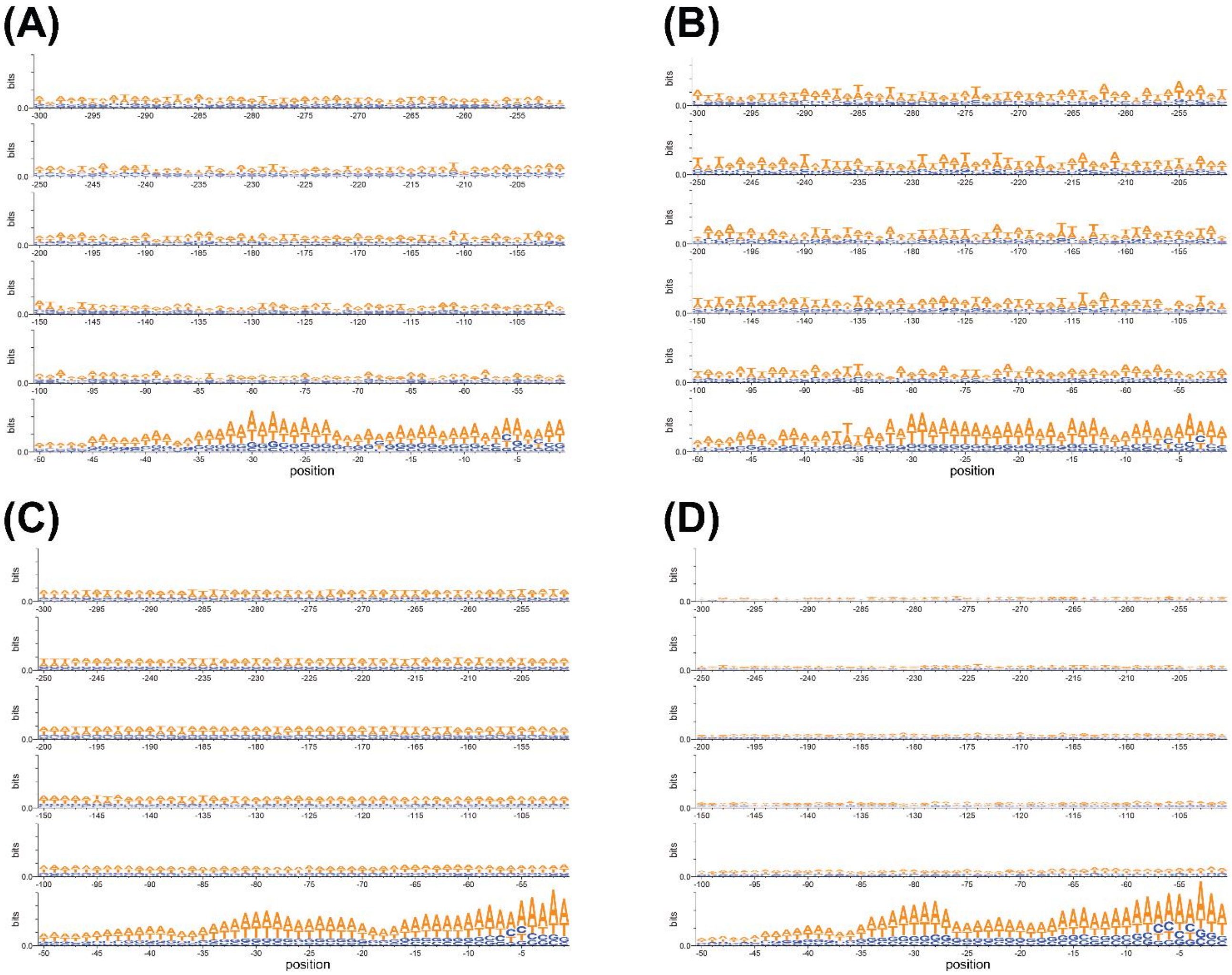

Supplementary Figure 5. Logo plots corresponding to the first 300 nucleotides upstream of nuclear tDNAs of (A) ANA, (B) *Ceratophyllum*, (C) eudicot, and (D) monocot genomes.

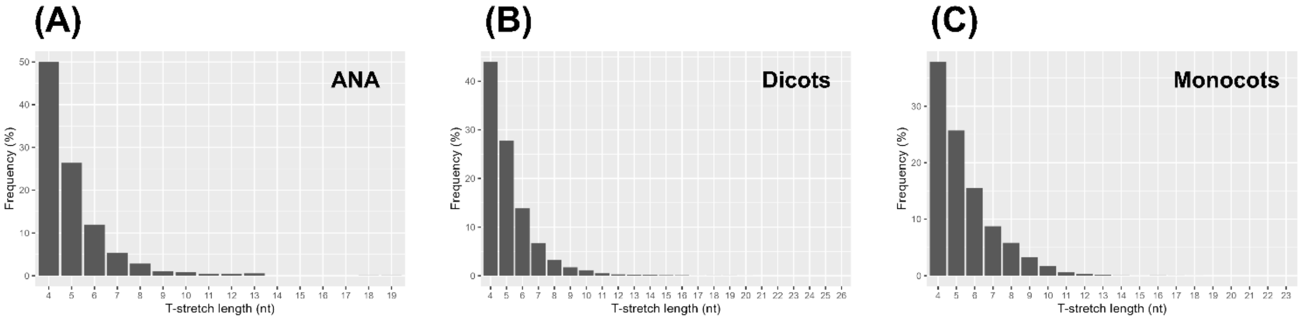

Supplementary Figure 6. Distribution of poly(T) stretch lengths in sequences downstream of tDNAs of (A) ANA, (B) eudicots, and (C) monocot genomes

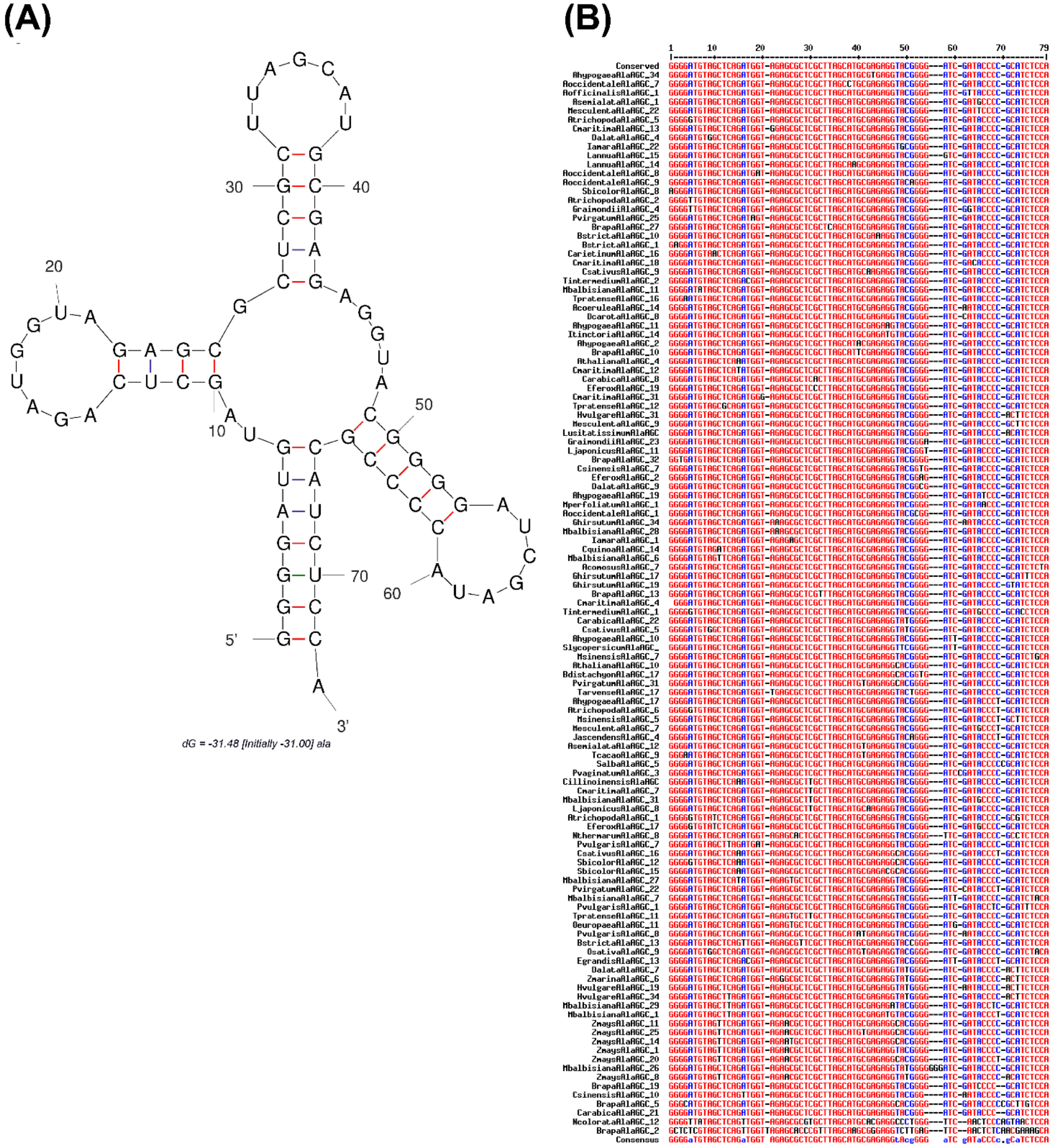

Supplementary Figure 7. Structure of the conserved tRNA$^{Ala-AGC}$ species. (A) The secondary structure of the tRNA$^{Ala-AGC}$ species found to be conserved in all 69 angiosperm genomes. Each unique base pair is colored differently. (B) Multiple sequence alignment showing all detected

polymorphic sequences of $tDNA^{Ala-AGC}$. The first sequence labeled “Conserved” corresponds to the sequence of (A).

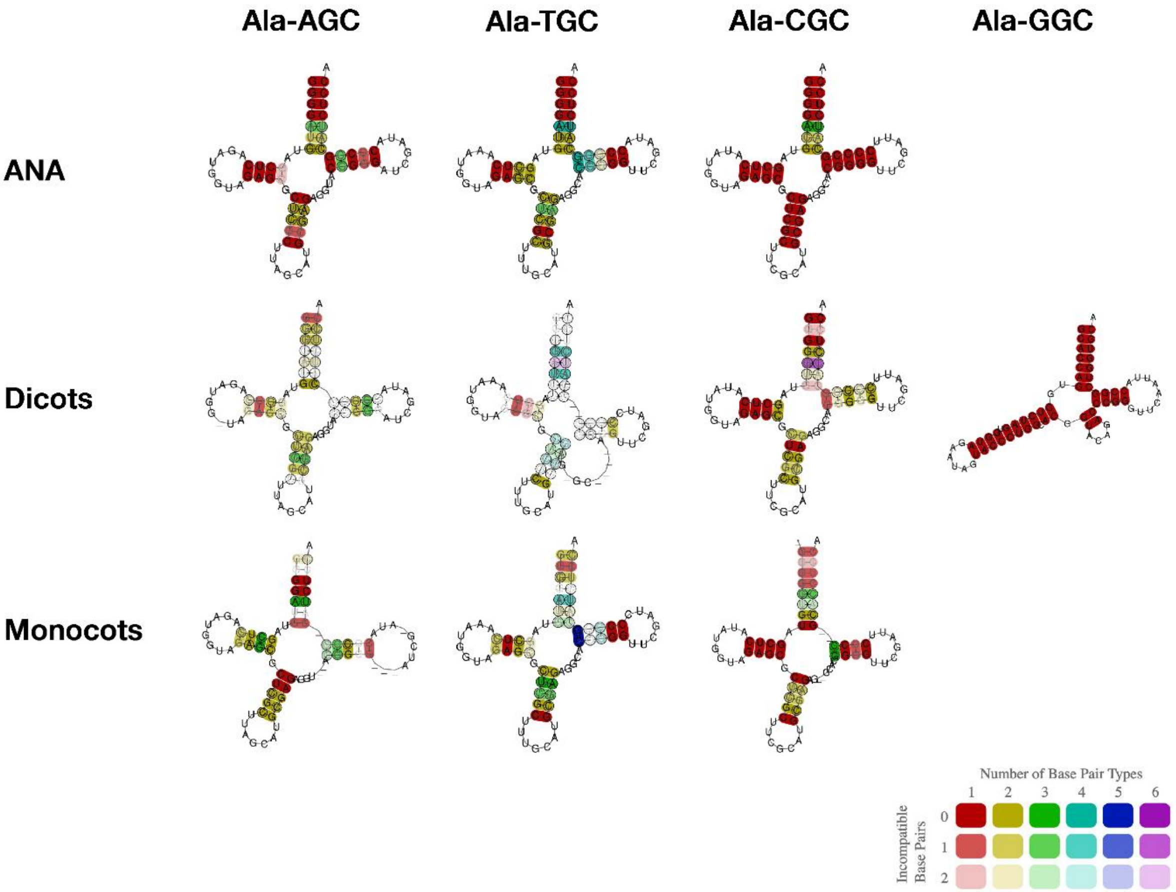

Supplementary Figure 8. Consensus structures of all $tRNA^{Ala}$ genes per isoacceptor as determined by RNAalifold (Bernhart et al., 2008).

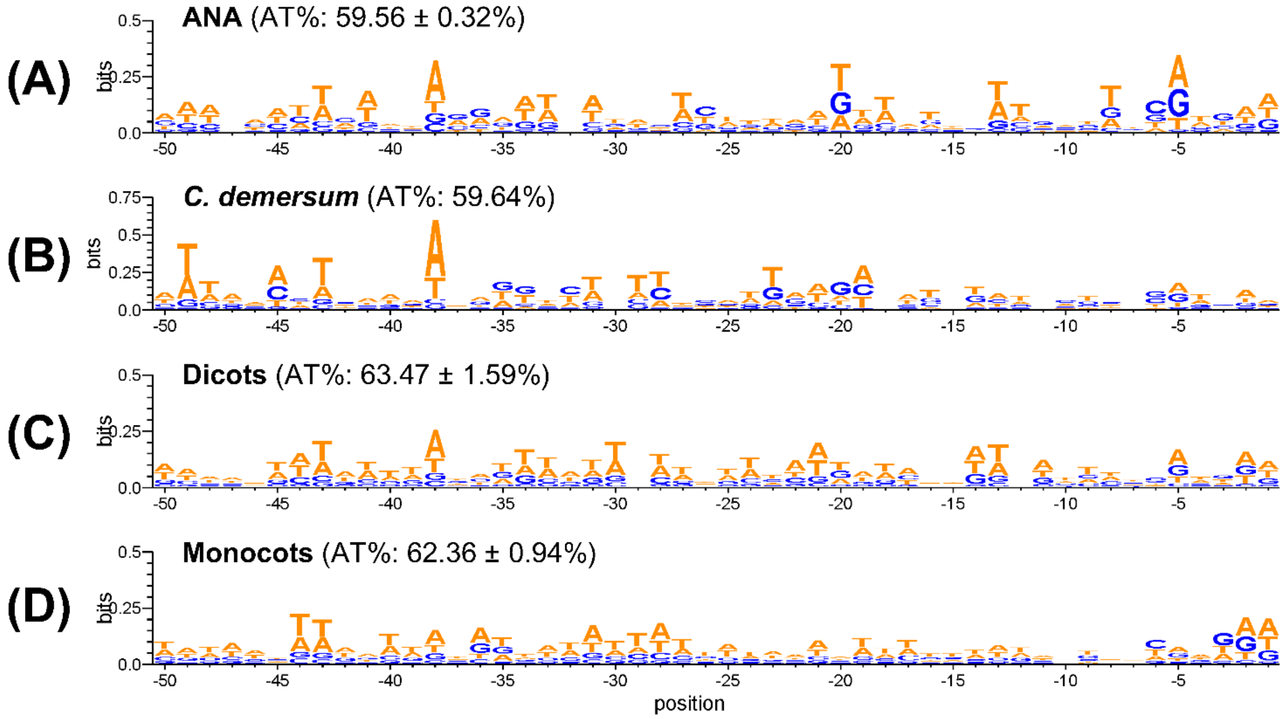

Supplementary Figure 9. Nature of the region 50 nucleotides upstream of chloroplast tDNAs. Logo plots corresponding to the first 50 nucleotides upstream of chloroplast tDNAs and their A/T content

(in percentage) are shown for the (A) ANA, (B) *Ceratophyllum demersum*, (C) eudicot, and (D) monocot genomes.

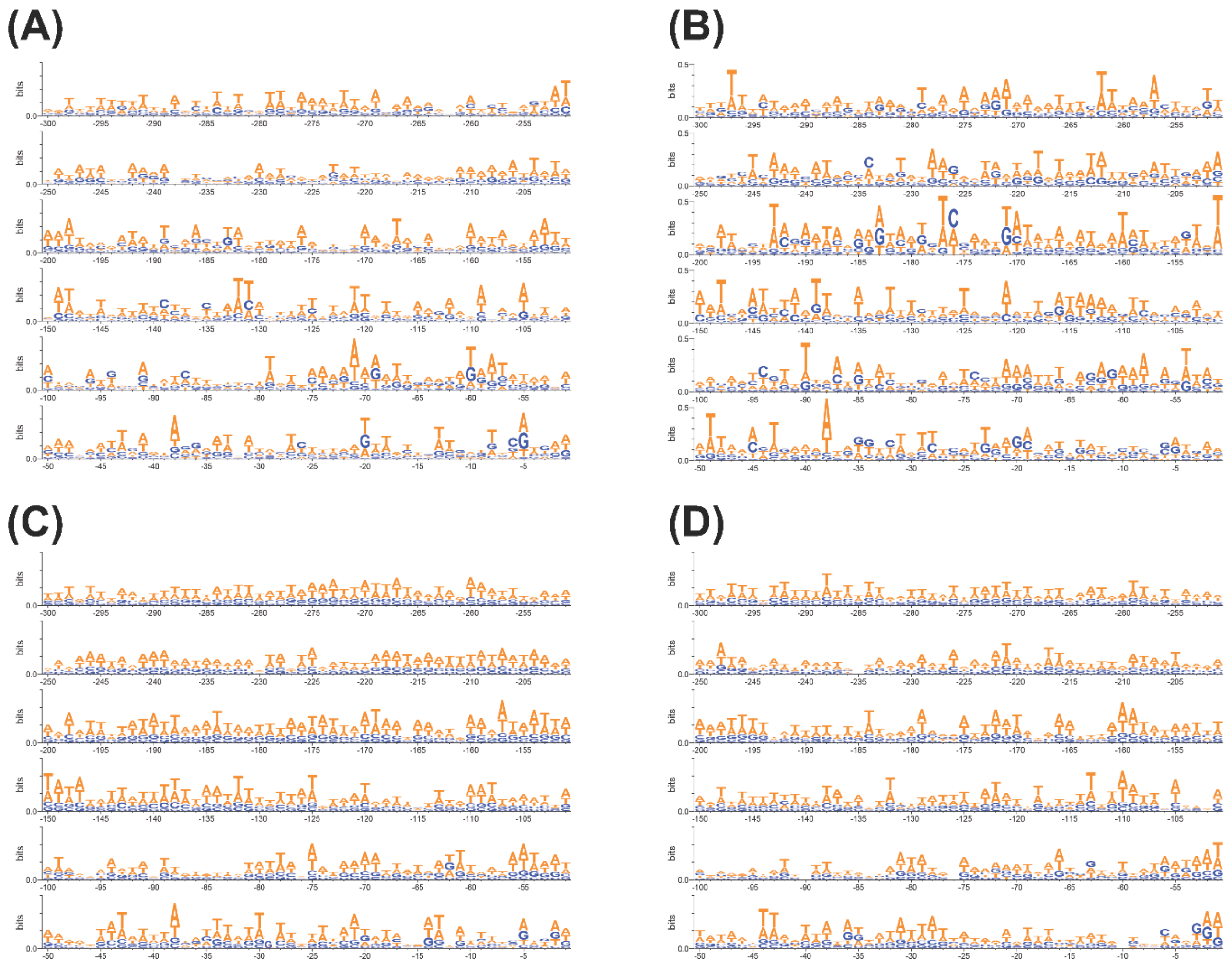

Supplementary Figure 10. Logo plots corresponding to the first 300 nucleotides upstream of chloroplast tDNAs for the (A) ANA, (B) *Ceratophyllum demersum*, (C) eudicot, and (D) monocot genomes.

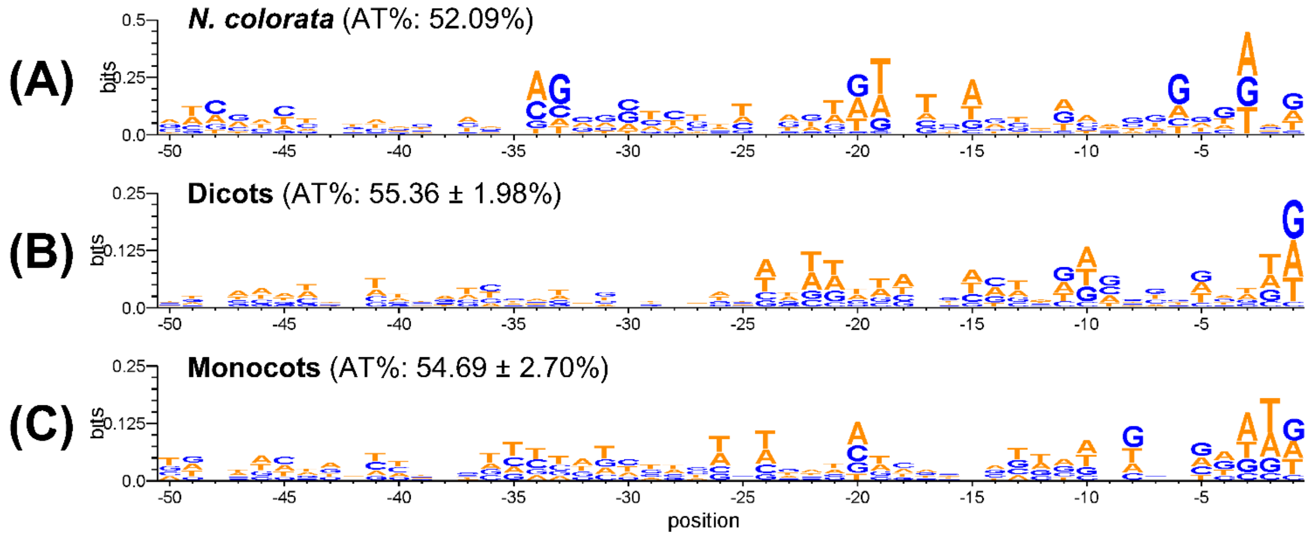

Supplementary Figure 11. Nature of the region 50 nucleotides upstream of mitochondrial tDNAs. Logo plots corresponding to the first 50 nucleotides upstream of mitochondrial tDNAs and their A/T content (in percentage) are shown for the (A) *N. colorata*, (B) eudicot, and (C) monocot genomes.

Only *N. colorata* is shown for ANA, as it is the only one in the group with an available mitochondrial genome (Supplementary Table 1).

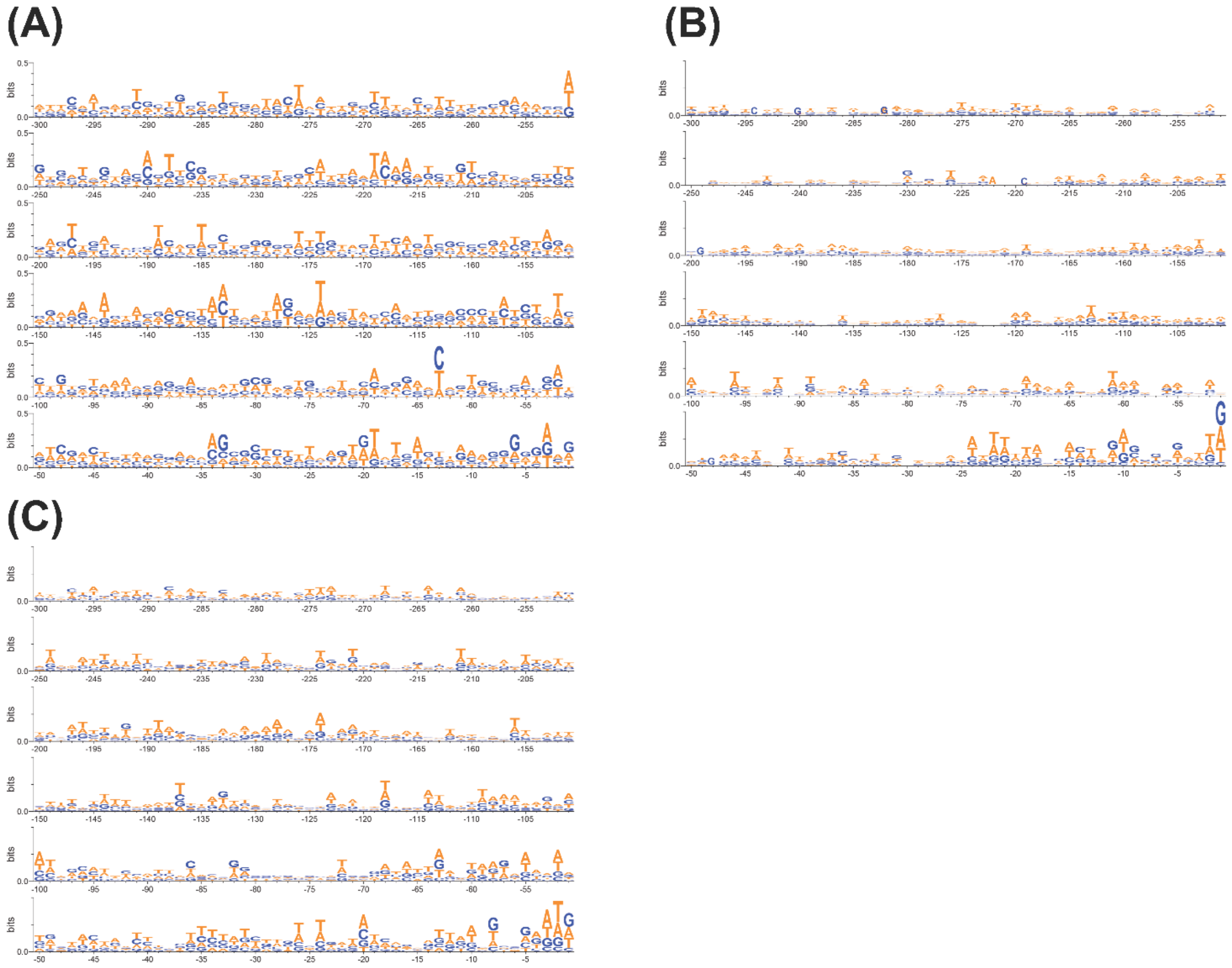

Supplementary Figure 12. Logo plots corresponding to the first 300 nucleotides upstream of mitochondrial tDNAs for (A) ANA, (B) *Ceratophyllum*, (C) eudicots, and (D) monocots. Only *N.*

colorata is shown for ANA as it is the only one in the group with an available mitochondrial genome (Supplementary Table 1).

1.2 Supplementary Tables

Supplementary Table 1. Plant genomes used for determining tRNA genes. Grey boxes indicate the availability of genomes. Numbers next to "P" and "N" indicate Phytozome genome IDs and NCBI accession IDs, respectively.

Lineage	Species	Nuclear	Chloroplast	Mitochondrial
**Dicots**	*Aquilegia coerulea*	P: 322	N: NC_041528.1	
	Arachis hypogaea	P: 530	N: NC_037358.1	
	Cicer arietinum	P: 492	N: NC_011163.1	
	Trifolium pratense	P: 385		N: NC_048499.1
	Lotus japonicus	P: 571	N: NC_002694.1	N: NC_016743.2
	Phaseolus vulgaris	P: 442	N: NC_009259.1	N: NC_045135.1
	Carya illinoinensis	P: 573	N: NC_041449.1	
	Cucumis sativus	P: 122	N: NC_007144.1	N: NC_016005.1
	Fragaris vesca	P: 677	N: NC_015206.1	
	Prunus persica	P: 298	N: NC_014697.1	
	Linum usitatissimum	P: 200	N: NC_036356.1	
	Manihot esculenta	P: 671	N: NC_010433.1	N: NC_045136.1
	Populus trichocarpa	P: 533	N: NC_009143.1	
	Salix purpurea	P: 519	N: NC_026722.1	N: NC_029693.1
	Eucalyptus grandis	P: 297	N: NC_014570.1	N: NC_040010.1
	Anacardium occidentale	P: 449	N: NC_035235.1	
	Citrus sinensis	P: 154	N: NC_008334.1	N: NC_037463.1
	Arabidopsis thaliana	P: 447	N: NC_000932.1	N: NC_037304.1
	Capsella grandiflora	P: 266	N: NC_028517.1	
	Malcolmia maritima	P: 477	N: NC_049671.1	
	Boechera stricta	P: 278	N: NC_049599.1	N: NC_042143.1

	Lepidium sativum	P: 478	N: NC_047178.1	
	Iberis amara	P: 485	N: NC_049655.1	
	Diptychocarpus strictus	P: 582	N: NC_049632.1	
	Brassica rapa	P: 197	N: NC_040849.1	N: NC_049892.1
	Cakile maritima	P: 481	N: NC_049614.1	
	Sinapsis alba	P: 584	N: NC_045948.1	
	Isatis tinctoria	N: GCA_010577795.1	N: NC_028415.1	
	Myagrum perfoliatum	P: 583	N: NC_049676.1	
	Schrenkiella parvula	P: 574	N: NC_028726.1	
	Thlaspi arvense	P: 479	N: NC_034362.1	
	Lunaria annua	P: 476	N: NC_049659.1	
	Carica papaya	P: 113	N: NC_010323.1	N: NC_012116.1
	Gossypium hirsutum	P: 578	N: NC_007944.1	N: NC_027406.1
	Gossypium raimondii	P: 221	N: NC_016668.1	N: NC_029998.1
	Theobroma cacao	P: 523	N: NC_014676.2	
	Coffea arabica	N: GCA_003713225.1	N: NC_008535.1	
	Solanum lycopersicum	P: 691	N: NC_007898.3	N: NC_035963.1
	Olea europaea	P: 451	N: NC_013707.2	
	Daucus carota	P: 388	N: NC_008325.1	N: NC_017855.1
	Helianthus annuus	P: 494	N: NC_007977.1	N: NC_023337.1
	Lactuca sativa	P: 467	N: NC_007578.1	
	Chenopodium quinoa	P: 392	N: NC_034949.1	N: NC_041093.1
	Spinacia oleracea	P: 575		N: NC_035618.1
**Monocots**	*Acorus americanus*	P: 586	N: NC_010093.1	
	Ananas comosus	P: 321	N: NC_026220.1	

	Alloteropsis semialata	N: GCA_004135705.1	N: NC_027824.1	N: QPGU01000688.1
	Panicum virgatum	P: 516	N: NC_015990.1	
	Setaria viridis	P: 500	N: NC_028075.1	
	Miscanthus sinensis	P: 497	N: NC_028721.1	
	Sorghum bicolor	P: 454	N: NC_008602.1	N: NC_008360.1
	Zea mays	P: 493	N: NC_001666.2	N: NC_007982.1
	Paspalum vaginatum	P: 672	N: NC_039462.1	
	Brachypodium distachyon	P: 556	N: NC_011032.1	
	Hordeum vulgare	P: 462	N: NC_008590.1	
	Thinopyrum intermedium	P: 503	N: NC_049153.1	
	Triticum aestivum	N: GCA_018294505.1	N: NC_002762.1	N: NC_036024.1
	Oryza sativa	P: 323	N: NC_031333.1	N: NC_066488.1
	Joinvillea ascendens	P: 587	N: NC_031427.1	
	Musa balbisiana	N: GCA_004837865.1	N: NC_028439.1	
	Asparagus officinalis	P: 498	N: NC_034777.1	N: NC_053642.1
	Dioscorea alata	P: 550	N: NC_039707.1	
	Spirodela polyrhiza	P: 290	N: NC_015891.1	N: NC_017840.1
	Zostera marina	P: 668	N: NC_036014.1	N: NC_035345.1
**Others**	*Ceratophyllum demersum*	N: PRJNA552433	N: NC_009962.1	
	Euryale ferox	N: PRJNA552436	N: NC_037719.1	
	Nymphaea colorata	P: 566	N: NC_057562.1	N: NC_037468.1
	Nymphaea thermarum	N: GCA_011799765.1	N: NC_056953.1	
	Amborella trichopoda	P: 291	N: NC_005086.1	

Supplementary Table 2. Accession IDs of the matK and rbcL genes used for phylogenetic tree construction.

Supplementary Material

**Species**	**Accession**	**Gene**	**Size (bp)**
Aquilegia coerulea	NC_041528.1	*matK*	1530
		rbcL	1428
Arachis hypogaea	NC_037358.1	*matK*	1530
		rbcL	1428
Anacardium occidentale	NC_035235.1	*matK*	1548
		rbcL	1428
Arabidopsis thaliana	NC_000932.1	*matK*	1515
		rbcL	1440
Brassica rapa	NC_040849.1	*matK*	1575
		rbcL	1440
Boechera stricta	NC_049599.1	*matK*	1515
		rbcL	1437
Coffea arabica	NC_008535.1	*matK*	1518
		rbcL	1446
Cicer arietinum	NC_011163.1	*matK*	1530
		rbcL	1428
Capsella grandiflora	NC_028517.1	*matK*	1581
		rbcL	1440
Carya illinoinensis	NC_041449.1	*matK*	1518
		rbcL	1428
Cakile maritima	NC_049614.1	*matK*	1575
		rbcL	1440
Carica papaya	NC_010323.1	*matK*	1521
		rbcL	1428
Chenopodium quinoa	NC_034949.1	*matK*	1527
		rbcL	1428
Cucumis sativus	NC_007144.1	*matK*	1539
		rbcL	1431
Citrus sinensis	NC_008334.1	*matK*	1530
		rbcL	1428
Daucus carota	NC_008325.1	*matK*	1539
		rbcL	1428
Diptychocarpus strictus	NC_049632.1	*matK*	1578
		rbcL	1440
Eucalyptus grandis	NC_014570.1	*matK*	1512
		rbcL	1428
Fragaria vesca	NC_015206.1	*matK*	1503
		rbcL	1428
Gossypium hirsutum	NC_007944.1	*matK*	1515
		rbcL	1443
Gossypium raimondii	NC_016668.1	*matK*	1515
		rbcL	1440
Helianthus annuus	NC_007977.1	*matK*	1503

		rbcL	1458
Iberis amara	NC_049655.1	*matK*	1587
		rbcL	1440
Isatis tinctoria	NC_028415.1	*matK*	1572
		rbcL	1440
Lunaria annua	NC_049659.1	*matK*	1512
		rbcL	1440
Lotus japonicus	NC_002694.1	*matK*	1527
		rbcL	1428
Lactuca sativa	NC_007578.1	*matK*	1521
		rbcL	1434
Lepidium sativum	NC_047178.1	*matK*	1581
		rbcL	1440
Linum usitatissimum	NC_036356.1	*matK*	1566
		rbcL	1428
Manihot esculenta	NC_010433.1	*matK*	1521
		rbcL	1434
Malcolmia maritima	NC_049671.1	*matK*	1581
		rbcL	1440
Myagrum perfoliatum	NC_049676.1	*matK*	1572
		rbcL	1440
Olea europaea	NC_013707.2	*matK*	1578
		rbcL	1428
Prunus persica	NC_014697.1	*matK*	1521
		rbcL	1428
Populus trichocarpa	NC_009143.1	*matK*	1533
		rbcL	1428
Phaseolus vulgaris	NC_009259.1	*matK*	1542
		rbcL	1431
Sinapis alba	NC_045948.1	*matK*	1575
		rbcL	1440
Solanum lycopersicum	NC_007898.3	*matK*	1530
		rbcL	1434
Spinacia oleracea	NC_002202.1	*matK*	1518
		rbcL	1428
Schrenkiella parvula	NC_028726.1	*matK*	1575
		rbcL	1440
Salix purpurea	NC_026722.1	*matK*	1533
		rbcL	1437
Thlaspi arvense	NC_034362.1	*matK*	1512
		rbcL	1440
Theobroma cacao	NC_014676.2	*matK*	1509
		rbcL	1455
Trifolium pratense	NC_047412.1	*matK*	1521
		rbcL	1428

Acorus americanus	NC_010093.1	*matK*	1536
		rbcL	1443
Ananas comosus	NC_026220.1	*matK*	1536
		rbcL	1440
Asparagus officinalis	NC_034777.1	*matK*	1557
		rbcL	1443
Alloteropsis semialata	NC_027824.1	*matK*	1545
		rbcL	1431
Brachypodium distachyon	NC_011032.1	*matK*	1536
		rbcL	1431
Dioscorea alata	NC_039707.1	*matK*	1560
		rbcL	1434
Hordeum vulgare	NC_008590.1	*matK*	1536
		rbcL	1440
Joinvillea ascendens	NC_031427.1	*matK*	1542
		rbcL	1434
Musa balbisiana	NC_028439.1	*matK*	1536
		rbcL	1464
Miscanthus sinensis	NC_028721.1	*matK*	1548
		rbcL	1431
Oryza sativa	NC_031333.1	*matK*	1536
		rbcL	1434
Paspalum vaginatum	NC_039462.1	*matK*	1542
		rbcL	1431
Panicum virgatum	NC_015990.1	*matK*	1542
		rbcL	1434
Sorghum bicolor	NC_008602.1	*matK*	1548
		rbcL	1431
Spirodela polyrhiza	NC_015891.1	*matK*	1533
		rbcL	1461
Setaria viridis	NC_028075.1	*matK*	1542
		rbcL	1428
Triticum aestivum	NC_002762.1	*matK*	1536
		rbcL	1434
Thinopyrum intermedium	NC_049153.1	*matK*	1539
		rbcL	1437
Zostera marina	NC_036014.1	*matK*	1503
		rbcL	1452
Zea mays	NC_001666.2	*matK*	1542
		rbcL	1431
Amborella trichopoda	NC_005086.1	*matK*	1506
		rbcL	1428
Ceratophyllum demersum	NC_009962.1	*matK*	1548
		rbcL	1428

Euryale ferox	NC_037719.1	*matK*	1524
		rbcL	1428
Nymphaea colorata	NC_057562.1	*matK*	1530
		rbcL	1428
Nymphaea thermarum	NC_056953.1	*matK*	1530
		rbcL	1428

Supplementary Table 3. Long tDNA^{eMet} and tDNA^{Tyr} introns in plant nuclear genomes.

**Isotype**	Size (bp)	Sequence	Found in
tRNA^{eMet}	81	5’-GCTAGATTGAGCGATCCTGGAACGGTGGCTGAGCCATGGGCCCGCGCACCTAGGGATAAATATCTCAGGGCTAAGTGAGTA-3’	*H. vulgare*
	76	5’-GCTGCATTGAGCGATGCCGTGGCTGGGCGGTGGGGCCGGCGCACGTTGAGGATAAATATCTCAGAGCTAGTGAGTA-3’	*T. aestivum*
	86	5’-GCAGAATTGAGCGATCCTGGGACGGTGTGGCTGAGCCTGGGGCCGGCGCACGCGTGGGAGGATAAAGATCTCAGAGCTAGTGAGTA-3’	*T. intermedium*
	83	5’-GCTGAATTGAGCGATCCTGGGACGGTGTGGCTGAGCCTGGGGCCGGCACACGTAGAGGATAAAGATCTCAGAGCTAGTGAGTA-3’	
	59	5’-GCTGGATTGAGCGATCCTCCCACGTGTGGGGAGGATAAAGATCTCAGGGCTAGTGAGTA-3’	
tRNA^{Tyr}	85	5’-TAGGTTGCAACCTTCCGCTAATCCTCAGTTCGCTGGTTCAAATCTGGTAGGGCTGAGGGCTGTAGTCGGTTGTAAGCTTTAGCTA-3’	*G. hirsutum*<br>*G. raimondii*
	172	5’-TGTCTGCAGATTATCAGCCTGTTCGTTTGCAGCCTGTTCGTTTGCCTGTGGCTCGTCGTAAACGATCGTAAATTTTCAATCGAAACAGTATTTTTCTCTCATATAAATCAGCCAGCAGTACTTCTTCACGAATCAGCAATGATACGAACCAGCCAACCGAACAGGCTGTATA-3’	*M. sinensis*

	64	5’-TGTCTGCAGATCAATCCTTAGGTCGCTNNNNNNNNNNG CGGAGGACTGTAGTGTCTGCAGATCA-3’	

Supplementary Table 4. A/T content (in percentage) of sequences 50 nucleotides upstream of tDNAs.

Lineage	A/T% (50 nt upstream)
ANA	67.71 ± 1.89%
Dicot	72.79 ± 2.55%
Monocot	70.79 ± 4.14%

Supplementary Table 5. A and B box sequences in angiosperm tDNAs that differ significantly from their corresponding consensus sequence.

Motif	Isotype	Lineage	Sequence
A Box	Glu	Monocot	5’-TAATCTACTTGTAGC-TGG-3’
	Lys	Dicot	5’-TAGCTCATCGCTCAGCTGG-3’
	Phe	Dicot	5’-TAGCTCAGTT-GGGACTTGG-3’
		Monocot	5’-TAGCTCAGTTGGTACTTGG-3’
	Pro	Monocot	5’-TGG-TCTAG-TGGAGTGG-3’
	Val	Monocot	5’-TGGTGTAGTTCGTGAATTTGG-3’
B Box	Gln	Dicot	5’---TTC-----C-T-3’
	Ile	Dicot	5’-GGTCGGT--------------TCGAGACC-3’
			5’-G----GTTCGAAGTCGCAGGTTCGAAACC-3’
	Phe	Monocot	5’-TGTTCGA-TCCACGATCCA-3’
	Ser	Dicot	5’-GGTTCGACAGT--TCGAACCC-3’

2 Supplementary File Captions

Supplementary File 1. Numbers of tRNA isoacceptors per genome.

Supplementary File 2. Logo plots showing the consensus A box sequences.

Supplementary File 3. Logo plots showing the consensus B box sequences.

Supplementary File 4. Reconciled $tRNA^{Ala\text{-}AGC}$ gene and species trees.

Supplementary File 5. Reconciled $tRNA^{Pro}$ gene and species trees.

Supplementary File 6. Reconciled $tRNA^{Ile}$ gene and species trees.

Supplementary File 7. Gene maps of dicot chloroplast genomes.

Supplementary File 8. Gene maps of monocot chloroplast genomes.

Supplementary File 9. Gene maps of ANA chloroplast genomes, including *Ceratophyllum*.

Supplementary File 10. Gene maps of dicot mitogenomes.

Supplementary File 11. Gene maps of monocot mitogenomes.

Supplementary File 12. Gene map of ANA mitogenomes. Only *N. colorata* is shown for ANA, as it is the only one in the group with an available mitochondrial genome (Supplementary Table 1).
