# Supplementary material for: tRNA gene content, structure, and organization in the flowering plant lineage
Source: Front Plant Sci. 2024 Dec 23;15:1486612. doi: 10.3389/fpls.2024.1486612 (PMC11700998; doi:10.3389/fpls.2024.1486612)
Supplement: Supplementary file 5 [file DataSheet5.pdf]

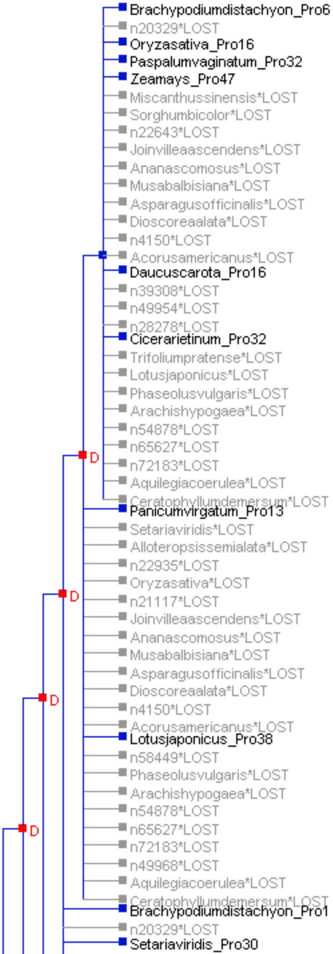
Brachypodiumdistachyon_Pro6
n20329*LOST
Oryzasativa_Pro16
Paspalumvaginatum_Pro32
Zeamays_Pro47
Miscanthussinensis*LOST
Sorghumbicolor*LOST
n22643*LOST
Joinvilleaascendens*LOST
Ananascomosus*LOST
Musabalbisiana*LOST
Asparagusofficinalis*LOST
Dioscoreaalata*LOST
n4150*LOST
Acorusamericanus*LOST
Daucuscarota_Pro16
n39308*LOST
n49954*LOST
n28278*LOST
Cicerarietinum_Pro32
Trifoliumpratense*LOST
Lotusjaponicus*LOST
Phaseolusvulgaris*LOST
Arachishypogaea*LOST
n54878*LOST
n65627*LOST
n72183*LOST
Aquilegiacoerulea*LOST
Ceratophyllumdemersum*LOST
D
Panicumvirgatum_Pro13
Setariaviridis*LOST
Alloteropsissemialata*LOST
n22935*LOST
Oryzasativa*LOST
D
n21117*LOST
Joinvilleaascendens*LOST
Ananascomosus*LOST
Musabalbisiana*LOST
Asparagusofficinalis*LOST
Dioscoreaalata*LOST
D
n4150*LOST
Acorusamericanus*LOST
Lotusjaponicus_Pro38
n58449*LOST
Phaseolusvulgaris*LOST
Arachishypogaea*LOST
n54878*LOST
n65627*LOST
D
n72183*LOST
n49968*LOST
Aquilegiacoerulea*LOST
Ceratophyllumdemersum*LOST
Brachypodiumdistachyon_Pro1
n20329*LOST
Setariaviridis_Pro30

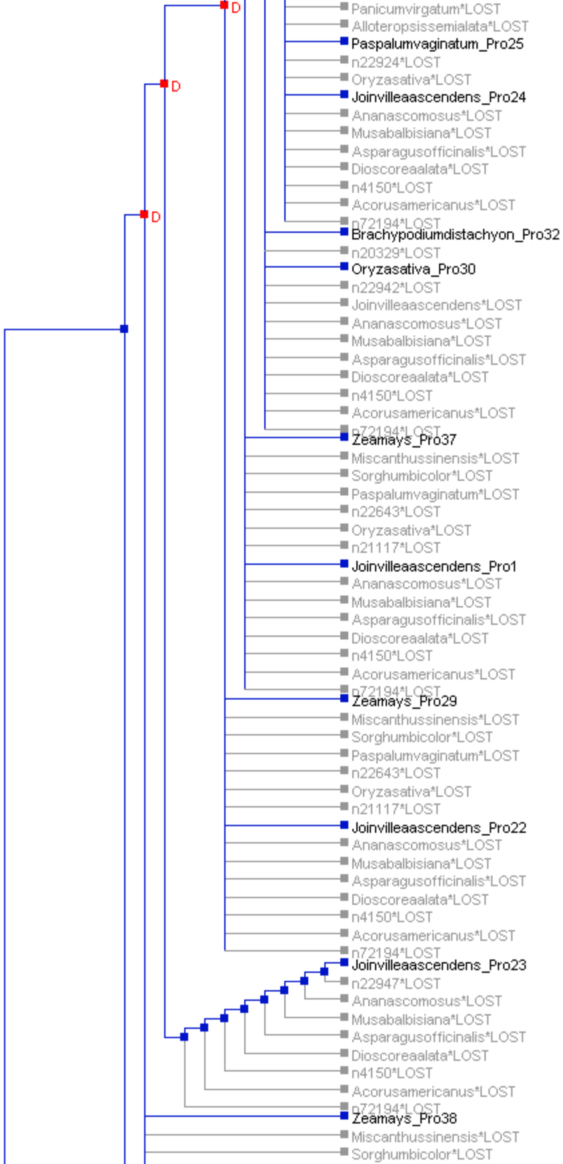
D
D
D
Panicumvirgatum*LOST
Alloteropsissemialata*LOST
Paspalumvaginatum_Pro25
n22924*LOST
Oryzasativa*LOST
Joinvilleaascendens_Pro24
Ananascomosus*LOST
Musabalbisiana*LOST
Asparagusofficinalis*LOST
Dioscoreaalata*LOST
n4150*LOST
Acorusamericanus*LOST
n72194*LOST
Brachypodiumdistachyon_Pro32
n20329*LOST
Oryzasativa_Pro30
n22942*LOST
Joinvilleaascendens*LOST
Ananascomosus*LOST
Musabalbisiana*LOST
Asparagusofficinalis*LOST
Dioscoreaalata*LOST
n4150*LOST
Acorusamericanus*LOST
n72194*LOST
Zeamays_Pro37
Miscanthussinensis*LOST
Sorghumbicolor*LOST
Paspalumvaginatum*LOST
n22643*LOST
Oryzasativa*LOST
n21117*LOST
Joinvilleaascendens_Pro1
Ananascomosus*LOST
Musabalbisiana*LOST
Asparagusofficinalis*LOST
Dioscoreaalata*LOST
n4150*LOST
Acorusamericanus*LOST
n72194*LOST
Zeamays_Pro29
Miscanthussinensis*LOST
Sorghumbicolor*LOST
Paspalumvaginatum*LOST
n22643*LOST
Oryzasativa*LOST
n21117*LOST
Joinvilleaascendens_Pro22
Ananascomosus*LOST
Musabalbisiana*LOST
Asparagusofficinalis*LOST
Dioscoreaalata*LOST
n4150*LOST
Acorusamericanus*LOST
n72194*LOST
Joinvilleaascendens_Pro23
n22947*LOST
Ananascomosus*LOST
Musabalbisiana*LOST
Asparagusofficinalis*LOST
Dioscoreaalata*LOST
n4150*LOST
Acorusamericanus*LOST
n72194*LOST
Zeamays_Pro38
Miscanthussinensis*LOST
Sorghumbicolor*LOST

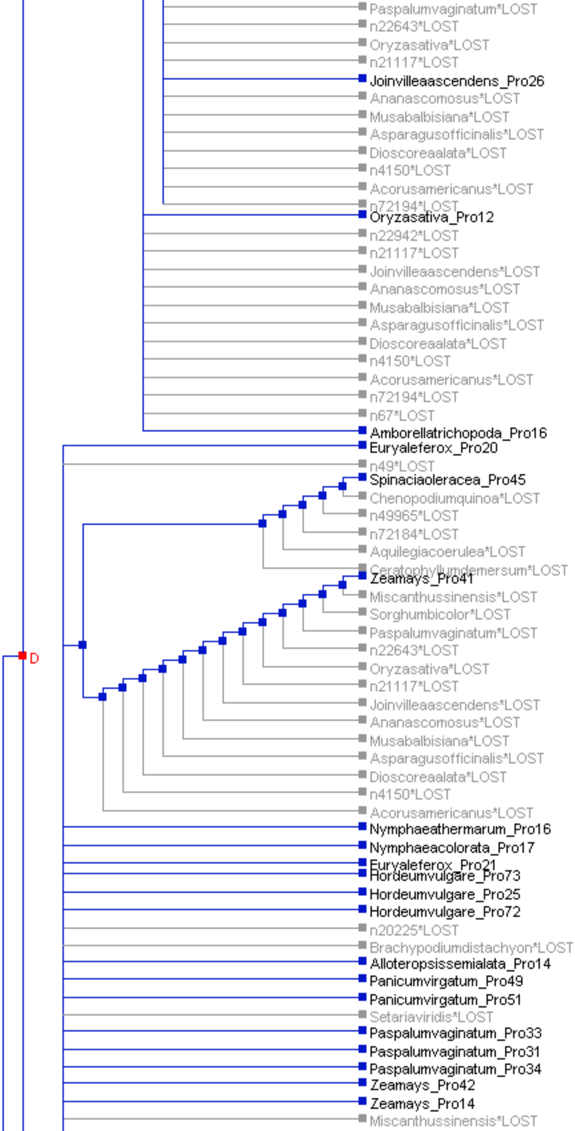
Paspalumvaginatum*LOST
n22643*LOST
Oryzasativa*LOST
n21117*LOST
Joinvilleaascendens_Pro26
Ananascomosus*LOST
Musabalbisiana*LOST
Asparagusofficinalis*LOST
Dioscoreaalata*LOST
n4150*LOST
Acorusamericanus*LOST
n72194*LOST
Oryzasativa_Pro12
n22942*LOST
n21117*LOST
Joinvilleaascendens*LOST
Ananascomosus*LOST
Musabalbisiana*LOST
Asparagusofficinalis*LOST
Dioscoreaalata*LOST
n4150*LOST
Acorusamericanus*LOST
n72194*LOST
n67*LOST
Amborellatrichopoda_Pro16
Euryaleferox_Pro20
n49*LOST
Spinaciaoleracea_Pro45
Chenopodiumquinoa*LOST
n49965*LOST
n72184*LOST
Aquilegiacoerulea*LOST
Ceratophyllumdemersum*LOST
Zeamays_Pro41
Miscanthussinensis*LOST
Sorghumbicolor*LOST
Paspalumvaginatum*LOST
n22643*LOST
Oryzasativa*LOST
n21117*LOST
Joinvilleaascendens*LOST
Ananascomosus*LOST
Musabalbisiana*LOST
Asparagusofficinalis*LOST
Dioscoreaalata*LOST
n4150*LOST
Acorusamericanus*LOST
D
Nymphaeathermarum_Pro16
Nymphaeacolorata_Pro17
Euryaleferox_Pro21
Hordeumvulgare_Pro73
Hordeumvulgare_Pro25
Hordeumvulgare_Pro72
n20225*LOST
Brachypodiumdistachyon*LOST
Alloteropsissemialata_Pro14
Panicumvirgatum_Pro49
Panicumvirgatum_Pro51
Setariaviridis*LOST
Paspalumvaginatum_Pro33
Paspalumvaginatum_Pro31
Paspalumvaginatum_Pro34
Zeamays_Pro42
Zeamays_Pro14
Miscanthussinensis*LOST

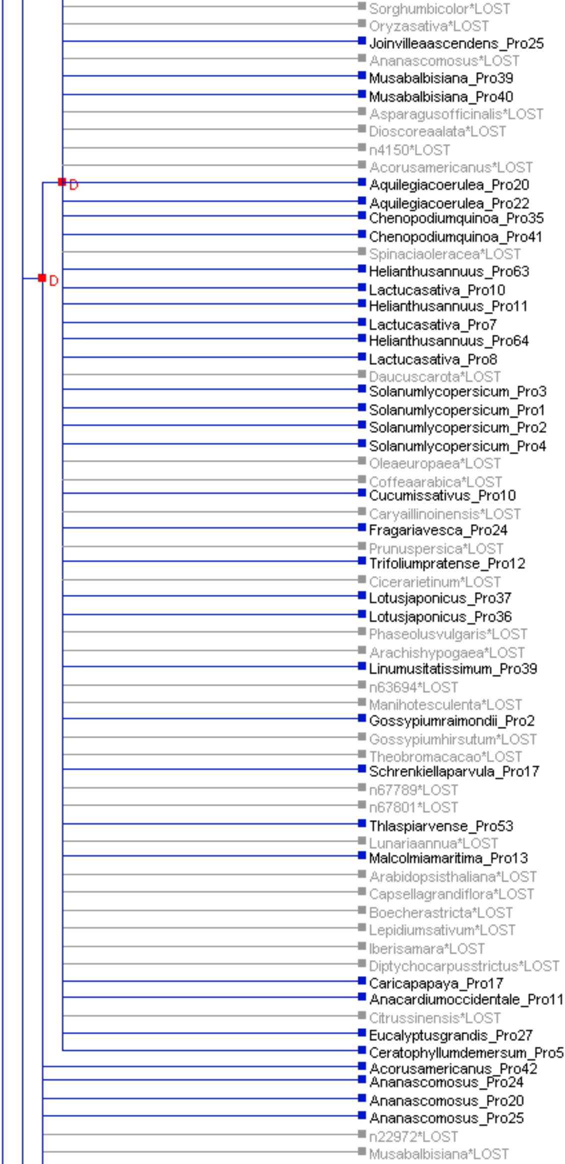
Sorghumbicolor*LOST
Oryzasativa*LOST
Joinvilleaascendens_Pro25
Ananascomosus*LOST
Musabalbisiana_Pro39
Musabalbisiana_Pro40
Asparagusofficinalis*LOST
Dioscoreaalata*LOST
n4150*LOST
Acorusamericanus*LOST
D
Aquilegiacoerulea_Pro20
Aquilegiacoerulea_Pro22
Chenopodiumquinoa_Pro35
Chenopodiumquinoa_Pro41
Spinaciaoleracea*LOST
Helianthusannuus_Pro63
D
Lactucasativa_Pro10
Helianthusannuus_Pro11
Lactucasativa_Pro7
Helianthusannuus_Pro64
Lactucasativa_Pro8
Daucuscarota*LOST
Solanumlycopersicum_Pro3
Solanumlycopersicum_Pro1
Solanumlycopersicum_Pro2
Solanumlycopersicum_Pro4
Oleaeuropaea*LOST
Coffeaarabica*LOST
Cucumissativus_Pro10
Caryaillinoinensis*LOST
Fragariavesca_Pro24
Prunuspersica*LOST
Trifoliumpratense_Pro12
Cicerarietinum*LOST
Lotusjaponicus_Pro37
Lotusjaponicus_Pro36
Phaseolusvulgaris*LOST
Arachishypogaea*LOST
Linumusitatissimum_Pro39
n63694*LOST
Manihotesculenta*LOST
Gossypiumraimondii_Pro2
Gossypiumhirsutum*LOST
Theobromacacao*LOST
Schrenkiellaparvula_Pro17
n67789*LOST
n67801*LOST
Thlaspiarvense_Pro53
Lunariaannua*LOST
Malcolmiamaritima_Pro13
Arabidopsisthaliana*LOST
Capsellagrandiflora*LOST
Boecherastricta*LOST
Lepidiumsativum*LOST
Iberisamara*LOST
Diptychocarpusstrictus*LOST
Caricapapaya_Pro17
Anacardiumoccidentale_Pro11
Citrussinensis*LOST
Eucalyptusgrandis_Pro27
Ceratophyllumdemersum_Pro5
Acorusamericanus_Pro42
Ananascomosus_Pro24
Ananascomosus_Pro20
Ananascomosus_Pro25
n22972*LOST
Musabalbisiana*LOST

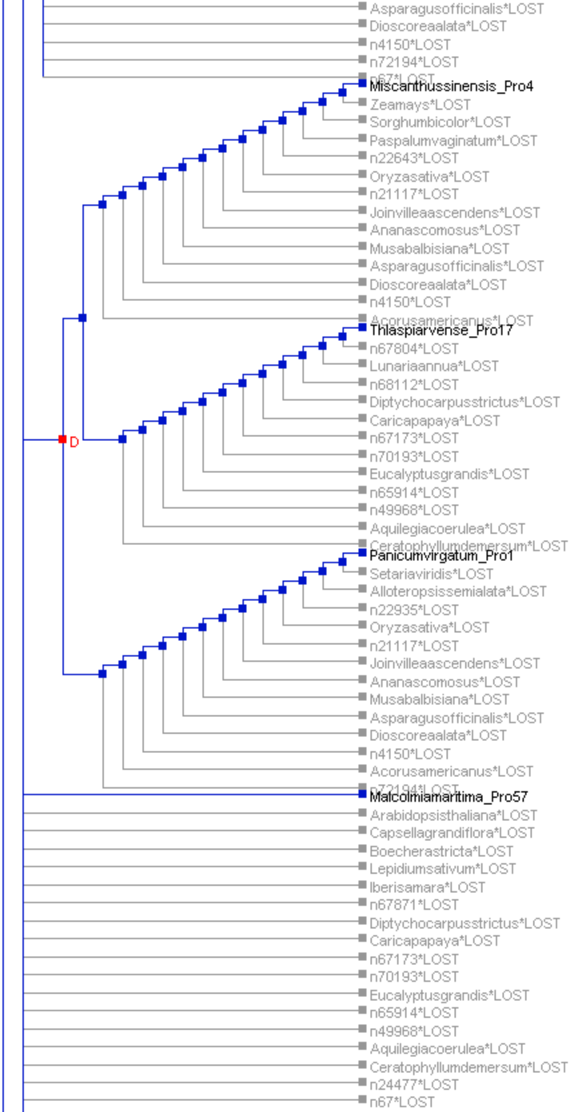
Asparagusofficinalis*LOST
Dioscoreaalata*LOST
n4150*LOST
n72194*LOST
n67*LOST
Miscanthussinensis_Pro4
Zeamays*LOST
Sorghumbicolor*LOST
Paspalumvaginatum*LOST
n22643*LOST
Oryzasativa*LOST
n21117*LOST
Joinvilleaascendens*LOST
Ananascomosus*LOST
Musabalbisiana*LOST
Asparagusofficinalis*LOST
Dioscoreaalata*LOST
n4150*LOST
Acorusamericanus*LOST
Thlaspiarvense_Pro17
n67804*LOST
Lunariaannua*LOST
n68112*LOST
Diptychocarpusstrictus*LOST
Caricapapaya*LOST
n67173*LOST
n70193*LOST
Eucalyptusgrandis*LOST
n65914*LOST
n49968*LOST
Aquilegiacoerulea*LOST
Ceratophyllumdemersum*LOST
D
Panicumvirgatum_Pro1
Setariaviridis*LOST
Alloteropsissemialata*LOST
n22935*LOST
Oryzasativa*LOST
n21117*LOST
Joinvilleaascendens*LOST
Ananascomosus*LOST
Musabalbisiana*LOST
Asparagusofficinalis*LOST
Dioscoreaalata*LOST
n4150*LOST
Acorusamericanus*LOST
n72194*LOST
Malcolmiamaritima_Pro57
Arabidopsisthaliana*LOST
Capsellagrandiflora*LOST
Boecherastricta*LOST
Lepidiumsativum*LOST
Iberisamara*LOST
n67871*LOST
Diptychocarpusstrictus*LOST
Caricapapaya*LOST
n67173*LOST
n70193*LOST
Eucalyptusgrandis*LOST
n65914*LOST
n49968*LOST
Aquilegiacoerulea*LOST
Ceratophyllumdemersum*LOST
n24477*LOST
n67*LOST

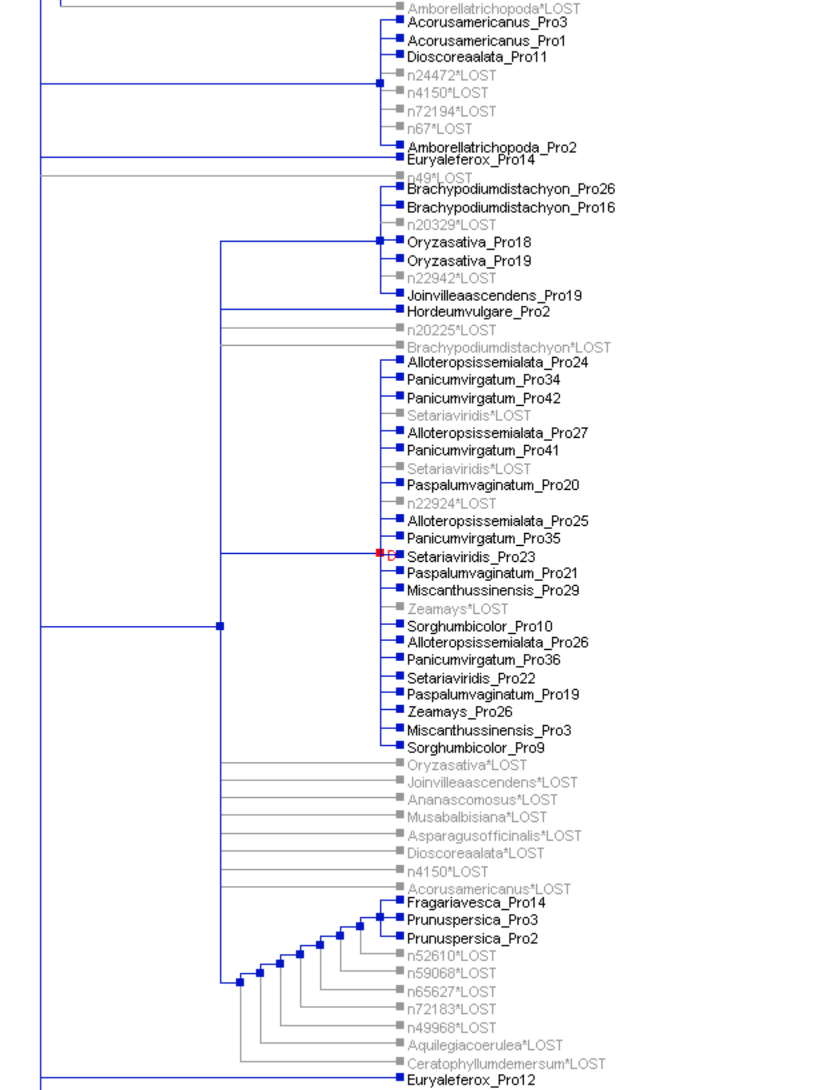

Amborellatrichopoda*LOST
Acorusamericanus_Pro3
Acorusamericanus_Pro1
Dioscoreaalata_Pro11
n24472*LOST
n4150*LOST
n72194*LOST
n67*LOST
Amborellatrichopoda_Pro2
Euryaleferox_Pro14
n49*LOST
Brachypodiumdistachyon_Pro26
Brachypodiumdistachyon_Pro16
n20329*LOST
Oryzasativa_Pro18
Oryzasativa_Pro19
n22942*LOST
Joinvilleaascendens_Pro19
Hordeumvulgare_Pro2
n20225*LOST
Brachypodiumdistachyon*LOST
Alloteropsissemialata_Pro24
Panicumvirgatum_Pro34
Panicumvirgatum_Pro42
Setariaviridis*LOST
Alloteropsissemialata_Pro27
Panicumvirgatum_Pro41
Setariaviridis*LOST
Paspalumvaginatum_Pro20
n22924*LOST
Alloteropsissemialata_Pro25
Panicumvirgatum_Pro35
D
Setariaviridis_Pro23
Paspalumvaginatum_Pro21
Miscanthussinensis_Pro29
Zeamays*LOST
Sorghumbicolor_Pro10
Alloteropsissemialata_Pro26
Panicumvirgatum_Pro36
Setariaviridis_Pro22
Paspalumvaginatum_Pro19
Zeamays_Pro26
Miscanthussinensis_Pro3
Sorghumbicolor_Pro9
Oryzasativa*LOST
Joinvilleaascendens*LOST
Ananascomosus*LOST
Musabalbisiana*LOST
Asparagusofficinalis*LOST
Dioscoreaalata*LOST
n4150*LOST
Acorusamericanus*LOST
Fragariavesca_Pro14
Prunuspersica_Pro3
Prunuspersica_Pro2
n52610*LOST
n59068*LOST
n65627*LOST
n72183*LOST
n49968*LOST
Aquilegiacoerulea*LOST
Ceratophyllumdemersum*LOST
Euryaleferox_Pro12

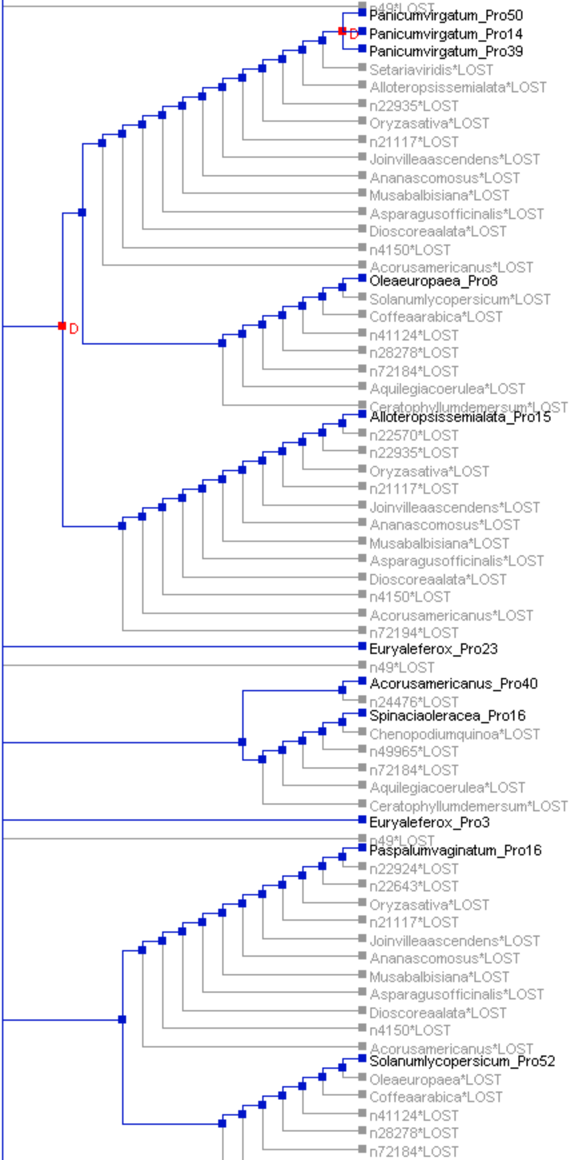

n49*LOST
Panicumvirgatum_Pro50
D
Panicumvirgatum_Pro14
Panicumvirgatum_Pro39
Setariaviridis*LOST
Alloteropsissemialata*LOST
n22935*LOST
Oryzasativa*LOST
n21117*LOST
Joinvilleaascendens*LOST
Ananascomosus*LOST
Musabalbisiana*LOST
Asparagusofficinalis*LOST
Dioscoreaalata*LOST
n4150*LOST
Acorusamericanus*LOST
Oleaeuropaea_Pro8
D
Solanumlycopersicum*LOST
Coffeaarabica*LOST
n41124*LOST
n28278*LOST
n72184*LOST
Aquilegiacoerulea*LOST
Ceratophyllumdemersum*LOST
Alloteropsissemialata_Pro15
n22570*LOST
n22935*LOST
Oryzasativa*LOST
n21117*LOST
Joinvilleaascendens*LOST
Ananascomosus*LOST
Musabalbisiana*LOST
Asparagusofficinalis*LOST
Dioscoreaalata*LOST
n4150*LOST
Acorusamericanus*LOST
n72194*LOST
Euryaleferox_Pro23
n49*LOST
Acorusamericanus_Pro40
n24476*LOST
Spinaciaoleracea_Pro16
Chenopodiumquinoa*LOST
n49965*LOST
n72184*LOST
Aquilegiacoerulea*LOST
Ceratophyllumdemersum*LOST
Euryaleferox_Pro3
n49*LOST
Paspalumvaginatum_Pro16
n22924*LOST
n22643*LOST
Oryzasativa*LOST
n21117*LOST
Joinvilleaascendens*LOST
Ananascomosus*LOST
Musabalbisiana*LOST
Asparagusofficinalis*LOST
Dioscoreaalata*LOST
n4150*LOST
Acorusamericanus*LOST
Solanumlycopersicum_Pro52
Oleaeuropaea*LOST
Coffeaarabica*LOST
n41124*LOST
n28278*LOST
n72184*LOST

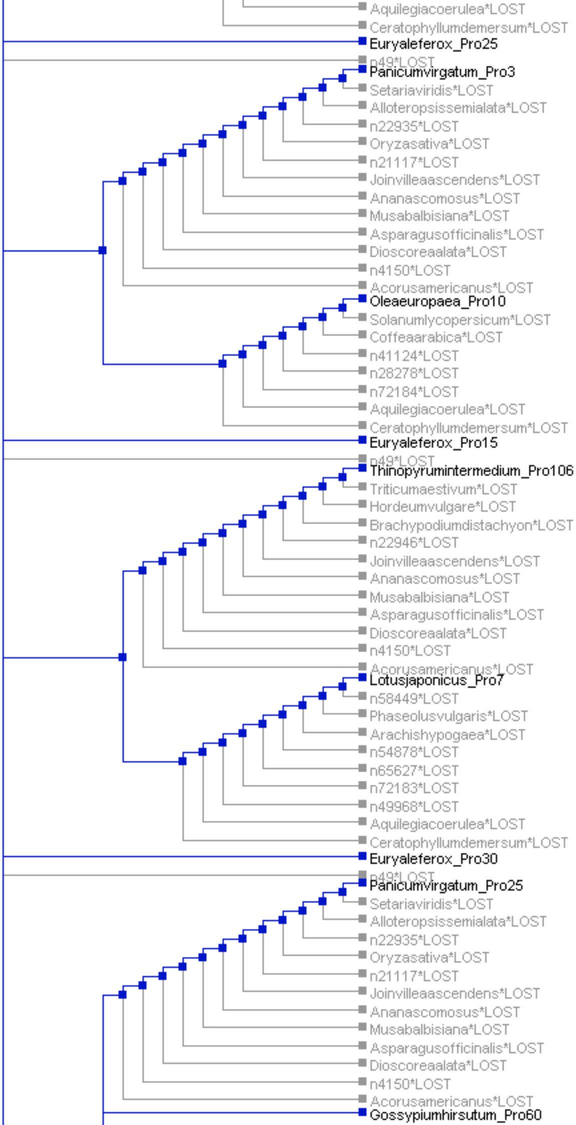
Aquilegiacoerulea*LOST
Ceratophyllumdemersum*LOST
Euryaleferox_Pro25
n49*LOST
Panicumvirgatum_Pro3
Setariaviridis*LOST
Alloteropsissemialata*LOST
n22935*LOST
Oryzasativa*LOST
n21117*LOST
Joinvilleaascendens*LOST
Ananascomosus*LOST
Musabalbisiana*LOST
Asparagusofficinalis*LOST
Dioscoreaalata*LOST
n4150*LOST
Acorusamericanus*LOST
Oleaeuropaea_Pro10
Solanumlycopersicum*LOST
Coffeaarabica*LOST
n41124*LOST
n28278*LOST
n72184*LOST
Aquilegiacoerulea*LOST
Ceratophyllumdemersum*LOST
Euryaleferox_Pro15
n49*LOST
Thinopyrumintermedium_Pro106
Triticumaestivum*LOST
Hordeumvulgare*LOST
Brachypodiumdistachyon*LOST
n22946*LOST
Joinvilleaascendens*LOST
Ananascomosus*LOST
Musabalbisiana*LOST
Asparagusofficinalis*LOST
Dioscoreaalata*LOST
n4150*LOST
Acorusamericanus*LOST
Lotusjaponicus_Pro7
n58449*LOST
Phaseolusvulgaris*LOST
Arachishypogaea*LOST
n54878*LOST
n65627*LOST
n72183*LOST
n49968*LOST
Aquilegiacoerulea*LOST
Ceratophyllumdemersum*LOST
Euryaleferox_Pro30
n49*LOST
Panicumvirgatum_Pro25
Setariaviridis*LOST
Alloteropsissemialata*LOST
n22935*LOST
Oryzasativa*LOST
n21117*LOST
Joinvilleaascendens*LOST
Ananascomosus*LOST
Musabalbisiana*LOST
Asparagusofficinalis*LOST
Dioscoreaalata*LOST
n4150*LOST
Acorusamericanus*LOST
Gossypiumhirsutum_Pro60

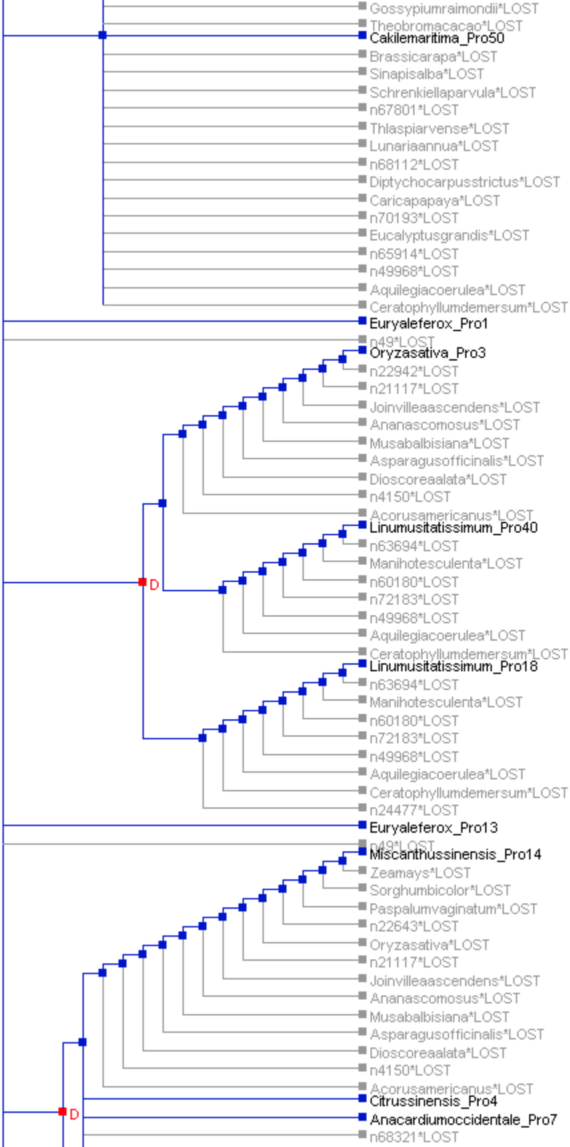
Gossypiumraimondii*LOST
Theobromacacao*LOST
Cakilemaritima_Pro50
Brassicarapa*LOST
Sinapisalba*LOST
Schrenkiellaparvula*LOST
n67801*LOST
Thlaspiarvense*LOST
Lunariaannua*LOST
n68112*LOST
Diptychocarpusstrictus*LOST
Caricapapaya*LOST
n70193*LOST
Eucalyptusgrandis*LOST
n65914*LOST
n49968*LOST
Aquilegiacoerulea*LOST
Ceratophyllumdemersum*LOST
Euryaleferox_Pro1
n49*LOST
Oryzasativa_Pro3
n22942*LOST
n21117*LOST
Joinvilleaascendens*LOST
Ananascomosus*LOST
Musabalbisiana*LOST
Asparagusofficinalis*LOST
Dioscoreaalata*LOST
n4150*LOST
Acorusamericanus*LOST
D
Linumusitatissimum_Pro40
n63694*LOST
Manihotesculenta*LOST
n60180*LOST
n72183*LOST
n49968*LOST
Aquilegiacoerulea*LOST
Ceratophyllumdemersum*LOST
Linumusitatissimum_Pro18
n63694*LOST
Manihotesculenta*LOST
n60180*LOST
n72183*LOST
n49968*LOST
Aquilegiacoerulea*LOST
Ceratophyllumdemersum*LOST
n24477*LOST
Euryaleferox_Pro13
n49*LOST
Miscanthussinensis_Pro14
Zeamays*LOST
Sorghumbicolor*LOST
Paspalumvaginatum*LOST
n22643*LOST
Oryzasativa*LOST
n21117*LOST
Joinvilleaascendens*LOST
Ananascomosus*LOST
Musabalbisiana*LOST
Asparagusofficinalis*LOST
Dioscoreaalata*LOST
n4150*LOST
Acorusamericanus*LOST
D
Citrussinensis_Pro4
Anacardiumoccidentale_Pro7
n68321*LOST

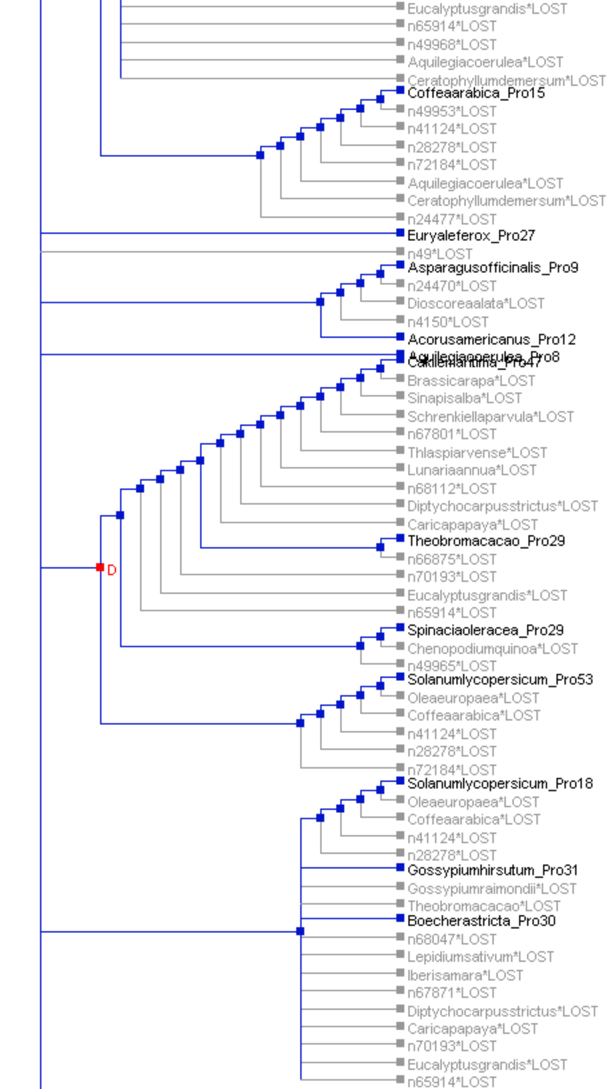
Eucalyptusgrandis*LOST
n65914*LOST
n49968*LOST
Aquilegiacoerulea*LOST
Ceratophyllumdemersum*LOST
Coffeaarabica_Pro15
n49953*LOST
n41124*LOST
n28278*LOST
n72184*LOST
Aquilegiacoerulea*LOST
Ceratophyllumdemersum*LOST
n24477*LOST
Euryaleferox_Pro27
n49*LOST
Asparagusofficinalis_Pro9
n24470*LOST
Dioscoreaalata*LOST
n4150*LOST
Acorusamericanus_Pro12
Aquilegiacoerulea_Pro8
Cakilemaritima_Pro47
Brassicarapa*LOST
Sinapisalba*LOST
Schrenkiellaparvula*LOST
n67801*LOST
Thlaspiarvense*LOST
Lunariaannua*LOST
n68112*LOST
Diptychocarpusstrictus*LOST
Caricapapaya*LOST
Theobromacacao_Pro29
n66875*LOST
n70193*LOST
Eucalyptusgrandis*LOST
n65914*LOST
D
Spinaciaoleracea_Pro29
Chenopodiumquinoa*LOST
n49965*LOST
Solanumlycopersicum_Pro53
Oleaeuropaea*LOST
Coffeaarabica*LOST
n41124*LOST
n28278*LOST
n72184*LOST
Solanumlycopersicum_Pro18
Oleaeuropaea*LOST
Coffeaarabica*LOST
n41124*LOST
n28278*LOST
Gossypiumhirsutum_Pro31
Gossypiumraimondii*LOST
Theobromacacao*LOST
Boecherastricta_Pro30
n68047*LOST
Lepidiumsativum*LOST
Iberisamara*LOST
n67871*LOST
Diptychocarpusstrictus*LOST
Caricapapaya*LOST
n70193*LOST
Eucalyptusgrandis*LOST
n65914*LOST

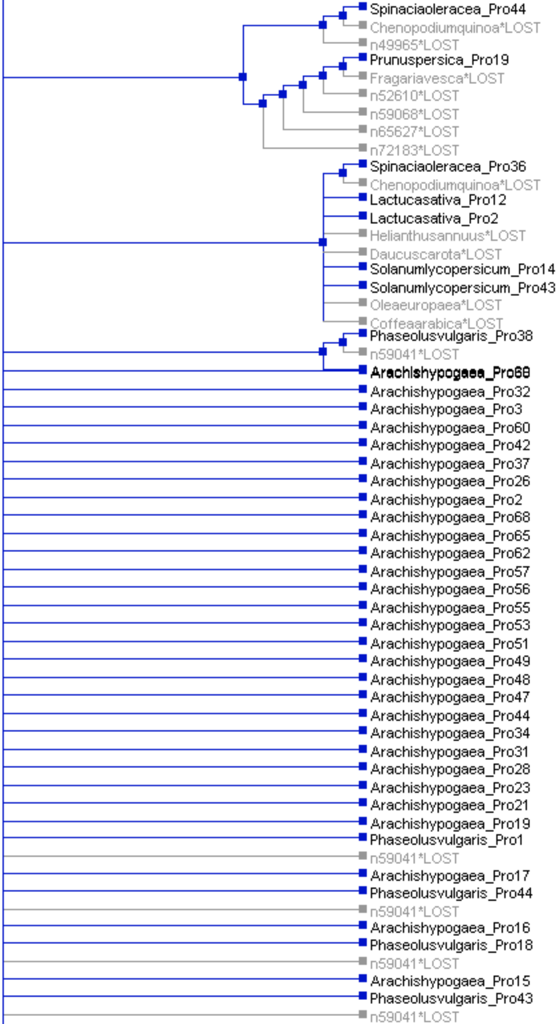
Spinaciaoleracea_Pro44
Chenopodiumquinoa*LOST
n49965*LOST
Prunuspersica_Pro19
Fragariavesca*LOST
n52610*LOST
n59068*LOST
n65627*LOST
n72183*LOST
Spinaciaoleracea_Pro36
Chenopodiumquinoa*LOST
Lactucasativa_Pro12
Lactucasativa_Pro2
Helianthusannuus*LOST
Daucuscarota*LOST
Solanumlycopersicum_Pro14
Solanumlycopersicum_Pro43
Oleaeuropaea*LOST
Coffeaarabica*LOST
Phaseolusvulgaris_Pro38
n59041*LOST
Arachishypogaea_Pro69
Arachishypogaea_Pro32
Arachishypogaea_Pro3
Arachishypogaea_Pro60
Arachishypogaea_Pro42
Arachishypogaea_Pro37
Arachishypogaea_Pro26
Arachishypogaea_Pro2
Arachishypogaea_Pro68
Arachishypogaea_Pro65
Arachishypogaea_Pro62
Arachishypogaea_Pro57
Arachishypogaea_Pro56
Arachishypogaea_Pro55
Arachishypogaea_Pro53
Arachishypogaea_Pro51
Arachishypogaea_Pro49
Arachishypogaea_Pro48
Arachishypogaea_Pro47
Arachishypogaea_Pro44
Arachishypogaea_Pro34
Arachishypogaea_Pro31
Arachishypogaea_Pro28
Arachishypogaea_Pro23
Arachishypogaea_Pro21
Arachishypogaea_Pro19
Phaseolusvulgaris_Pro1
n59041*LOST
Arachishypogaea_Pro17
Phaseolusvulgaris_Pro44
n59041*LOST
Arachishypogaea_Pro16
Phaseolusvulgaris_Pro18
n59041*LOST
Arachishypogaea_Pro15
Phaseolusvulgaris_Pro43
n59041*LOST

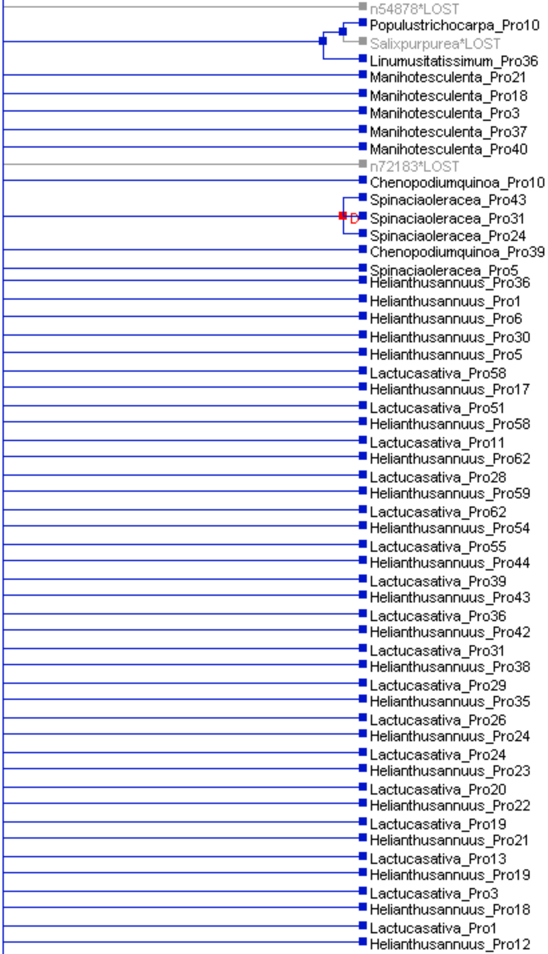
n54878*LOST
Populustrichocarpa_Pro10
Salixpurpurea*LOST
Linumusitatissimum_Pro36
Manihotesculenta_Pro21
Manihotesculenta_Pro18
Manihotesculenta_Pro3
Manihotesculenta_Pro37
Manihotesculenta_Pro40
n72183*LOST
Chenopodiumquinoa_Pro10
Spinaciaoleracea_Pro43
D
Spinaciaoleracea_Pro31
Spinaciaoleracea_Pro24
Chenopodiumquinoa_Pro39
Spinaciaoleracea_Pro5
Helianthusannuus_Pro36
Helianthusannuus_Pro1
Helianthusannuus_Pro6
Helianthusannuus_Pro30
Helianthusannuus_Pro5
Lactucasativa_Pro58
Helianthusannuus_Pro17
Lactucasativa_Pro51
Helianthusannuus_Pro58
Lactucasativa_Pro11
Helianthusannuus_Pro62
Lactucasativa_Pro28
Helianthusannuus_Pro59
Lactucasativa_Pro62
Helianthusannuus_Pro54
Lactucasativa_Pro55
Helianthusannuus_Pro44
Lactucasativa_Pro39
Helianthusannuus_Pro43
Lactucasativa_Pro36
Helianthusannuus_Pro42
Lactucasativa_Pro31
Helianthusannuus_Pro38
Lactucasativa_Pro29
Helianthusannuus_Pro35
Lactucasativa_Pro26
Helianthusannuus_Pro24
Lactucasativa_Pro24
Helianthusannuus_Pro23
Lactucasativa_Pro20
Helianthusannuus_Pro22
Lactucasativa_Pro19
Helianthusannuus_Pro21
Lactucasativa_Pro13
Helianthusannuus_Pro19
Lactucasativa_Pro3
Helianthusannuus_Pro18
Lactucasativa_Pro1
Helianthusannuus_Pro12

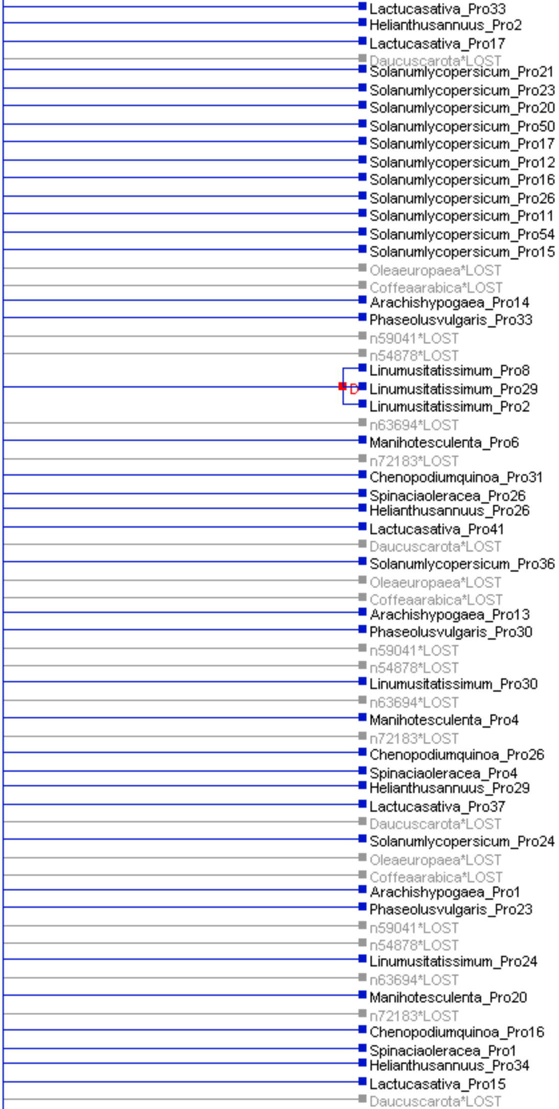

Lactucasativa_Pro33
Helianthusannuus_Pro2
Lactucasativa_Pro17
Daucuscarota*LOST
Solanumlycopersicum_Pro21
Solanumlycopersicum_Pro23
Solanumlycopersicum_Pro20
Solanumlycopersicum_Pro50
Solanumlycopersicum_Pro17
Solanumlycopersicum_Pro12
Solanumlycopersicum_Pro16
Solanumlycopersicum_Pro26
Solanumlycopersicum_Pro11
Solanumlycopersicum_Pro54
Solanumlycopersicum_Pro15
Oleaeuropaea*LOST
Coffeaarabica*LOST
Arachishypogaea_Pro14
Phaseolusvulgaris_Pro33
n59041*LOST
n54878*LOST
Linumusitatissimum_Pro8
D
Linumusitatissimum_Pro29
Linumusitatissimum_Pro2
n63694*LOST
Manihotesculenta_Pro6
n72183*LOST
Chenopodiumquinoa_Pro31
Spinaciaoleracea_Pro26
Helianthusannuus_Pro26
Lactucasativa_Pro41
Daucuscarota*LOST
Solanumlycopersicum_Pro36
Oleaeuropaea*LOST
Coffeaarabica*LOST
Arachishypogaea_Pro13
Phaseolusvulgaris_Pro30
n59041*LOST
n54878*LOST
Linumusitatissimum_Pro30
n63694*LOST
Manihotesculenta_Pro4
n72183*LOST
Chenopodiumquinoa_Pro26
Spinaciaoleracea_Pro4
Helianthusannuus_Pro29
Lactucasativa_Pro37
Daucuscarota*LOST
Solanumlycopersicum_Pro24
Oleaeuropaea*LOST
Coffeaarabica*LOST
Arachishypogaea_Pro1
Phaseolusvulgaris_Pro23
n59041*LOST
n54878*LOST
Linumusitatissimum_Pro24
n63694*LOST
Manihotesculenta_Pro20
n72183*LOST
Chenopodiumquinoa_Pro16
Spinaciaoleracea_Pro1
Helianthusannuus_Pro34
Lactucasativa_Pro15
Daucuscarota*LOST

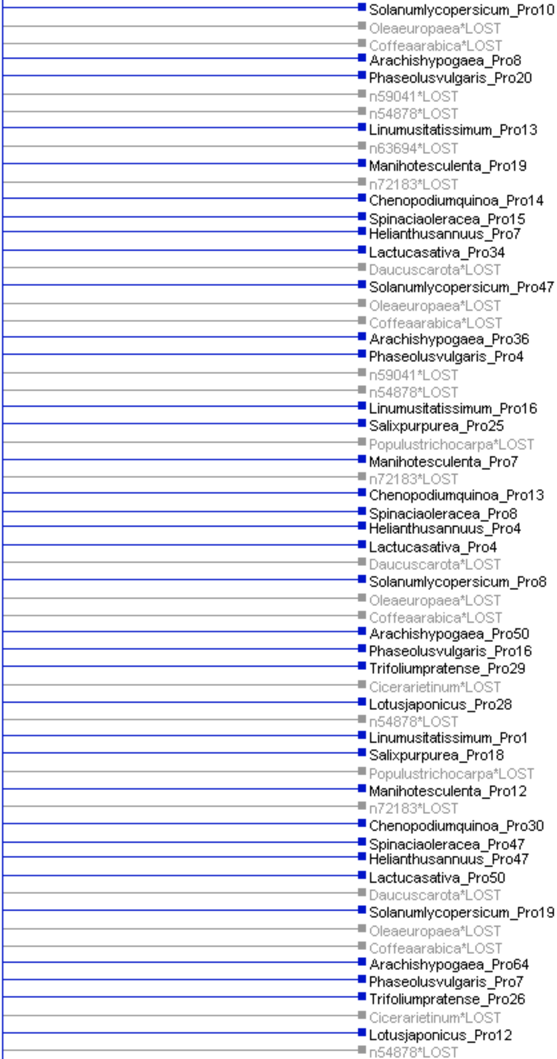
Solanumlycopersicum_Pro10
Oleaeuropaea*LOST
Coffeaarabica*LOST
Arachishypogaea_Pro8
Phaseolusvulgaris_Pro20
n59041*LOST
n54878*LOST
Linumusitatissimum_Pro13
n63694*LOST
Manihotesculenta_Pro19
n72183*LOST
Chenopodiumquinoa_Pro14
Spinaciaoleracea_Pro15
Helianthusannuus_Pro7
Lactucasativa_Pro34
Daucuscarota*LOST
Solanumlycopersicum_Pro47
Oleaeuropaea*LOST
Coffeaarabica*LOST
Arachishypogaea_Pro36
Phaseolusvulgaris_Pro4
n59041*LOST
n54878*LOST
Linumusitatissimum_Pro16
Salixpurpurea_Pro25
Populustrichocarpa*LOST
Manihotesculenta_Pro7
n72183*LOST
Chenopodiumquinoa_Pro13
Spinaciaoleracea_Pro8
Helianthusannuus_Pro4
Lactucasativa_Pro4
Daucuscarota*LOST
Solanumlycopersicum_Pro8
Oleaeuropaea*LOST
Coffeaarabica*LOST
Arachishypogaea_Pro50
Phaseolusvulgaris_Pro16
Trifoliumpratense_Pro29
Cicerarietinum*LOST
Lotusjaponicus_Pro28
n54878*LOST
Linumusitatissimum_Pro1
Salixpurpurea_Pro18
Populustrichocarpa*LOST
Manihotesculenta_Pro12
n72183*LOST
Chenopodiumquinoa_Pro30
Spinaciaoleracea_Pro47
Helianthusannuus_Pro47
Lactucasativa_Pro50
Daucuscarota*LOST
Solanumlycopersicum_Pro19
Oleaeuropaea*LOST
Coffeaarabica*LOST
Arachishypogaea_Pro64
Phaseolusvulgaris_Pro7
Trifoliumpratense_Pro26
Cicerarietinum*LOST
Lotusjaponicus_Pro12
n54878*LOST

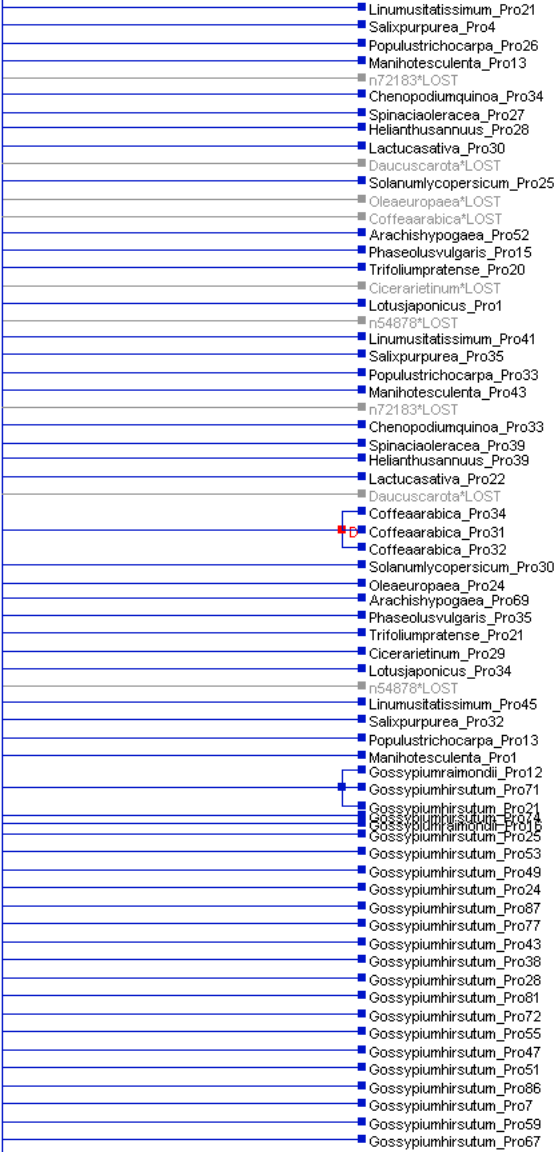

Linumusitatissimum_Pro21
Salixpurpurea_Pro4
Populustrichocarpa_Pro26
Manihotesculenta_Pro13
n72183*LOST
Chenopodiumquinoa_Pro34
Spinaciaoleracea_Pro27
Helianthusannuus_Pro28
Lactucasativa_Pro30
Daucuscarota*LOST
Solanumlycopersicum_Pro25
Oleaeuropaea*LOST
Coffeaarabica*LOST
Arachishypogaea_Pro52
Phaseolusvulgaris_Pro15
Trifoliumpratense_Pro20
Cicerarietinum*LOST
Lotusjaponicus_Pro1
n54878*LOST
Linumusitatissimum_Pro41
Salixpurpurea_Pro35
Populustrichocarpa_Pro33
Manihotesculenta_Pro43
n72183*LOST
Chenopodiumquinoa_Pro33
Spinaciaoleracea_Pro39
Helianthusannuus_Pro39
Lactucasativa_Pro22
Daucuscarota*LOST
Coffeaarabica_Pro34
D
Coffeaarabica_Pro31
Coffeaarabica_Pro32
Solanumlycopersicum_Pro30
Oleaeuropaea_Pro24
Arachishypogaea_Pro69
Phaseolusvulgaris_Pro35
Trifoliumpratense_Pro21
Cicerarietinum_Pro29
Lotusjaponicus_Pro34
n54878*LOST
Linumusitatissimum_Pro45
Salixpurpurea_Pro32
Populustrichocarpa_Pro13
Manihotesculenta_Pro1
Gossypiumraimondii_Pro12
Gossypiumhirsutum_Pro71
Gossypiumhirsutum_Pro21
Gossypiumhirsutum_Pro74
Gossypiumraimondii_Pro16
Gossypiumhirsutum_Pro25
Gossypiumhirsutum_Pro53
Gossypiumhirsutum_Pro49
Gossypiumhirsutum_Pro24
Gossypiumhirsutum_Pro87
Gossypiumhirsutum_Pro77
Gossypiumhirsutum_Pro43
Gossypiumhirsutum_Pro38
Gossypiumhirsutum_Pro28
Gossypiumhirsutum_Pro81
Gossypiumhirsutum_Pro72
Gossypiumhirsutum_Pro55
Gossypiumhirsutum_Pro47
Gossypiumhirsutum_Pro51
Gossypiumhirsutum_Pro86
Gossypiumhirsutum_Pro7
Gossypiumhirsutum_Pro59
Gossypiumhirsutum_Pro67

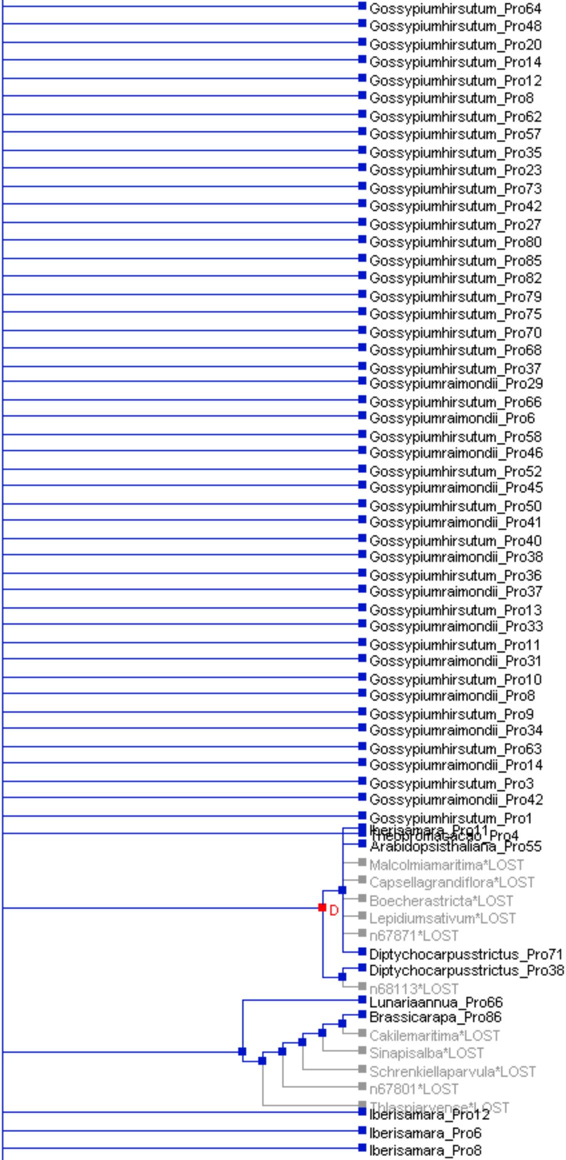
Gossypiumhirsutum_Pro64
Gossypiumhirsutum_Pro48
Gossypiumhirsutum_Pro20
Gossypiumhirsutum_Pro14
Gossypiumhirsutum_Pro12
Gossypiumhirsutum_Pro8
Gossypiumhirsutum_Pro62
Gossypiumhirsutum_Pro57
Gossypiumhirsutum_Pro35
Gossypiumhirsutum_Pro23
Gossypiumhirsutum_Pro73
Gossypiumhirsutum_Pro42
Gossypiumhirsutum_Pro27
Gossypiumhirsutum_Pro80
Gossypiumhirsutum_Pro85
Gossypiumhirsutum_Pro82
Gossypiumhirsutum_Pro79
Gossypiumhirsutum_Pro75
Gossypiumhirsutum_Pro70
Gossypiumhirsutum_Pro68
Gossypiumhirsutum_Pro37
Gossypiumraimondii_Pro29
Gossypiumhirsutum_Pro66
Gossypiumraimondii_Pro6
Gossypiumhirsutum_Pro58
Gossypiumraimondii_Pro46
Gossypiumhirsutum_Pro52
Gossypiumraimondii_Pro45
Gossypiumhirsutum_Pro50
Gossypiumraimondii_Pro41
Gossypiumhirsutum_Pro40
Gossypiumraimondii_Pro38
Gossypiumhirsutum_Pro36
Gossypiumraimondii_Pro37
Gossypiumhirsutum_Pro13
Gossypiumraimondii_Pro33
Gossypiumhirsutum_Pro11
Gossypiumraimondii_Pro31
Gossypiumhirsutum_Pro10
Gossypiumraimondii_Pro8
Gossypiumhirsutum_Pro9
Gossypiumraimondii_Pro34
Gossypiumhirsutum_Pro63
Gossypiumraimondii_Pro14
Gossypiumhirsutum_Pro3
Gossypiumraimondii_Pro42
Gossypiumhirsutum_Pro1
Iberisamara_Pro11
Theobromacacao_Pro4
Arabidopsisthaliana_Pro55
Malcolmiamaritima*LOST
Capsellagrandiflora*LOST
D
Boecherastricta*LOST
Lepidiumsativum*LOST
n67871*LOST
Diptychocarpusstrictus_Pro71
Diptychocarpusstrictus_Pro38
n68113*LOST
Lunariaannua_Pro66
Brassicarapa_Pro86
Cakilemaritima*LOST
Sinapisalba*LOST
Schrenkiellaparvula*LOST
n67801*LOST
Thlaspiarvense*LOST
Iberisamara_Pro12
Iberisamara_Pro6
Iberisamara_Pro8

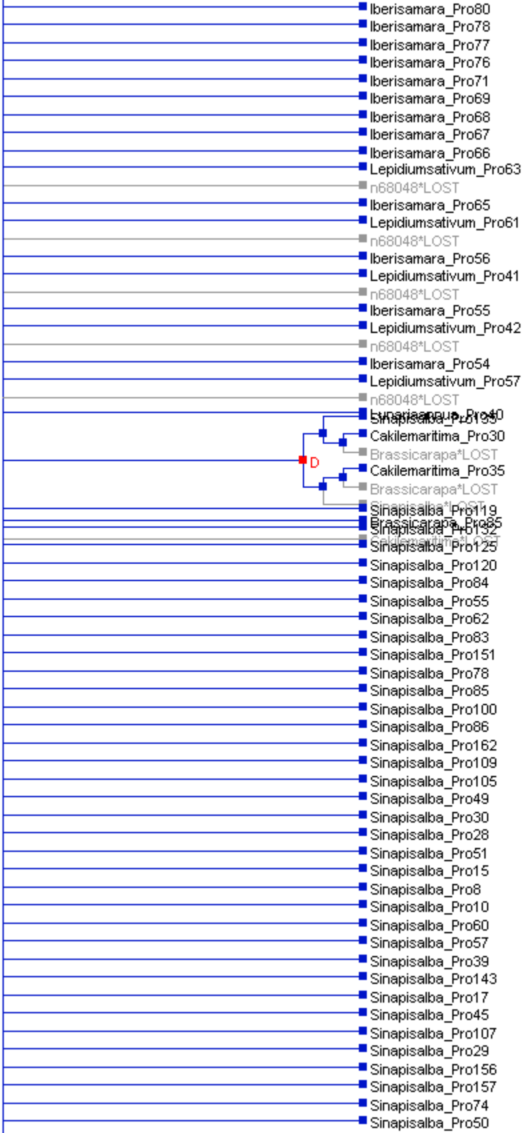

Iberisamara_Pro80
Iberisamara_Pro78
Iberisamara_Pro77
Iberisamara_Pro76
Iberisamara_Pro71
Iberisamara_Pro69
Iberisamara_Pro68
Iberisamara_Pro67
Iberisamara_Pro66
Lepidiumsativum_Pro63
n68048*LOST
Iberisamara_Pro65
Lepidiumsativum_Pro61
n68048*LOST
Iberisamara_Pro56
Lepidiumsativum_Pro41
n68048*LOST
Iberisamara_Pro55
Lepidiumsativum_Pro42
n68048*LOST
Iberisamara_Pro54
Lepidiumsativum_Pro57
n68048*LOST
Lunariaannua_Pro40
Sinapisalba_Pro135
Cakilemaritima_Pro30
Brassicarapa*LOST
D
Cakilemaritima_Pro35
Brassicarapa*LOST
Sinapisalba*LOST
Sinapisalba_Pro119
Brassicarapa_Pro85
Sinapisalba_Pro132
Cakilemaritima*LOST
Sinapisalba_Pro125
Sinapisalba_Pro120
Sinapisalba_Pro84
Sinapisalba_Pro55
Sinapisalba_Pro62
Sinapisalba_Pro83
Sinapisalba_Pro151
Sinapisalba_Pro78
Sinapisalba_Pro85
Sinapisalba_Pro100
Sinapisalba_Pro86
Sinapisalba_Pro162
Sinapisalba_Pro109
Sinapisalba_Pro105
Sinapisalba_Pro49
Sinapisalba_Pro30
Sinapisalba_Pro28
Sinapisalba_Pro51
Sinapisalba_Pro15
Sinapisalba_Pro8
Sinapisalba_Pro10
Sinapisalba_Pro60
Sinapisalba_Pro57
Sinapisalba_Pro39
Sinapisalba_Pro143
Sinapisalba_Pro17
Sinapisalba_Pro45
Sinapisalba_Pro107
Sinapisalba_Pro29
Sinapisalba_Pro156
Sinapisalba_Pro157
Sinapisalba_Pro74
Sinapisalba_Pro50

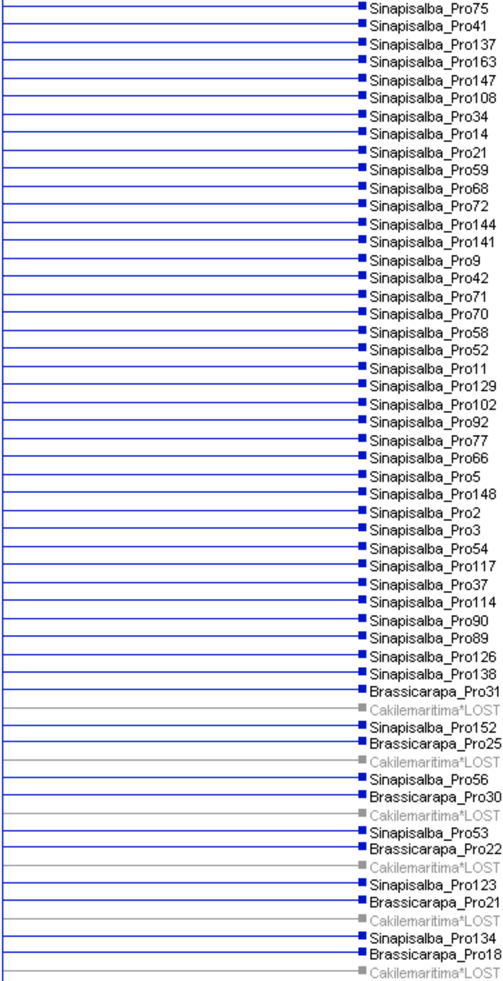
Sinapisalba_Pro75
Sinapisalba_Pro41
Sinapisalba_Pro137
Sinapisalba_Pro163
Sinapisalba_Pro147
Sinapisalba_Pro108
Sinapisalba_Pro34
Sinapisalba_Pro14
Sinapisalba_Pro21
Sinapisalba_Pro59
Sinapisalba_Pro68
Sinapisalba_Pro72
Sinapisalba_Pro144
Sinapisalba_Pro141
Sinapisalba_Pro9
Sinapisalba_Pro42
Sinapisalba_Pro71
Sinapisalba_Pro70
Sinapisalba_Pro58
Sinapisalba_Pro52
Sinapisalba_Pro11
Sinapisalba_Pro129
Sinapisalba_Pro102
Sinapisalba_Pro92
Sinapisalba_Pro77
Sinapisalba_Pro66
Sinapisalba_Pro5
Sinapisalba_Pro148
Sinapisalba_Pro2
Sinapisalba_Pro3
Sinapisalba_Pro54
Sinapisalba_Pro117
Sinapisalba_Pro37
Sinapisalba_Pro114
Sinapisalba_Pro90
Sinapisalba_Pro89
Sinapisalba_Pro126
Sinapisalba_Pro138
Brassicarapa_Pro31
Cakilemaritima*LOST
Sinapisalba_Pro152
Brassicarapa_Pro25
Cakilemaritima*LOST
Sinapisalba_Pro56
Brassicarapa_Pro30
Cakilemaritima*LOST
Sinapisalba_Pro53
Brassicarapa_Pro22
Cakilemaritima*LOST
Sinapisalba_Pro123
Brassicarapa_Pro21
Cakilemaritima*LOST
Sinapisalba_Pro134
Brassicarapa_Pro18
Cakilemaritima*LOST

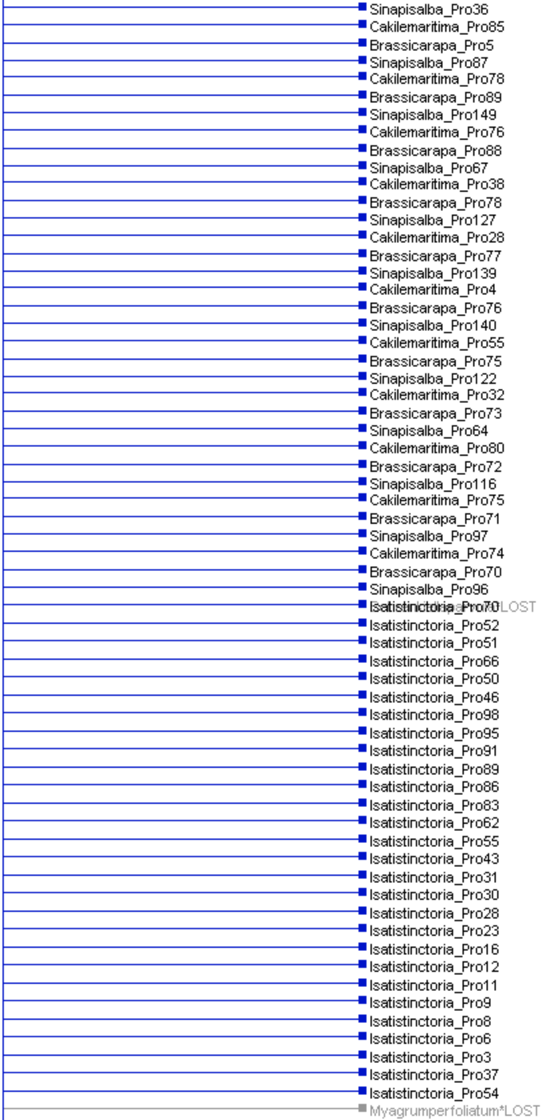
Sinapisalba_Pro36
Cakilemaritima_Pro85
Brassicarapa_Pro5
Sinapisalba_Pro87
Cakilemaritima_Pro78
Brassicarapa_Pro89
Sinapisalba_Pro149
Cakilemaritima_Pro76
Brassicarapa_Pro88
Sinapisalba_Pro67
Cakilemaritima_Pro38
Brassicarapa_Pro78
Sinapisalba_Pro127
Cakilemaritima_Pro28
Brassicarapa_Pro77
Sinapisalba_Pro139
Cakilemaritima_Pro4
Brassicarapa_Pro76
Sinapisalba_Pro140
Cakilemaritima_Pro55
Brassicarapa_Pro75
Sinapisalba_Pro122
Cakilemaritima_Pro32
Brassicarapa_Pro73
Sinapisalba_Pro64
Cakilemaritima_Pro80
Brassicarapa_Pro72
Sinapisalba_Pro116
Cakilemaritima_Pro75
Brassicarapa_Pro71
Sinapisalba_Pro97
Cakilemaritima_Pro74
Brassicarapa_Pro70
Sinapisalba_Pro96
Isatistinctoria_Pro70
LOST
Isatistinctoria_Pro52
Isatistinctoria_Pro51
Isatistinctoria_Pro66
Isatistinctoria_Pro50
Isatistinctoria_Pro46
Isatistinctoria_Pro98
Isatistinctoria_Pro95
Isatistinctoria_Pro91
Isatistinctoria_Pro89
Isatistinctoria_Pro86
Isatistinctoria_Pro83
Isatistinctoria_Pro62
Isatistinctoria_Pro55
Isatistinctoria_Pro43
Isatistinctoria_Pro31
Isatistinctoria_Pro30
Isatistinctoria_Pro28
Isatistinctoria_Pro23
Isatistinctoria_Pro16
Isatistinctoria_Pro12
Isatistinctoria_Pro11
Isatistinctoria_Pro9
Isatistinctoria_Pro8
Isatistinctoria_Pro6
Isatistinctoria_Pro3
Isatistinctoria_Pro37
Isatistinctoria_Pro54
Myagrumperfoliatum*LOST

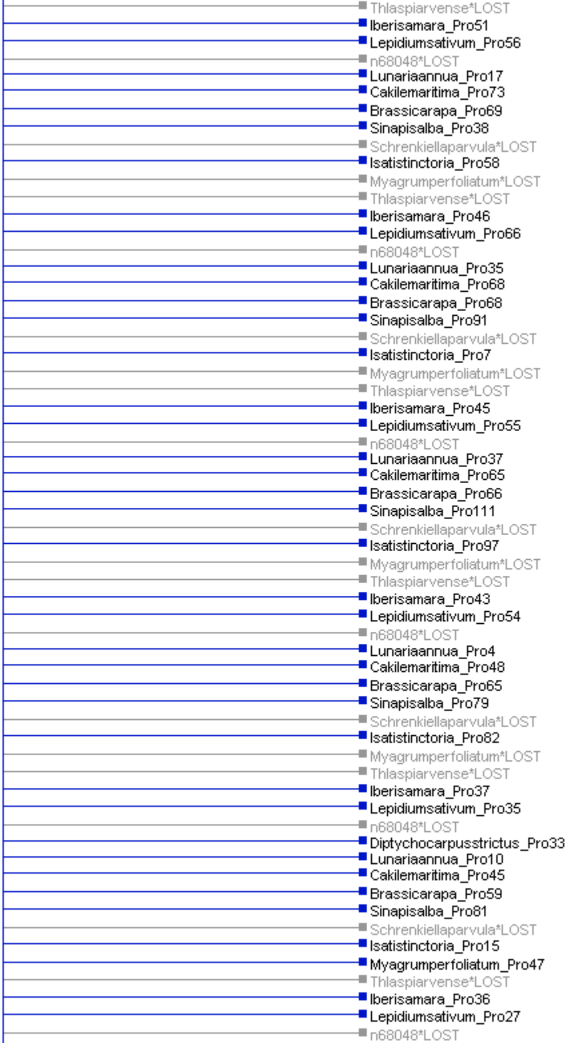

Thlaspiarvense*LOST
Iberisamara_Pro51
Lepidiumsativum_Pro56
n68048*LOST
Lunariaannua_Pro17
Cakilemaritima_Pro73
Brassicarapa_Pro69
Sinapisalba_Pro38
Schrenkiellaparvula*LOST
Isatistinctoria_Pro58
Myagrumperfoliatum*LOST
Thlaspiarvense*LOST
Iberisamara_Pro46
Lepidiumsativum_Pro66
n68048*LOST
Lunariaannua_Pro35
Cakilemaritima_Pro68
Brassicarapa_Pro68
Sinapisalba_Pro91
Schrenkiellaparvula*LOST
Isatistinctoria_Pro7
Myagrumperfoliatum*LOST
Thlaspiarvense*LOST
Iberisamara_Pro45
Lepidiumsativum_Pro55
n68048*LOST
Lunariaannua_Pro37
Cakilemaritima_Pro65
Brassicarapa_Pro66
Sinapisalba_Pro111
Schrenkiellaparvula*LOST
Isatistinctoria_Pro97
Myagrumperfoliatum*LOST
Thlaspiarvense*LOST
Iberisamara_Pro43
Lepidiumsativum_Pro54
n68048*LOST
Lunariaannua_Pro4
Cakilemaritima_Pro48
Brassicarapa_Pro65
Sinapisalba_Pro79
Schrenkiellaparvula*LOST
Isatistinctoria_Pro82
Myagrumperfoliatum*LOST
Thlaspiarvense*LOST
Iberisamara_Pro37
Lepidiumsativum_Pro35
n68048*LOST
Diptychocarpusstrictus_Pro33
Lunariaannua_Pro10
Cakilemaritima_Pro45
Brassicarapa_Pro59
Sinapisalba_Pro81
Schrenkiellaparvula*LOST
Isatistinctoria_Pro15
Myagrumperfoliatum_Pro47
Thlaspiarvense*LOST
Iberisamara_Pro36
Lepidiumsativum_Pro27
n68048*LOST

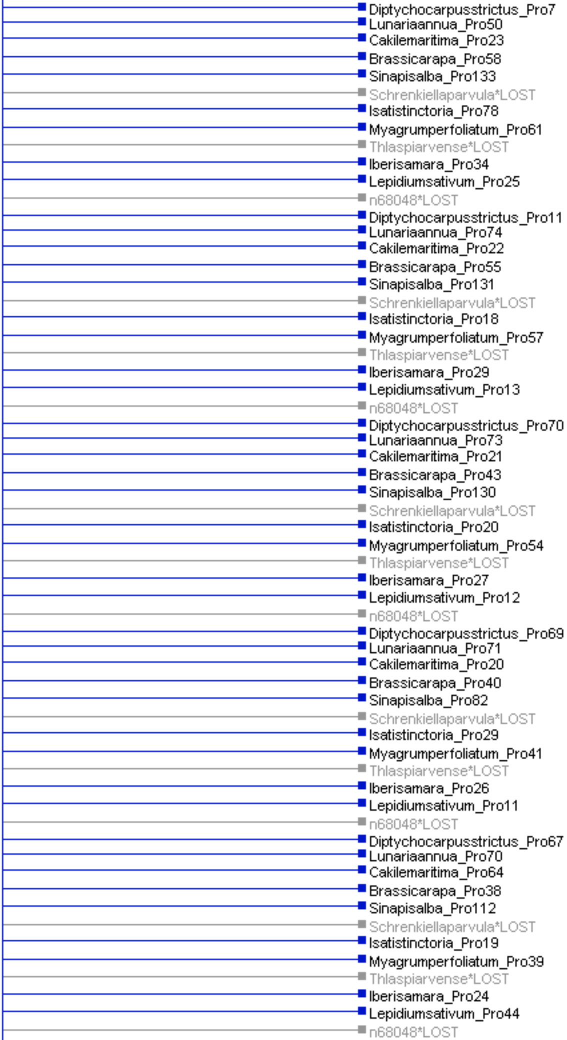

Diptychocarpusstrictus_Pro7
Lunariaannua_Pro50
Cakilemaritima_Pro23
Brassicarapa_Pro58
Sinapisalba_Pro133
Schrenkiellaparvula*LOST
Isatistinctoria_Pro78
Myagrumperfoliatum_Pro61
Thlaspiarvense*LOST
Iberisamara_Pro34
Lepidiumsativum_Pro25
n68048*LOST
Diptychocarpusstrictus_Pro11
Lunariaannua_Pro74
Cakilemaritima_Pro22
Brassicarapa_Pro55
Sinapisalba_Pro131
Schrenkiellaparvula*LOST
Isatistinctoria_Pro18
Myagrumperfoliatum_Pro57
Thlaspiarvense*LOST
Iberisamara_Pro29
Lepidiumsativum_Pro13
n68048*LOST
Diptychocarpusstrictus_Pro70
Lunariaannua_Pro73
Cakilemaritima_Pro21
Brassicarapa_Pro43
Sinapisalba_Pro130
Schrenkiellaparvula*LOST
Isatistinctoria_Pro20
Myagrumperfoliatum_Pro54
Thlaspiarvense*LOST
Iberisamara_Pro27
Lepidiumsativum_Pro12
n68048*LOST
Diptychocarpusstrictus_Pro69
Lunariaannua_Pro71
Cakilemaritima_Pro20
Brassicarapa_Pro40
Sinapisalba_Pro82
Schrenkiellaparvula*LOST
Isatistinctoria_Pro29
Myagrumperfoliatum_Pro41
Thlaspiarvense*LOST
Iberisamara_Pro26
Lepidiumsativum_Pro11
n68048*LOST
Diptychocarpusstrictus_Pro67
Lunariaannua_Pro70
Cakilemaritima_Pro64
Brassicarapa_Pro38
Sinapisalba_Pro112
Schrenkiellaparvula*LOST
Isatistinctoria_Pro19
Myagrumperfoliatum_Pro39
Thlaspiarvense*LOST
Iberisamara_Pro24
Lepidiumsativum_Pro44
n68048*LOST

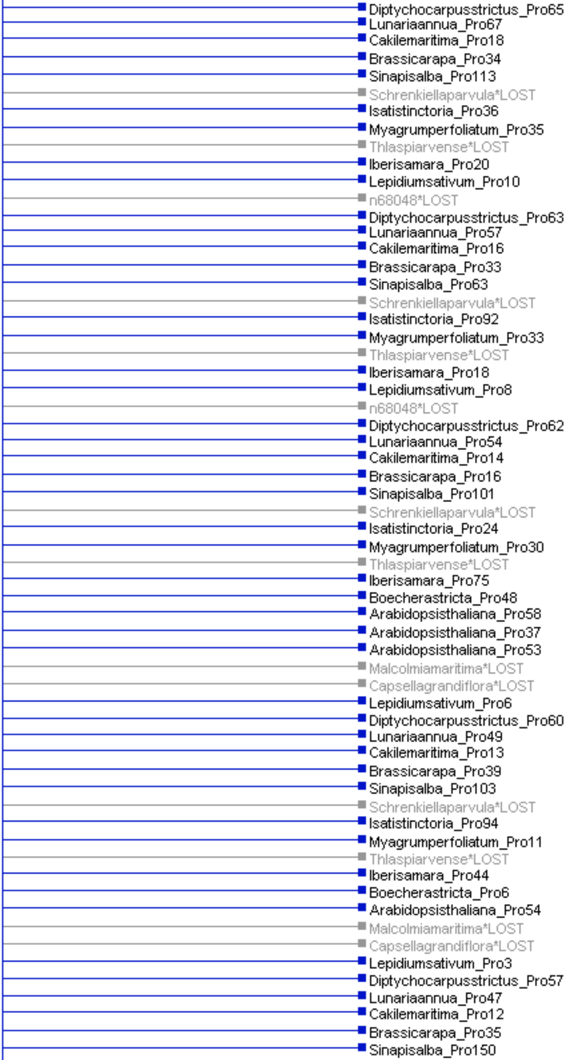

Diptychocarpusstrictus_Pro65
Lunariaannua_Pro67
Cakilemaritima_Pro18
Brassicarapa_Pro34
Sinapisalba_Pro113
Schrenkiellaparvula*LOST
Isatistinctoria_Pro36
Myagrumperfoliatum_Pro35
Thlaspiarvense*LOST
Iberisamara_Pro20
Lepidiumsativum_Pro10
n68048*LOST
Diptychocarpusstrictus_Pro63
Lunariaannua_Pro57
Cakilemaritima_Pro16
Brassicarapa_Pro33
Sinapisalba_Pro63
Schrenkiellaparvula*LOST
Isatistinctoria_Pro92
Myagrumperfoliatum_Pro33
Thlaspiarvense*LOST
Iberisamara_Pro18
Lepidiumsativum_Pro8
n68048*LOST
Diptychocarpusstrictus_Pro62
Lunariaannua_Pro54
Cakilemaritima_Pro14
Brassicarapa_Pro16
Sinapisalba_Pro101
Schrenkiellaparvula*LOST
Isatistinctoria_Pro24
Myagrumperfoliatum_Pro30
Thlaspiarvense*LOST
Iberisamara_Pro75
Boecherastricta_Pro48
Arabidopsisthaliana_Pro58
Arabidopsisthaliana_Pro37
Arabidopsisthaliana_Pro53
Malcolmiamaritima*LOST
Capsellagrandiflora*LOST
Lepidiumsativum_Pro6
Diptychocarpusstrictus_Pro60
Lunariaannua_Pro49
Cakilemaritima_Pro13
Brassicarapa_Pro39
Sinapisalba_Pro103
Schrenkiellaparvula*LOST
Isatistinctoria_Pro94
Myagrumperfoliatum_Pro11
Thlaspiarvense*LOST
Iberisamara_Pro44
Boecherastricta_Pro6
Arabidopsisthaliana_Pro54
Malcolmiamaritima*LOST
Capsellagrandiflora*LOST
Lepidiumsativum_Pro3
Diptychocarpusstrictus_Pro57
Lunariaannua_Pro47
Cakilemaritima_Pro12
Brassicarapa_Pro35
Sinapisalba_Pro150

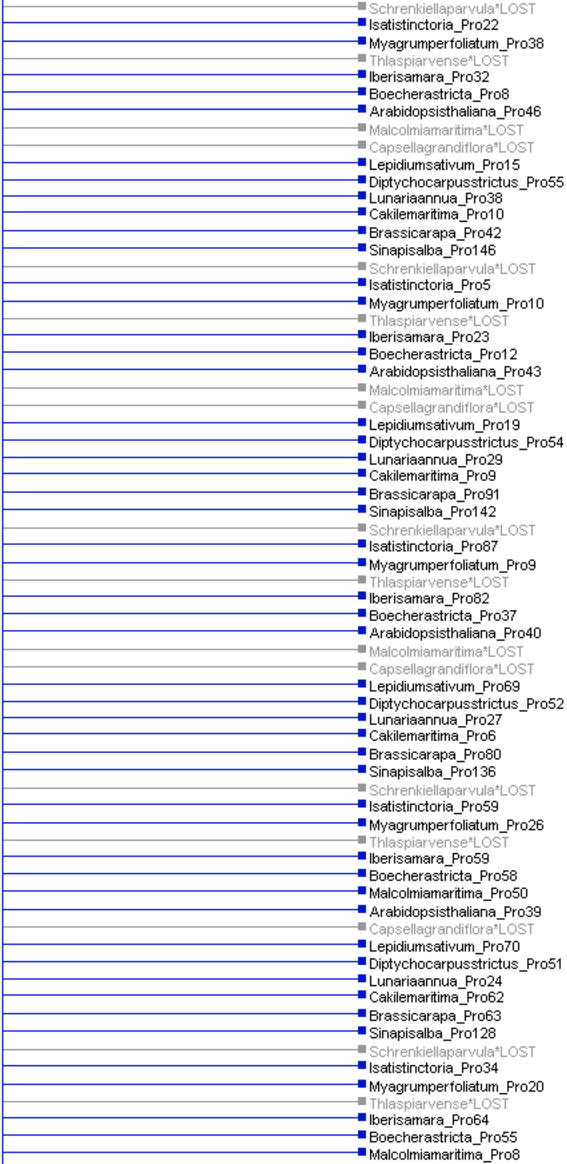

Schrenkiellaparvula*LOST
Isatistinctoria_Pro22
Myagrumperfoliatum_Pro38
Thlaspiarvense*LOST
Iberisamara_Pro32
Boecherastricta_Pro8
Arabidopsisthaliana_Pro46
Malcolmiamaritima*LOST
Capsellagrandiflora*LOST
Lepidiumsativum_Pro15
Diptychocarpusstrictus_Pro55
Lunariaannua_Pro38
Cakilemaritima_Pro10
Brassicarapa_Pro42
Sinapisalba_Pro146
Schrenkiellaparvula*LOST
Isatistinctoria_Pro5
Myagrumperfoliatum_Pro10
Thlaspiarvense*LOST
Iberisamara_Pro23
Boecherastricta_Pro12
Arabidopsisthaliana_Pro43
Malcolmiamaritima*LOST
Capsellagrandiflora*LOST
Lepidiumsativum_Pro19
Diptychocarpusstrictus_Pro54
Lunariaannua_Pro29
Cakilemaritima_Pro9
Brassicarapa_Pro91
Sinapisalba_Pro142
Schrenkiellaparvula*LOST
Isatistinctoria_Pro87
Myagrumperfoliatum_Pro9
Thlaspiarvense*LOST
Iberisamara_Pro82
Boecherastricta_Pro37
Arabidopsisthaliana_Pro40
Malcolmiamaritima*LOST
Capsellagrandiflora*LOST
Lepidiumsativum_Pro69
Diptychocarpusstrictus_Pro52
Lunariaannua_Pro27
Cakilemaritima_Pro6
Brassicarapa_Pro80
Sinapisalba_Pro136
Schrenkiellaparvula*LOST
Isatistinctoria_Pro59
Myagrumperfoliatum_Pro26
Thlaspiarvense*LOST
Iberisamara_Pro59
Boecherastricta_Pro58
Malcolmiamaritima_Pro50
Arabidopsisthaliana_Pro39
Capsellagrandiflora*LOST
Lepidiumsativum_Pro70
Diptychocarpusstrictus_Pro51
Lunariaannua_Pro24
Cakilemaritima_Pro62
Brassicarapa_Pro63
Sinapisalba_Pro128
Schrenkiellaparvula*LOST
Isatistinctoria_Pro34
Myagrumperfoliatum_Pro20
Thlaspiarvense*LOST
Iberisamara_Pro64
Boecherastricta_Pro55
Malcolmiamaritima_Pro8

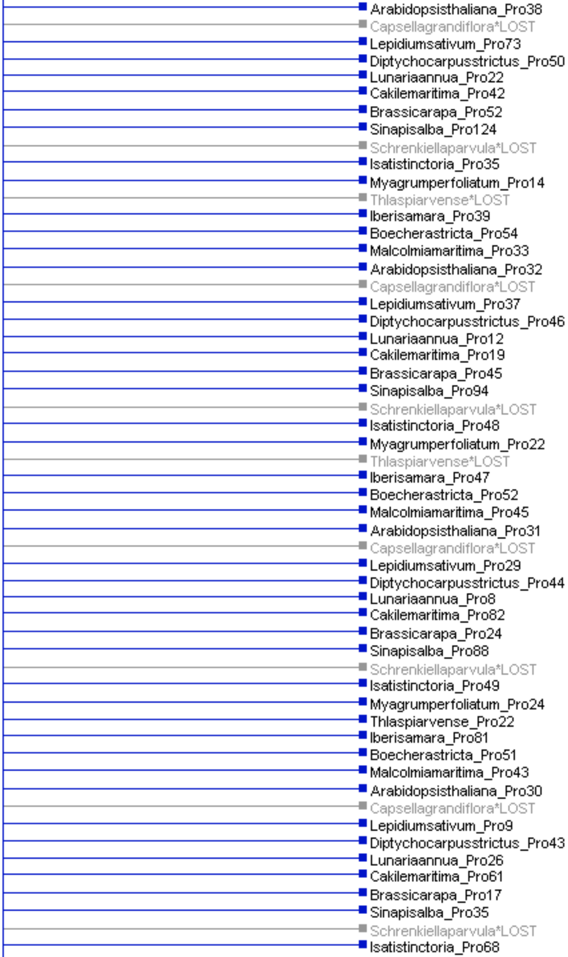
Arabidopsisthaliana_Pro38
Capsellagrandiflora*LOST
Lepidiumsativum_Pro73
Diptychocarpusstrictus_Pro50
Lunariaannua_Pro22
Cakilemaritima_Pro42
Brassicarapa_Pro52
Sinapisalba_Pro124
Schrenkiellaparvula*LOST
Isatistinctoria_Pro35
Myagrumperfoliatum_Pro14
Thlaspiarvense*LOST
Iberisamara_Pro39
Boecherastricta_Pro54
Malcolmiamaritima_Pro33
Arabidopsisthaliana_Pro32
Capsellagrandiflora*LOST
Lepidiumsativum_Pro37
Diptychocarpusstrictus_Pro46
Lunariaannua_Pro12
Cakilemaritima_Pro19
Brassicarapa_Pro45
Sinapisalba_Pro94
Schrenkiellaparvula*LOST
Isatistinctoria_Pro48
Myagrumperfoliatum_Pro22
Thlaspiarvense*LOST
Iberisamara_Pro47
Boecherastricta_Pro52
Malcolmiamaritima_Pro45
Arabidopsisthaliana_Pro31
Capsellagrandiflora*LOST
Lepidiumsativum_Pro29
Diptychocarpusstrictus_Pro44
Lunariaannua_Pro8
Cakilemaritima_Pro82
Brassicarapa_Pro24
Sinapisalba_Pro88
Schrenkiellaparvula*LOST
Isatistinctoria_Pro49
Myagrumperfoliatum_Pro24
Thlaspiarvense_Pro22
Iberisamara_Pro81
Boecherastricta_Pro51
Malcolmiamaritima_Pro43
Arabidopsisthaliana_Pro30
Capsellagrandiflora*LOST
Lepidiumsativum_Pro9
Diptychocarpusstrictus_Pro43
Lunariaannua_Pro26
Cakilemaritima_Pro61
Brassicarapa_Pro17
Sinapisalba_Pro35
Schrenkiellaparvula*LOST
Isatistinctoria_Pro68

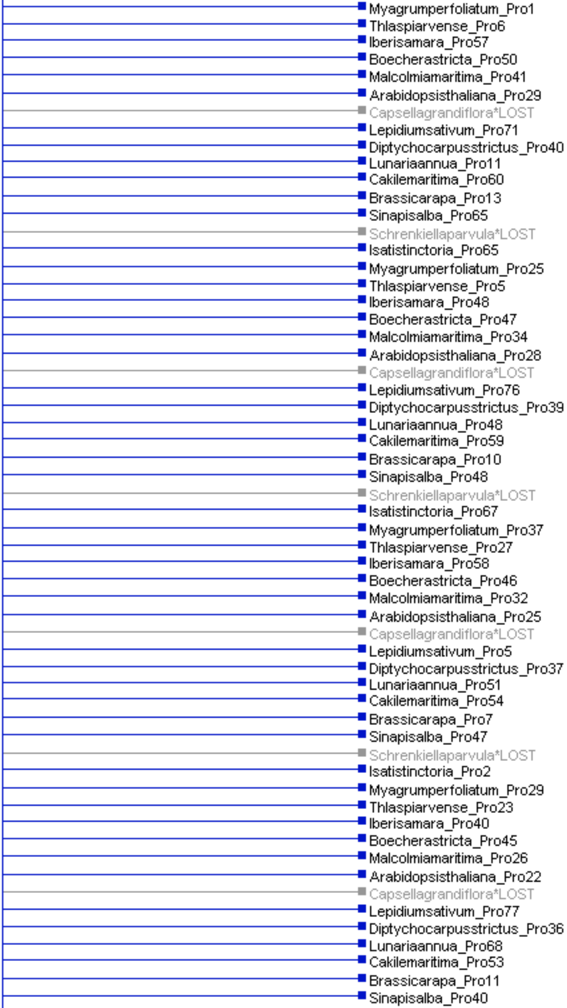

Myagrumperfoliatum_Pro1
Thlaspiarvense_Pro6
Iberisamara_Pro57
Boecherastricta_Pro50
Malcolmiamaritima_Pro41
Arabidopsisthaliana_Pro29
Capsellagrandiflora*LOST
Lepidiumsativum_Pro71
Diptychocarpusstrictus_Pro40
Lunariaannua_Pro11
Cakilemaritima_Pro60
Brassicarapa_Pro13
Sinapisalba_Pro65
Schrenkiellaparvula*LOST
Isatistinctoria_Pro65
Myagrumperfoliatum_Pro25
Thlaspiarvense_Pro5
Iberisamara_Pro48
Boecherastricta_Pro47
Malcolmiamaritima_Pro34
Arabidopsisthaliana_Pro28
Capsellagrandiflora*LOST
Lepidiumsativum_Pro76
Diptychocarpusstrictus_Pro39
Lunariaannua_Pro48
Cakilemaritima_Pro59
Brassicarapa_Pro10
Sinapisalba_Pro48
Schrenkiellaparvula*LOST
Isatistinctoria_Pro67
Myagrumperfoliatum_Pro37
Thlaspiarvense_Pro27
Iberisamara_Pro58
Boecherastricta_Pro46
Malcolmiamaritima_Pro32
Arabidopsisthaliana_Pro25
Capsellagrandiflora*LOST
Lepidiumsativum_Pro5
Diptychocarpusstrictus_Pro37
Lunariaannua_Pro51
Cakilemaritima_Pro54
Brassicarapa_Pro7
Sinapisalba_Pro47
Schrenkiellaparvula*LOST
Isatistinctoria_Pro2
Myagrumperfoliatum_Pro29
Thlaspiarvense_Pro23
Iberisamara_Pro40
Boecherastricta_Pro45
Malcolmiamaritima_Pro26
Arabidopsisthaliana_Pro22
Capsellagrandiflora*LOST
Lepidiumsativum_Pro77
Diptychocarpusstrictus_Pro36
Lunariaannua_Pro68
Cakilemaritima_Pro53
Brassicarapa_Pro11
Sinapisalba_Pro40

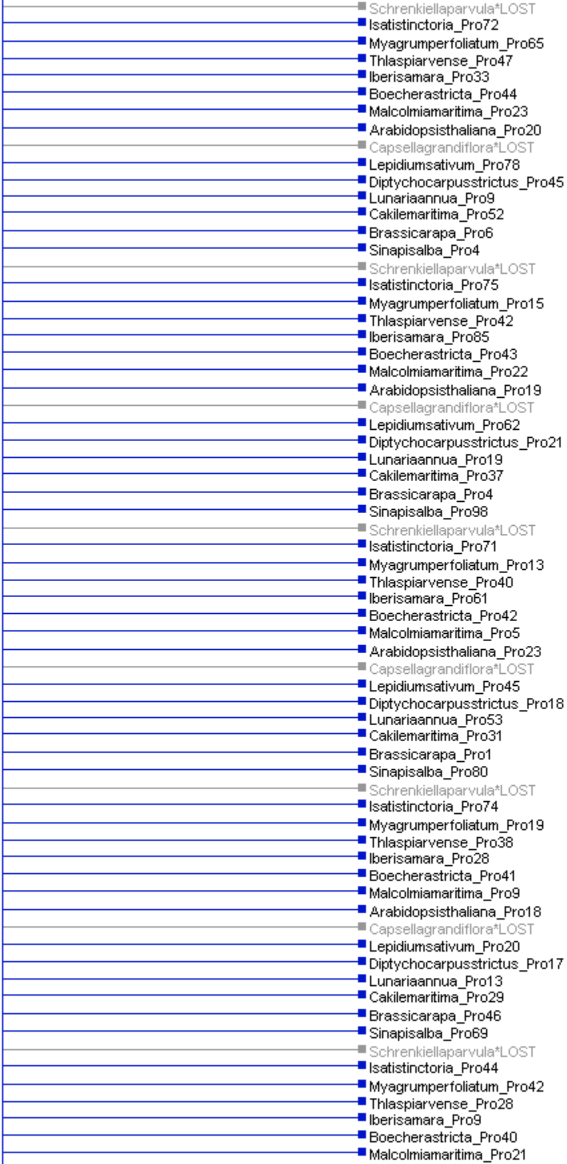

Schrenkiellaparvula*LOST
Isatistinctoria_Pro72
Myagrumperfoliatum_Pro65
Thlaspiarvense_Pro47
Iberisamara_Pro33
Boecherastricta_Pro44
Malcolmiamaritima_Pro23
Arabidopsisthaliana_Pro20
Capsellagrandiflora*LOST
Lepidiumsativum_Pro78
Diptychocarpusstrictus_Pro45
Lunariaannua_Pro9
Cakilemaritima_Pro52
Brassicarapa_Pro6
Sinapisalba_Pro4
Schrenkiellaparvula*LOST
Isatistinctoria_Pro75
Myagrumperfoliatum_Pro15
Thlaspiarvense_Pro42
Iberisamara_Pro85
Boecherastricta_Pro43
Malcolmiamaritima_Pro22
Arabidopsisthaliana_Pro19
Capsellagrandiflora*LOST
Lepidiumsativum_Pro62
Diptychocarpusstrictus_Pro21
Lunariaannua_Pro19
Cakilemaritima_Pro37
Brassicarapa_Pro4
Sinapisalba_Pro98
Schrenkiellaparvula*LOST
Isatistinctoria_Pro71
Myagrumperfoliatum_Pro13
Thlaspiarvense_Pro40
Iberisamara_Pro61
Boecherastricta_Pro42
Malcolmiamaritima_Pro5
Arabidopsisthaliana_Pro23
Capsellagrandiflora*LOST
Lepidiumsativum_Pro45
Diptychocarpusstrictus_Pro18
Lunariaannua_Pro53
Cakilemaritima_Pro31
Brassicarapa_Pro1
Sinapisalba_Pro80
Schrenkiellaparvula*LOST
Isatistinctoria_Pro74
Myagrumperfoliatum_Pro19
Thlaspiarvense_Pro38
Iberisamara_Pro28
Boecherastricta_Pro41
Malcolmiamaritima_Pro9
Arabidopsisthaliana_Pro18
Capsellagrandiflora*LOST
Lepidiumsativum_Pro20
Diptychocarpusstrictus_Pro17
Lunariaannua_Pro13
Cakilemaritima_Pro29
Brassicarapa_Pro46
Sinapisalba_Pro69
Schrenkiellaparvula*LOST
Isatistinctoria_Pro44
Myagrumperfoliatum_Pro42
Thlaspiarvense_Pro28
Iberisamara_Pro9
Boecherastricta_Pro40
Malcolmiamaritima_Pro21

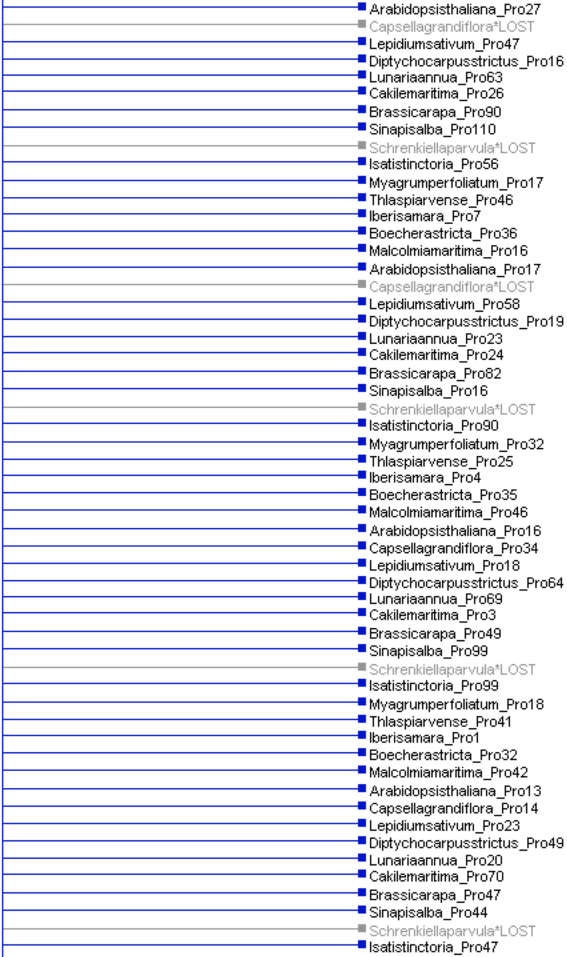
Arabidopsisthaliana_Pro27
Capsellagrandiflora*LOST
Lepidiumsativum_Pro47
Diptychocarpusstrictus_Pro16
Lunariaannua_Pro63
Cakilemaritima_Pro26
Brassicarapa_Pro90
Sinapisalba_Pro110
Schrenkiellaparvula*LOST
Isatistinctoria_Pro56
Myagrumperfoliatum_Pro17
Thlaspiarvense_Pro46
Iberisamara_Pro7
Boecherastricta_Pro36
Malcolmiamaritima_Pro16
Arabidopsisthaliana_Pro17
Capsellagrandiflora*LOST
Lepidiumsativum_Pro58
Diptychocarpusstrictus_Pro19
Lunariaannua_Pro23
Cakilemaritima_Pro24
Brassicarapa_Pro82
Sinapisalba_Pro16
Schrenkiellaparvula*LOST
Isatistinctoria_Pro90
Myagrumperfoliatum_Pro32
Thlaspiarvense_Pro25
Iberisamara_Pro4
Boecherastricta_Pro35
Malcolmiamaritima_Pro46
Arabidopsisthaliana_Pro16
Capsellagrandiflora_Pro34
Lepidiumsativum_Pro18
Diptychocarpusstrictus_Pro64
Lunariaannua_Pro69
Cakilemaritima_Pro3
Brassicarapa_Pro49
Sinapisalba_Pro99
Schrenkiellaparvula*LOST
Isatistinctoria_Pro99
Myagrumperfoliatum_Pro18
Thlaspiarvense_Pro41
Iberisamara_Pro1
Boecherastricta_Pro32
Malcolmiamaritima_Pro42
Arabidopsisthaliana_Pro13
Capsellagrandiflora_Pro14
Lepidiumsativum_Pro23
Diptychocarpusstrictus_Pro49
Lunariaannua_Pro20
Cakilemaritima_Pro70
Brassicarapa_Pro47
Sinapisalba_Pro44
Schrenkiellaparvula*LOST
Isatistinctoria_Pro47

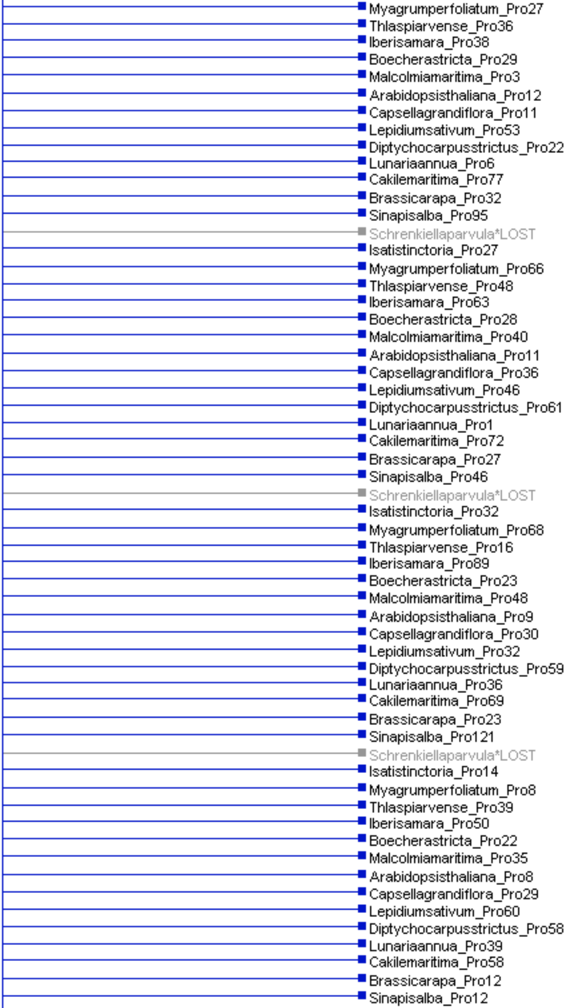
Myagrumperfoliatum_Pro27
Thlaspiarvense_Pro36
Iberisamara_Pro38
Boecherastricta_Pro29
Malcolmiamaritima_Pro3
Arabidopsisthaliana_Pro12
Capsellagrandiflora_Pro11
Lepidiumsativum_Pro53
Diptychocarpusstrictus_Pro22
Lunariaannua_Pro6
Cakilemaritima_Pro77
Brassicarapa_Pro32
Sinapisalba_Pro95
Schrenkiellaparvula*LOST
Isatistinctoria_Pro27
Myagrumperfoliatum_Pro66
Thlaspiarvense_Pro48
Iberisamara_Pro63
Boecherastricta_Pro28
Malcolmiamaritima_Pro40
Arabidopsisthaliana_Pro11
Capsellagrandiflora_Pro36
Lepidiumsativum_Pro46
Diptychocarpusstrictus_Pro61
Lunariaannua_Pro1
Cakilemaritima_Pro72
Brassicarapa_Pro27
Sinapisalba_Pro46
Schrenkiellaparvula*LOST
Isatistinctoria_Pro32
Myagrumperfoliatum_Pro68
Thlaspiarvense_Pro16
Iberisamara_Pro89
Boecherastricta_Pro23
Malcolmiamaritima_Pro48
Arabidopsisthaliana_Pro9
Capsellagrandiflora_Pro30
Lepidiumsativum_Pro32
Diptychocarpusstrictus_Pro59
Lunariaannua_Pro36
Cakilemaritima_Pro69
Brassicarapa_Pro23
Sinapisalba_Pro121
Schrenkiellaparvula*LOST
Isatistinctoria_Pro14
Myagrumperfoliatum_Pro8
Thlaspiarvense_Pro39
Iberisamara_Pro50
Boecherastricta_Pro22
Malcolmiamaritima_Pro35
Arabidopsisthaliana_Pro8
Capsellagrandiflora_Pro29
Lepidiumsativum_Pro60
Diptychocarpusstrictus_Pro58
Lunariaannua_Pro39
Cakilemaritima_Pro58
Brassicarapa_Pro12
Sinapisalba_Pro12

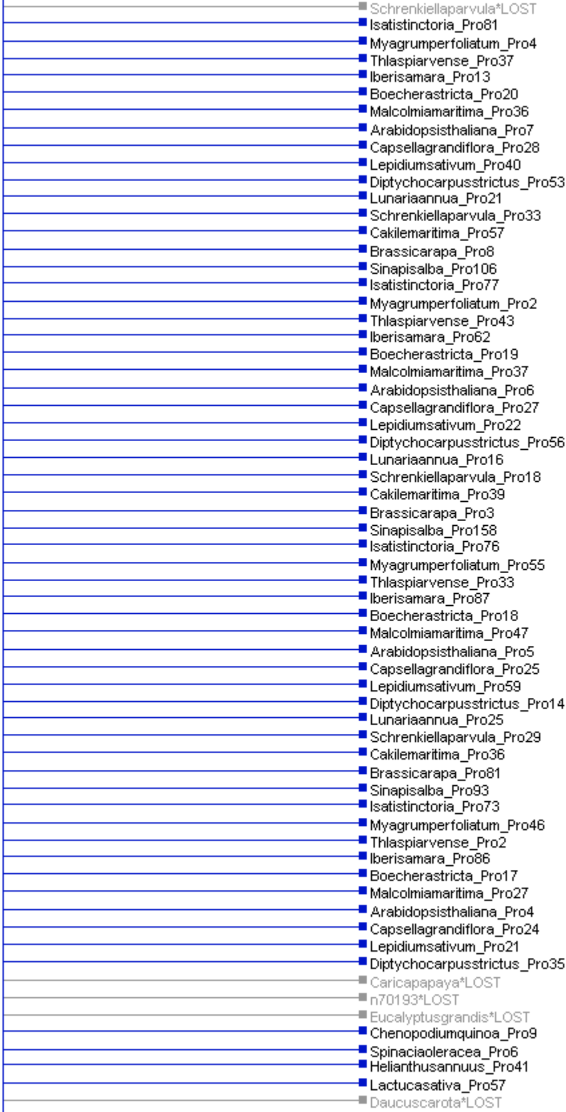
Schrenkiellaparvula*LOST
Isatistinctoria_Pro81
Myagrumperfoliatum_Pro4
Thlaspiarvense_Pro37
Iberisamara_Pro13
Boecherastricta_Pro20
Malcolmiamaritima_Pro36
Arabidopsisthaliana_Pro7
Capsellagrandiflora_Pro28
Lepidiumsativum_Pro40
Diptychocarpusstrictus_Pro53
Lunariaannua_Pro21
Schrenkiellaparvula_Pro33
Cakilemaritima_Pro57
Brassicarapa_Pro8
Sinapisalba_Pro106
Isatistinctoria_Pro77
Myagrumperfoliatum_Pro2
Thlaspiarvense_Pro43
Iberisamara_Pro62
Boecherastricta_Pro19
Malcolmiamaritima_Pro37
Arabidopsisthaliana_Pro6
Capsellagrandiflora_Pro27
Lepidiumsativum_Pro22
Diptychocarpusstrictus_Pro56
Lunariaannua_Pro16
Schrenkiellaparvula_Pro18
Cakilemaritima_Pro39
Brassicarapa_Pro3
Sinapisalba_Pro158
Isatistinctoria_Pro76
Myagrumperfoliatum_Pro55
Thlaspiarvense_Pro33
Iberisamara_Pro87
Boecherastricta_Pro18
Malcolmiamaritima_Pro47
Arabidopsisthaliana_Pro5
Capsellagrandiflora_Pro25
Lepidiumsativum_Pro59
Diptychocarpusstrictus_Pro14
Lunariaannua_Pro25
Schrenkiellaparvula_Pro29
Cakilemaritima_Pro36
Brassicarapa_Pro81
Sinapisalba_Pro93
Isatistinctoria_Pro73
Myagrumperfoliatum_Pro46
Thlaspiarvense_Pro2
Iberisamara_Pro86
Boecherastricta_Pro17
Malcolmiamaritima_Pro27
Arabidopsisthaliana_Pro4
Capsellagrandiflora_Pro24
Lepidiumsativum_Pro21
Diptychocarpusstrictus_Pro35
Caricapapaya*LOST
n70193*LOST
Eucalyptusgrandis*LOST
Chenopodiumquinoa_Pro9
Spinaciaoleracea_Pro6
Helianthusannuus_Pro41
Lactucasativa_Pro57
Daucuscarota*LOST

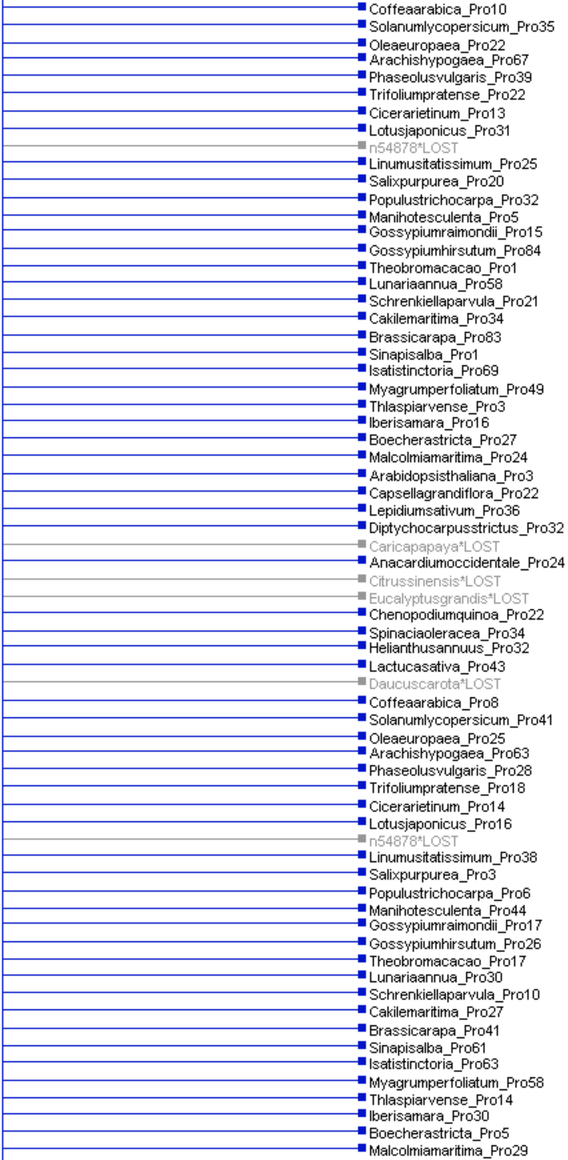
Coffeaarabica_Pro10
Solanumlycopersicum_Pro35
Oleaeuropaea_Pro22
Arachishypogaea_Pro67
Phaseolusvulgaris_Pro39
Trifoliumpratense_Pro22
Cicerarietinum_Pro13
Lotusjaponicus_Pro31
n54878*LOST
Linumusitatissimum_Pro25
Salixpurpurea_Pro20
Populustrichocarpa_Pro32
Manihotesculenta_Pro5
Gossypiumraimondii_Pro15
Gossypiumhirsutum_Pro84
Theobromacacao_Pro1
Lunariaannua_Pro58
Schrenkiellaparvula_Pro21
Cakilemaritima_Pro34
Brassicarapa_Pro83
Sinapisalba_Pro1
Isatistinctoria_Pro69
Myagrumperfoliatum_Pro49
Thlaspiarvense_Pro3
Iberisamara_Pro16
Boecherastricta_Pro27
Malcolmiamaritima_Pro24
Arabidopsisthaliana_Pro3
Capsellagrandiflora_Pro22
Lepidiumsativum_Pro36
Diptychocarpusstrictus_Pro32
Caricapapaya*LOST
Anacardiumoccidentale_Pro24
Citrussinensis*LOST
Eucalyptusgrandis*LOST
Chenopodiumquinoa_Pro22
Spinaciaoleracea_Pro34
Helianthusannuus_Pro32
Lactucasativa_Pro43
Daucuscarota*LOST
Coffeaarabica_Pro8
Solanumlycopersicum_Pro41
Oleaeuropaea_Pro25
Arachishypogaea_Pro63
Phaseolusvulgaris_Pro28
Trifoliumpratense_Pro18
Cicerarietinum_Pro14
Lotusjaponicus_Pro16
n54878*LOST
Linumusitatissimum_Pro38
Salixpurpurea_Pro3
Populustrichocarpa_Pro6
Manihotesculenta_Pro44
Gossypiumraimondii_Pro17
Gossypiumhirsutum_Pro26
Theobromacacao_Pro17
Lunariaannua_Pro30
Schrenkiellaparvula_Pro10
Cakilemaritima_Pro27
Brassicarapa_Pro41
Sinapisalba_Pro61
Isatistinctoria_Pro63
Myagrumperfoliatum_Pro58
Thlaspiarvense_Pro14
Iberisamara_Pro30
Boecherastricta_Pro5
Malcolmiamaritima_Pro29

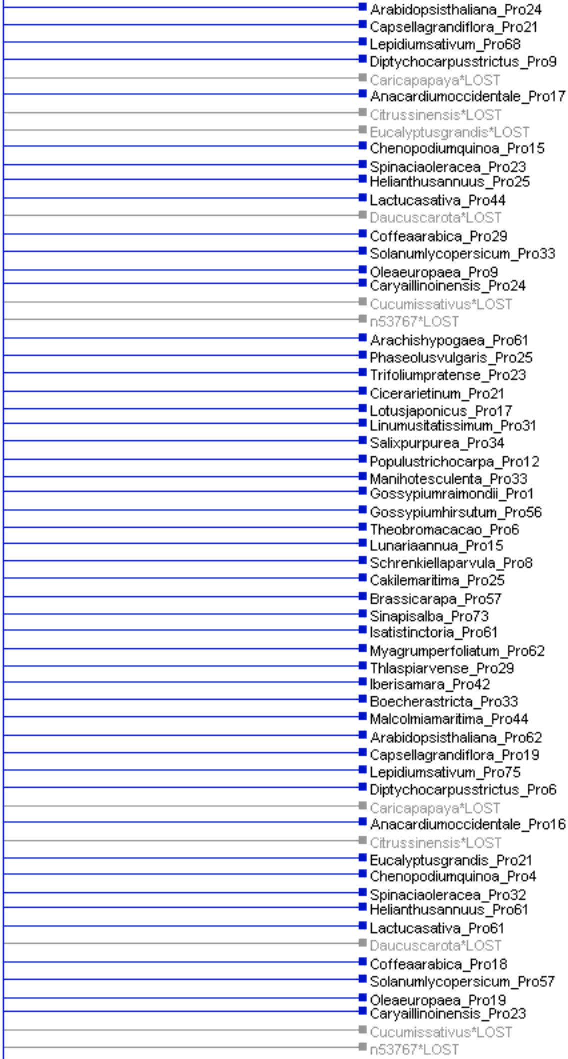
Arabidopsisthaliana_Pro24
Capsellagrandiflora_Pro21
Lepidiumsativum_Pro68
Diptychocarpusstrictus_Pro9
Caricapapaya*LOST
Anacardiumoccidentale_Pro17
Citrussinensis*LOST
Eucalyptusgrandis*LOST
Chenopodiumquinoa_Pro15
Spinaciaoleracea_Pro23
Helianthusannuus_Pro25
Lactucasativa_Pro44
Daucuscarota*LOST
Coffeaarabica_Pro29
Solanumlycopersicum_Pro33
Oleaeuropaea_Pro9
Caryaillinoinensis_Pro24
Cucumissativus*LOST
n53767*LOST
Arachishypogaea_Pro61
Phaseolusvulgaris_Pro25
Trifoliumpratense_Pro23
Cicerarietinum_Pro21
Lotusjaponicus_Pro17
Linumusitatissimum_Pro31
Salixpurpurea_Pro34
Populustrichocarpa_Pro12
Manihotesculenta_Pro33
Gossypiumraimondii_Pro1
Gossypiumhirsutum_Pro56
Theobromacacao_Pro6
Lunariaannua_Pro15
Schrenkiellaparvula_Pro8
Cakilemaritima_Pro25
Brassicarapa_Pro57
Sinapisalba_Pro73
Isatistinctoria_Pro61
Myagrumperfoliatum_Pro62
Thlaspiarvense_Pro29
Iberisamara_Pro42
Boecherastricta_Pro33
Malcolmiamaritima_Pro44
Arabidopsisthaliana_Pro62
Capsellagrandiflora_Pro19
Lepidiumsativum_Pro75
Diptychocarpusstrictus_Pro6
Caricapapaya*LOST
Anacardiumoccidentale_Pro16
Citrussinensis*LOST
Eucalyptusgrandis_Pro21
Chenopodiumquinoa_Pro4
Spinaciaoleracea_Pro32
Helianthusannuus_Pro61
Lactucasativa_Pro61
Daucuscarota*LOST
Coffeaarabica_Pro18
Solanumlycopersicum_Pro57
Oleaeuropaea_Pro19
Caryaillinoinensis_Pro23
Cucumissativus*LOST
n53767*LOST

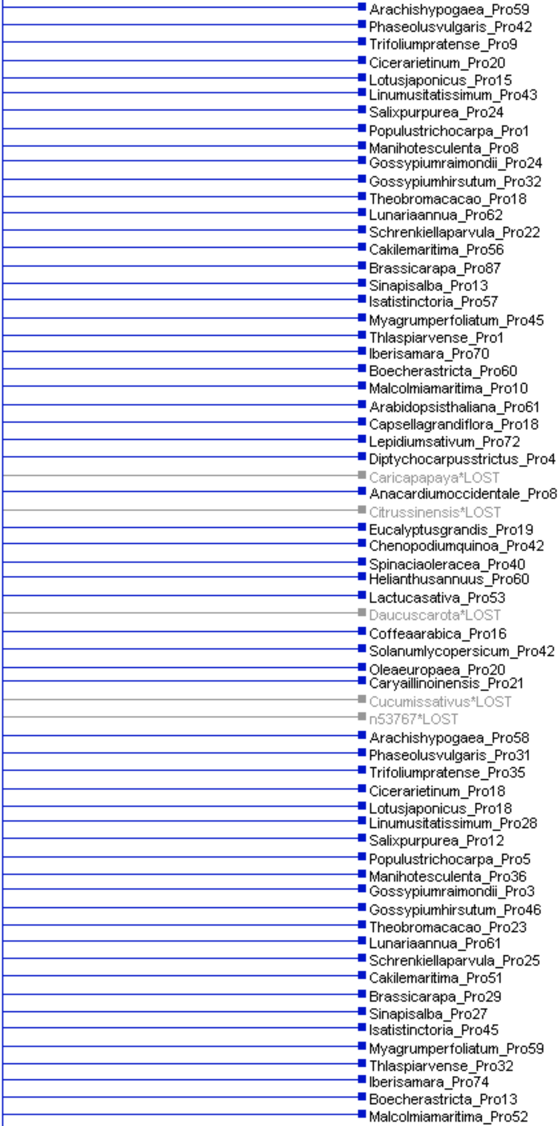
Arachishypogaea_Pro59
Phaseolusvulgaris_Pro42
Trifoliumpratense_Pro9
Cicerarietinum_Pro20
Lotusjaponicus_Pro15
Linumusitatissimum_Pro43
Salixpurpurea_Pro24
Populustrichocarpa_Pro1
Manihotesculenta_Pro8
Gossypiumraimondii_Pro24
Gossypiumhirsutum_Pro32
Theobromacacao_Pro18
Lunariaannua_Pro62
Schrenkiellaparvula_Pro22
Cakilemaritima_Pro56
Brassicarapa_Pro87
Sinapisalba_Pro13
Isatistinctoria_Pro57
Myagrumperfoliatum_Pro45
Thlaspiarvense_Pro1
Iberisamara_Pro70
Boecherastricta_Pro60
Malcolmiamaritima_Pro10
Arabidopsisthaliana_Pro61
Capsellagrandiflora_Pro18
Lepidiumsativum_Pro72
Diptychocarpusstrictus_Pro4
Caricapapaya*LOST
Anacardiumoccidentale_Pro8
Citrussinensis*LOST
Eucalyptusgrandis_Pro19
Chenopodiumquinoa_Pro42
Spinaciaoleracea_Pro40
Helianthusannuus_Pro60
Lactucasativa_Pro53
Daucuscarota*LOST
Coffeaarabica_Pro16
Solanumlycopersicum_Pro42
Oleaeuropaea_Pro20
Caryaillinoinensis_Pro21
Cucumissativus*LOST
n53767*LOST
Arachishypogaea_Pro58
Phaseolusvulgaris_Pro31
Trifoliumpratense_Pro35
Cicerarietinum_Pro18
Lotusjaponicus_Pro18
Linumusitatissimum_Pro28
Salixpurpurea_Pro12
Populustrichocarpa_Pro5
Manihotesculenta_Pro36
Gossypiumraimondii_Pro3
Gossypiumhirsutum_Pro46
Theobromacacao_Pro23
Lunariaannua_Pro61
Schrenkiellaparvula_Pro25
Cakilemaritima_Pro51
Brassicarapa_Pro29
Sinapisalba_Pro27
Isatistinctoria_Pro45
Myagrumperfoliatum_Pro59
Thlaspiarvense_Pro32
Iberisamara_Pro74
Boecherastricta_Pro13
Malcolmiamaritima_Pro52

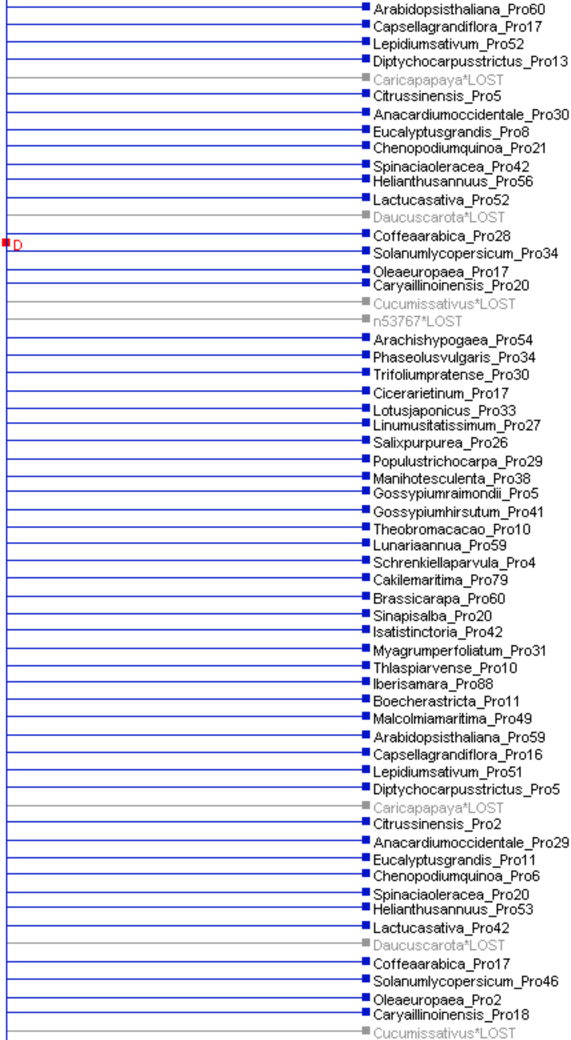
Arabidopsisthaliana_Pro60
Capsellagrandiflora_Pro17
Lepidiumsativum_Pro52
Diptychocarpusstrictus_Pro13
Caricapapaya*LOST
Citrussinensis_Pro5
Anacardiumoccidentale_Pro30
Eucalyptusgrandis_Pro8
Chenopodiumquinoa_Pro21
Spinaciaoleracea_Pro42
Helianthusannuus_Pro56
Lactucasativa_Pro52
Daucuscarota*LOST
Coffeaarabica_Pro28
D
Solanumlycopersicum_Pro34
Oleaeuropaea_Pro17
Caryaillinoinensis_Pro20
Cucumissativus*LOST
n53767*LOST
Arachishypogaea_Pro54
Phaseolusvulgaris_Pro34
Trifoliumpratense_Pro30
Cicerarietinum_Pro17
Lotusjaponicus_Pro33
Linumusitatissimum_Pro27
Salixpurpurea_Pro26
Populustrichocarpa_Pro29
Manihotesculenta_Pro38
Gossypiumraimondii_Pro5
Gossypiumhirsutum_Pro41
Theobromacacao_Pro10
Lunariaannua_Pro59
Schrenkiellaparvula_Pro4
Cakilemaritima_Pro79
Brassicarapa_Pro60
Sinapisalba_Pro20
Isatistinctoria_Pro42
Myagrumperfoliatum_Pro31
Thlaspiarvense_Pro10
Iberisamara_Pro88
Boecherastricta_Pro11
Malcolmiamaritima_Pro49
Arabidopsisthaliana_Pro59
Capsellagrandiflora_Pro16
Lepidiumsativum_Pro51
Diptychocarpusstrictus_Pro5
Caricapapaya*LOST
Citrussinensis_Pro2
Anacardiumoccidentale_Pro29
Eucalyptusgrandis_Pro11
Chenopodiumquinoa_Pro6
Spinaciaoleracea_Pro20
Helianthusannuus_Pro53
Lactucasativa_Pro42
Daucuscarota*LOST
Coffeaarabica_Pro17
Solanumlycopersicum_Pro46
Oleaeuropaea_Pro2
Caryaillinoinensis_Pro18
Cucumissativus*LOST

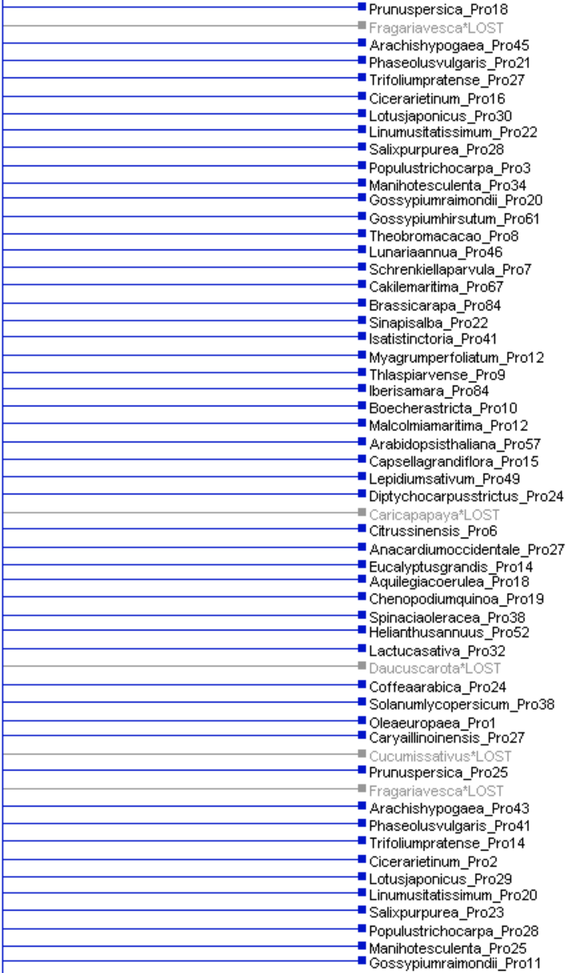
Prunuspersica_Pro18
Fragariavesca*LOST
Arachishypogaea_Pro45
Phaseolusvulgaris_Pro21
Trifoliumpratense_Pro27
Cicerarietinum_Pro16
Lotusjaponicus_Pro30
Linumusitatissimum_Pro22
Salixpurpurea_Pro28
Populustrichocarpa_Pro3
Manihotesculenta_Pro34
Gossypiumraimondii_Pro20
Gossypiumhirsutum_Pro61
Theobromacacao_Pro8
Lunariaannua_Pro46
Schrenkiellaparvula_Pro7
Cakilemaritima_Pro67
Brassicarapa_Pro84
Sinapisalba_Pro22
Isatistinctoria_Pro41
Myagrumperfoliatum_Pro12
Thlaspiarvense_Pro9
Iberisamara_Pro84
Boecherastricta_Pro10
Malcolmiamaritima_Pro12
Arabidopsisthaliana_Pro57
Capsellagrandiflora_Pro15
Lepidiumsativum_Pro49
Diptychocarpusstrictus_Pro24
Caricapapaya*LOST
Citrussinensis_Pro6
Anacardiumoccidentale_Pro27
Eucalyptusgrandis_Pro14
Aquilegiacoerulea_Pro18
Chenopodiumquinoa_Pro19
Spinaciaoleracea_Pro38
Helianthusannuus_Pro52
Lactucasativa_Pro32
Daucuscarota*LOST
Coffeaarabica_Pro24
Solanumlycopersicum_Pro38
Oleaeuropaea_Pro1
Caryaillinoinensis_Pro27
Cucumissativus*LOST
Prunuspersica_Pro25
Fragariavesca*LOST
Arachishypogaea_Pro43
Phaseolusvulgaris_Pro41
Trifoliumpratense_Pro14
Cicerarietinum_Pro2
Lotusjaponicus_Pro29
Linumusitatissimum_Pro20
Salixpurpurea_Pro23
Populustrichocarpa_Pro28
Manihotesculenta_Pro25
Gossypiumraimondii_Pro11

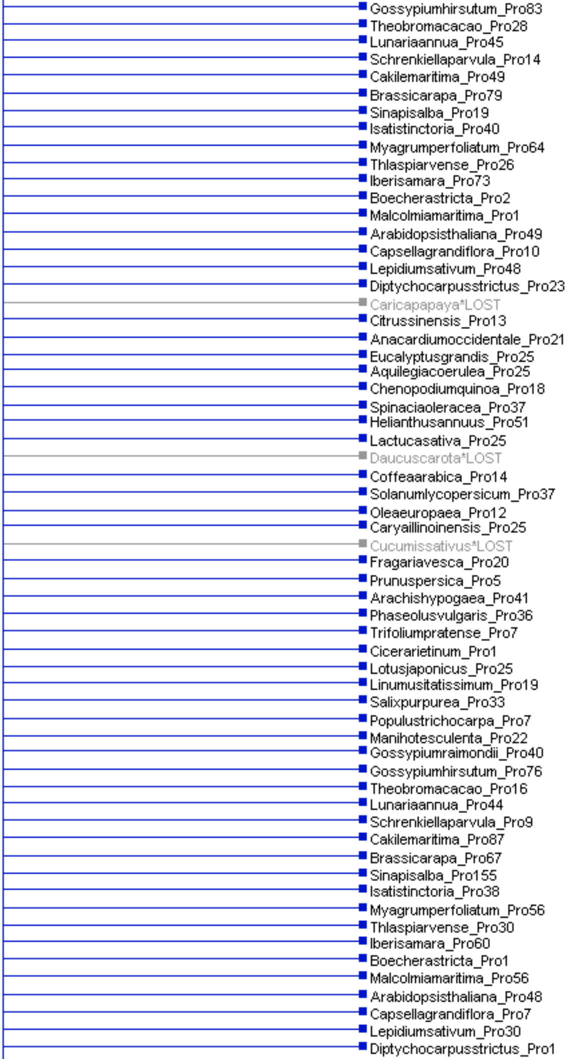
Gossypiumhirsutum_Pro83
Theobromacacao_Pro28
Lunariaannua_Pro45
Schrenkiellaparvula_Pro14
Cakilemaritima_Pro49
Brassicarapa_Pro79
Sinapisalba_Pro19
Isatistinctoria_Pro40
Myagrumperfoliatum_Pro64
Thlaspiarvense_Pro26
Iberisamara_Pro73
Boecherastricta_Pro2
Malcolmiamaritima_Pro1
Arabidopsisthaliana_Pro49
Capsellagrandiflora_Pro10
Lepidiumsativum_Pro48
Diptychocarpusstrictus_Pro23
Caricapapaya*LOST
Citrussinensis_Pro13
Anacardiumoccidentale_Pro21
Eucalyptusgrandis_Pro25
Aquilegiacoerulea_Pro25
Chenopodiumquinoa_Pro18
Spinaciaoleracea_Pro37
Helianthusannuus_Pro51
Lactucasativa_Pro25
Daucuscarota*LOST
Coffeaarabica_Pro14
Solanumlycopersicum_Pro37
Oleaeuropaea_Pro12
Caryaillinoinensis_Pro25
Cucumissativus*LOST
Fragariavesca_Pro20
Prunuspersica_Pro5
Arachishypogaea_Pro41
Phaseolusvulgaris_Pro36
Trifoliumpratense_Pro7
Cicerarietinum_Pro1
Lotusjaponicus_Pro25
Linumusitatissimum_Pro19
Salixpurpurea_Pro33
Populustrichocarpa_Pro7
Manihotesculenta_Pro22
Gossypiumraimondii_Pro40
Gossypiumhirsutum_Pro76
Theobromacacao_Pro16
Lunariaannua_Pro44
Schrenkiellaparvula_Pro9
Cakilemaritima_Pro87
Brassicarapa_Pro67
Sinapisalba_Pro155
Isatistinctoria_Pro38
Myagrumperfoliatum_Pro56
Thlaspiarvense_Pro30
Iberisamara_Pro60
Boecherastricta_Pro1
Malcolmiamaritima_Pro56
Arabidopsisthaliana_Pro48
Capsellagrandiflora_Pro7
Lepidiumsativum_Pro30
Diptychocarpusstrictus_Pro1

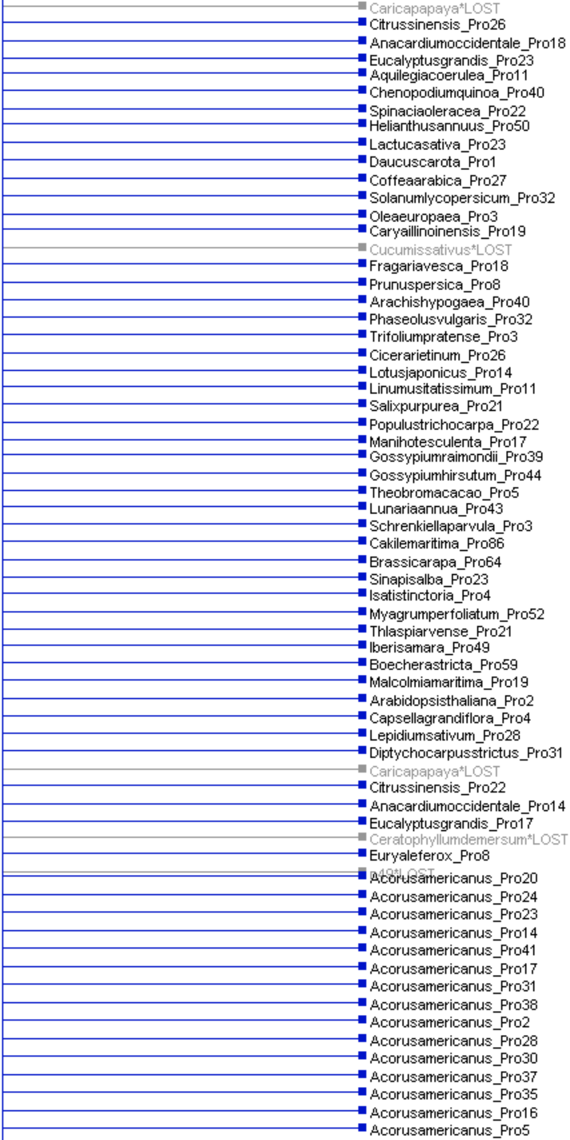
Caricapapaya*LOST
Citrussinensis_Pro26
Anacardiumoccidentale_Pro18
Eucalyptusgrandis_Pro23
Aquilegiacoerulea_Pro11
Chenopodiumquinoa_Pro40
Spinaciaoleracea_Pro22
Helianthusannuus_Pro50
Lactucasativa_Pro23
Daucuscarota_Pro1
Coffeaarabica_Pro27
Solanumlycopersicum_Pro32
Oleaeuropaea_Pro3
Caryaillinoinensis_Pro19
Cucumissativus*LOST
Fragariavesca_Pro18
Prunuspersica_Pro8
Arachishypogaea_Pro40
Phaseolusvulgaris_Pro32
Trifoliumpratense_Pro3
Cicerarietinum_Pro26
Lotusjaponicus_Pro14
Linumusitatissimum_Pro11
Salixpurpurea_Pro21
Populustrichocarpa_Pro22
Manihotesculenta_Pro17
Gossypiumraimondii_Pro39
Gossypiumhirsutum_Pro44
Theobromacacao_Pro5
Lunariaannua_Pro43
Schrenkiellaparvula_Pro3
Cakilemaritima_Pro86
Brassicarapa_Pro64
Sinapisalba_Pro23
Isatistinctoria_Pro4
Myagrumperfoliatum_Pro52
Thlaspiarvense_Pro21
Iberisamara_Pro49
Boecherastricta_Pro59
Malcolmiamaritima_Pro19
Arabidopsisthaliana_Pro2
Capsellagrandiflora_Pro4
Lepidiumsativum_Pro28
Diptychocarpusstrictus_Pro31
Caricapapaya*LOST
Citrussinensis_Pro22
Anacardiumoccidentale_Pro14
Eucalyptusgrandis_Pro17
Ceratophyllumdemersum*LOST
Euryaleferox_Pro8
Acorusamericanus_Pro20
Acorusamericanus_Pro24
Acorusamericanus_Pro23
Acorusamericanus_Pro14
Acorusamericanus_Pro41
Acorusamericanus_Pro17
Acorusamericanus_Pro31
Acorusamericanus_Pro38
Acorusamericanus_Pro2
Acorusamericanus_Pro28
Acorusamericanus_Pro30
Acorusamericanus_Pro37
Acorusamericanus_Pro35
Acorusamericanus_Pro16
Acorusamericanus_Pro5

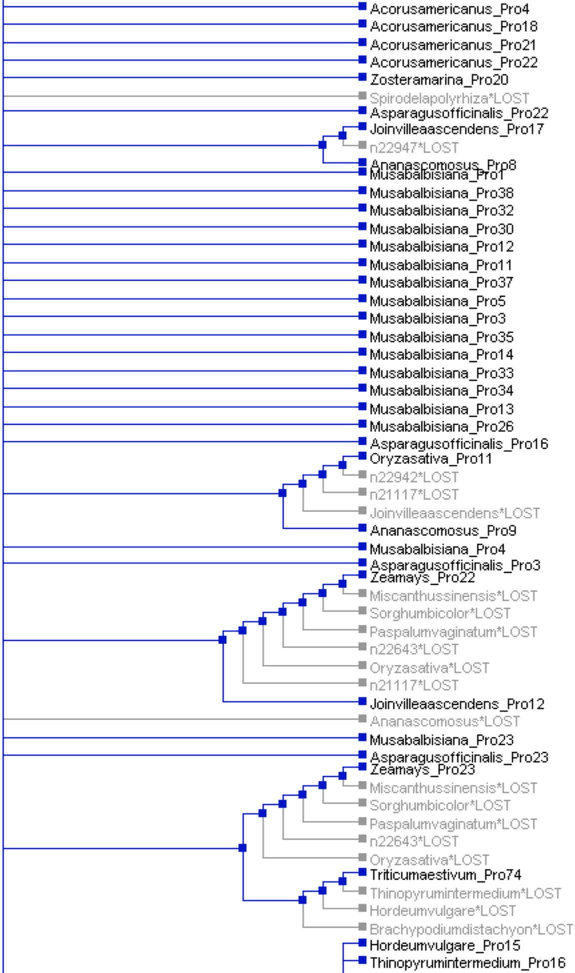
Acorusamericanus_Pro4
Acorusamericanus_Pro18
Acorusamericanus_Pro21
Acorusamericanus_Pro22
Zosteramarina_Pro20
Spirodelapolyrhiza*LOST
Asparagusofficinalis_Pro22
Joinvilleaascendens_Pro17
n22947*LOST
Ananascomosus_Pro8
Musabalbisiana_Pro1
Musabalbisiana_Pro38
Musabalbisiana_Pro32
Musabalbisiana_Pro30
Musabalbisiana_Pro12
Musabalbisiana_Pro11
Musabalbisiana_Pro37
Musabalbisiana_Pro5
Musabalbisiana_Pro3
Musabalbisiana_Pro35
Musabalbisiana_Pro14
Musabalbisiana_Pro33
Musabalbisiana_Pro34
Musabalbisiana_Pro13
Musabalbisiana_Pro26
Asparagusofficinalis_Pro16
Oryzasativa_Pro11
n22942*LOST
n21117*LOST
Joinvilleaascendens*LOST
Ananascomosus_Pro9
Musabalbisiana_Pro4
Asparagusofficinalis_Pro3
Zeamays_Pro22
Miscanthussinensis*LOST
Sorghumbicolor*LOST
Paspalumvaginatum*LOST
n22643*LOST
Oryzasativa*LOST
n21117*LOST
Joinvilleaascendens_Pro12
Ananascomosus*LOST
Musabalbisiana_Pro23
Asparagusofficinalis_Pro23
Zeamays_Pro23
Miscanthussinensis*LOST
Sorghumbicolor*LOST
Paspalumvaginatum*LOST
n22643*LOST
Oryzasativa*LOST
Triticumaestivum_Pro74
Thinopyrumintermedium*LOST
Hordeumvulgare*LOST
Brachypodiumdistachyon*LOST
Hordeumvulgare_Pro15
Thinopyrumintermedium_Pro16

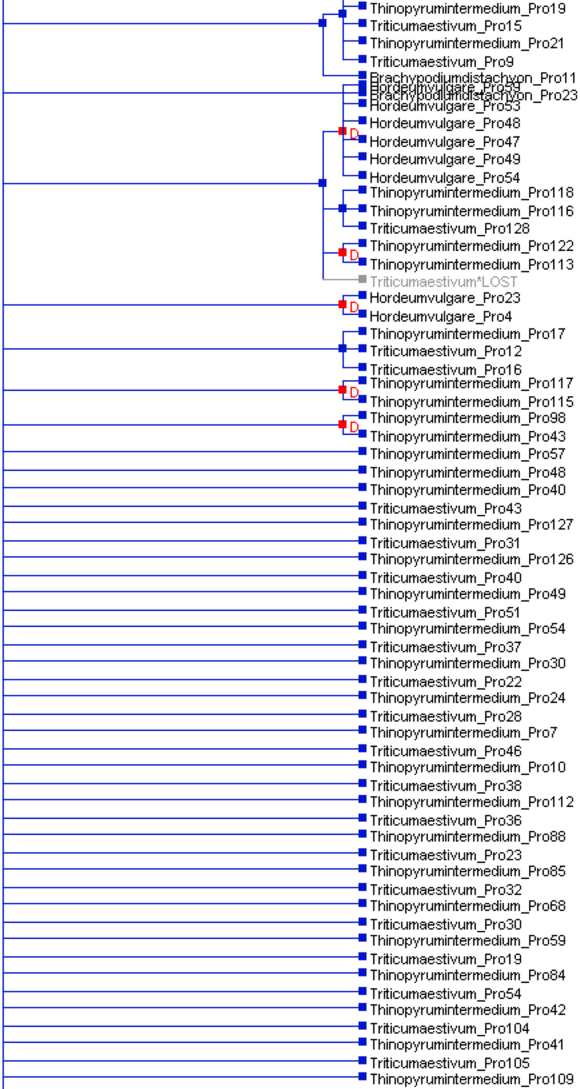
Thinopyrumintermedium_Pro19
Triticumaestivum_Pro15
Thinopyrumintermedium_Pro21
Triticumaestivum_Pro9
Brachypodiumdistachyon_Pro11
Hordeumvulgare_Pro59
Brachypodiumdistachyon_Pro23
Hordeumvulgare_Pro53
Hordeumvulgare_Pro48
D
Hordeumvulgare_Pro47
Hordeumvulgare_Pro49
Hordeumvulgare_Pro54
Thinopyrumintermedium_Pro118
Thinopyrumintermedium_Pro116
Triticumaestivum_Pro128
Thinopyrumintermedium_Pro122
D
Thinopyrumintermedium_Pro113
Triticumaestivum*LOST
Hordeumvulgare_Pro23
D
Hordeumvulgare_Pro4
Thinopyrumintermedium_Pro17
Triticumaestivum_Pro12
Triticumaestivum_Pro16
Thinopyrumintermedium_Pro117
D
Thinopyrumintermedium_Pro115
Thinopyrumintermedium_Pro98
D
Thinopyrumintermedium_Pro43
Thinopyrumintermedium_Pro57
Thinopyrumintermedium_Pro48
Thinopyrumintermedium_Pro40
Triticumaestivum_Pro43
Thinopyrumintermedium_Pro127
Triticumaestivum_Pro31
Thinopyrumintermedium_Pro126
Triticumaestivum_Pro40
Thinopyrumintermedium_Pro49
Triticumaestivum_Pro51
Thinopyrumintermedium_Pro54
Triticumaestivum_Pro37
Thinopyrumintermedium_Pro30
Triticumaestivum_Pro22
Thinopyrumintermedium_Pro24
Triticumaestivum_Pro28
Thinopyrumintermedium_Pro7
Triticumaestivum_Pro46
Thinopyrumintermedium_Pro10
Triticumaestivum_Pro38
Thinopyrumintermedium_Pro112
Triticumaestivum_Pro36
Thinopyrumintermedium_Pro88
Triticumaestivum_Pro23
Thinopyrumintermedium_Pro85
Triticumaestivum_Pro32
Thinopyrumintermedium_Pro68
Triticumaestivum_Pro30
Thinopyrumintermedium_Pro59
Triticumaestivum_Pro19
Thinopyrumintermedium_Pro84
Triticumaestivum_Pro54
Thinopyrumintermedium_Pro42
Triticumaestivum_Pro104
Thinopyrumintermedium_Pro41
Triticumaestivum_Pro105
Thinopyrumintermedium_Pro109

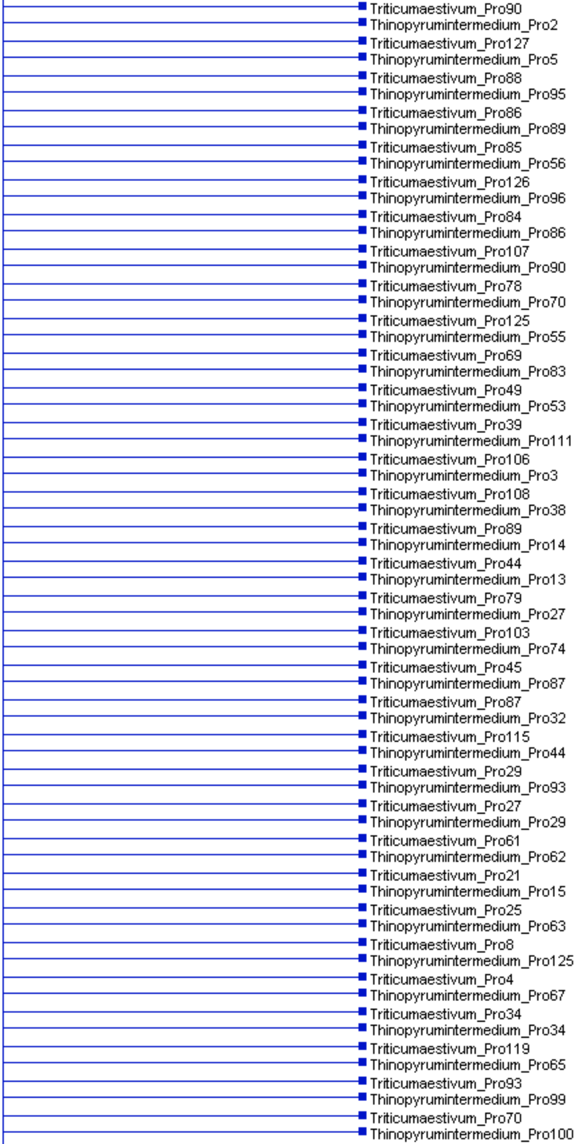
Triticumaestivum_Pro90
Thinopyrumintermedium_Pro2
Triticumaestivum_Pro127
Thinopyrumintermedium_Pro5
Triticumaestivum_Pro88
Thinopyrumintermedium_Pro95
Triticumaestivum_Pro86
Thinopyrumintermedium_Pro89
Triticumaestivum_Pro85
Thinopyrumintermedium_Pro56
Triticumaestivum_Pro126
Thinopyrumintermedium_Pro96
Triticumaestivum_Pro84
Thinopyrumintermedium_Pro86
Triticumaestivum_Pro107
Thinopyrumintermedium_Pro90
Triticumaestivum_Pro78
Thinopyrumintermedium_Pro70
Triticumaestivum_Pro125
Thinopyrumintermedium_Pro55
Triticumaestivum_Pro69
Thinopyrumintermedium_Pro83
Triticumaestivum_Pro49
Thinopyrumintermedium_Pro53
Triticumaestivum_Pro39
Thinopyrumintermedium_Pro111
Triticumaestivum_Pro106
Thinopyrumintermedium_Pro3
Triticumaestivum_Pro108
Thinopyrumintermedium_Pro38
Triticumaestivum_Pro89
Thinopyrumintermedium_Pro14
Triticumaestivum_Pro44
Thinopyrumintermedium_Pro13
Triticumaestivum_Pro79
Thinopyrumintermedium_Pro27
Triticumaestivum_Pro103
Thinopyrumintermedium_Pro74
Triticumaestivum_Pro45
Thinopyrumintermedium_Pro87
Triticumaestivum_Pro87
Thinopyrumintermedium_Pro32
Triticumaestivum_Pro115
Thinopyrumintermedium_Pro44
Triticumaestivum_Pro29
Thinopyrumintermedium_Pro93
Triticumaestivum_Pro27
Thinopyrumintermedium_Pro29
Triticumaestivum_Pro61
Thinopyrumintermedium_Pro62
Triticumaestivum_Pro21
Thinopyrumintermedium_Pro15
Triticumaestivum_Pro25
Thinopyrumintermedium_Pro63
Triticumaestivum_Pro8
Thinopyrumintermedium_Pro125
Triticumaestivum_Pro4
Thinopyrumintermedium_Pro67
Triticumaestivum_Pro34
Thinopyrumintermedium_Pro34
Triticumaestivum_Pro119
Thinopyrumintermedium_Pro65
Triticumaestivum_Pro93
Thinopyrumintermedium_Pro99
Triticumaestivum_Pro70
Thinopyrumintermedium_Pro100

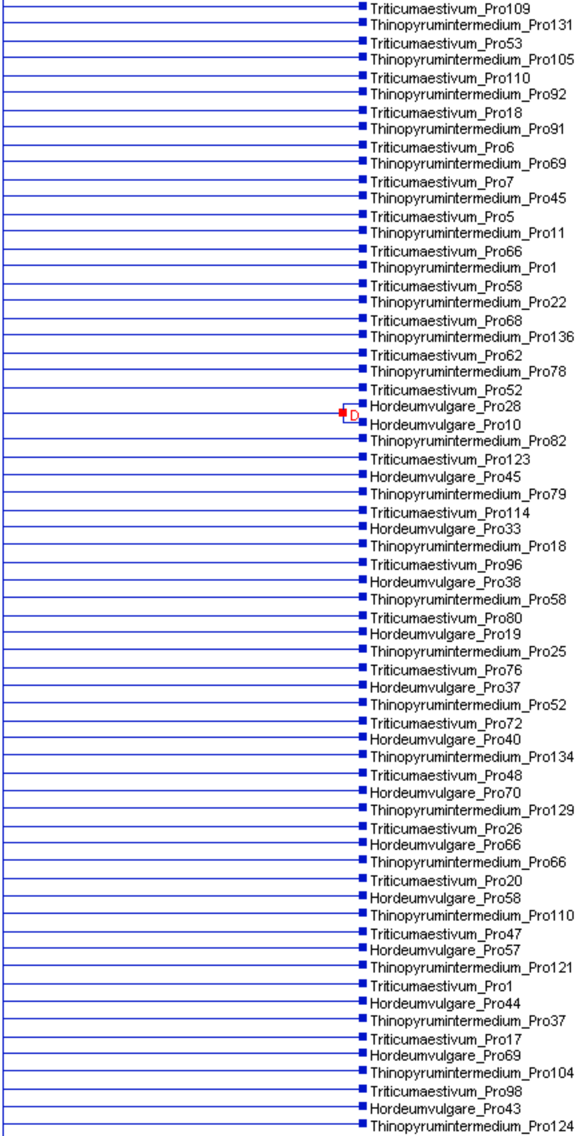

Triticumaestivum_Pro109
Thinopyrumintermedium_Pro131
Triticumaestivum_Pro53
Thinopyrumintermedium_Pro105
Triticumaestivum_Pro110
Thinopyrumintermedium_Pro92
Triticumaestivum_Pro18
Thinopyrumintermedium_Pro91
Triticumaestivum_Pro6
Thinopyrumintermedium_Pro69
Triticumaestivum_Pro7
Thinopyrumintermedium_Pro45
Triticumaestivum_Pro5
Thinopyrumintermedium_Pro11
Triticumaestivum_Pro66
Thinopyrumintermedium_Pro1
Triticumaestivum_Pro58
Thinopyrumintermedium_Pro22
Triticumaestivum_Pro68
Thinopyrumintermedium_Pro136
Triticumaestivum_Pro62
Thinopyrumintermedium_Pro78
Triticumaestivum_Pro52
Hordeumvulgare_Pro28
D
Hordeumvulgare_Pro10
Thinopyrumintermedium_Pro82
Triticumaestivum_Pro123
Hordeumvulgare_Pro45
Thinopyrumintermedium_Pro79
Triticumaestivum_Pro114
Hordeumvulgare_Pro33
Thinopyrumintermedium_Pro18
Triticumaestivum_Pro96
Hordeumvulgare_Pro38
Thinopyrumintermedium_Pro58
Triticumaestivum_Pro80
Hordeumvulgare_Pro19
Thinopyrumintermedium_Pro25
Triticumaestivum_Pro76
Hordeumvulgare_Pro37
Thinopyrumintermedium_Pro52
Triticumaestivum_Pro72
Hordeumvulgare_Pro40
Thinopyrumintermedium_Pro134
Triticumaestivum_Pro48
Hordeumvulgare_Pro70
Thinopyrumintermedium_Pro129
Triticumaestivum_Pro26
Hordeumvulgare_Pro66
Thinopyrumintermedium_Pro66
Triticumaestivum_Pro20
Hordeumvulgare_Pro58
Thinopyrumintermedium_Pro110
Triticumaestivum_Pro47
Hordeumvulgare_Pro57
Thinopyrumintermedium_Pro121
Triticumaestivum_Pro1
Hordeumvulgare_Pro44
Thinopyrumintermedium_Pro37
Triticumaestivum_Pro17
Hordeumvulgare_Pro69
Thinopyrumintermedium_Pro104
Triticumaestivum_Pro98
Hordeumvulgare_Pro43
Thinopyrumintermedium_Pro124

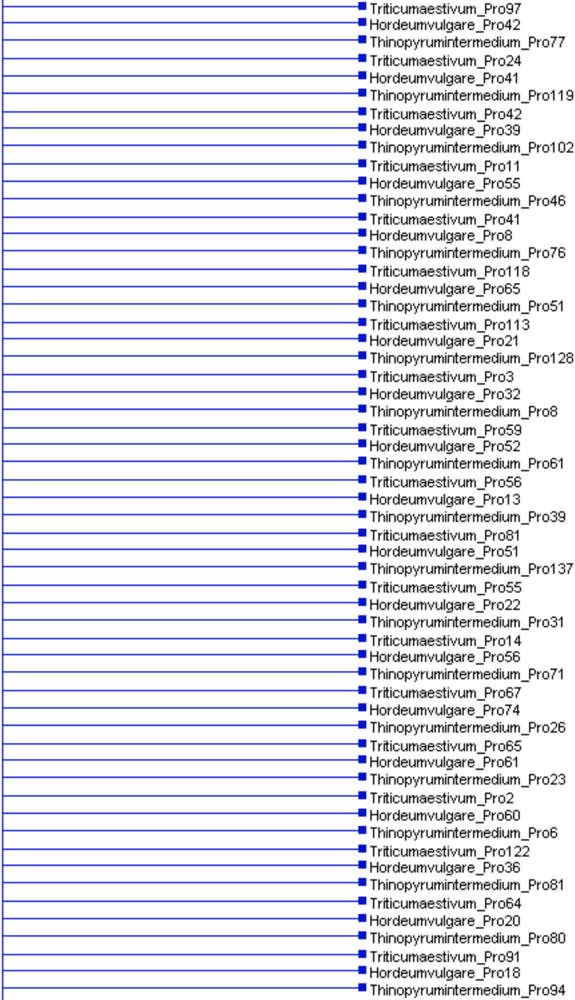
Triticumaestivum_Pro97
Hordeumvulgare_Pro42
Thinopyrumintermedium_Pro77
Triticumaestivum_Pro24
Hordeumvulgare_Pro41
Thinopyrumintermedium_Pro119
Triticumaestivum_Pro42
Hordeumvulgare_Pro39
Thinopyrumintermedium_Pro102
Triticumaestivum_Pro11
Hordeumvulgare_Pro55
Thinopyrumintermedium_Pro46
Triticumaestivum_Pro41
Hordeumvulgare_Pro8
Thinopyrumintermedium_Pro76
Triticumaestivum_Pro118
Hordeumvulgare_Pro65
Thinopyrumintermedium_Pro51
Triticumaestivum_Pro113
Hordeumvulgare_Pro21
Thinopyrumintermedium_Pro128
Triticumaestivum_Pro3
Hordeumvulgare_Pro32
Thinopyrumintermedium_Pro8
Triticumaestivum_Pro59
Hordeumvulgare_Pro52
Thinopyrumintermedium_Pro61
Triticumaestivum_Pro56
Hordeumvulgare_Pro13
Thinopyrumintermedium_Pro39
Triticumaestivum_Pro81
Hordeumvulgare_Pro51
Thinopyrumintermedium_Pro137
Triticumaestivum_Pro55
Hordeumvulgare_Pro22
Thinopyrumintermedium_Pro31
Triticumaestivum_Pro14
Hordeumvulgare_Pro56
Thinopyrumintermedium_Pro71
Triticumaestivum_Pro67
Hordeumvulgare_Pro74
Thinopyrumintermedium_Pro26
Triticumaestivum_Pro65
Hordeumvulgare_Pro61
Thinopyrumintermedium_Pro23
Triticumaestivum_Pro2
Hordeumvulgare_Pro60
Thinopyrumintermedium_Pro6
Triticumaestivum_Pro122
Hordeumvulgare_Pro36
Thinopyrumintermedium_Pro81
Triticumaestivum_Pro64
Hordeumvulgare_Pro20
Thinopyrumintermedium_Pro80
Triticumaestivum_Pro91
Hordeumvulgare_Pro18
Thinopyrumintermedium_Pro94

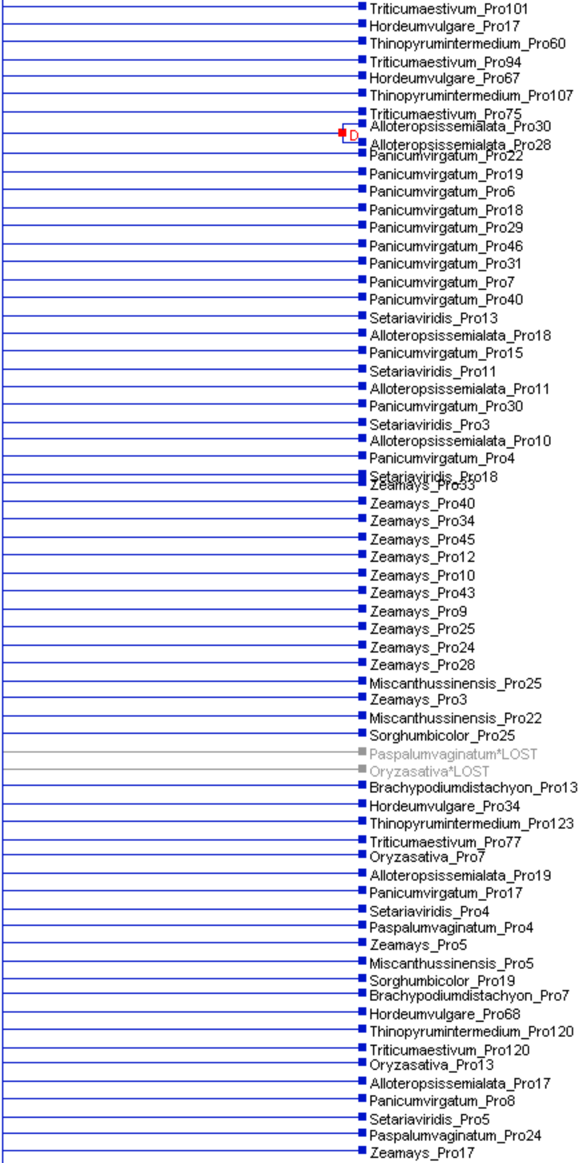
Triticumaestivum_Pro101
Hordeumvulgare_Pro17
Thinopyrumintermedium_Pro60
Triticumaestivum_Pro94
Hordeumvulgare_Pro67
Thinopyrumintermedium_Pro107
Triticumaestivum_Pro75
Alloteropsissemialata_Pro30
D
Alloteropsissemialata_Pro28
Panicumvirgatum_Pro22
Panicumvirgatum_Pro19
Panicumvirgatum_Pro6
Panicumvirgatum_Pro18
Panicumvirgatum_Pro29
Panicumvirgatum_Pro46
Panicumvirgatum_Pro31
Panicumvirgatum_Pro7
Panicumvirgatum_Pro40
Setariaviridis_Pro13
Alloteropsissemialata_Pro18
Panicumvirgatum_Pro15
Setariaviridis_Pro11
Alloteropsissemialata_Pro11
Panicumvirgatum_Pro30
Setariaviridis_Pro3
Alloteropsissemialata_Pro10
Panicumvirgatum_Pro4
Setariaviridis_Pro18
Zeamays_Pro33
Zeamays_Pro40
Zeamays_Pro34
Zeamays_Pro45
Zeamays_Pro12
Zeamays_Pro10
Zeamays_Pro43
Zeamays_Pro9
Zeamays_Pro25
Zeamays_Pro24
Zeamays_Pro28
Miscanthussinensis_Pro25
Zeamays_Pro3
Miscanthussinensis_Pro22
Sorghumbicolor_Pro25
Paspalumvaginatum*LOST
Oryzasativa*LOST
Brachypodiumdistachyon_Pro13
Hordeumvulgare_Pro34
Thinopyrumintermedium_Pro123
Triticumaestivum_Pro77
Oryzasativa_Pro7
Alloteropsissemialata_Pro19
Panicumvirgatum_Pro17
Setariaviridis_Pro4
Paspalumvaginatum_Pro4
Zeamays_Pro5
Miscanthussinensis_Pro5
Sorghumbicolor_Pro19
Brachypodiumdistachyon_Pro7
Hordeumvulgare_Pro68
Thinopyrumintermedium_Pro120
Triticumaestivum_Pro120
Oryzasativa_Pro13
Alloteropsissemialata_Pro17
Panicumvirgatum_Pro8
Setariaviridis_Pro5
Paspalumvaginatum_Pro24
Zeamays_Pro17

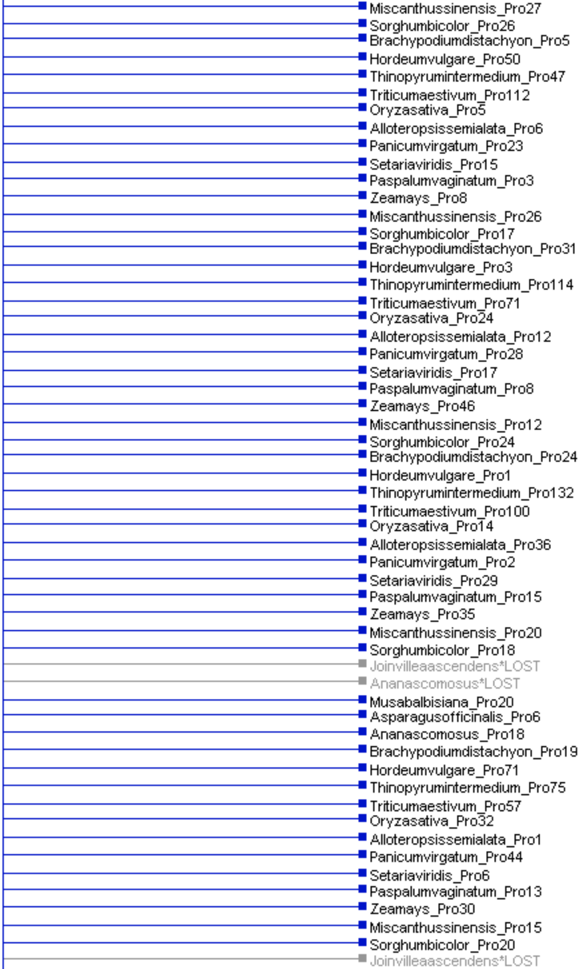
Miscanthussinensis_Pro27
Sorghumbicolor_Pro26
Brachypodiumdistachyon_Pro5
Hordeumvulgare_Pro50
Thinopyrumintermedium_Pro47
Triticumaestivum_Pro112
Oryzasativa_Pro5
Alloteropsissemialata_Pro6
Panicumvirgatum_Pro23
Setariaviridis_Pro15
Paspalumvaginatum_Pro3
Zeamays_Pro8
Miscanthussinensis_Pro26
Sorghumbicolor_Pro17
Brachypodiumdistachyon_Pro31
Hordeumvulgare_Pro3
Thinopyrumintermedium_Pro114
Triticumaestivum_Pro71
Oryzasativa_Pro24
Alloteropsissemialata_Pro12
Panicumvirgatum_Pro28
Setariaviridis_Pro17
Paspalumvaginatum_Pro8
Zeamays_Pro46
Miscanthussinensis_Pro12
Sorghumbicolor_Pro24
Brachypodiumdistachyon_Pro24
Hordeumvulgare_Pro1
Thinopyrumintermedium_Pro132
Triticumaestivum_Pro100
Oryzasativa_Pro14
Alloteropsissemialata_Pro36
Panicumvirgatum_Pro2
Setariaviridis_Pro29
Paspalumvaginatum_Pro15
Zeamays_Pro35
Miscanthussinensis_Pro20
Sorghumbicolor_Pro18
Joinvilleaascendens*LOST
Ananascomosus*LOST
Musabalbisiana_Pro20
Asparagusofficinalis_Pro6
Ananascomosus_Pro18
Brachypodiumdistachyon_Pro19
Hordeumvulgare_Pro71
Thinopyrumintermedium_Pro75
Triticumaestivum_Pro57
Oryzasativa_Pro32
Alloteropsissemialata_Pro1
Panicumvirgatum_Pro44
Setariaviridis_Pro6
Paspalumvaginatum_Pro13
Zeamays_Pro30
Miscanthussinensis_Pro15
Sorghumbicolor_Pro20
Joinvilleaascendens*LOST

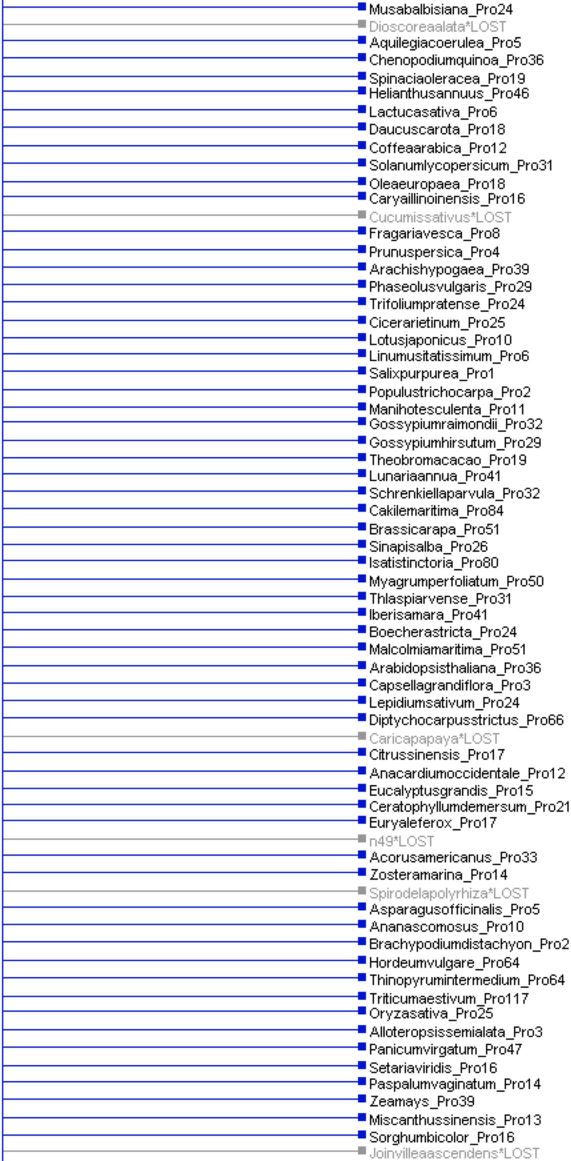
Musabalbisiana_Pro24
Dioscoreaalata*LOST
Aquilegiacoerulea_Pro5
Chenopodiumquinoa_Pro36
Spinaciaoleracea_Pro19
Helianthusannuus_Pro46
Lactucasativa_Pro6
Daucuscarota_Pro18
Coffeaarabica_Pro12
Solanumlycopersicum_Pro31
Oleaeuropaea_Pro18
Caryaillinoinensis_Pro16
Cucumissativus*LOST
Fragariavesca_Pro8
Prunuspersica_Pro4
Arachishypogaea_Pro39
Phaseolusvulgaris_Pro29
Trifoliumpratense_Pro24
Cicerarietinum_Pro25
Lotusjaponicus_Pro10
Linumusitatissimum_Pro6
Salixpurpurea_Pro1
Populustrichocarpa_Pro2
Manihotesculenta_Pro11
Gossypiumraimondii_Pro32
Gossypiumhirsutum_Pro29
Theobromacacao_Pro19
Lunariaannua_Pro41
Schrenkiellaparvula_Pro32
Cakilemaritima_Pro84
Brassicarapa_Pro51
Sinapisalba_Pro26
Isatistinctoria_Pro80
Myagrumperfoliatum_Pro50
Thlaspiarvense_Pro31
Iberisamara_Pro41
Boecherastricta_Pro24
Malcolmiamaritima_Pro51
Arabidopsisthaliana_Pro36
Capsellagrandiflora_Pro3
Lepidiumsativum_Pro24
Diptychocarpusstrictus_Pro66
Caricapapaya*LOST
Citrussinensis_Pro17
Anacardiumoccidentale_Pro12
Eucalyptusgrandis_Pro15
Ceratophyllumdemersum_Pro21
Euryaleferox_Pro17
n49*LOST
Acorusamericanus_Pro33
Zosteramarina_Pro14
Spirodelapolyrhiza*LOST
Asparagusofficinalis_Pro5
Ananascomosus_Pro10
Brachypodiumdistachyon_Pro2
Hordeumvulgare_Pro64
Thinopyrumintermedium_Pro64
Triticumaestivum_Pro117
Oryzasativa_Pro25
Alloteropsissemialata_Pro3
Panicumvirgatum_Pro47
Setariaviridis_Pro16
Paspalumvaginatum_Pro14
Zeamays_Pro39
Miscanthussinensis_Pro13
Sorghumbicolor_Pro16
Joinvilleaascendens*LOST

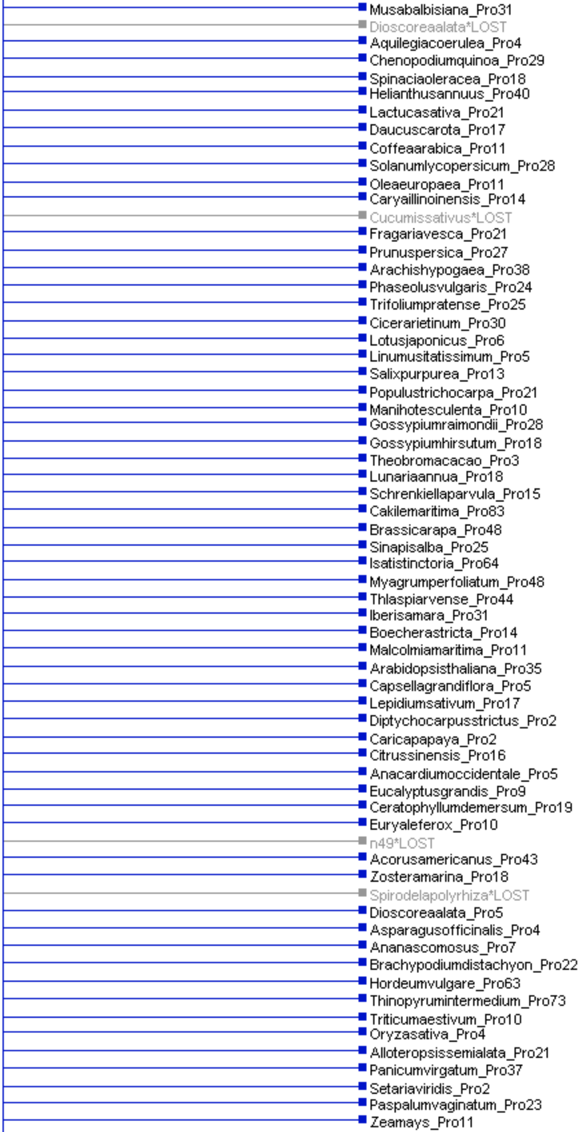
Musabalbisiana_Pro31
Dioscoreaalata*LOST
Aquilegiacoerulea_Pro4
Chenopodiumquinoa_Pro29
Spinaciaoleracea_Pro18
Helianthusannuus_Pro40
Lactucasativa_Pro21
Daucuscarota_Pro17
Coffeaarabica_Pro11
Solanumlycopersicum_Pro28
Oleaeuropaea_Pro11
Caryaillinoinensis_Pro14
Cucumissativus*LOST
Fragariavesca_Pro21
Prunuspersica_Pro27
Arachishypogaea_Pro38
Phaseolusvulgaris_Pro24
Trifoliumpratense_Pro25
Cicerarietinum_Pro30
Lotusjaponicus_Pro6
Linumusitatissimum_Pro5
Salixpurpurea_Pro13
Populustrichocarpa_Pro21
Manihotesculenta_Pro10
Gossypiumraimondii_Pro28
Gossypiumhirsutum_Pro18
Theobromacacao_Pro3
Lunariaannua_Pro18
Schrenkiellaparvula_Pro15
Cakilemaritima_Pro83
Brassicarapa_Pro48
Sinapisalba_Pro25
Isatistinctoria_Pro64
Myagrumperfoliatum_Pro48
Thlaspiarvense_Pro44
Iberisamara_Pro31
Boecherastricta_Pro14
Malcolmiamaritima_Pro11
Arabidopsisthaliana_Pro35
Capsellagrandiflora_Pro5
Lepidiumsativum_Pro17
Diptychocarpusstrictus_Pro2
Caricapapaya_Pro2
Citrussinensis_Pro16
Anacardiumoccidentale_Pro5
Eucalyptusgrandis_Pro9
Ceratophyllumdemersum_Pro19
Euryaleferox_Pro10
n49*LOST
Acorusamericanus_Pro43
Zosteramarina_Pro18
Spirodelapolyrhiza*LOST
Dioscoreaalata_Pro5
Asparagusofficinalis_Pro4
Ananascomosus_Pro7
Brachypodiumdistachyon_Pro22
Hordeumvulgare_Pro63
Thinopyrumintermedium_Pro73
Triticumaestivum_Pro10
Oryzasativa_Pro4
Alloteropsissemialata_Pro21
Panicumvirgatum_Pro37
Setariaviridis_Pro2
Paspalumvaginatum_Pro23
Zeamays_Pro11

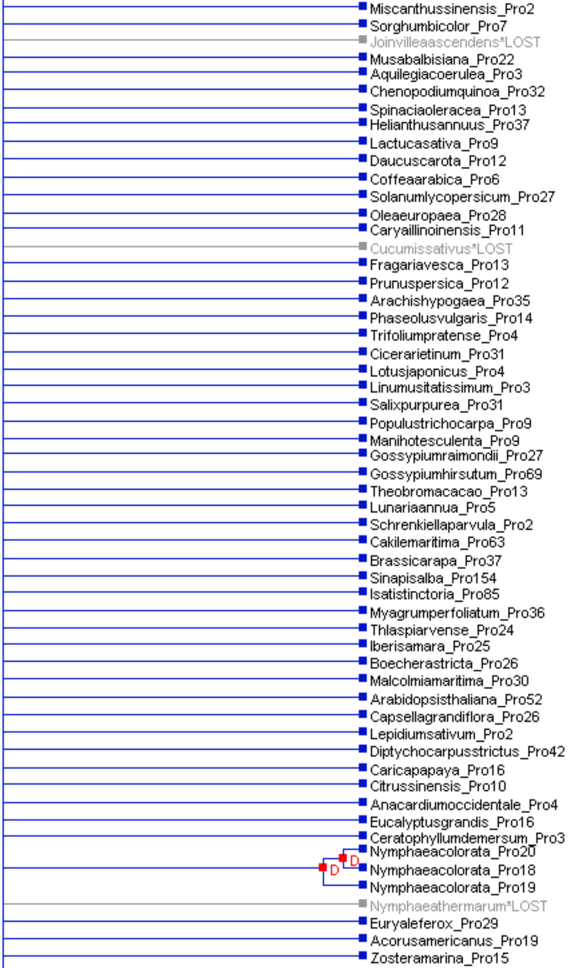
Miscanthussinensis_Pro2
Sorghumbicolor_Pro7
Joinvilleaascendens*LOST
Musabalbisiana_Pro22
Aquilegiacoerulea_Pro3
Chenopodiumquinoa_Pro32
Spinaciaoleracea_Pro13
Helianthusannuus_Pro37
Lactucasativa_Pro9
Daucuscarota_Pro12
Coffeaarabica_Pro6
Solanumlycopersicum_Pro27
Oleaeuropaea_Pro28
Caryaillinoinensis_Pro11
Cucumissativus*LOST
Fragariavesca_Pro13
Prunuspersica_Pro12
Arachishypogaea_Pro35
Phaseolusvulgaris_Pro14
Trifoliumpratense_Pro4
Cicerarietinum_Pro31
Lotusjaponicus_Pro4
Linumusitatissimum_Pro3
Salixpurpurea_Pro31
Populustrichocarpa_Pro9
Manihotesculenta_Pro9
Gossypiumraimondii_Pro27
Gossypiumhirsutum_Pro69
Theobromacacao_Pro13
Lunariaannua_Pro5
Schrenkiellaparvula_Pro2
Cakilemaritima_Pro63
Brassicarapa_Pro37
Sinapisalba_Pro154
Isatistinctoria_Pro85
Myagrumperfoliatum_Pro36
Thlaspiarvense_Pro24
Iberisamara_Pro25
Boecherastricta_Pro26
Malcolmiamaritima_Pro30
Arabidopsisthaliana_Pro52
Capsellagrandiflora_Pro26
Lepidiumsativum_Pro2
Diptychocarpusstrictus_Pro42
Caricapapaya_Pro16
Citrussinensis_Pro10
Anacardiumoccidentale_Pro4
Eucalyptusgrandis_Pro16
Ceratophyllumdemersum_Pro3
Nymphaeacolorata_Pro20
D
Nymphaeacolorata_Pro18
D
Nymphaeacolorata_Pro19
Nymphaeathermarum*LOST
Euryaleferox_Pro29
Acorusamericanus_Pro19
Zosteramarina_Pro15

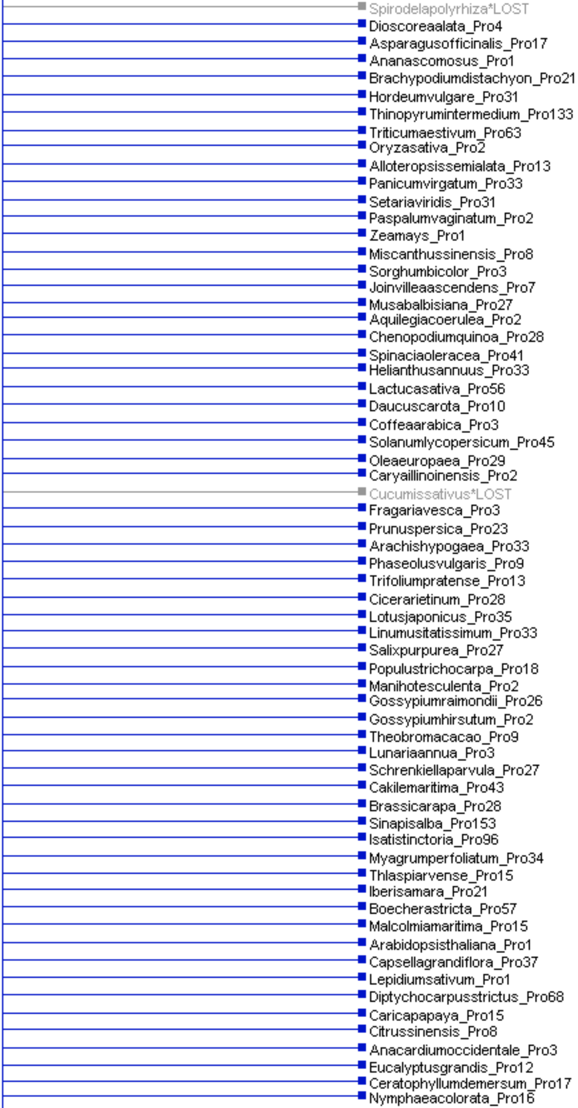
Spirodelapolyrhiza*LOST
Dioscoreaalata_Pro4
Asparagusofficinalis_Pro17
Ananascomosus_Pro1
Brachypodiumdistachyon_Pro21
Hordeumvulgare_Pro31
Thinopyrumintermedium_Pro133
Triticumaestivum_Pro63
Oryzasativa_Pro2
Alloteropsissemialata_Pro13
Panicumvirgatum_Pro33
Setariaviridis_Pro31
Paspalumvaginatum_Pro2
Zeamays_Pro1
Miscanthussinensis_Pro8
Sorghumbicolor_Pro3
Joinvilleaascendens_Pro7
Musabalbisiana_Pro27
Aquilegiacoerulea_Pro2
Chenopodiumquinoa_Pro28
Spinaciaoleracea_Pro41
Helianthusannuus_Pro33
Lactucasativa_Pro56
Daucuscarota_Pro10
Coffeaarabica_Pro3
Solanumlycopersicum_Pro45
Oleaeuropaea_Pro29
Caryaillinoinensis_Pro2
Cucumissativus*LOST
Fragariavesca_Pro3
Prunuspersica_Pro23
Arachishypogaea_Pro33
Phaseolusvulgaris_Pro9
Trifoliumpratense_Pro13
Cicerarietinum_Pro28
Lotusjaponicus_Pro35
Linumusitatissimum_Pro33
Salixpurpurea_Pro27
Populustrichocarpa_Pro18
Manihotesculenta_Pro2
Gossypiumraimondii_Pro26
Gossypiumhirsutum_Pro2
Theobromacacao_Pro9
Lunariaannua_Pro3
Schrenkiellaparvula_Pro27
Cakilemaritima_Pro43
Brassicarapa_Pro28
Sinapisalba_Pro153
Isatistinctoria_Pro96
Myagrumperfoliatum_Pro34
Thlaspiarvense_Pro15
Iberisamara_Pro21
Boecherastricta_Pro57
Malcolmiamaritima_Pro15
Arabidopsisthaliana_Pro1
Capsellagrandiflora_Pro37
Lepidiumsativum_Pro1
Diptychocarpusstrictus_Pro68
Caricapapaya_Pro15
Citrussinensis_Pro8
Anacardiumoccidentale_Pro3
Eucalyptusgrandis_Pro12
Ceratophyllumdemersum_Pro17
Nymphaeacolorata_Pro16

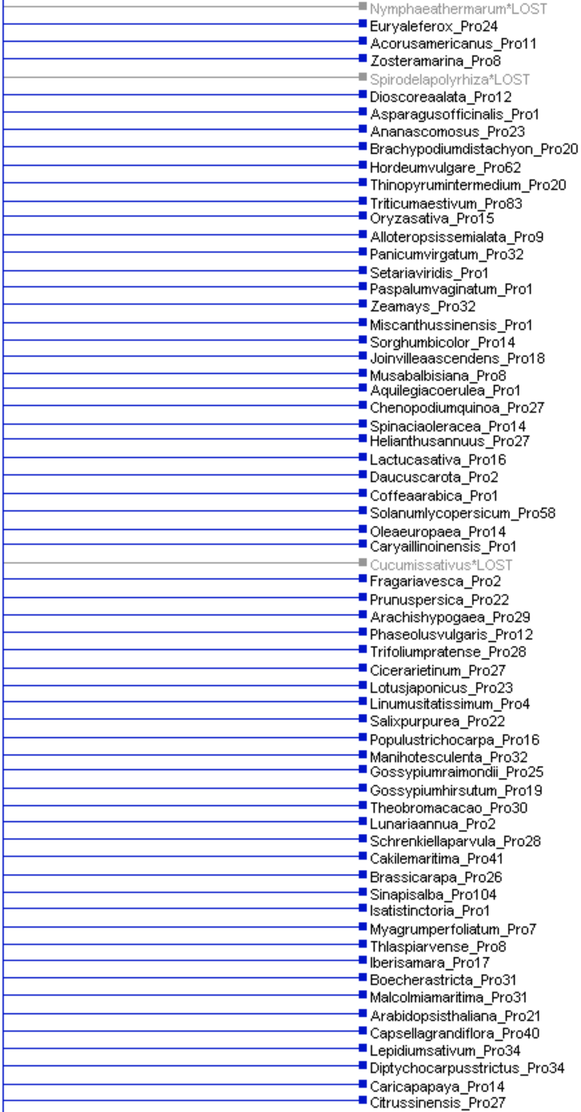
Nymphaeathermarum*LOST
Euryaleferox_Pro24
Acorusamericanus_Pro11
Zosteramarina_Pro8
Spirodelapolyrhiza*LOST
Dioscoreaalata_Pro12
Asparagusofficinalis_Pro1
Ananascomosus_Pro23
Brachypodiumdistachyon_Pro20
Hordeumvulgare_Pro62
Thinopyrumintermedium_Pro20
Triticumaestivum_Pro83
Oryzasativa_Pro15
Alloteropsissemialata_Pro9
Panicumvirgatum_Pro32
Setariaviridis_Pro1
Paspalumvaginatum_Pro1
Zeamays_Pro32
Miscanthussinensis_Pro1
Sorghumbicolor_Pro14
Joinvilleaascendens_Pro18
Musabalbisiana_Pro8
Aquilegiacoerulea_Pro1
Chenopodiumquinoa_Pro27
Spinaciaoleracea_Pro14
Helianthusannuus_Pro27
Lactucasativa_Pro16
Daucuscarota_Pro2
Coffeaarabica_Pro1
Solanumlycopersicum_Pro58
Oleaeuropaea_Pro14
Caryaillinoinensis_Pro1
Cucumissativus*LOST
Fragariavesca_Pro2
Prunuspersica_Pro22
Arachishypogaea_Pro29
Phaseolusvulgaris_Pro12
Trifoliumpratense_Pro28
Cicerarietinum_Pro27
Lotusjaponicus_Pro23
Linumusitatissimum_Pro4
Salixpurpurea_Pro22
Populustrichocarpa_Pro16
Manihotesculenta_Pro32
Gossypiumraimondii_Pro25
Gossypiumhirsutum_Pro19
Theobromacacao_Pro30
Lunariaannua_Pro2
Schrenkiellaparvula_Pro28
Cakilemaritima_Pro41
Brassicarapa_Pro26
Sinapisalba_Pro104
Isatistinctoria_Pro1
Myagrumperfoliatum_Pro7
Thlaspiarvense_Pro8
Iberisamara_Pro17
Boecherastricta_Pro31
Malcolmiamaritima_Pro31
Arabidopsisthaliana_Pro21
Capsellagrandiflora_Pro40
Lepidiumsativum_Pro34
Diptychocarpusstrictus_Pro34
Caricapapaya_Pro14
Citrussinensis_Pro27

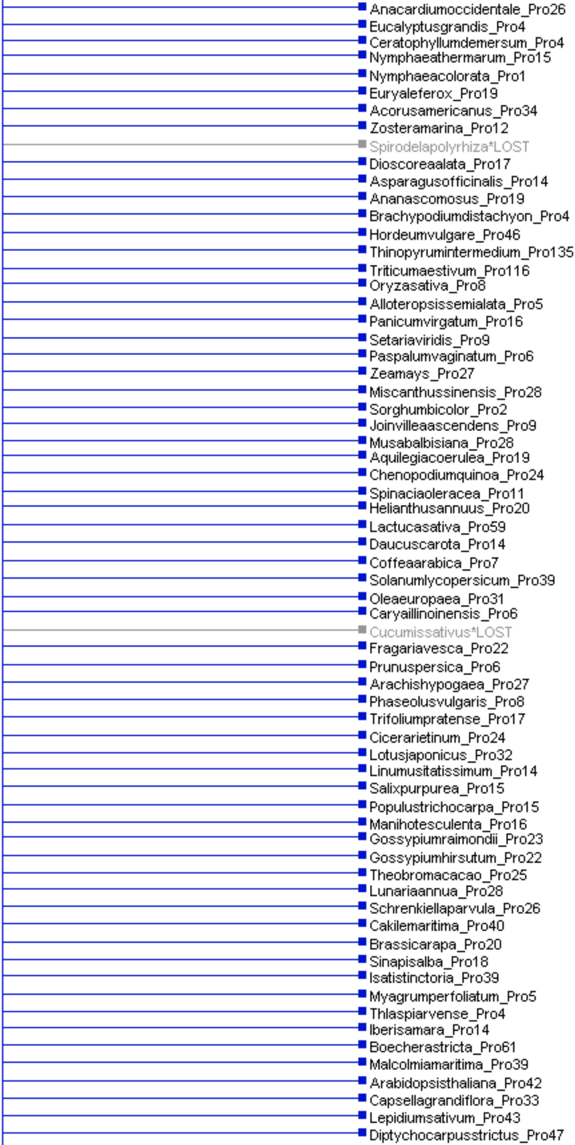
Anacardiumoccidentale_Pro26
Eucalyptusgrandis_Pro4
Ceratophyllumdemersum_Pro4
Nymphaeathermarum_Pro15
Nymphaeacolorata_Pro1
Euryaleferox_Pro19
Acorusamericanus_Pro34
Zosteramarina_Pro12
Spirodelapolyrhiza*LOST
Dioscoreaalata_Pro17
Asparagusofficinalis_Pro14
Ananascomosus_Pro19
Brachypodiumdistachyon_Pro4
Hordeumvulgare_Pro46
Thinopyrumintermedium_Pro135
Triticumaestivum_Pro116
Oryzasativa_Pro8
Alloteropsissemialata_Pro5
Panicumvirgatum_Pro16
Setariaviridis_Pro9
Paspalumvaginatum_Pro6
Zeamays_Pro27
Miscanthussinensis_Pro28
Sorghumbicolor_Pro2
Joinvilleaascendens_Pro9
Musabalbisiana_Pro28
Aquilegiacoerulea_Pro19
Chenopodiumquinoa_Pro24
Spinaciaoleracea_Pro11
Helianthusannuus_Pro20
Lactucasativa_Pro59
Daucuscarota_Pro14
Coffeaarabica_Pro7
Solanumlycopersicum_Pro39
Oleaeuropaea_Pro31
Caryaillinoinensis_Pro6
Cucumissativus*LOST
Fragariavesca_Pro22
Prunuspersica_Pro6
Arachishypogaea_Pro27
Phaseolusvulgaris_Pro8
Trifoliumpratense_Pro17
Cicerarietinum_Pro24
Lotusjaponicus_Pro32
Linumusitatissimum_Pro14
Salixpurpurea_Pro15
Populustrichocarpa_Pro15
Manihotesculenta_Pro16
Gossypiumraimondii_Pro23
Gossypiumhirsutum_Pro22
Theobromacacao_Pro25
Lunariaannua_Pro28
Schrenkiellaparvula_Pro26
Cakilemaritima_Pro40
Brassicarapa_Pro20
Sinapisalba_Pro18
Isatistinctoria_Pro39
Myagrumperfoliatum_Pro5
Thlaspiarvense_Pro4
Iberisamara_Pro14
Boecherastricta_Pro61
Malcolmiamaritima_Pro39
Arabidopsisthaliana_Pro42
Capsellagrandiflora_Pro33
Lepidiumsativum_Pro43
Diptychocarpusstrictus_Pro47

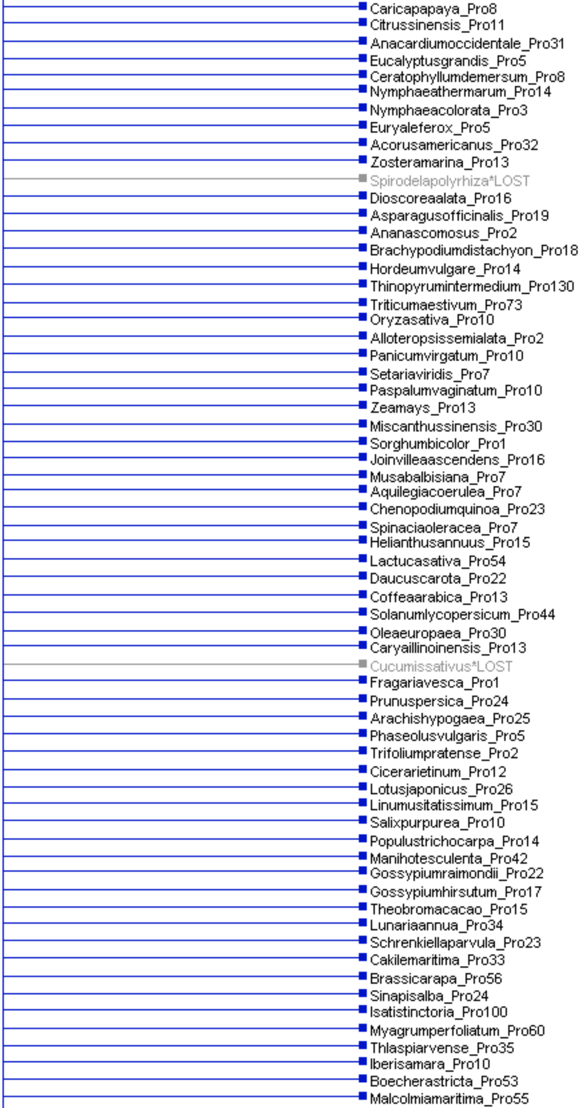
Caricapapaya_Pro8
Citrussinensis_Pro11
Anacardiumoccidentale_Pro31
Eucalyptusgrandis_Pro5
Ceratophyllumdemersum_Pro8
Nymphaeathermarum_Pro14
Nymphaeacolorata_Pro3
Euryaleferox_Pro5
Acorusamericanus_Pro32
Zosteramarina_Pro13
Spirodelapolyrhiza*LOST
Dioscoreaalata_Pro16
Asparagusofficinalis_Pro19
Ananascomosus_Pro2
Brachypodiumdistachyon_Pro18
Hordeumvulgare_Pro14
Thinopyrumintermedium_Pro130
Triticumaestivum_Pro73
Oryzasativa_Pro10
Alloteropsissemialata_Pro2
Panicumvirgatum_Pro10
Setariaviridis_Pro7
Paspalumvaginatum_Pro10
Zeamays_Pro13
Miscanthussinensis_Pro30
Sorghumbicolor_Pro1
Joinvilleaascendens_Pro16
Musabalbisiana_Pro7
Aquilegiacoerulea_Pro7
Chenopodiumquinoa_Pro23
Spinaciaoleracea_Pro7
Helianthusannuus_Pro15
Lactucasativa_Pro54
Daucuscarota_Pro22
Coffeaarabica_Pro13
Solanumlycopersicum_Pro44
Oleaeuropaea_Pro30
Caryaillinoinensis_Pro13
Cucumissativus*LOST
Fragariavesca_Pro1
Prunuspersica_Pro24
Arachishypogaea_Pro25
Phaseolusvulgaris_Pro5
Trifoliumpratense_Pro2
Cicerarietinum_Pro12
Lotusjaponicus_Pro26
Linumusitatissimum_Pro15
Salixpurpurea_Pro10
Populustrichocarpa_Pro14
Manihotesculenta_Pro42
Gossypiumraimondii_Pro22
Gossypiumhirsutum_Pro17
Theobromacacao_Pro15
Lunariaannua_Pro34
Schrenkiellaparvula_Pro23
Cakilemaritima_Pro33
Brassicarapa_Pro56
Sinapisalba_Pro24
Isatistinctoria_Pro100
Myagrumperfoliatum_Pro60
Thlaspiarvense_Pro35
Iberisamara_Pro10
Boecherastricta_Pro53
Malcolmiamaritima_Pro55

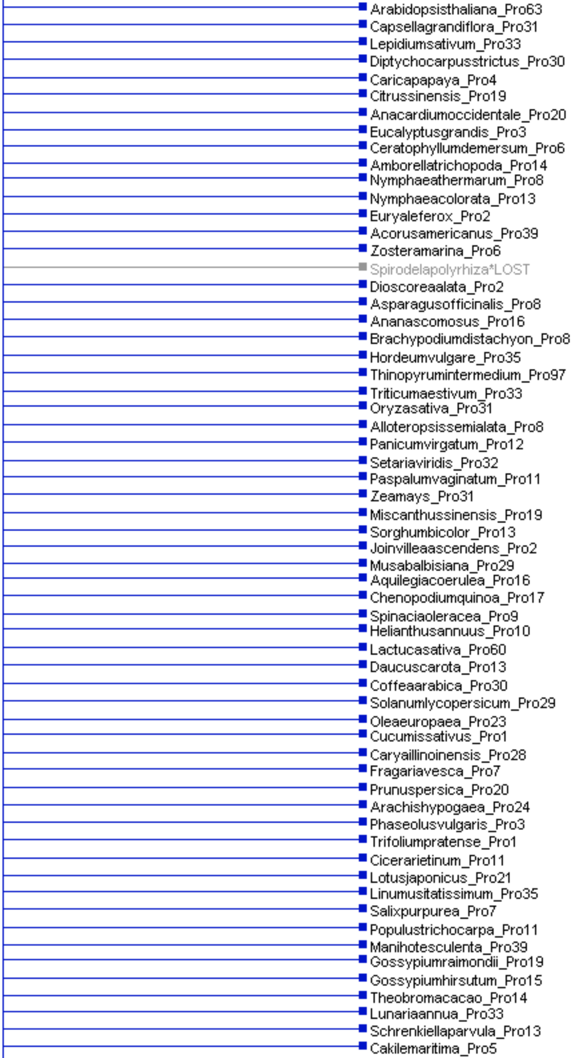
Arabidopsisthaliana_Pro63
Capsellagrandiflora_Pro31
Lepidiumsativum_Pro33
Diptychocarpusstrictus_Pro30
Caricapapaya_Pro4
Citrussinensis_Pro19
Anacardiumoccidentale_Pro20
Eucalyptusgrandis_Pro3
Ceratophyllumdemersum_Pro6
Amborellatrichopoda_Pro14
Nymphaeathermarum_Pro8
Nymphaeacolorata_Pro13
Euryaleferox_Pro2
Acorusamericanus_Pro39
Zosteramarina_Pro6
Spirodelapolyrhiza*LOST
Dioscoreaalata_Pro2
Asparagusofficinalis_Pro8
Ananascomosus_Pro16
Brachypodiumdistachyon_Pro8
Hordeumvulgare_Pro35
Thinopyrumintermedium_Pro97
Triticumaestivum_Pro33
Oryzasativa_Pro31
Alloteropsissemialata_Pro8
Panicumvirgatum_Pro12
Setariaviridis_Pro32
Paspalumvaginatum_Pro11
Zeamays_Pro31
Miscanthussinensis_Pro19
Sorghumbicolor_Pro13
Joinvilleaascendens_Pro2
Musabalbisiana_Pro29
Aquilegiacoerulea_Pro16
Chenopodiumquinoa_Pro17
Spinaciaoleracea_Pro9
Helianthusannuus_Pro10
Lactucasativa_Pro60
Daucuscarota_Pro13
Coffeaarabica_Pro30
Solanumlycopersicum_Pro29
Oleaeuropaea_Pro23
Cucumissativus_Pro1
Caryaillinoinensis_Pro28
Fragariavesca_Pro7
Prunuspersica_Pro20
Arachishypogaea_Pro24
Phaseolusvulgaris_Pro3
Trifoliumpratense_Pro1
Cicerarietinum_Pro11
Lotusjaponicus_Pro21
Linumusitatissimum_Pro35
Salixpurpurea_Pro7
Populustrichocarpa_Pro11
Manihotesculenta_Pro39
Gossypiumraimondii_Pro19
Gossypiumhirsutum_Pro15
Theobromacacao_Pro14
Lunariaannua_Pro33
Schrenkiellaparvula_Pro13
Cakilemaritima_Pro5

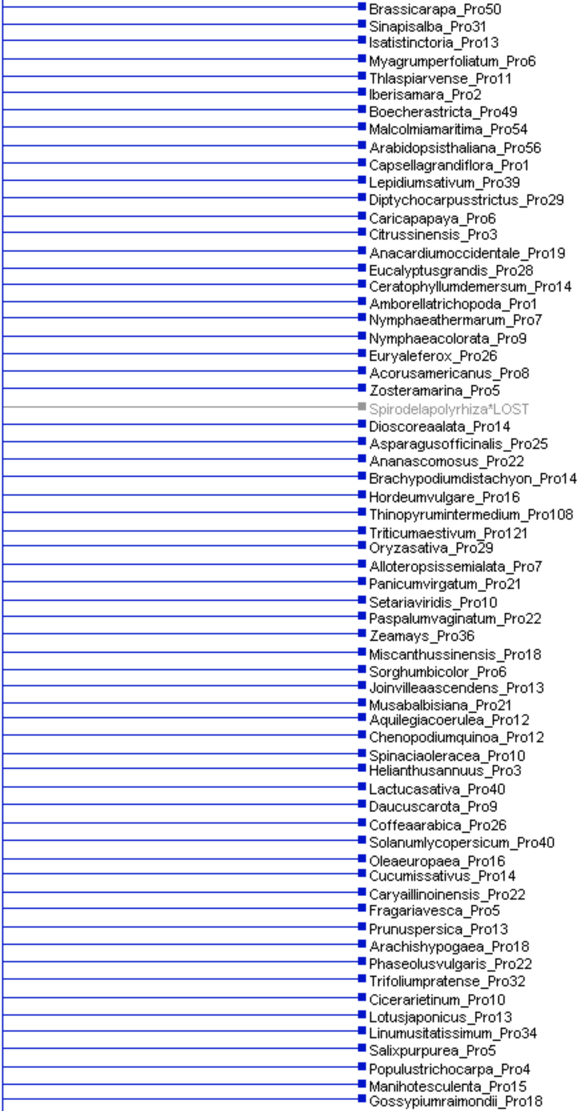
Brassicarapa_Pro50
Sinapisalba_Pro31
Isatistinctoria_Pro13
Myagrumperfoliatum_Pro6
Thlaspiarvense_Pro11
Iberisamara_Pro2
Boecherastricta_Pro49
Malcolmiamaritima_Pro54
Arabidopsisthaliana_Pro56
Capsellagrandiflora_Pro1
Lepidiumsativum_Pro39
Diptychocarpusstrictus_Pro29
Caricapapaya_Pro6
Citrussinensis_Pro3
Anacardiumoccidentale_Pro19
Eucalyptusgrandis_Pro28
Ceratophyllumdemersum_Pro14
Amborellatrichopoda_Pro1
Nymphaeathermarum_Pro7
Nymphaeacolorata_Pro9
Euryaleferox_Pro26
Acorusamericanus_Pro8
Zosteramarina_Pro5
Spirodelapolyrhiza*LOST
Dioscoreaalata_Pro14
Asparagusofficinalis_Pro25
Ananascomosus_Pro22
Brachypodiumdistachyon_Pro14
Hordeumvulgare_Pro16
Thinopyrumintermedium_Pro108
Triticumaestivum_Pro121
Oryzasativa_Pro29
Alloteropsissemialata_Pro7
Panicumvirgatum_Pro21
Setariaviridis_Pro10
Paspalumvaginatum_Pro22
Zeamays_Pro36
Miscanthussinensis_Pro18
Sorghumbicolor_Pro6
Joinvilleaascendens_Pro13
Musabalbisiana_Pro21
Aquilegiacoerulea_Pro12
Chenopodiumquinoa_Pro12
Spinaciaoleracea_Pro10
Helianthusannuus_Pro3
Lactucasativa_Pro40
Daucuscarota_Pro9
Coffeaarabica_Pro26
Solanumlycopersicum_Pro40
Oleaeuropaea_Pro16
Cucumissativus_Pro14
Caryaillinoinensis_Pro22
Fragariavesca_Pro5
Prunuspersica_Pro13
Arachishypogaea_Pro18
Phaseolusvulgaris_Pro22
Trifoliumpratense_Pro32
Cicerarietinum_Pro10
Lotusjaponicus_Pro13
Linumusitatissimum_Pro34
Salixpurpurea_Pro5
Populustrichocarpa_Pro4
Manihotesculenta_Pro15
Gossypiumraimondii_Pro18

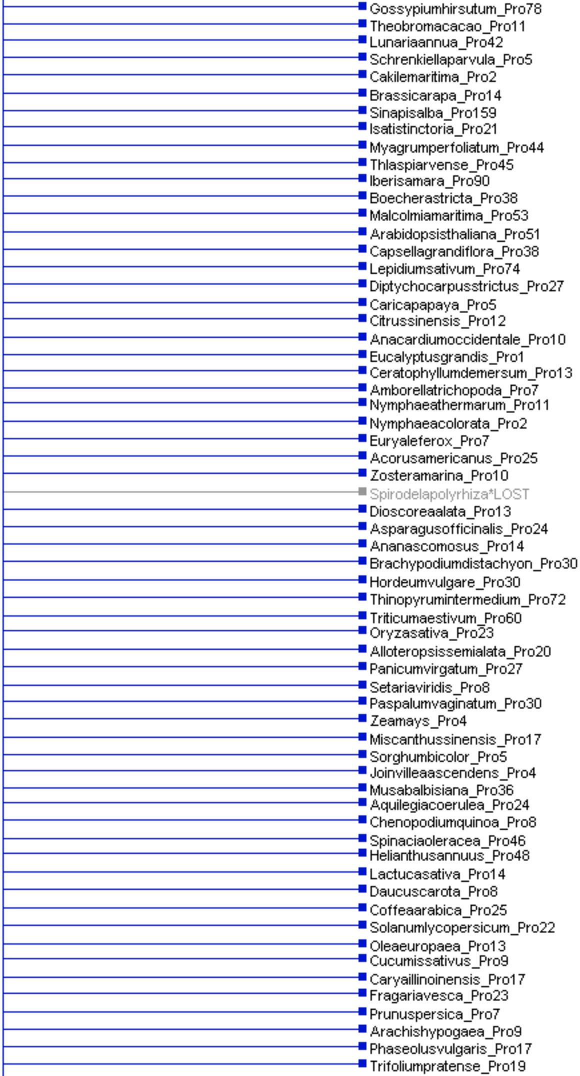
Gossypiumhirsutum_Pro78
Theobromacacao_Pro11
Lunariaannua_Pro42
Schrenkiellaparvula_Pro5
Cakilemaritima_Pro2
Brassicarapa_Pro14
Sinapisalba_Pro159
Isatistinctoria_Pro21
Myagrumperfoliatum_Pro44
Thlaspiarvense_Pro45
Iberisamara_Pro90
Boecherastricta_Pro38
Malcolmiamaritima_Pro53
Arabidopsisthaliana_Pro51
Capsellagrandiflora_Pro38
Lepidiumsativum_Pro74
Diptychocarpusstrictus_Pro27
Caricapapaya_Pro5
Citrussinensis_Pro12
Anacardiumoccidentale_Pro10
Eucalyptusgrandis_Pro1
Ceratophyllumdemersum_Pro13
Amborellatrichopoda_Pro7
Nymphaeathermarum_Pro11
Nymphaeacolorata_Pro2
Euryaleferox_Pro7
Acorusamericanus_Pro25
Zosteramarina_Pro10
Spirodelapolyrhiza*LOST
Dioscoreaalata_Pro13
Asparagusofficinalis_Pro24
Ananascomosus_Pro14
Brachypodiumdistachyon_Pro30
Hordeumvulgare_Pro30
Thinopyrumintermedium_Pro72
Triticumaestivum_Pro60
Oryzasativa_Pro23
Alloteropsissemialata_Pro20
Panicumvirgatum_Pro27
Setariaviridis_Pro8
Paspalumvaginatum_Pro30
Zeamays_Pro4
Miscanthussinensis_Pro17
Sorghumbicolor_Pro5
Joinvilleaascendens_Pro4
Musabalbisiana_Pro36
Aquilegiacoerulea_Pro24
Chenopodiumquinoa_Pro8
Spinaciaoleracea_Pro46
Helianthusannuus_Pro48
Lactucasativa_Pro14
Daucuscarota_Pro8
Coffeaarabica_Pro25
Solanumlycopersicum_Pro22
Oleaeuropaea_Pro13
Cucumissativus_Pro9
Caryaillinoinensis_Pro17
Fragariavesca_Pro23
Prunuspersica_Pro7
Arachishypogaea_Pro9
Phaseolusvulgaris_Pro17
Trifoliumpratense_Pro19

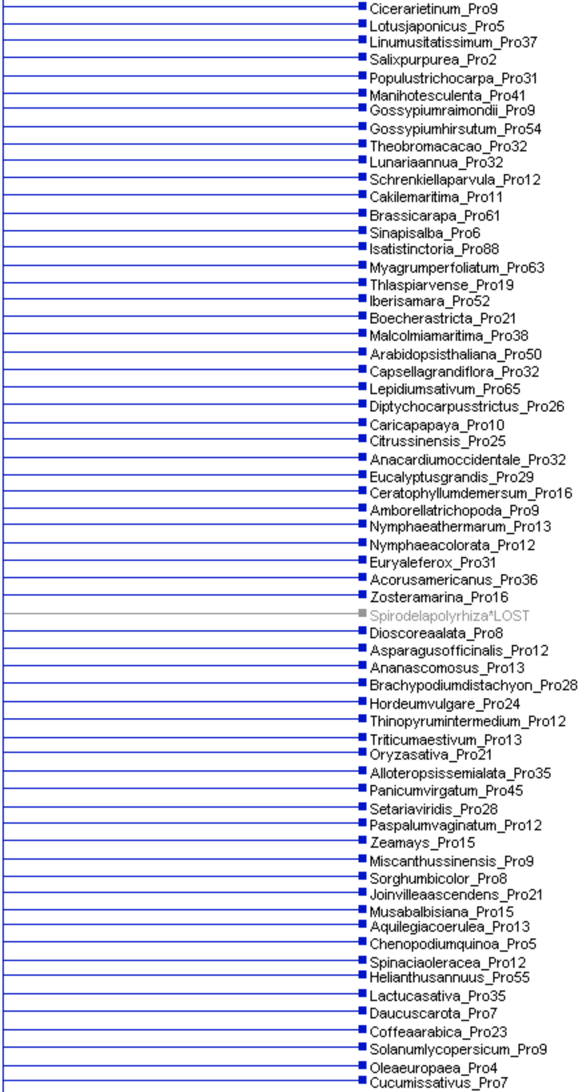
Cicerarietinum_Pro9
Lotusjaponicus_Pro5
Linumusitatissimum_Pro37
Salixpurpurea_Pro2
Populustrichocarpa_Pro31
Manihotesculenta_Pro41
Gossypiumraimondii_Pro9
Gossypiumhirsutum_Pro54
Theobromacacao_Pro32
Lunariaannua_Pro32
Schrenkiellaparvula_Pro12
Cakilemaritima_Pro11
Brassicarapa_Pro61
Sinapisalba_Pro6
Isatistinctoria_Pro88
Myagrumperfoliatum_Pro63
Thlaspiarvense_Pro19
Iberisamara_Pro52
Boecherastricta_Pro21
Malcolmiamaritima_Pro38
Arabidopsisthaliana_Pro50
Capsellagrandiflora_Pro32
Lepidiumsativum_Pro65
Diptychocarpusstrictus_Pro26
Caricapapaya_Pro10
Citrussinensis_Pro25
Anacardiumoccidentale_Pro32
Eucalyptusgrandis_Pro29
Ceratophyllumdemersum_Pro16
Amborellatrichopoda_Pro9
Nymphaeathermarum_Pro13
Nymphaeacolorata_Pro12
Euryaleferox_Pro31
Acorusamericanus_Pro36
Zosteramarina_Pro16
Spirodelapolyrhiza*LOST
Dioscoreaalata_Pro8
Asparagusofficinalis_Pro12
Ananascomosus_Pro13
Brachypodiumdistachyon_Pro28
Hordeumvulgare_Pro24
Thinopyrumintermedium_Pro12
Triticumaestivum_Pro13
Oryzasativa_Pro21
Alloteropsissemialata_Pro35
Panicumvirgatum_Pro45
Setariaviridis_Pro28
Paspalumvaginatum_Pro12
Zeamays_Pro15
Miscanthussinensis_Pro9
Sorghumbicolor_Pro8
Joinvilleaascendens_Pro21
Musabalbisiana_Pro15
Aquilegiacoerulea_Pro13
Chenopodiumquinoa_Pro5
Spinaciaoleracea_Pro12
Helianthusannuus_Pro55
Lactucasativa_Pro35
Daucuscarota_Pro7
Coffeaarabica_Pro23
Solanumlycopersicum_Pro9
Oleaeuropaea_Pro4
Cucumissativus_Pro7

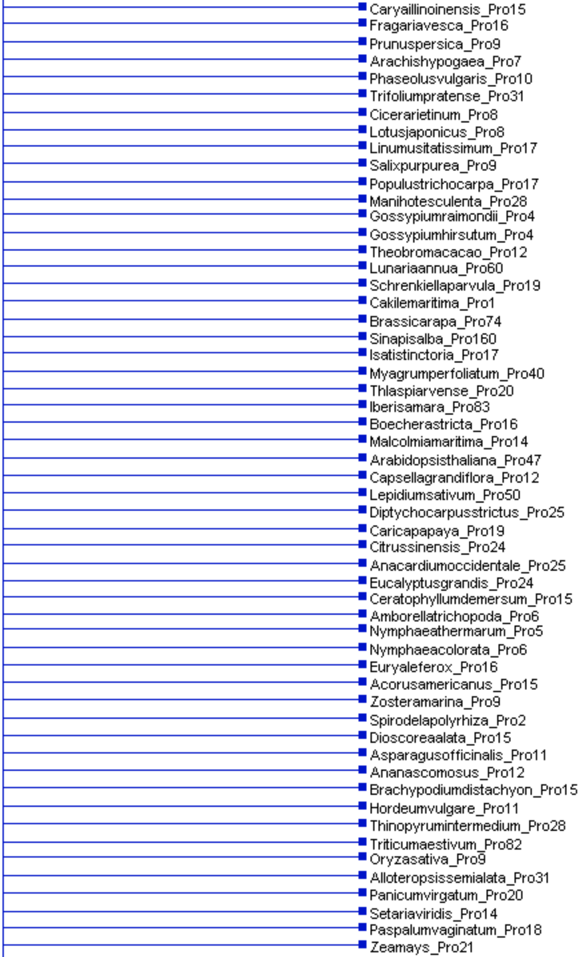
Caryaillinoinensis_Pro15
Fragariavesca_Pro16
Prunuspersica_Pro9
Arachishypogaea_Pro7
Phaseolusvulgaris_Pro10
Trifoliumpratense_Pro31
Cicerarietinum_Pro8
Lotusjaponicus_Pro8
Linumusitatissimum_Pro17
Salixpurpurea_Pro9
Populustrichocarpa_Pro17
Manihotesculenta_Pro28
Gossypiumraimondii_Pro4
Gossypiumhirsutum_Pro4
Theobromacacao_Pro12
Lunariaannua_Pro60
Schrenkiellaparvula_Pro19
Cakilemaritima_Pro1
Brassicarapa_Pro74
Sinapisalba_Pro160
Isatistinctoria_Pro17
Myagrumperfoliatum_Pro40
Thlaspiarvense_Pro20
Iberisamara_Pro83
Boecherastricta_Pro16
Malcolmiamaritima_Pro14
Arabidopsisthaliana_Pro47
Capsellagrandiflora_Pro12
Lepidiumsativum_Pro50
Diptychocarpusstrictus_Pro25
Caricapapaya_Pro19
Citrussinensis_Pro24
Anacardiumoccidentale_Pro25
Eucalyptusgrandis_Pro24
Ceratophyllumdemersum_Pro15
Amborellatrichopoda_Pro6
Nymphaeathermarum_Pro5
Nymphaeacolorata_Pro6
Euryaleferox_Pro16
Acorusamericanus_Pro15
Zosteramarina_Pro9
Spirodelapolyrhiza_Pro2
Dioscoreaalata_Pro15
Asparagusofficinalis_Pro11
Ananascomosus_Pro12
Brachypodiumdistachyon_Pro15
Hordeumvulgare_Pro11
Thinopyrumintermedium_Pro28
Triticumaestivum_Pro82
Oryzasativa_Pro9
Alloteropsissemialata_Pro31
Panicumvirgatum_Pro20
Setariaviridis_Pro14
Paspalumvaginatum_Pro18
Zeamays_Pro21

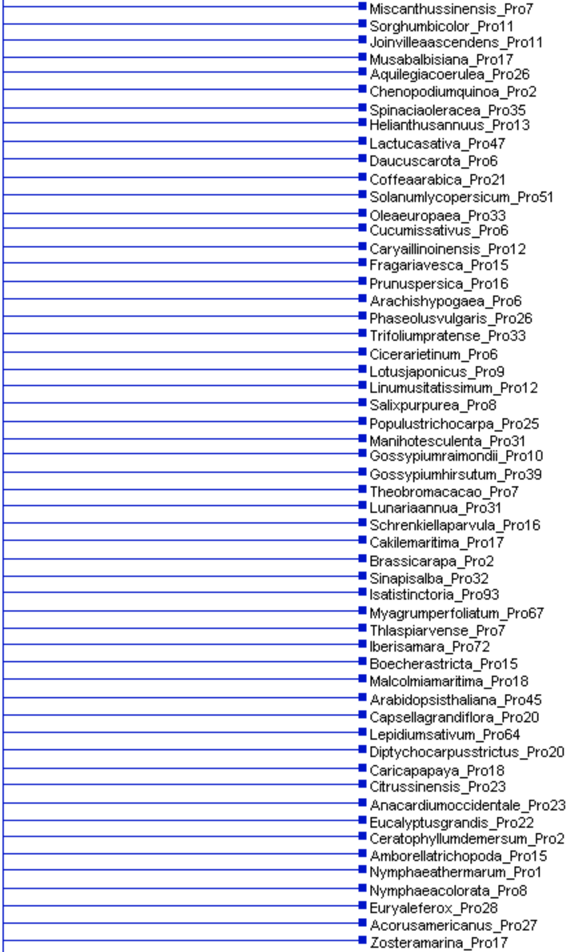
Miscanthussinensis_Pro7
Sorghumbicolor_Pro11
Joinvilleaascendens_Pro11
Musabalbisiana_Pro17
Aquilegiacoerulea_Pro26
Chenopodiumquinoa_Pro2
Spinaciaoleracea_Pro35
Helianthusannuus_Pro13
Lactucasativa_Pro47
Daucuscarota_Pro6
Coffeaarabica_Pro21
Solanumlycopersicum_Pro51
Oleaeuropaea_Pro33
Cucumissativus_Pro6
Caryaillinoinensis_Pro12
Fragariavesca_Pro15
Prunuspersica_Pro16
Arachishypogaea_Pro6
Phaseolusvulgaris_Pro26
Trifoliumpratense_Pro33
Cicerarietinum_Pro6
Lotusjaponicus_Pro9
Linumusitatissimum_Pro12
Salixpurpurea_Pro8
Populustrichocarpa_Pro25
Manihotesculenta_Pro31
Gossypiumraimondii_Pro10
Gossypiumhirsutum_Pro39
Theobromacacao_Pro7
Lunariaannua_Pro31
Schrenkiellaparvula_Pro16
Cakilemaritima_Pro17
Brassicarapa_Pro2
Sinapisalba_Pro32
Isatistinctoria_Pro93
Myagrumperfoliatum_Pro67
Thlaspiarvense_Pro7
Iberisamara_Pro72
Boecherastricta_Pro15
Malcolmiamaritima_Pro18
Arabidopsisthaliana_Pro45
Capsellagrandiflora_Pro20
Lepidiumsativum_Pro64
Diptychocarpusstrictus_Pro20
Caricapapaya_Pro18
Citrussinensis_Pro23
Anacardiumoccidentale_Pro23
Eucalyptusgrandis_Pro22
Ceratophyllumdemersum_Pro2
Amborellatrichopoda_Pro15
Nymphaeathermarum_Pro1
Nymphaeacolorata_Pro8
Euryaleferox_Pro28
Acorusamericanus_Pro27
Zosteramarina_Pro17

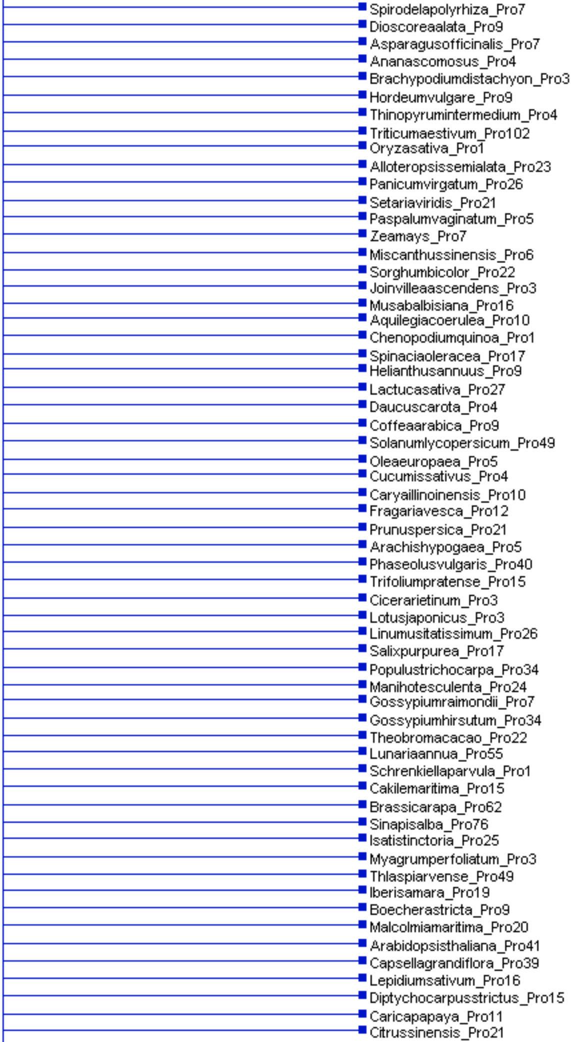
Spirodelapolyrhiza_Pro7
Dioscoreaalata_Pro9
Asparagusofficinalis_Pro7
Ananascomosus_Pro4
Brachypodiumdistachyon_Pro3
Hordeumvulgare_Pro9
Thinopyrumintermedium_Pro4
Triticumaestivum_Pro102
Oryzasativa_Pro1
Alloteropsissemialata_Pro23
Panicumvirgatum_Pro26
Setariaviridis_Pro21
Paspalumvaginatum_Pro5
Zeamays_Pro7
Miscanthussinensis_Pro6
Sorghumbicolor_Pro22
Joinvilleaascendens_Pro3
Musabalbisiana_Pro16
Aquilegiacoerulea_Pro10
Chenopodiumquinoa_Pro1
Spinaciaoleracea_Pro17
Helianthusannuus_Pro9
Lactucasativa_Pro27
Daucuscarota_Pro4
Coffeaarabica_Pro9
Solanumlycopersicum_Pro49
Oleaeuropaea_Pro5
Cucumissativus_Pro4
Caryaillinoinensis_Pro10
Fragariavesca_Pro12
Prunuspersica_Pro21
Arachishypogaea_Pro5
Phaseolusvulgaris_Pro40
Trifoliumpratense_Pro15
Cicerarietinum_Pro3
Lotusjaponicus_Pro3
Linumusitatissimum_Pro26
Salixpurpurea_Pro17
Populustrichocarpa_Pro34
Manihotesculenta_Pro24
Gossypiumraimondii_Pro7
Gossypiumhirsutum_Pro34
Theobromacacao_Pro22
Lunariaannua_Pro55
Schrenkiellaparvula_Pro1
Cakilemaritima_Pro15
Brassicarapa_Pro62
Sinapisalba_Pro76
Isatistinctoria_Pro25
Myagrumperfoliatum_Pro3
Thlaspiarvense_Pro49
Iberisamara_Pro19
Boecherastricta_Pro9
Malcolmiamaritima_Pro20
Arabidopsisthaliana_Pro41
Capsellagrandiflora_Pro39
Lepidiumsativum_Pro16
Diptychocarpusstrictus_Pro15
Caricapapaya_Pro11
Citrussinensis_Pro21

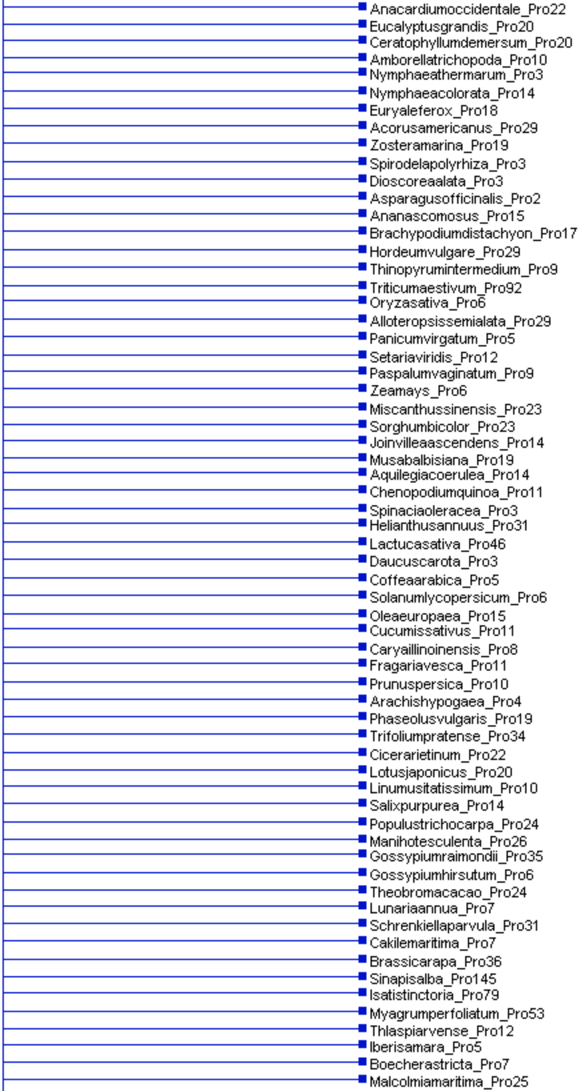
Anacardiumoccidentale_Pro22
Eucalyptusgrandis_Pro20
Ceratophyllumdemersum_Pro20
Amborellatrichopoda_Pro10
Nymphaeathermarum_Pro3
Nymphaeacolorata_Pro14
Euryaleferox_Pro18
Acorusamericanus_Pro29
Zosteramarina_Pro19
Spirodelapolyrhiza_Pro3
Dioscoreaalata_Pro3
Asparagusofficinalis_Pro2
Ananascomosus_Pro15
Brachypodiumdistachyon_Pro17
Hordeumvulgare_Pro29
Thinopyrumintermedium_Pro9
Triticumaestivum_Pro92
Oryzasativa_Pro6
Alloteropsissemialata_Pro29
Panicumvirgatum_Pro5
Setariaviridis_Pro12
Paspalumvaginatum_Pro9
Zeamays_Pro6
Miscanthussinensis_Pro23
Sorghumbicolor_Pro23
Joinvilleaascendens_Pro14
Musabalbisiana_Pro19
Aquilegiacoerulea_Pro14
Chenopodiumquinoa_Pro11
Spinaciaoleracea_Pro3
Helianthusannuus_Pro31
Lactucasativa_Pro46
Daucuscarota_Pro3
Coffeaarabica_Pro5
Solanumlycopersicum_Pro6
Oleaeuropaea_Pro15
Cucumissativus_Pro11
Caryaillinoinensis_Pro8
Fragariavesca_Pro11
Prunuspersica_Pro10
Arachishypogaea_Pro4
Phaseolusvulgaris_Pro19
Trifoliumpratense_Pro34
Cicerarietinum_Pro22
Lotusjaponicus_Pro20
Linumusitatissimum_Pro10
Salixpurpurea_Pro14
Populustrichocarpa_Pro24
Manihotesculenta_Pro26
Gossypiumraimondii_Pro35
Gossypiumhirsutum_Pro6
Theobromacacao_Pro24
Lunariaannua_Pro7
Schrenkiellaparvula_Pro31
Cakilemaritima_Pro7
Brassicarapa_Pro36
Sinapisalba_Pro145
Isatistinctoria_Pro79
Myagrumperfoliatum_Pro53
Thlaspiarvense_Pro12
Iberisamara_Pro5
Boecherastricta_Pro7
Malcolmiamaritima_Pro25

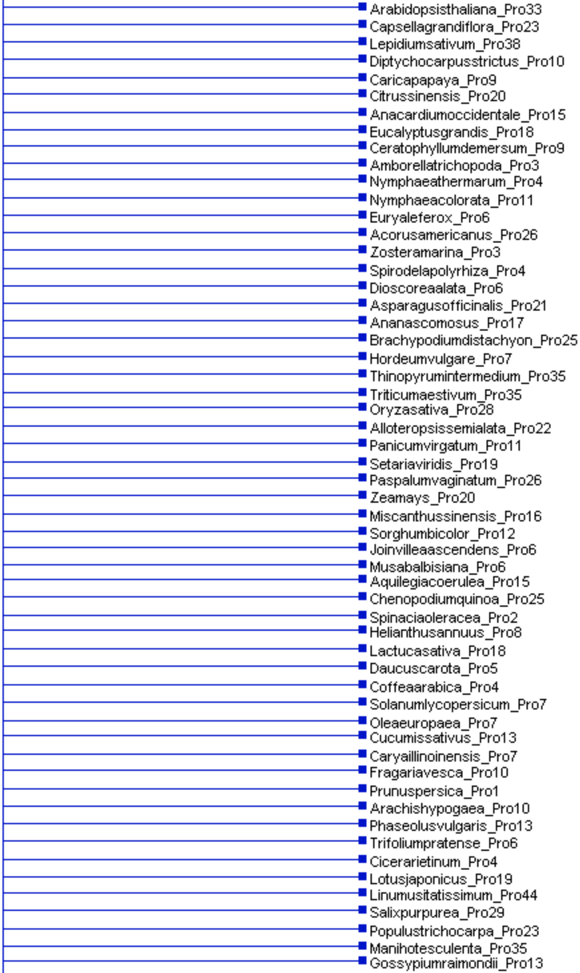

Arabidopsisthaliana_Pro33
Capsellagrandiflora_Pro23
Lepidiumsativum_Pro38
Diptychocarpusstrictus_Pro10
Caricapapaya_Pro9
Citrussinensis_Pro20
Anacardiumoccidentale_Pro15
Eucalyptusgrandis_Pro18
Ceratophyllumdemersum_Pro9
Amborellatrichopoda_Pro3
Nymphaeathermarum_Pro4
Nymphaeacolorata_Pro11
Euryaleferox_Pro6
Acorusamericanus_Pro26
Zosteramarina_Pro3
Spirodelapolyrhiza_Pro4
Dioscoreaalata_Pro6
Asparagusofficinalis_Pro21
Ananascomosus_Pro17
Brachypodiumdistachyon_Pro25
Hordeumvulgare_Pro7
Thinopyrumintermedium_Pro35
Triticumaestivum_Pro35
Oryzasativa_Pro28
Alloteropsissemialata_Pro22
Panicumvirgatum_Pro11
Setariaviridis_Pro19
Paspalumvaginatum_Pro26
Zeamays_Pro20
Miscanthussinensis_Pro16
Sorghumbicolor_Pro12
Joinvilleaascendens_Pro6
Musabalbisiana_Pro6
Aquilegiacoerulea_Pro15
Chenopodiumquinoa_Pro25
Spinaciaoleracea_Pro2
Helianthusannuus_Pro8
Lactucasativa_Pro18
Daucuscarota_Pro5
Coffeaarabica_Pro4
Solanumlycopersicum_Pro7
Oleaeuropaea_Pro7
Cucumissativus_Pro13
Caryaillinoinensis_Pro7
Fragariavesca_Pro10
Prunuspersica_Pro1
Arachishypogaea_Pro10
Phaseolusvulgaris_Pro13
Trifoliumpratense_Pro6
Cicerarietinum_Pro4
Lotusjaponicus_Pro19
Linumusitatissimum_Pro44
Salixpurpurea_Pro29
Populustrichocarpa_Pro23
Manihotesculenta_Pro35
Gossypiumraimondii_Pro13

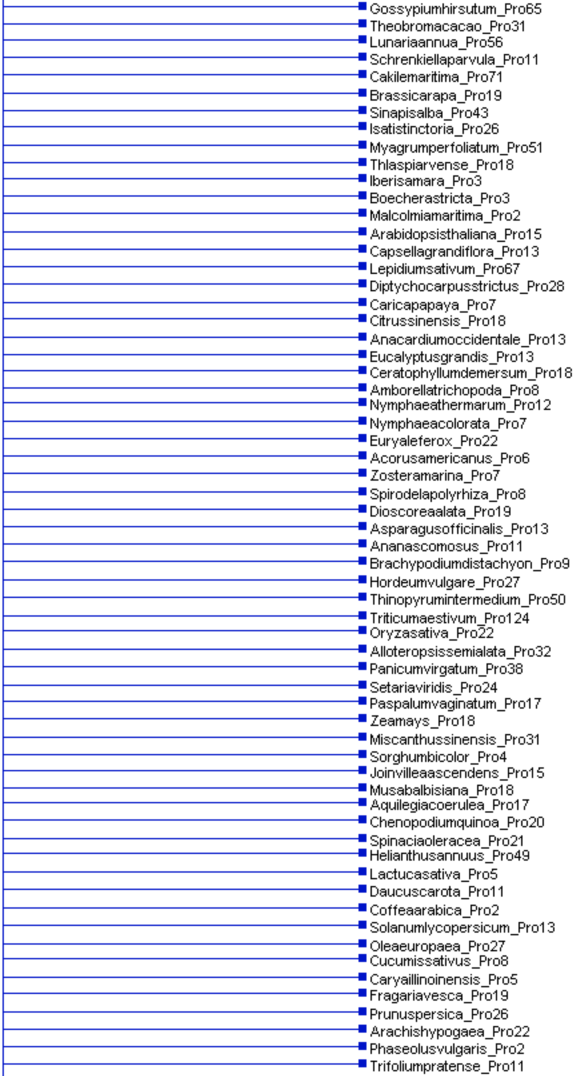
Gossypiumhirsutum_Pro65
Theobromacacao_Pro31
Lunariaannua_Pro56
Schrenkiellaparvula_Pro11
Cakilemaritima_Pro71
Brassicarapa_Pro19
Sinapisalba_Pro43
Isatistinctoria_Pro26
Myagrumperfoliatum_Pro51
Thlaspiarvense_Pro18
Iberisamara_Pro3
Boecherastricta_Pro3
Malcolmiamaritima_Pro2
Arabidopsisthaliana_Pro15
Capsellagrandiflora_Pro13
Lepidiumsativum_Pro67
Diptychocarpusstrictus_Pro28
Caricapapaya_Pro7
Citrussinensis_Pro18
Anacardiumoccidentale_Pro13
Eucalyptusgrandis_Pro13
Ceratophyllumdemersum_Pro18
Amborellatrichopoda_Pro8
Nymphaeathermarum_Pro12
Nymphaeacolorata_Pro7
Euryaleferox_Pro22
Acorusamericanus_Pro6
Zosteramarina_Pro7
Spirodelapolyrhiza_Pro8
Dioscoreaalata_Pro19
Asparagusofficinalis_Pro13
Ananascomosus_Pro11
Brachypodiumdistachyon_Pro9
Hordeumvulgare_Pro27
Thinopyrumintermedium_Pro50
Triticumaestivum_Pro124
Oryzasativa_Pro22
Alloteropsissemialata_Pro32
Panicumvirgatum_Pro38
Setariaviridis_Pro24
Paspalumvaginatum_Pro17
Zeamays_Pro18
Miscanthussinensis_Pro31
Sorghumbicolor_Pro4
Joinvilleaascendens_Pro15
Musabalbisiana_Pro18
Aquilegiacoerulea_Pro17
Chenopodiumquinoa_Pro20
Spinaciaoleracea_Pro21
Helianthusannuus_Pro49
Lactucasativa_Pro5
Daucuscarota_Pro11
Coffeaarabica_Pro2
Solanumlycopersicum_Pro13
Oleaeuropaea_Pro27
Cucumissativus_Pro8
Caryaillinoinensis_Pro5
Fragariavesca_Pro19
Prunuspersica_Pro26
Arachishypogaea_Pro22
Phaseolusvulgaris_Pro2
Trifoliumpratense_Pro11

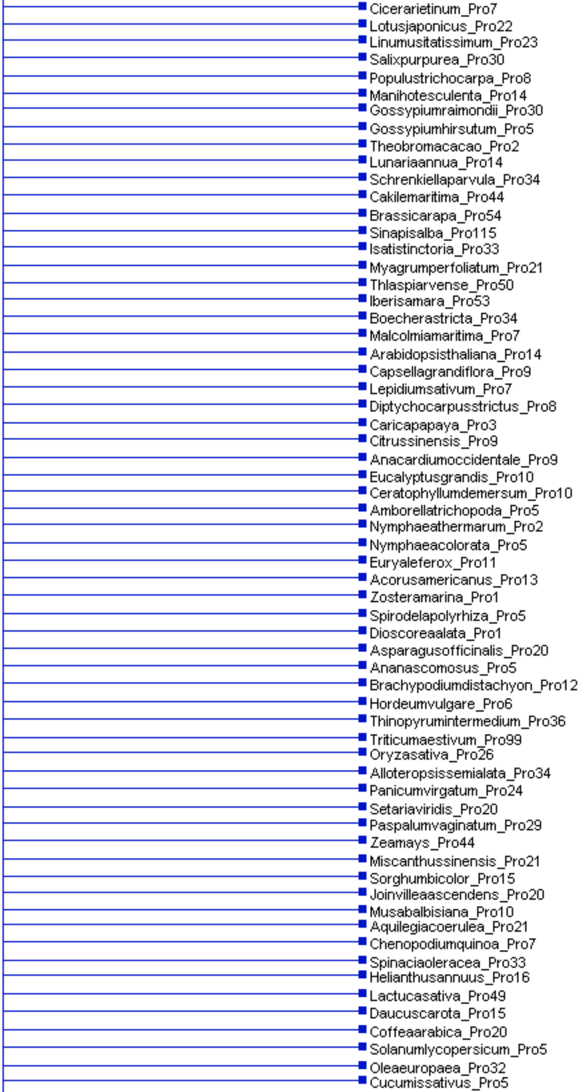
Cicerarietinum_Pro7
Lotusjaponicus_Pro22
Linumusitatissimum_Pro23
Salixpurpurea_Pro30
Populustrichocarpa_Pro8
Manihotesculenta_Pro14
Gossypiumraimondii_Pro30
Gossypiumhirsutum_Pro5
Theobromacacao_Pro2
Lunariaannua_Pro14
Schrenkiellaparvula_Pro34
Cakilemaritima_Pro44
Brassicarapa_Pro54
Sinapisalba_Pro115
Isatistinctoria_Pro33
Myagrumperfoliatum_Pro21
Thlaspiarvense_Pro50
Iberisamara_Pro53
Boecherastricta_Pro34
Malcolmiamaritima_Pro7
Arabidopsisthaliana_Pro14
Capsellagrandiflora_Pro9
Lepidiumsativum_Pro7
Diptychocarpusstrictus_Pro8
Caricapapaya_Pro3
Citrussinensis_Pro9
Anacardiumoccidentale_Pro9
Eucalyptusgrandis_Pro10
Ceratophyllumdemersum_Pro10
Amborellatrichopoda_Pro5
Nymphaeathermarum_Pro2
Nymphaeacolorata_Pro5
Euryaleferox_Pro11
Acorusamericanus_Pro13
Zosteramarina_Pro1
Spirodelapolyrhiza_Pro5
Dioscoreaalata_Pro1
Asparagusofficinalis_Pro20
Ananascomosus_Pro5
Brachypodiumdistachyon_Pro12
Hordeumvulgare_Pro6
Thinopyrumintermedium_Pro36
Triticumaestivum_Pro99
Oryzasativa_Pro26
Alloteropsissemialata_Pro34
Panicumvirgatum_Pro24
Setariaviridis_Pro20
Paspalumvaginatum_Pro29
Zeamays_Pro44
Miscanthussinensis_Pro21
Sorghumbicolor_Pro15
Joinvilleaascendens_Pro20
Musabalbisiana_Pro10
Aquilegiacoerulea_Pro21
Chenopodiumquinoa_Pro7
Spinaciaoleracea_Pro33
Helianthusannuus_Pro16
Lactucasativa_Pro49
Daucuscarota_Pro15
Coffeaarabica_Pro20
Solanumlycopersicum_Pro5
Oleaeuropaea_Pro32
Cucumissativus_Pro5

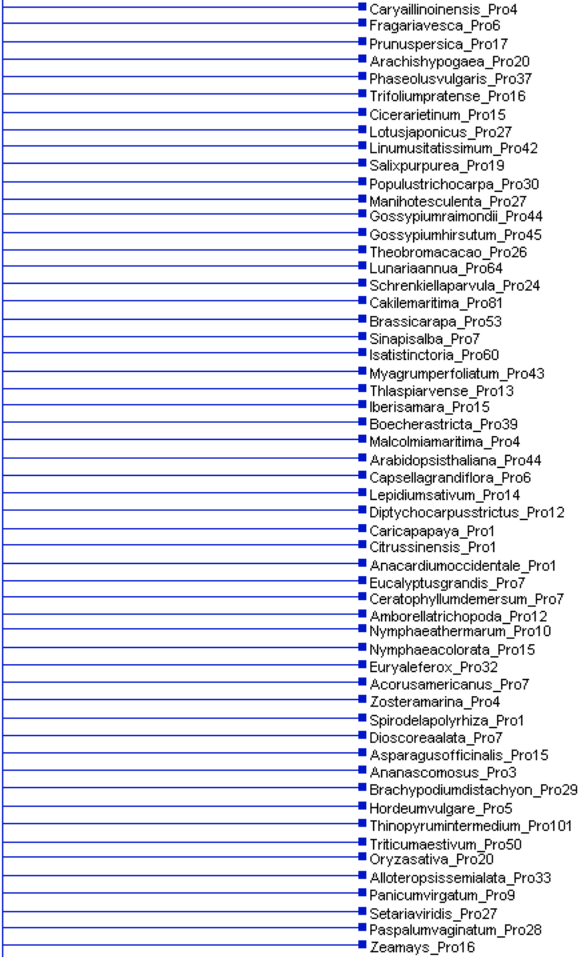
Caryaillinoinensis_Pro4
Fragariavesca_Pro6
Prunuspersica_Pro17
Arachishypogaea_Pro20
Phaseolusvulgaris_Pro37
Trifoliumpratense_Pro16
Cicerarietinum_Pro15
Lotusjaponicus_Pro27
Linumusitatissimum_Pro42
Salixpurpurea_Pro19
Populustrichocarpa_Pro30
Manihotesculenta_Pro27
Gossypiumraimondii_Pro44
Gossypiumhirsutum_Pro45
Theobromacacao_Pro26
Lunariaannua_Pro64
Schrenkiellaparvula_Pro24
Cakilemaritima_Pro81
Brassicarapa_Pro53
Sinapisalba_Pro7
Isatistinctoria_Pro60
Myagrumperfoliatum_Pro43
Thlaspiarvense_Pro13
Iberisamara_Pro15
Boecherastricta_Pro39
Malcolmiamaritima_Pro4
Arabidopsisthaliana_Pro44
Capsellagrandiflora_Pro6
Lepidiumsativum_Pro14
Diptychocarpusstrictus_Pro12
Caricapapaya_Pro1
Citrussinensis_Pro1
Anacardiumoccidentale_Pro1
Eucalyptusgrandis_Pro7
Ceratophyllumdemersum_Pro7
Amborellatrichopoda_Pro12
Nymphaeathermarum_Pro10
Nymphaeacolorata_Pro15
Euryaleferox_Pro32
Acorusamericanus_Pro7
Zosteramarina_Pro4
Spirodelapolyrhiza_Pro1
Dioscoreaalata_Pro7
Asparagusofficinalis_Pro15
Ananascomosus_Pro3
Brachypodiumdistachyon_Pro29
Hordeumvulgare_Pro5
Thinopyrumintermedium_Pro101
Triticumaestivum_Pro50
Oryzasativa_Pro20
Alloteropsissemialata_Pro33
Panicumvirgatum_Pro9
Setariaviridis_Pro27
Paspalumvaginatum_Pro28
Zeamays_Pro16

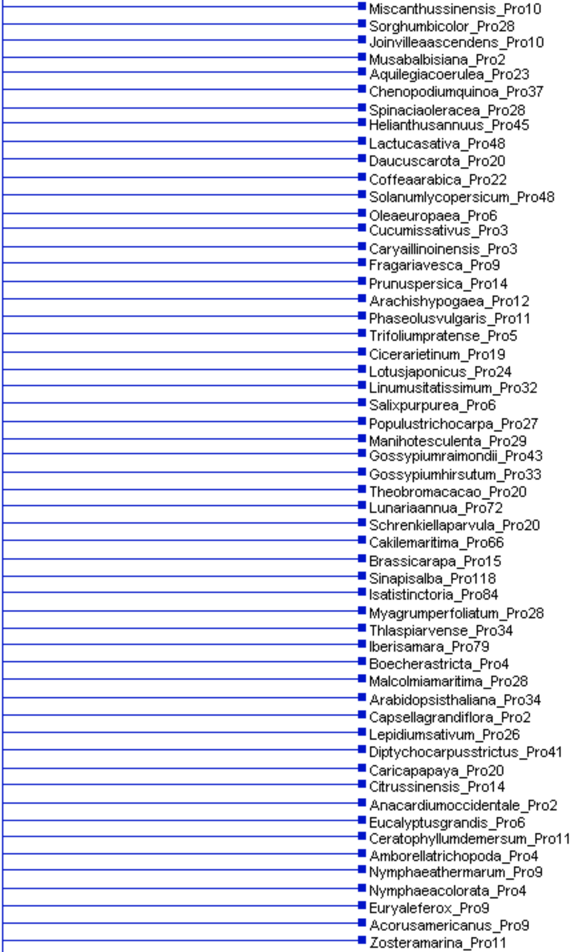
Miscanthussinensis_Pro10
Sorghumbicolor_Pro28
Joinvilleaascendens_Pro10
Musabalbisiana_Pro2
Aquilegiacoerulea_Pro23
Chenopodiumquinoa_Pro37
Spinaciaoleracea_Pro28
Helianthusannuus_Pro45
Lactucasativa_Pro48
Daucuscarota_Pro20
Coffeaarabica_Pro22
Solanumlycopersicum_Pro48
Oleaeuropaea_Pro6
Cucumissativus_Pro3
Caryaillinoinensis_Pro3
Fragariavesca_Pro9
Prunuspersica_Pro14
Arachishypogaea_Pro12
Phaseolusvulgaris_Pro11
Trifoliumpratense_Pro5
Cicerarietinum_Pro19
Lotusjaponicus_Pro24
Linumusitatissimum_Pro32
Salixpurpurea_Pro6
Populustrichocarpa_Pro27
Manihotesculenta_Pro29
Gossypiumraimondii_Pro43
Gossypiumhirsutum_Pro33
Theobromacacao_Pro20
Lunariaannua_Pro72
Schrenkiellaparvula_Pro20
Cakilemaritima_Pro66
Brassicarapa_Pro15
Sinapisalba_Pro118
Isatistinctoria_Pro84
Myagrumperfoliatum_Pro28
Thlaspiarvense_Pro34
Iberisamara_Pro79
Boecherastricta_Pro4
Malcolmiamaritima_Pro28
Arabidopsisthaliana_Pro34
Capsellagrandiflora_Pro2
Lepidiumsativum_Pro26
Diptychocarpusstrictus_Pro41
Caricapapaya_Pro20
Citrussinensis_Pro14
Anacardiumoccidentale_Pro2
Eucalyptusgrandis_Pro6
Ceratophyllumdemersum_Pro11
Amborellatrichopoda_Pro4
Nymphaeathermarum_Pro9
Nymphaeacolorata_Pro4
Euryaleferox_Pro9
Acorusamericanus_Pro9
Zosteramarina_Pro11

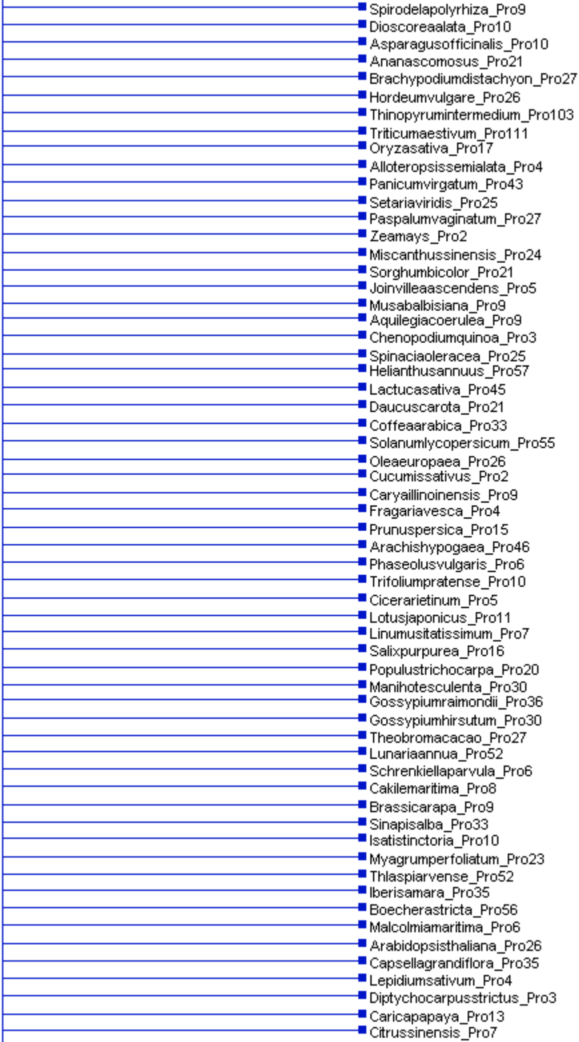
Spirodelapolyrhiza_Pro9
Dioscoreaalata_Pro10
Asparagusofficinalis_Pro10
Ananascomosus_Pro21
Brachypodiumdistachyon_Pro27
Hordeumvulgare_Pro26
Thinopyrumintermedium_Pro103
Triticumaestivum_Pro111
Oryzasativa_Pro17
Alloteropsissemialata_Pro4
Panicumvirgatum_Pro43
Setariaviridis_Pro25
Paspalumvaginatum_Pro27
Zeamays_Pro2
Miscanthussinensis_Pro24
Sorghumbicolor_Pro21
Joinvilleaascendens_Pro5
Musabalbisiana_Pro9
Aquilegiacoerulea_Pro9
Chenopodiumquinoa_Pro3
Spinaciaoleracea_Pro25
Helianthusannuus_Pro57
Lactucasativa_Pro45
Daucuscarota_Pro21
Coffeaarabica_Pro33
Solanumlycopersicum_Pro55
Oleaeuropaea_Pro26
Cucumissativus_Pro2
Caryaillinoinensis_Pro9
Fragariavesca_Pro4
Prunuspersica_Pro15
Arachishypogaea_Pro46
Phaseolusvulgaris_Pro6
Trifoliumpratense_Pro10
Cicerarietinum_Pro5
Lotusjaponicus_Pro11
Linumusitatissimum_Pro7
Salixpurpurea_Pro16
Populustrichocarpa_Pro20
Manihotesculenta_Pro30
Gossypiumraimondii_Pro36
Gossypiumhirsutum_Pro30
Theobromacacao_Pro27
Lunariaannua_Pro52
Schrenkiellaparvula_Pro6
Cakilemaritima_Pro8
Brassicarapa_Pro9
Sinapisalba_Pro33
Isatistinctoria_Pro10
Myagrumperfoliatum_Pro23
Thlaspiarvense_Pro52
Iberisamara_Pro35
Boecherastricta_Pro56
Malcolmiamaritima_Pro6
Arabidopsisthaliana_Pro26
Capsellagrandiflora_Pro35
Lepidiumsativum_Pro4
Diptychocarpusstrictus_Pro3
Caricapapaya_Pro13
Citrussinensis_Pro7

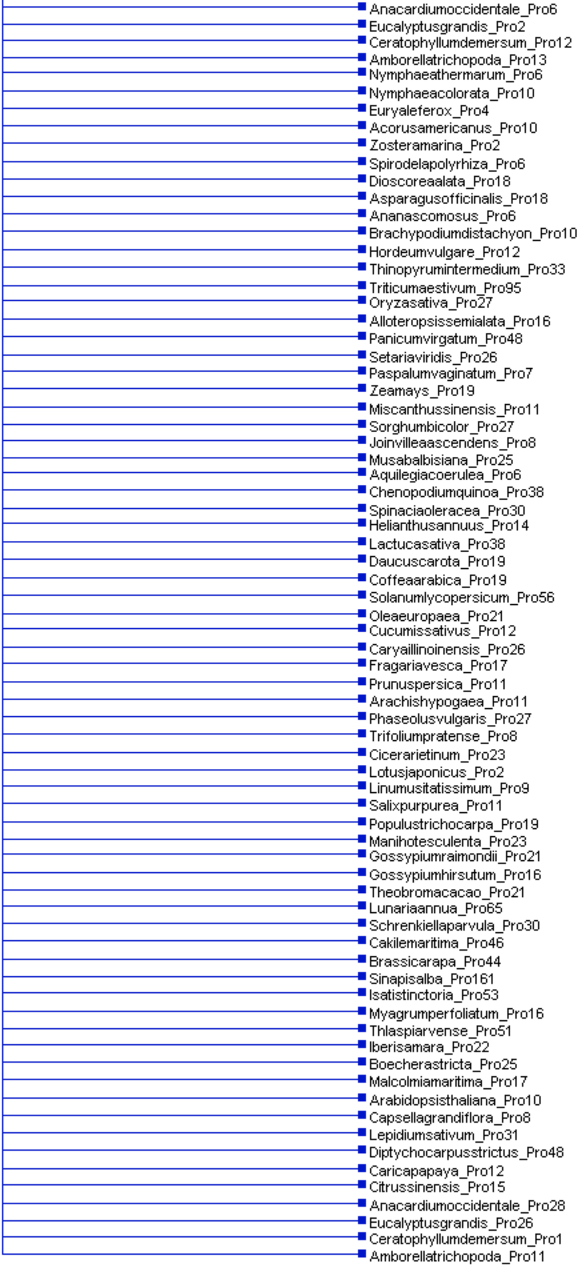

Image generated with Notung 2.9.1.5, on 05 22, 23
