# Supplementary material for: tRNA gene content, structure, and organization in the flowering plant lineage
Source: Front Plant Sci. 2024 Dec 23;15:1486612. doi: 10.3389/fpls.2024.1486612 (PMC11700998; doi:10.3389/fpls.2024.1486612)
Supplement: Supplementary file 6 [file DataSheet6.pdf]

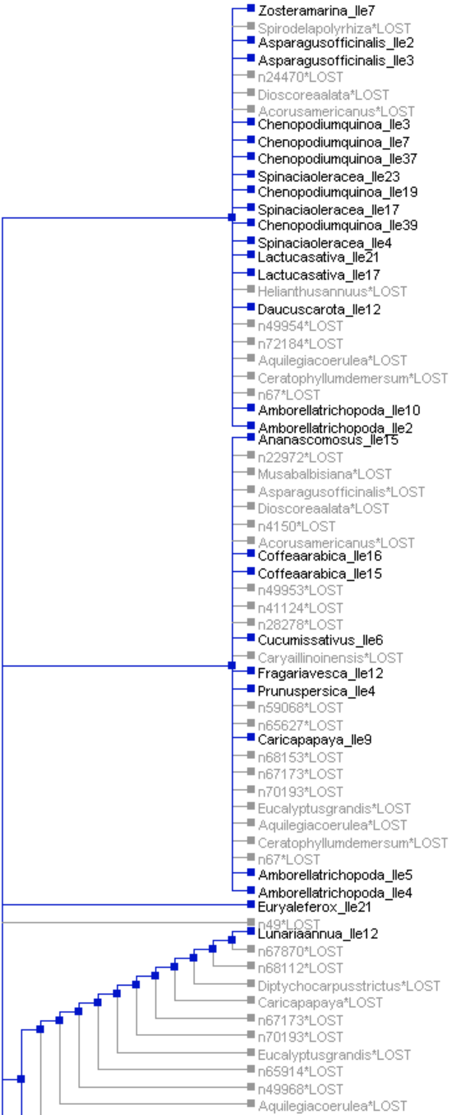
Zosteramarina_lle7
Spirodelapolyrhiza*LOST
Asparagusofficinalis_lle2
Asparagusofficinalis_lle3
n24470*LOST
Dioscoreaalata*LOST
Acorusamericanus*LOST
Chenopodiumquinoa_lle3
Chenopodiumquinoa_lle7
Chenopodiumquinoa_lle37
Spinaciaoleracea_lle23
Chenopodiumquinoa_lle19
Spinaciaoleracea_lle17
Chenopodiumquinoa_lle39
Spinaciaoleracea_lle4
Lactucasativa_lle21
Lactucasativa_lle17
Helianthusannuus*LOST
Daucuscarota_lle12
n49954*LOST
n72184*LOST
Aquilegiacoerulea*LOST
Ceratophyllumdemersum*LOST
n67*LOST
Amborellatrichopoda_lle10
Amborellatrichopoda_lle2
Ananascomosus_lle15
n22972*LOST
Musabalbisiana*LOST
Asparagusofficinalis*LOST
Dioscoreaalata*LOST
n4150*LOST
Acorusamericanus*LOST
Coffeaarabica_lle16
Coffeaarabica_lle15
n49953*LOST
n41124*LOST
n28278*LOST
Cucumissativus_lle6
Caryaillinoinensis*LOST
Fragariavesca_lle12
Prunuspersica_lle4
n59068*LOST
n65627*LOST
Caricapapaya_lle9
n68153*LOST
n67173*LOST
n70193*LOST
Eucalyptusgrandis*LOST
Aquilegiacoerulea*LOST
Ceratophyllumdemersum*LOST
n67*LOST
Amborellatrichopoda_lle5
Amborellatrichopoda_lle4
Euryaleferox_lle21
n49*LOST
Lunariaannua_lle12
n67870*LOST
n68112*LOST
Diptychocarpusstrictus*LOST
Caricapapaya*LOST
n67173*LOST
n70193*LOST
Eucalyptusgrandis*LOST
n65914*LOST
n49968*LOST
Aquilegiacoerulea*LOST

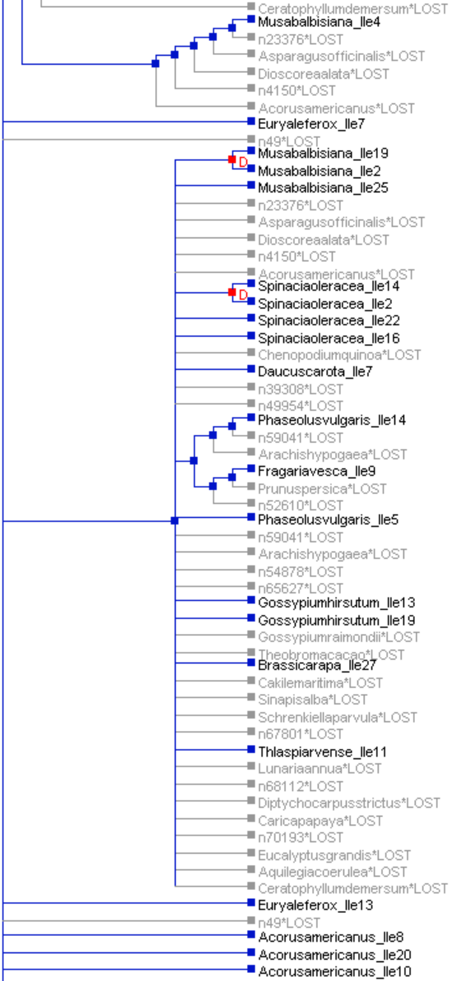
Ceratophyllumdemersum*LOST
Musabalbisiana_lle4
n23376*LOST
Asparagusofficinalis*LOST
Dioscoreaalata*LOST
n4150*LOST
Acorusamericanus*LOST
Euryaleferox_lle7
n49*LOST
Musabalbisiana_lle19
D
Musabalbisiana_lle2
Musabalbisiana_lle25
n23376*LOST
Asparagusofficinalis*LOST
Dioscoreaalata*LOST
n4150*LOST
Acorusamericanus*LOST
Spinaciaoleracea_lle14
D
Spinaciaoleracea_lle2
Spinaciaoleracea_lle22
Spinaciaoleracea_lle16
Chenopodiumquinoa*LOST
Daucuscarota_lle7
n39308*LOST
n49954*LOST
Phaseolusvulgaris_lle14
n59041*LOST
Arachishypogaea*LOST
Fragariavesca_lle9
Prunuspersica*LOST
n52610*LOST
Phaseolusvulgaris_lle5
n59041*LOST
Arachishypogaea*LOST
n54878*LOST
n65627*LOST
Gossypiumhirsutum_lle13
Gossypiumhirsutum_lle19
Gossypiumraimondii*LOST
Theobromacacao*LOST
Brassicarapa_lle27
Cakilemaritima*LOST
Sinapisalba*LOST
Schrenkiellaparvula*LOST
n67801*LOST
Thlaspiarvense_lle11
Lunariaannua*LOST
n68112*LOST
Diptychocarpusstrictus*LOST
Caricapapaya*LOST
n70193*LOST
Eucalyptusgrandis*LOST
Aquilegiacoerulea*LOST
Ceratophyllumdemersum*LOST
Euryaleferox_lle13
n49*LOST
Acorusamericanus_lle8
Acorusamericanus_lle20
Acorusamericanus_lle10

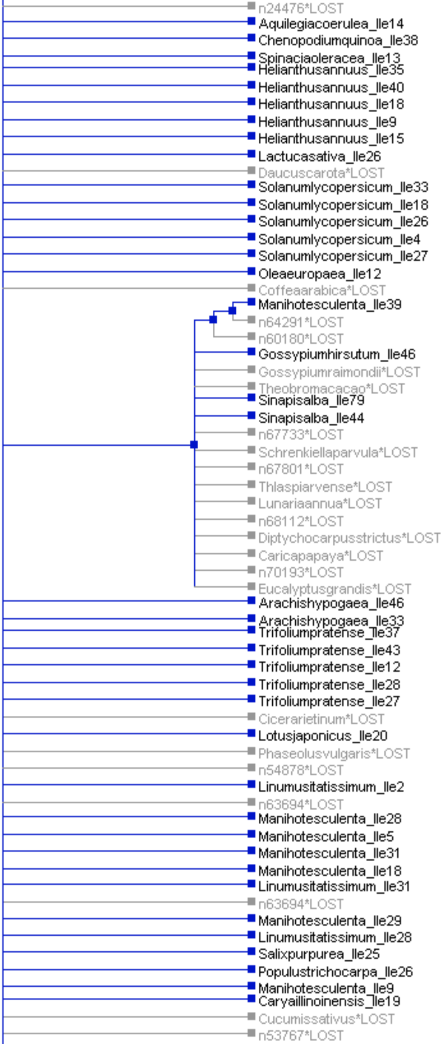
n24476*LOST
Aquilegiacoerulea_lle14
Chenopodiumquinoa_lle38
Spinaciaoleracea_lle13
Helianthusannuus_lle35
Helianthusannuus_lle40
Helianthusannuus_lle18
Helianthusannuus_lle9
Helianthusannuus_lle15
Lactucasativa_lle26
Daucuscarota*LOST
Solanumlycopersicum_lle33
Solanumlycopersicum_lle18
Solanumlycopersicum_lle26
Solanumlycopersicum_lle4
Solanumlycopersicum_lle27
Oleaeuropaea_lle12
Coffeaarabica*LOST
Manihotesculenta_lle39
n64291*LOST
n60180*LOST
Gossypiumhirsutum_lle46
Gossypiumraimondii*LOST
Theobromacacao*LOST
Sinapisalba_lle79
Sinapisalba_lle44
n67733*LOST
Schrenkiellaparvula*LOST
n67801*LOST
Thlaspiarvense*LOST
Lunariaannua*LOST
n68112*LOST
Diptychocarpusstrictus*LOST
Caricapapaya*LOST
n70193*LOST
Eucalyptusgrandis*LOST
Arachishypogaea_lle46
Arachishypogaea_lle33
Trifoliumpratense_lle37
Trifoliumpratense_lle43
Trifoliumpratense_lle12
Trifoliumpratense_lle28
Trifoliumpratense_lle27
Cicerarietinum*LOST
Lotusjaponicus_lle20
Phaseolusvulgaris*LOST
n54878*LOST
Linumusitatissimum_lle2
n63694*LOST
Manihotesculenta_lle28
Manihotesculenta_lle5
Manihotesculenta_lle31
Manihotesculenta_lle18
Linumusitatissimum_lle31
n63694*LOST
Manihotesculenta_lle29
Linumusitatissimum_lle28
Salixpurpurea_lle25
Populustrichocarpa_lle26
Manihotesculenta_lle9
Caryaillinoinensis_lle19
Cucumissativus*LOST
n53767*LOST

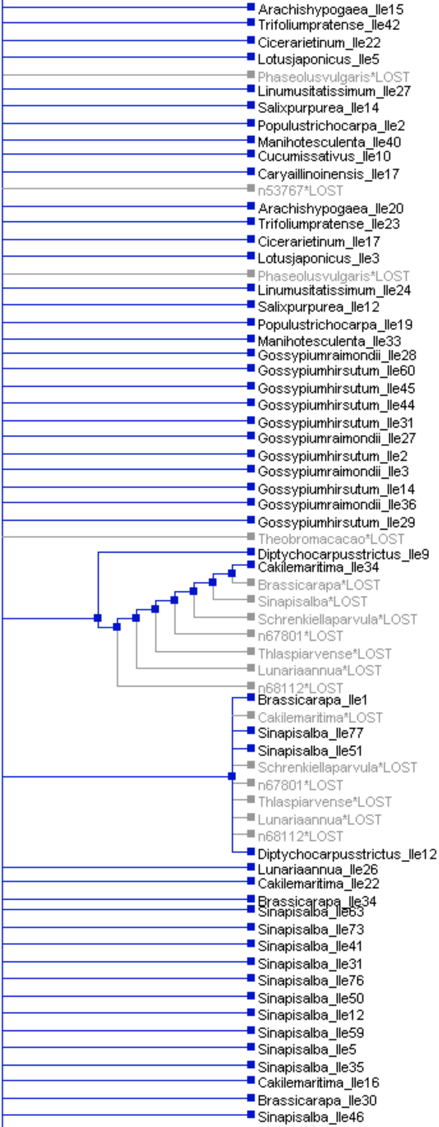
Arachishypogaea_lle15
Trifoliumpratense_lle42
Cicerarietinum_lle22
Lotusjaponicus_lle5
Phaseolusvulgaris*LOST
Linumusitatissimum_lle27
Salixpurpurea_lle14
Populustrichocarpa_lle2
Manihotesculenta_lle40
Cucumissativus_lle10
Caryaillinoinensis_lle17
n53767*LOST
Arachishypogaea_lle20
Trifoliumpratense_lle23
Cicerarietinum_lle17
Lotusjaponicus_lle3
Phaseolusvulgaris*LOST
Linumusitatissimum_lle24
Salixpurpurea_lle12
Populustrichocarpa_lle19
Manihotesculenta_lle33
Gossypiumraimondii_lle28
Gossypiumhirsutum_lle60
Gossypiumhirsutum_lle45
Gossypiumhirsutum_lle44
Gossypiumhirsutum_lle31
Gossypiumraimondii_lle27
Gossypiumhirsutum_lle2
Gossypiumraimondii_lle3
Gossypiumhirsutum_lle14
Gossypiumraimondii_lle36
Gossypiumhirsutum_lle29
Theobromacacao*LOST
Diptychocarpusstrictus_lle9
Cakilemaritima_lle34
Brassicarapa*LOST
Sinapisalba*LOST
Schrenkiellaparvula*LOST
n67801*LOST
Thlaspiarvense*LOST
Lunariaannua*LOST
n68112*LOST
Brassicarapa_lle1
Cakilemaritima*LOST
Sinapisalba_lle77
Sinapisalba_lle51
Schrenkiellaparvula*LOST
n67801*LOST
Thlaspiarvense*LOST
Lunariaannua*LOST
n68112*LOST
Diptychocarpusstrictus_lle12
Lunariaannua_lle26
Cakilemaritima_lle22
Brassicarapa_lle34
Sinapisalba_lle63
Sinapisalba_lle73
Sinapisalba_lle41
Sinapisalba_lle31
Sinapisalba_lle76
Sinapisalba_lle50
Sinapisalba_lle12
Sinapisalba_lle59
Sinapisalba_lle5
Sinapisalba_lle35
Cakilemaritima_lle16
Brassicarapa_lle30
Sinapisalba_lle46

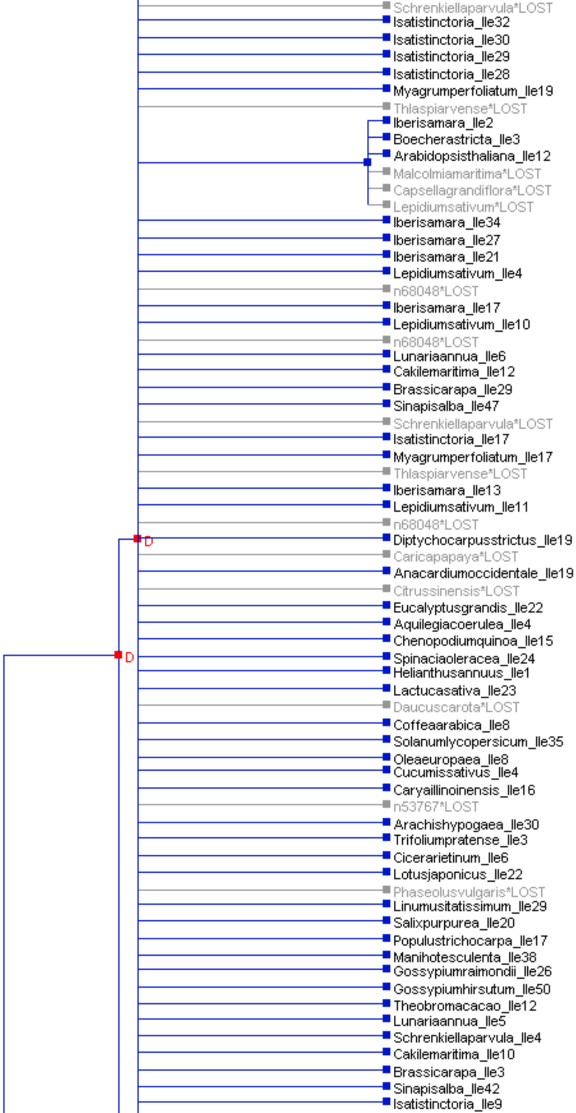
Schrenkiellaparvula*LOST
Isatistinctoria_lle32
Isatistinctoria_lle30
Isatistinctoria_lle29
Isatistinctoria_lle28
Myagrumperfoliatum_lle19
Thlaspiarvense*LOST
Iberisamara_lle2
Boecherastricta_lle3
Arabidopsisthaliana_lle12
Malcolmiamaritima*LOST
Capsellagrandiflora*LOST
Lepidiumsativum*LOST
Iberisamara_lle34
Iberisamara_lle27
Iberisamara_lle21
Lepidiumsativum_lle4
n68048*LOST
Iberisamara_lle17
Lepidiumsativum_lle10
n68048*LOST
Lunariaannua_lle6
Cakilemaritima_lle12
Brassicarapa_lle29
Sinapisalba_lle47
Schrenkiellaparvula*LOST
Isatistinctoria_lle17
Myagrumperfoliatum_lle17
Thlaspiarvense*LOST
Iberisamara_lle13
Lepidiumsativum_lle11
n68048*LOST
D
Diptychocarpusstrictus_lle19
Caricapapaya*LOST
Anacardiumoccidentale_lle19
Citrussinensis*LOST
Eucalyptusgrandis_lle22
Aquilegiacoerulea_lle4
Chenopodiumquinoa_lle15
D
Spinaciaoleracea_lle24
Helianthusannuus_lle1
Lactucasativa_lle23
Daucuscarota*LOST
Coffeaarabica_lle8
Solanumlycopersicum_lle35
Oleaeuropaea_lle8
Cucumissativus_lle4
Caryaillinoinensis_lle16
n53767*LOST
Arachishypogaea_lle30
Trifoliumpratense_lle3
Cicerarietinum_lle6
Lotusjaponicus_lle22
Phaseolusvulgaris*LOST
Linumusitatissimum_lle29
Salixpurpurea_lle20
Populustrichocarpa_lle17
Manihotesculenta_lle38
Gossypiumraimondii_lle26
Gossypiumhirsutum_lle50
Theobromacacao_lle12
Lunariaannua_lle5
Schrenkiellaparvula_lle4
Cakilemaritima_lle10
Brassicarapa_lle3
Sinapisalba_lle42
Isatistinctoria_lle9

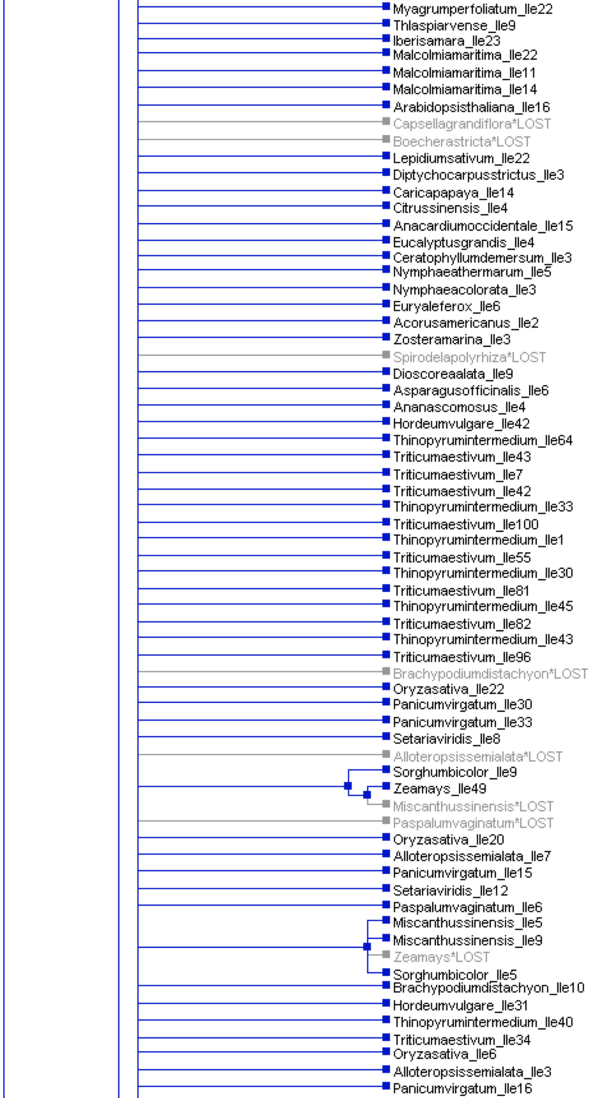
Myagrumperfoliatum_lle22
Thlaspiarvense_lle9
Iberisamara_lle23
Malcolmiamaritima_lle22
Malcolmiamaritima_lle11
Malcolmiamaritima_lle14
Arabidopsisthaliana_lle16
Capsellagrandiflora*LOST
Boecherastricta*LOST
Lepidiumsativum_lle22
Diptychocarpusstrictus_lle3
Caricapapaya_lle14
Citrussinensis_lle4
Anacardiumoccidentale_lle15
Eucalyptusgrandis_lle4
Ceratophyllumdemersum_lle3
Nymphaeathermarum_lle5
Nymphaeacolorata_lle3
Euryaleferox_lle6
Acorusamericanus_lle2
Zosteramarina_lle3
Spirodelapolyrhiza*LOST
Dioscoreaalata_lle9
Asparagusofficinalis_lle6
Ananascomosus_lle4
Hordeumvulgare_lle42
Thinopyrumintermedium_lle64
Triticumaestivum_lle43
Triticumaestivum_lle7
Triticumaestivum_lle42
Thinopyrumintermedium_lle33
Triticumaestivum_lle100
Thinopyrumintermedium_lle1
Triticumaestivum_lle55
Thinopyrumintermedium_lle30
Triticumaestivum_lle81
Thinopyrumintermedium_lle45
Triticumaestivum_lle82
Thinopyrumintermedium_lle43
Triticumaestivum_lle96
Brachypodiumdistachyon*LOST
Oryzasativa_lle22
Panicumvirgatum_lle30
Panicumvirgatum_lle33
Setariaviridis_lle8
Alloteropsissemialata*LOST
Sorghumbicolor_lle9
Zeamays_lle49
Miscanthussinensis*LOST
Paspalumvaginatum*LOST
Oryzasativa_lle20
Alloteropsissemialata_lle7
Panicumvirgatum_lle15
Setariaviridis_lle12
Paspalumvaginatum_lle6
Miscanthussinensis_lle5
Miscanthussinensis_lle9
Zeamays*LOST
Sorghumbicolor_lle5
Brachypodiumdistachyon_lle10
Hordeumvulgare_lle31
Thinopyrumintermedium_lle40
Triticumaestivum_lle34
Oryzasativa_lle6
Alloteropsissemialata_lle3
Panicumvirgatum_lle16

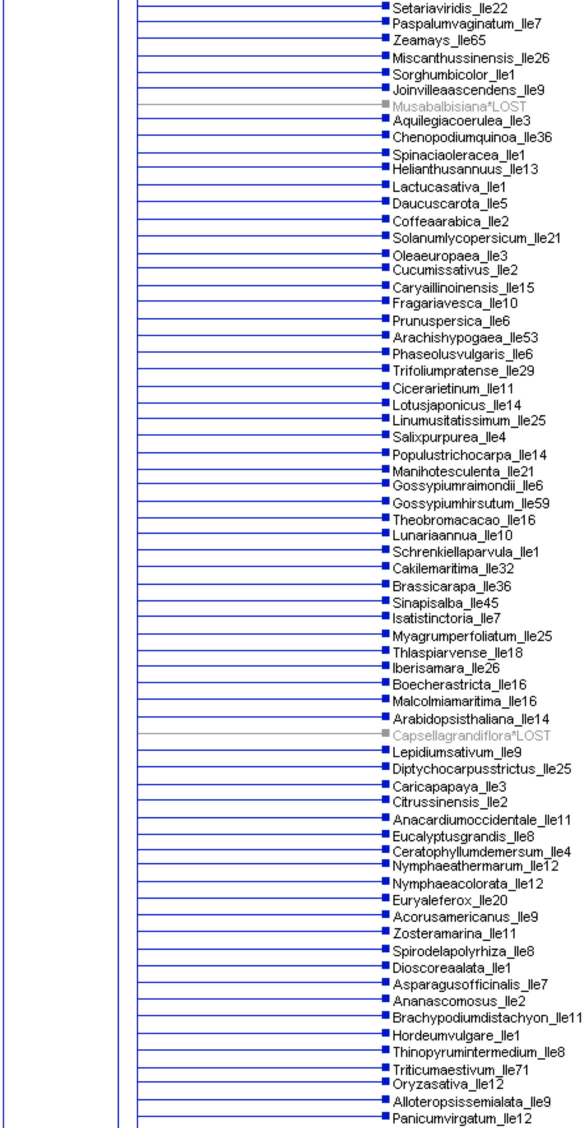
Setariaviridis_lle22
Paspalumvaginatum_lle7
Zeamays_lle65
Miscanthussinensis_lle26
Sorghumbicolor_lle1
Joinvilleaascendens_lle9
Musabalbisiana*LOST
Aquilegiacoerulea_lle3
Chenopodiumquinoa_lle36
Spinaciaoleracea_lle1
Helianthusannuus_lle13
Lactucasativa_lle1
Daucuscarota_lle5
Coffeaarabica_lle2
Solanumlycopersicum_lle21
Oleaeuropaea_lle3
Cucumissativus_lle2
Caryaillinoinensis_lle15
Fragariavesca_lle10
Prunuspersica_lle6
Arachishypogaea_lle53
Phaseolusvulgaris_lle6
Trifoliumpratense_lle29
Cicerarietinum_lle11
Lotusjaponicus_lle14
Linumusitatissimum_lle25
Salixpurpurea_lle4
Populustrichocarpa_lle14
Manihotesculenta_lle21
Gossypiumraimondii_lle6
Gossypiumhirsutum_lle59
Theobromacacao_lle16
Lunariaannua_lle10
Schrenkiellaparvula_lle1
Cakilemaritima_lle32
Brassicarapa_lle36
Sinapisalba_lle45
Isatistinctoria_lle7
Myagrumperfoliatum_lle25
Thlaspiarvense_lle18
Iberisamara_lle26
Boecherastricta_lle16
Malcolmiamaritima_lle16
Arabidopsisthaliana_lle14
Capsellagrandiflora*LOST
Lepidiumsativum_lle9
Diptychocarpusstrictus_lle25
Caricapapaya_lle3
Citrussinensis_lle2
Anacardiumoccidentale_lle11
Eucalyptusgrandis_lle8
Ceratophyllumdemersum_lle4
Nymphaeathermarum_lle12
Nymphaeacolorata_lle12
Euryaleferox_lle20
Acorusamericanus_lle9
Zosteramarina_lle11
Spirodelapolyrhiza_lle8
Dioscoreaalata_lle1
Asparagusofficinalis_lle7
Ananascomosus_lle2
Brachypodiumdistachyon_lle11
Hordeumvulgare_lle1
Thinopyrumintermedium_lle8
Triticumaestivum_lle71
Oryzasativa_lle12
Alloteropsissemialata_lle9
Panicumvirgatum_lle12

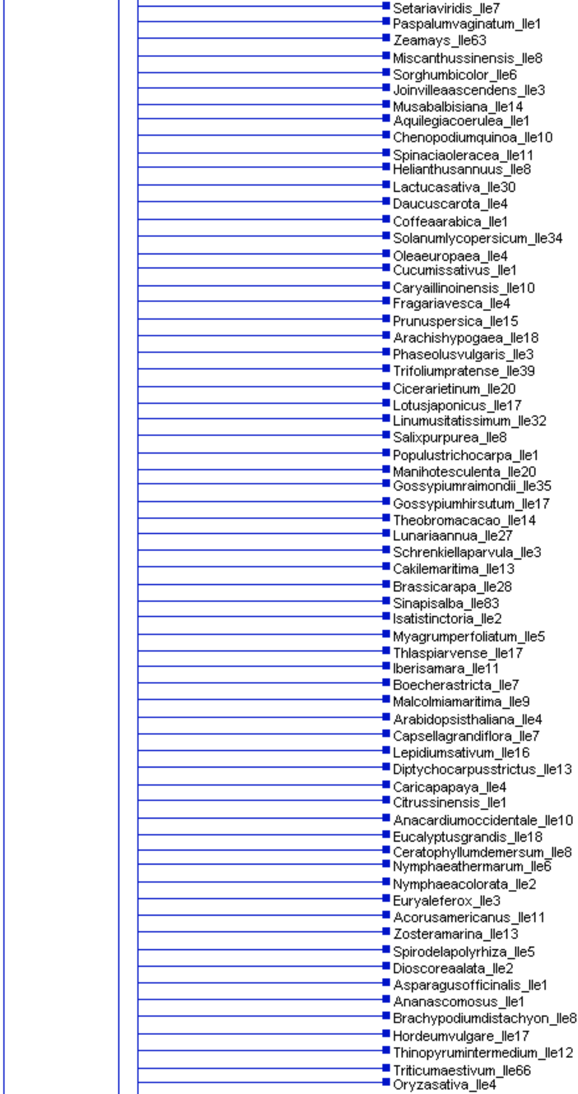
Setariaviridis_lle7
Paspalumvaginatum_lle1
Zeamays_lle63
Miscanthussinensis_lle8
Sorghumbicolor_lle6
Joinvilleaascendens_lle3
Musabalbisiana_lle14
Aquilegiacoerulea_lle1
Chenopodiumquinoa_lle10
Spinaciaoleracea_lle11
Helianthusannuus_lle8
Lactucasativa_lle30
Daucuscarota_lle4
Coffeaarabica_lle1
Solanumlycopersicum_lle34
Oleaeuropaea_lle4
Cucumissativus_lle1
Caryaillinoinensis_lle10
Fragariavesca_lle4
Prunuspersica_lle15
Arachishypogaea_lle18
Phaseolusvulgaris_lle3
Trifoliumpratense_lle39
Cicerarietinum_lle20
Lotusjaponicus_lle17
Linumusitatissimum_lle32
Salixpurpurea_lle8
Populustrichocarpa_lle1
Manihotesculenta_lle20
Gossypiumraimondii_lle35
Gossypiumhirsutum_lle17
Theobromacacao_lle14
Lunariaannua_lle27
Schrenkiellaparvula_lle3
Cakilemaritima_lle13
Brassicarapa_lle28
Sinapisalba_lle83
Isatistinctoria_lle2
Myagrumperfoliatum_lle5
Thlaspiarvense_lle17
Iberisamara_lle11
Boecherastricta_lle7
Malcolmiamaritima_lle9
Arabidopsisthaliana_lle4
Capsellagrandiflora_lle7
Lepidiumsativum_lle16
Diptychocarpusstrictus_lle13
Caricapapaya_lle4
Citrussinensis_lle1
Anacardiumoccidentale_lle10
Eucalyptusgrandis_lle18
Ceratophyllumdemersum_lle8
Nymphaeathermarum_lle6
Nymphaeacolorata_lle2
Euryaleferox_lle3
Acorusamericanus_lle11
Zosteramarina_lle13
Spirodelapolyrhiza_lle5
Dioscoreaalata_lle2
Asparagusofficinalis_lle1
Ananascomosus_lle1
Brachypodiumdistachyon_lle8
Hordeumvulgare_lle17
Thinopyrumintermedium_lle12
Triticumaestivum_lle66
Oryzasativa_lle4

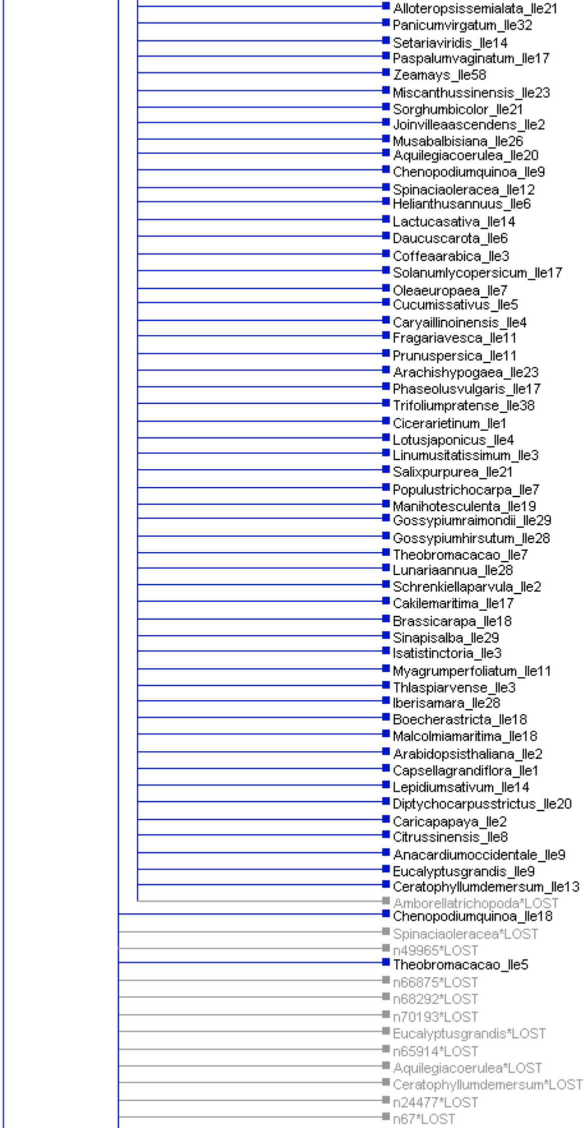
Alloteropsissemialata_Ile21
Panicumvirgatum_Ile32
Setariaviridis_Ile14
Paspalumvaginatum_Ile17
Zeamays_Ile58
Miscanthussinensis_Ile23
Sorghumbicolor_Ile21
Joinvilleaascendens_Ile2
Musabalbisiana_Ile26
Aquilegiacoerulea_Ile20
Chenopodiumquinoa_Ile9
Spinaciaoleracea_Ile12
Helianthusannuus_Ile6
Lactucasativa_Ile14
Daucuscarota_Ile6
Coffeaarabica_Ile3
Solanumlycopersicum_Ile17
Oleaeuropaea_Ile7
Cucumissativus_Ile5
Caryaillinoinensis_Ile4
Fragariavesca_Ile11
Prunuspersica_Ile11
Arachishypogaea_Ile23
Phaseolusvulgaris_Ile17
Trifoliumpratense_Ile38
Cicerarietinum_Ile1
Lotusjaponicus_Ile4
Linumusitatissimum_Ile3
Salixpurpurea_Ile21
Populustrichocarpa_Ile7
Manihotesculenta_Ile19
Gossypiumraimondii_Ile29
Gossypiumhirsutum_Ile28
Theobromacacao_Ile7
Lunariaannua_Ile28
Schrenkiellaparvula_Ile2
Cakilemaritima_Ile17
Brassicarapa_Ile18
Sinapisalba_Ile29
Isatistinctoria_Ile3
Myagrumperfoliatum_Ile11
Thlaspiarvense_Ile3
Iberisamara_Ile28
Boecherastricta_Ile18
Malcolmiamaritima_Ile18
Arabidopsisthaliana_Ile2
Capsellagrandiflora_Ile1
Lepidiumsativum_Ile14
Diptychocarpusstrictus_Ile20
Caricapapaya_Ile2
Citrussinensis_Ile8
Anacardiumoccidentale_Ile9
Eucalyptusgrandis_Ile9
Ceratophyllumdemersum_Ile13
Amborellatrichopoda*LOST
Chenopodiumquinoa_Ile18
Spinaciaoleracea*LOST
n49965*LOST
Theobromacacao_Ile5
n66875*LOST
n68292*LOST
n70193*LOST
Eucalyptusgrandis*LOST
n65914*LOST
Aquilegiacoerulea*LOST
Ceratophyllumdemersum*LOST
n24477*LOST
n67*LOST

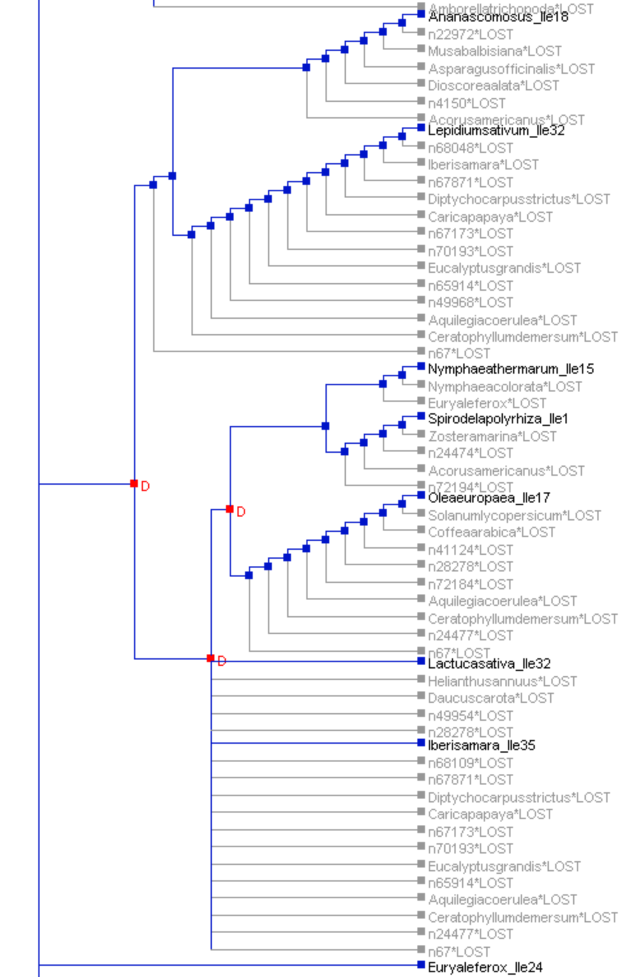
Amborellatrichopoda*LOST
Ananascomosus_Ile18
n22972*LOST
Musabalbisiana*LOST
Asparagusofficinalis*LOST
Dioscoreaalata*LOST
n4150*LOST
Acorusamericanus*LOST
Lepidiumsativum_Ile32
n68048*LOST
Iberisamara*LOST
n67871*LOST
Diptychocarpusstrictus*LOST
Caricapapaya*LOST
n67173*LOST
n70193*LOST
Eucalyptusgrandis*LOST
n65914*LOST
n49968*LOST
Aquilegiacoerulea*LOST
Ceratophyllumdemersum*LOST
n67*LOST
D
Nymphaeathermarum_Ile15
Nymphaeacolorata*LOST
Euryaleferox*LOST
Spirodelapolyrhiza_Ile1
Zosteramarina*LOST
n24474*LOST
Acorusamericanus*LOST
n72194*LOST
D
Oleaeuropaea_Ile17
Solanumlycopersicum*LOST
Coffeaarabica*LOST
n41124*LOST
n28278*LOST
n72184*LOST
Aquilegiacoerulea*LOST
Ceratophyllumdemersum*LOST
n24477*LOST
n67*LOST
D
Lactucasativa_Ile32
Helianthusannuus*LOST
Daucuscarota*LOST
n49954*LOST
n28278*LOST
Iberisamara_Ile35
n68109*LOST
n67871*LOST
Diptychocarpusstrictus*LOST
Caricapapaya*LOST
n67173*LOST
n70193*LOST
Eucalyptusgrandis*LOST
n65914*LOST
Aquilegiacoerulea*LOST
Ceratophyllumdemersum*LOST
n24477*LOST
n67*LOST
Euryaleferox_Ile24

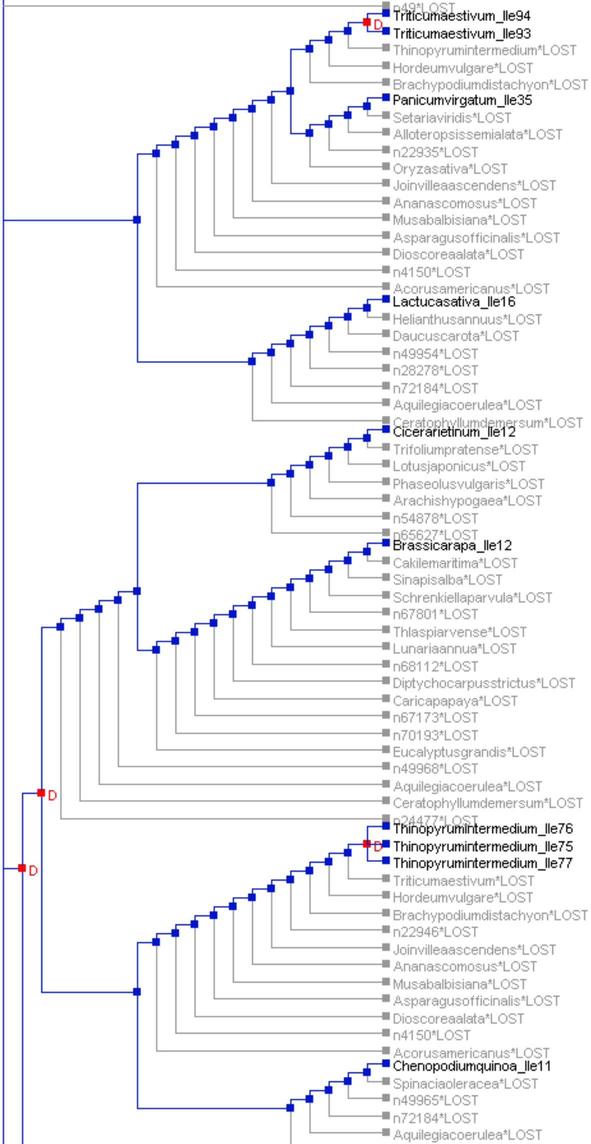

n49*LOST
Triticumaestivum_lle94
D
Triticumaestivum_lle93
Thinopyrumintermedium*LOST
Hordeumvulgare*LOST
Brachypodiumdistachyon*LOST
Panicumvirgatum_lle35
Setariaviridis*LOST
Alloteropsissemialata*LOST
n22935*LOST
Oryzasativa*LOST
Joinvilleaascendens*LOST
Ananascomosus*LOST
Musabalbisiana*LOST
Asparagusofficinalis*LOST
Dioscoreaalata*LOST
n4150*LOST
Acorusamericanus*LOST
Lactucasativa_lle16
Helianthusannuus*LOST
Daucuscarota*LOST
n49954*LOST
n28278*LOST
n72184*LOST
Aquilegiacoerulea*LOST
Ceratophyllumdemersum*LOST
Cicerarietinum_lle12
Trifoliumpratense*LOST
Lotusjaponicus*LOST
Phaseolusvulgaris*LOST
Arachishypogaea*LOST
n54878*LOST
n65627*LOST
Brassicarapa_lle12
Cakilemaritima*LOST
Sinapisalba*LOST
Schrenkiellaparvula*LOST
n67801*LOST
Thlaspiarvense*LOST
Lunariaannua*LOST
n68112*LOST
Diptychocarpusstrictus*LOST
Caricapapaya*LOST
n67173*LOST
n70193*LOST
Eucalyptusgrandis*LOST
n49968*LOST
Aquilegiacoerulea*LOST
Ceratophyllumdemersum*LOST
D
n24477*LOST
Thinopyrumintermedium_lle76
D
Thinopyrumintermedium_lle75
Thinopyrumintermedium_lle77
D
Triticumaestivum*LOST
Hordeumvulgare*LOST
Brachypodiumdistachyon*LOST
n22946*LOST
Joinvilleaascendens*LOST
Ananascomosus*LOST
Musabalbisiana*LOST
Asparagusofficinalis*LOST
Dioscoreaalata*LOST
n4150*LOST
Acorusamericanus*LOST
Chenopodiumquinoa_lle11
Spinaciaoleracea*LOST
n49965*LOST
n72184*LOST
Aquilegiacoerulea*LOST

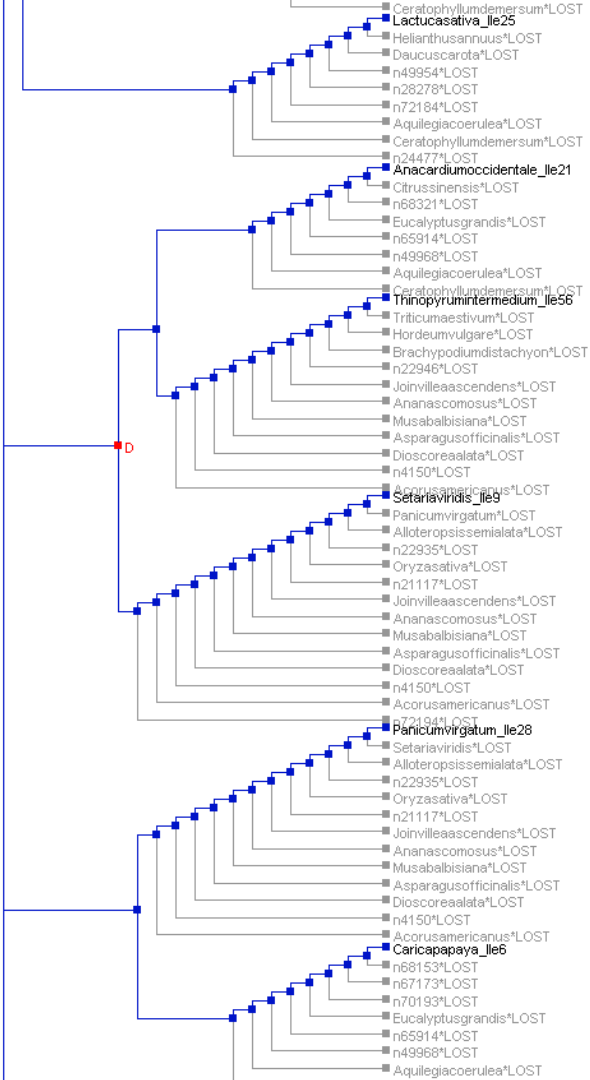
Ceratophyllumdemersum*LOST
Lactucasativa_Ile25
Helianthusannuus*LOST
Daucuscarota*LOST
n49954*LOST
n28278*LOST
n72184*LOST
Aquilegiacoerulea*LOST
Ceratophyllumdemersum*LOST
n24477*LOST
Anacardiumoccidentale_Ile21
Citrussinensis*LOST
n68321*LOST
Eucalyptusgrandis*LOST
n65914*LOST
n49968*LOST
Aquilegiacoerulea*LOST
Ceratophyllumdemersum*LOST
Thinopyrumintermedium_Ile56
Triticumaestivum*LOST
Hordeumvulgare*LOST
Brachypodiumdistachyon*LOST
n22946*LOST
Joinvilleaascendens*LOST
Ananascomosus*LOST
Musabalbisiana*LOST
Asparagusofficinalis*LOST
Dioscoreaalata*LOST
n4150*LOST
Acorusamericanus*LOST
D
Setariaviridis_Ile9
Panicumvirgatum*LOST
Alloteropsissemialata*LOST
n22935*LOST
Oryzasativa*LOST
n21117*LOST
Joinvilleaascendens*LOST
Ananascomosus*LOST
Musabalbisiana*LOST
Asparagusofficinalis*LOST
Dioscoreaalata*LOST
n4150*LOST
Acorusamericanus*LOST
n72194*LOST
Panicumvirgatum_Ile28
Setariaviridis*LOST
Alloteropsissemialata*LOST
n22935*LOST
Oryzasativa*LOST
n21117*LOST
Joinvilleaascendens*LOST
Ananascomosus*LOST
Musabalbisiana*LOST
Asparagusofficinalis*LOST
Dioscoreaalata*LOST
n4150*LOST
Acorusamericanus*LOST
Caricapapaya_Ile6
n68153*LOST
n67173*LOST
n70193*LOST
Eucalyptusgrandis*LOST
n65914*LOST
n49968*LOST
Aquilegiacoerulea*LOST

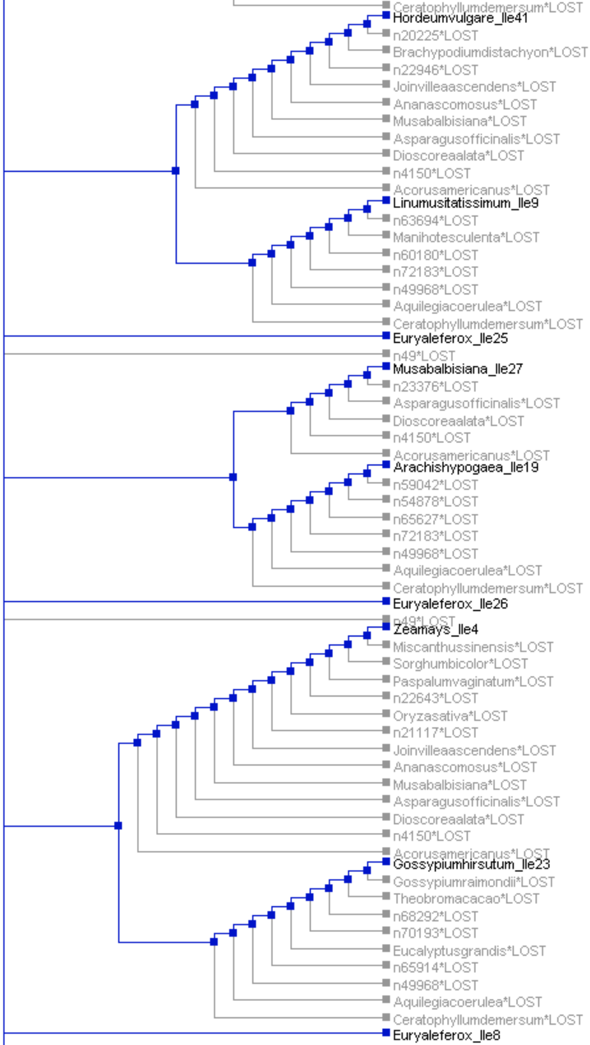
Ceratophyllumdemersum*LOST
Hordeumvulgare_lle41
n20225*LOST
Brachypodiumdistachyon*LOST
n22946*LOST
Joinvilleaascendens*LOST
Ananascomosus*LOST
Musabalbisiana*LOST
Asparagusofficinalis*LOST
Dioscoreaalata*LOST
n4150*LOST
Acorusamericanus*LOST
Linumusitatissimum_lle9
n63694*LOST
Manihotesculenta*LOST
n60180*LOST
n72183*LOST
n49968*LOST
Aquilegiacoerulea*LOST
Ceratophyllumdemersum*LOST
Euryaleferox_lle25
n49*LOST
Musabalbisiana_lle27
n23376*LOST
Asparagusofficinalis*LOST
Dioscoreaalata*LOST
n4150*LOST
Acorusamericanus*LOST
Arachishypogaea_lle19
n59042*LOST
n54878*LOST
n65627*LOST
n72183*LOST
n49968*LOST
Aquilegiacoerulea*LOST
Ceratophyllumdemersum*LOST
Euryaleferox_lle26
n49*LOST
Zeamays_lle4
Miscanthussinensis*LOST
Sorghumbicolor*LOST
Paspalumvaginatum*LOST
n22643*LOST
Oryzasativa*LOST
n21117*LOST
Joinvilleaascendens*LOST
Ananascomosus*LOST
Musabalbisiana*LOST
Asparagusofficinalis*LOST
Dioscoreaalata*LOST
n4150*LOST
Acorusamericanus*LOST
Gossypiumhirsutum_lle23
Gossypiumraimondii*LOST
Theobromacacao*LOST
n68292*LOST
n70193*LOST
Eucalyptusgrandis*LOST
n65914*LOST
n49968*LOST
Aquilegiacoerulea*LOST
Ceratophyllumdemersum*LOST
Euryaleferox_lle8

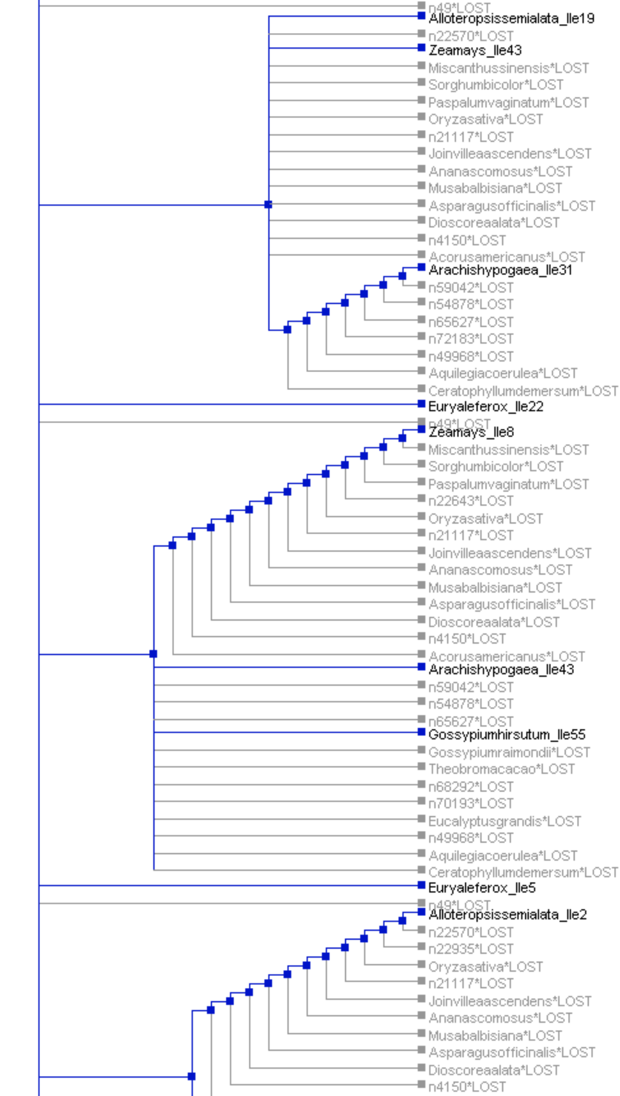
n49*LOST
Alloteropsissemialata_lle19
n22570*LOST
Zeamays_lle43
Miscanthussinensis*LOST
Sorghumbicolor*LOST
Paspalumvaginatum*LOST
Oryzasativa*LOST
n21117*LOST
Joinvilleaascendens*LOST
Ananascomosus*LOST
Musabalbisiana*LOST
Asparagusofficinalis*LOST
Dioscoreaalata*LOST
n4150*LOST
Acorusamericanus*LOST
Arachishypogaea_lle31
n59042*LOST
n54878*LOST
n65627*LOST
n72183*LOST
n49968*LOST
Aquilegiacoerulea*LOST
Ceratophyllumdemersum*LOST
Euryaleferox_lle22
n49*LOST
Zeamays_lle8
Miscanthussinensis*LOST
Sorghumbicolor*LOST
Paspalumvaginatum*LOST
n22643*LOST
Oryzasativa*LOST
n21117*LOST
Joinvilleaascendens*LOST
Ananascomosus*LOST
Musabalbisiana*LOST
Asparagusofficinalis*LOST
Dioscoreaalata*LOST
n4150*LOST
Acorusamericanus*LOST
Arachishypogaea_lle43
n59042*LOST
n54878*LOST
n65627*LOST
Gossypiumhirsutum_lle55
Gossypiumraimondii*LOST
Theobromacacao*LOST
n68292*LOST
n70193*LOST
Eucalyptusgrandis*LOST
n49968*LOST
Aquilegiacoerulea*LOST
Ceratophyllumdemersum*LOST
Euryaleferox_lle5
n49*LOST
Alloteropsissemialata_lle2
n22570*LOST
n22935*LOST
Oryzasativa*LOST
n21117*LOST
Joinvilleaascendens*LOST
Ananascomosus*LOST
Musabalbisiana*LOST
Asparagusofficinalis*LOST
Dioscoreaalata*LOST
n4150*LOST

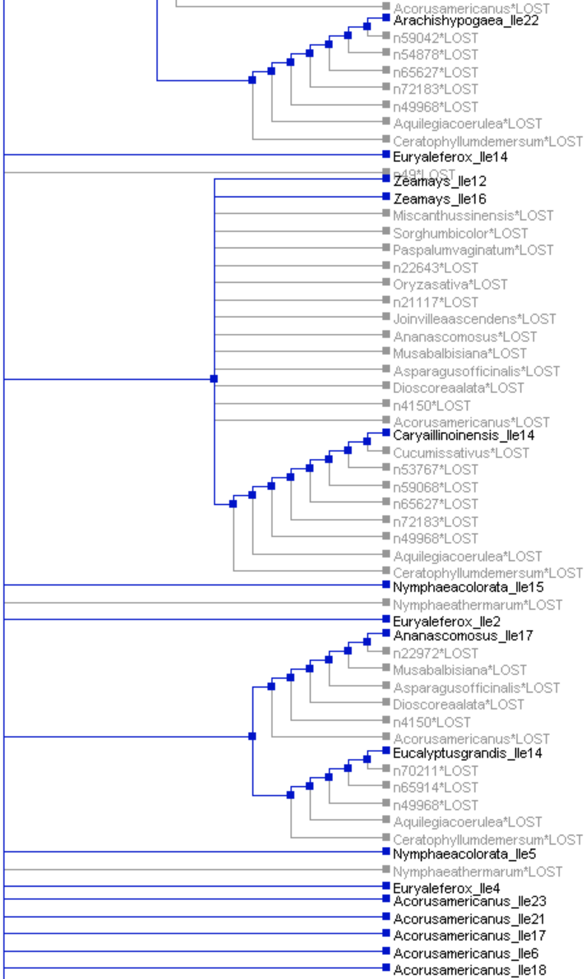

Acorusamericanus*LOST
Arachishypogaea_lle22
n59042*LOST
n54878*LOST
n65627*LOST
n72183*LOST
n49968*LOST
Aquilegiacoerulea*LOST
Ceratophyllumdemersum*LOST
Euryaleferox_lle14
Zeamays_lle12
Zeamays_lle16
Miscanthussinensis*LOST
Sorghumbicolor*LOST
Paspalumvaginatum*LOST
n22643*LOST
Oryzasativa*LOST
n21117*LOST
Joinvilleaascendens*LOST
Ananascomosus*LOST
Musabalbisiana*LOST
Asparagusofficinalis*LOST
Dioscoreaalata*LOST
n4150*LOST
Acorusamericanus*LOST
Caryaillinoinensis_lle14
Cucumissativus*LOST
n53767*LOST
n59068*LOST
n65627*LOST
n72183*LOST
n49968*LOST
Aquilegiacoerulea*LOST
Ceratophyllumdemersum*LOST
Nymphaeacolorata_lle15
Nymphaeathermarum*LOST
Euryaleferox_lle2
Ananascomosus_lle17
n22972*LOST
Musabalbisiana*LOST
Asparagusofficinalis*LOST
Dioscoreaalata*LOST
n4150*LOST
Acorusamericanus*LOST
Eucalyptusgrandis_lle14
n70211*LOST
n65914*LOST
n49968*LOST
Aquilegiacoerulea*LOST
Ceratophyllumdemersum*LOST
Nymphaeacolorata_lle5
Nymphaeathermarum*LOST
Euryaleferox_lle4
Acorusamericanus_lle23
Acorusamericanus_lle21
Acorusamericanus_lle17
Acorusamericanus_lle6
Acorusamericanus_lle18

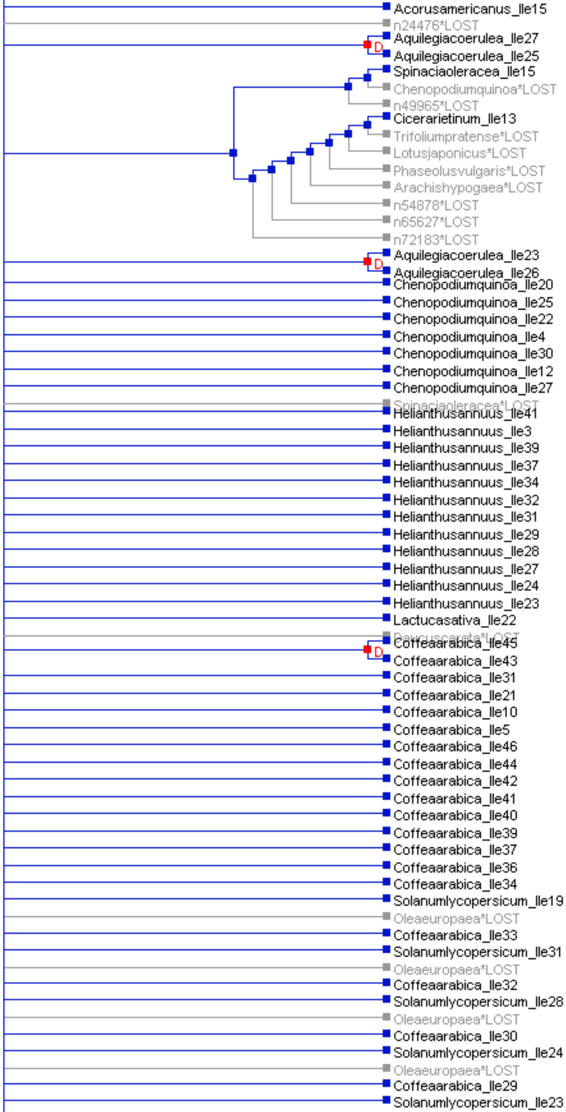
Acorusamericanus_Ile15
n24476*LOST
D
Aquilegiacoerulea_Ile27
Aquilegiacoerulea_Ile25
Spinaciaoleracea_Ile15
Chenopodiumquinoa*LOST
n49965*LOST
Cicerarietinum_Ile13
Trifoliumpratense*LOST
Lotusjaponicus*LOST
Phaseolusvulgaris*LOST
Arachishypogaea*LOST
n54878*LOST
n65627*LOST
n72183*LOST
D
Aquilegiacoerulea_Ile23
Aquilegiacoerulea_Ile26
Chenopodiumquinoa_Ile20
Chenopodiumquinoa_Ile25
Chenopodiumquinoa_Ile22
Chenopodiumquinoa_Ile4
Chenopodiumquinoa_Ile30
Chenopodiumquinoa_Ile12
Chenopodiumquinoa_Ile27
Spinaciaoleracea*LOST
Helianthusannuus_Ile41
Helianthusannuus_Ile3
Helianthusannuus_Ile39
Helianthusannuus_Ile37
Helianthusannuus_Ile34
Helianthusannuus_Ile32
Helianthusannuus_Ile31
Helianthusannuus_Ile29
Helianthusannuus_Ile28
Helianthusannuus_Ile27
Helianthusannuus_Ile24
Helianthusannuus_Ile23
Lactucasativa_Ile22
Daucuscarota*LOST
D
Coffeaarabica_Ile45
Coffeaarabica_Ile43
Coffeaarabica_Ile31
Coffeaarabica_Ile21
Coffeaarabica_Ile10
Coffeaarabica_Ile5
Coffeaarabica_Ile46
Coffeaarabica_Ile44
Coffeaarabica_Ile42
Coffeaarabica_Ile41
Coffeaarabica_Ile40
Coffeaarabica_Ile39
Coffeaarabica_Ile37
Coffeaarabica_Ile36
Coffeaarabica_Ile34
Solanumlycopersicum_Ile19
Oleaeuropaea*LOST
Coffeaarabica_Ile33
Solanumlycopersicum_Ile31
Oleaeuropaea*LOST
Coffeaarabica_Ile32
Solanumlycopersicum_Ile28
Oleaeuropaea*LOST
Coffeaarabica_Ile30
Solanumlycopersicum_Ile24
Oleaeuropaea*LOST
Coffeaarabica_Ile29
Solanumlycopersicum_Ile23

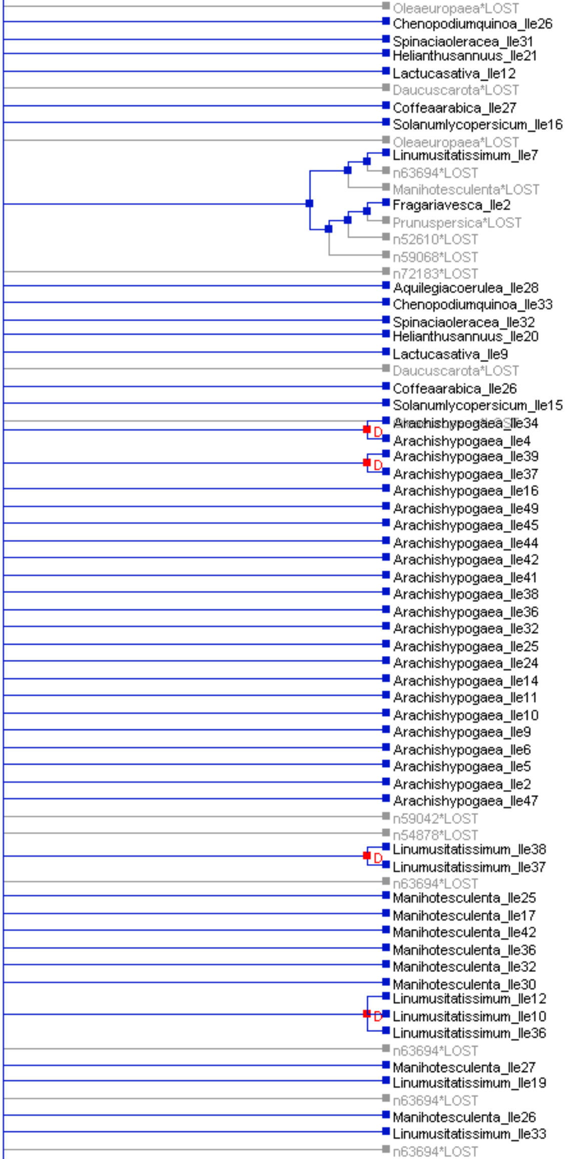

Oleaeuropaea*LOST
Chenopodiumquinoa_lle26
Spinaciaoleracea_lle31
Helianthusannuus_lle21
Lactucasativa_lle12
Daucuscarota*LOST
Coffeaarabica_lle27
Solanumlycopersicum_lle16
Oleaeuropaea*LOST
Linumusitatissimum_lle7
n63694*LOST
Manihotesculenta*LOST
Fragariavesca_lle2
Prunuspersica*LOST
n52610*LOST
n59068*LOST
n72183*LOST
Aquilegiacoerulea_lle28
Chenopodiumquinoa_lle33
Spinaciaoleracea_lle32
Helianthusannuus_lle20
Lactucasativa_lle9
Daucuscarota*LOST
Coffeaarabica_lle26
Solanumlycopersicum_lle15
Arachishypogaea_lle34
D
Arachishypogaea_lle4
Arachishypogaea_lle39
D
Arachishypogaea_lle37
Arachishypogaea_lle16
Arachishypogaea_lle49
Arachishypogaea_lle45
Arachishypogaea_lle44
Arachishypogaea_lle42
Arachishypogaea_lle41
Arachishypogaea_lle38
Arachishypogaea_lle36
Arachishypogaea_lle32
Arachishypogaea_lle25
Arachishypogaea_lle24
Arachishypogaea_lle14
Arachishypogaea_lle11
Arachishypogaea_lle10
Arachishypogaea_lle9
Arachishypogaea_lle6
Arachishypogaea_lle5
Arachishypogaea_lle2
Arachishypogaea_lle47
n59042*LOST
n54878*LOST
Linumusitatissimum_lle38
D
Linumusitatissimum_lle37
n63694*LOST
Manihotesculenta_lle25
Manihotesculenta_lle17
Manihotesculenta_lle42
Manihotesculenta_lle36
Manihotesculenta_lle32
Manihotesculenta_lle30
Linumusitatissimum_lle12
D
Linumusitatissimum_lle10
Linumusitatissimum_lle36
n63694*LOST
Manihotesculenta_lle27
Linumusitatissimum_lle19
n63694*LOST
Manihotesculenta_lle26
Linumusitatissimum_lle33
n63694*LOST

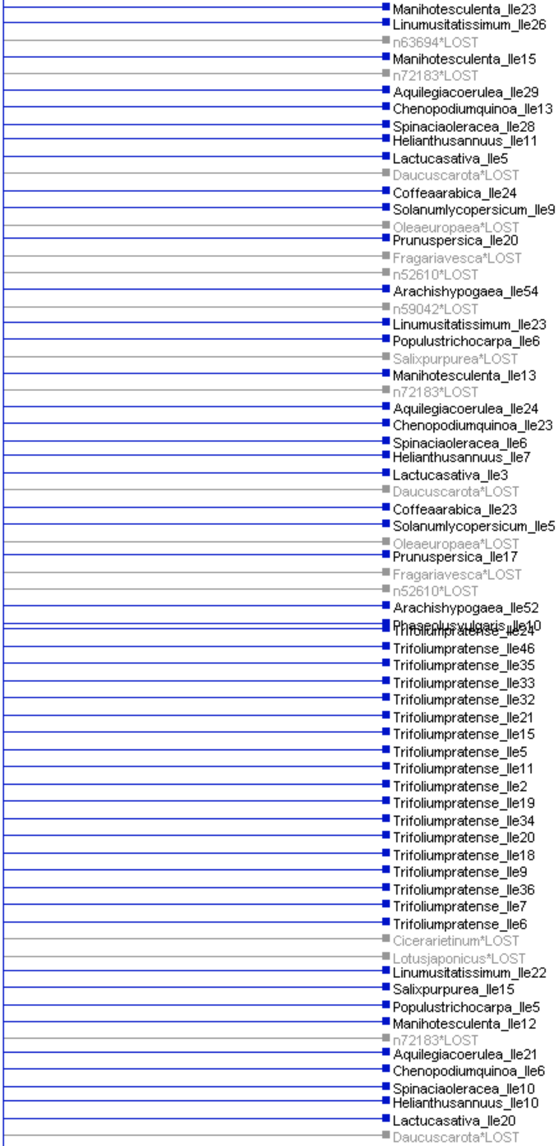
Manihotesculenta_lle23
Linumusitatissimum_lle26
n63694*LOST
Manihotesculenta_lle15
n72183*LOST
Aquilegiacoerulea_lle29
Chenopodiumquinoa_lle13
Spinaciaoleracea_lle28
Helianthusannuus_lle11
Lactucasativa_lle5
Daucuscarota*LOST
Coffeaarabica_lle24
Solanumlycopersicum_lle9
Oleaeuropaea*LOST
Prunuspersica_lle20
Fragariavesca*LOST
n52610*LOST
Arachishypogaea_lle54
n59042*LOST
Linumusitatissimum_lle23
Populustrichocarpa_lle6
Salixpurpurea*LOST
Manihotesculenta_lle13
n72183*LOST
Aquilegiacoerulea_lle24
Chenopodiumquinoa_lle23
Spinaciaoleracea_lle6
Helianthusannuus_lle7
Lactucasativa_lle3
Daucuscarota*LOST
Coffeaarabica_lle23
Solanumlycopersicum_lle5
Oleaeuropaea*LOST
Prunuspersica_lle17
Fragariavesca*LOST
n52610*LOST
Arachishypogaea_lle52
Phaseolusvulgaris_lle10
Trifoliumpratense_lle24
Trifoliumpratense_lle46
Trifoliumpratense_lle35
Trifoliumpratense_lle33
Trifoliumpratense_lle32
Trifoliumpratense_lle21
Trifoliumpratense_lle15
Trifoliumpratense_lle5
Trifoliumpratense_lle11
Trifoliumpratense_lle2
Trifoliumpratense_lle19
Trifoliumpratense_lle34
Trifoliumpratense_lle20
Trifoliumpratense_lle18
Trifoliumpratense_lle9
Trifoliumpratense_lle36
Trifoliumpratense_lle7
Trifoliumpratense_lle6
Cicerarietinum*LOST
Lotusjaponicus*LOST
Linumusitatissimum_lle22
Salixpurpurea_lle15
Populustrichocarpa_lle5
Manihotesculenta_lle12
n72183*LOST
Aquilegiacoerulea_lle21
Chenopodiumquinoa_lle6
Spinaciaoleracea_lle10
Helianthusannuus_lle10
Lactucasativa_lle20
Daucuscarota*LOST

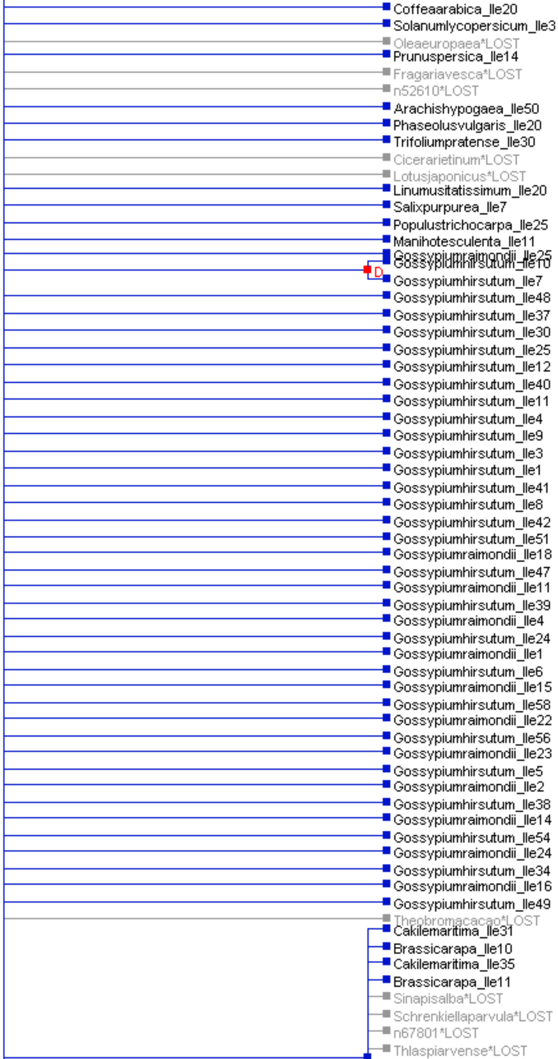
Coffeaarabica_lle20
Solanumlycopersicum_lle3
Oleaeuropaea*LOST
Prunuspersica_lle14
Fragariavesca*LOST
n52610*LOST
Arachishypogaea_lle50
Phaseolusvulgaris_lle20
Trifoliumpratense_lle30
Cicerarietinum*LOST
Lotusjaponicus*LOST
Linumusitatissimum_lle20
Salixpurpurea_lle7
Populustrichocarpa_lle25
Manihotesculenta_lle11
Gossypiumraimondii_lle25
Gossypiumhirsutum_lle10
D
Gossypiumhirsutum_lle7
Gossypiumhirsutum_lle48
Gossypiumhirsutum_lle37
Gossypiumhirsutum_lle30
Gossypiumhirsutum_lle25
Gossypiumhirsutum_lle12
Gossypiumhirsutum_lle40
Gossypiumhirsutum_lle11
Gossypiumhirsutum_lle4
Gossypiumhirsutum_lle9
Gossypiumhirsutum_lle3
Gossypiumhirsutum_lle1
Gossypiumhirsutum_lle41
Gossypiumhirsutum_lle8
Gossypiumhirsutum_lle42
Gossypiumhirsutum_lle51
Gossypiumraimondii_lle18
Gossypiumhirsutum_lle47
Gossypiumraimondii_lle11
Gossypiumhirsutum_lle39
Gossypiumraimondii_lle4
Gossypiumhirsutum_lle24
Gossypiumraimondii_lle1
Gossypiumhirsutum_lle6
Gossypiumraimondii_lle15
Gossypiumhirsutum_lle58
Gossypiumraimondii_lle22
Gossypiumhirsutum_lle56
Gossypiumraimondii_lle23
Gossypiumhirsutum_lle5
Gossypiumraimondii_lle2
Gossypiumhirsutum_lle38
Gossypiumraimondii_lle14
Gossypiumhirsutum_lle54
Gossypiumraimondii_lle24
Gossypiumhirsutum_lle34
Gossypiumraimondii_lle16
Gossypiumhirsutum_lle49
Theobromacacao*LOST
Cakilemaritima_lle31
Brassicarapa_lle10
Cakilemaritima_lle35
Brassicarapa_lle11
Sinapisalba*LOST
Schrenkiellaparvula*LOST
n67801*LOST
Thlaspiarvense*LOST

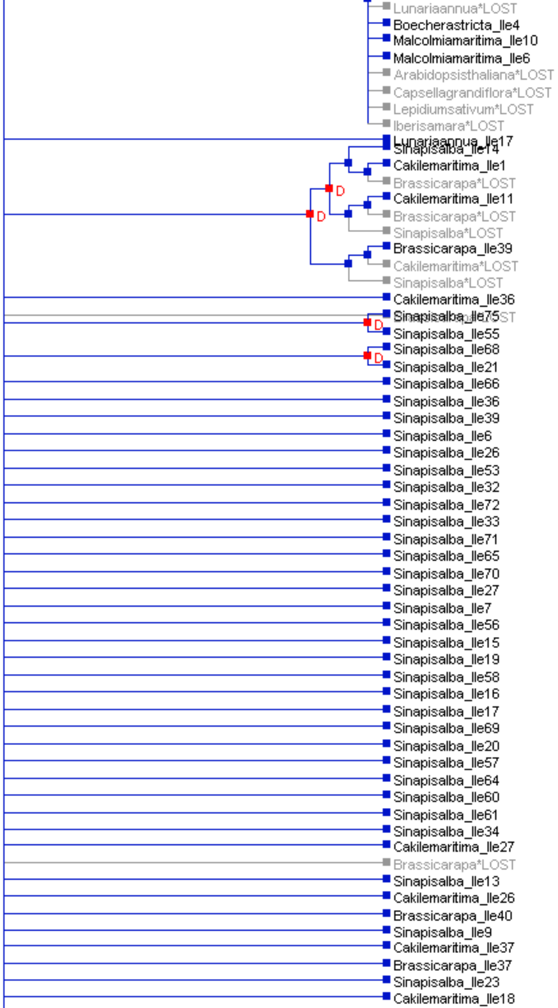
Lunariaannua*LOST
Boecherastricta_lle4
Malcolmiamaritima_lle10
Malcolmiamaritima_lle6
Arabidopsisthaliana*LOST
Capsellagrandiflora*LOST
Lepidiumsativum*LOST
Iberisamara*LOST
Lunariaannua_lle17
Sinapisalba_lle14
Cakilemaritima_lle1
Brassicarapa*LOST
D
D
Cakilemaritima_lle11
Brassicarapa*LOST
Sinapisalba*LOST
Brassicarapa_lle39
Cakilemaritima*LOST
Sinapisalba*LOST
Cakilemaritima_lle36
Sinapisalba_lle75
D
Sinapisalba_lle55
Sinapisalba_lle68
D
Sinapisalba_lle21
Sinapisalba_lle66
Sinapisalba_lle36
Sinapisalba_lle39
Sinapisalba_lle6
Sinapisalba_lle26
Sinapisalba_lle53
Sinapisalba_lle32
Sinapisalba_lle72
Sinapisalba_lle33
Sinapisalba_lle71
Sinapisalba_lle65
Sinapisalba_lle70
Sinapisalba_lle27
Sinapisalba_lle7
Sinapisalba_lle56
Sinapisalba_lle15
Sinapisalba_lle19
Sinapisalba_lle58
Sinapisalba_lle16
Sinapisalba_lle17
Sinapisalba_lle69
Sinapisalba_lle20
Sinapisalba_lle57
Sinapisalba_lle64
Sinapisalba_lle60
Sinapisalba_lle61
Sinapisalba_lle34
Cakilemaritima_lle27
Brassicarapa*LOST
Sinapisalba_lle13
Cakilemaritima_lle26
Brassicarapa_lle40
Sinapisalba_lle9
Cakilemaritima_lle37
Brassicarapa_lle37
Sinapisalba_lle23
Cakilemaritima_lle18

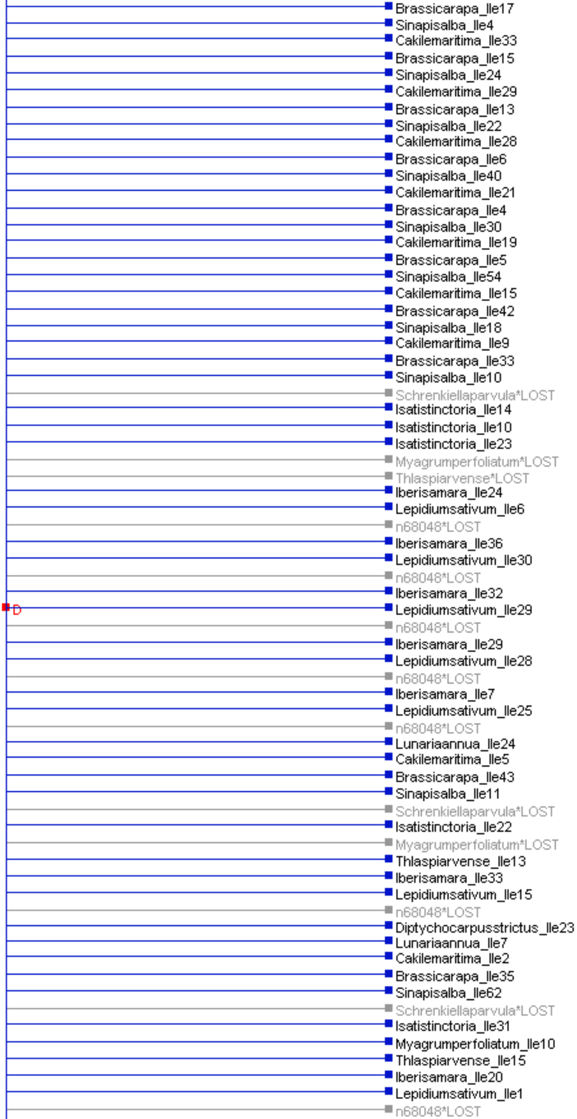
Brassicarapa_lle17
Sinapisalba_lle4
Cakilemaritima_lle33
Brassicarapa_lle15
Sinapisalba_lle24
Cakilemaritima_lle29
Brassicarapa_lle13
Sinapisalba_lle22
Cakilemaritima_lle28
Brassicarapa_lle6
Sinapisalba_lle40
Cakilemaritima_lle21
Brassicarapa_lle4
Sinapisalba_lle30
Cakilemaritima_lle19
Brassicarapa_lle5
Sinapisalba_lle54
Cakilemaritima_lle15
Brassicarapa_lle42
Sinapisalba_lle18
Cakilemaritima_lle9
Brassicarapa_lle33
Sinapisalba_lle10
Schrenkiellaparvula*LOST
Isatistinctoria_lle14
Isatistinctoria_lle10
Isatistinctoria_lle23
Myagrumperfoliatum*LOST
Thlaspiarvense*LOST
Iberisamara_lle24
Lepidiumsativum_lle6
n68048*LOST
Iberisamara_lle36
Lepidiumsativum_lle30
n68048*LOST
Iberisamara_lle32
D
Lepidiumsativum_lle29
n68048*LOST
Iberisamara_lle29
Lepidiumsativum_lle28
n68048*LOST
Iberisamara_lle7
Lepidiumsativum_lle25
n68048*LOST
Lunariaannua_lle24
Cakilemaritima_lle5
Brassicarapa_lle43
Sinapisalba_lle11
Schrenkiellaparvula*LOST
Isatistinctoria_lle22
Myagrumperfoliatum*LOST
Thlaspiarvense_lle13
Iberisamara_lle33
Lepidiumsativum_lle15
n68048*LOST
Diptychocarpusstrictus_lle23
Lunariaannua_lle7
Cakilemaritima_lle2
Brassicarapa_lle35
Sinapisalba_lle62
Schrenkiellaparvula*LOST
Isatistinctoria_lle31
Myagrumperfoliatum_lle10
Thlaspiarvense_lle15
Iberisamara_lle20
Lepidiumsativum_lle1
n68048*LOST

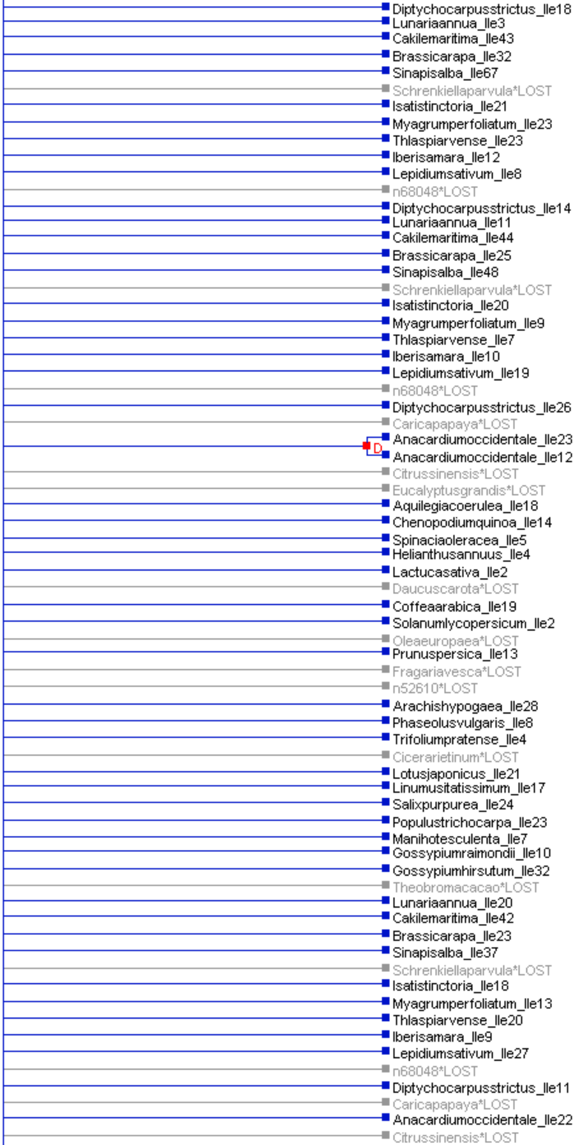
Diptychocarpusstrictus_lle18
Lunariaannua_lle3
Cakilemaritima_lle43
Brassicarapa_lle32
Sinapisalba_lle67
Schrenkiellaparvula*LOST
Isatistinctoria_lle21
Myagrumperfoliatum_lle23
Thlaspiarvense_lle23
Iberisamara_lle12
Lepidiumsativum_lle8
n68048*LOST
Diptychocarpusstrictus_lle14
Lunariaannua_lle11
Cakilemaritima_lle44
Brassicarapa_lle25
Sinapisalba_lle48
Schrenkiellaparvula*LOST
Isatistinctoria_lle20
Myagrumperfoliatum_lle9
Thlaspiarvense_lle7
Iberisamara_lle10
Lepidiumsativum_lle19
n68048*LOST
Diptychocarpusstrictus_lle26
Caricapapaya*LOST
D
Anacardiumoccidentale_lle23
Anacardiumoccidentale_lle12
Citrussinensis*LOST
Eucalyptusgrandis*LOST
Aquilegiacoerulea_lle18
Chenopodiumquinoa_lle14
Spinaciaoleracea_lle5
Helianthusannuus_lle4
Lactucasativa_lle2
Daucuscarota*LOST
Coffeaarabica_lle19
Solanumlycopersicum_lle2
Oleaeuropaea*LOST
Prunuspersica_lle13
Fragariavesca*LOST
n52610*LOST
Arachishypogaea_lle28
Phaseolusvulgaris_lle8
Trifoliumpratense_lle4
Cicerarietinum*LOST
Lotusjaponicus_lle21
Linumusitatissimum_lle17
Salixpurpurea_lle24
Populustrichocarpa_lle23
Manihotesculenta_lle7
Gossypiumraimondii_lle10
Gossypiumhirsutum_lle32
Theobromacacao*LOST
Lunariaannua_lle20
Cakilemaritima_lle42
Brassicarapa_lle23
Sinapisalba_lle37
Schrenkiellaparvula*LOST
Isatistinctoria_lle18
Myagrumperfoliatum_lle13
Thlaspiarvense_lle20
Iberisamara_lle9
Lepidiumsativum_lle27
n68048*LOST
Diptychocarpusstrictus_lle11
Caricapapaya*LOST
Anacardiumoccidentale_lle22
Citrussinensis*LOST

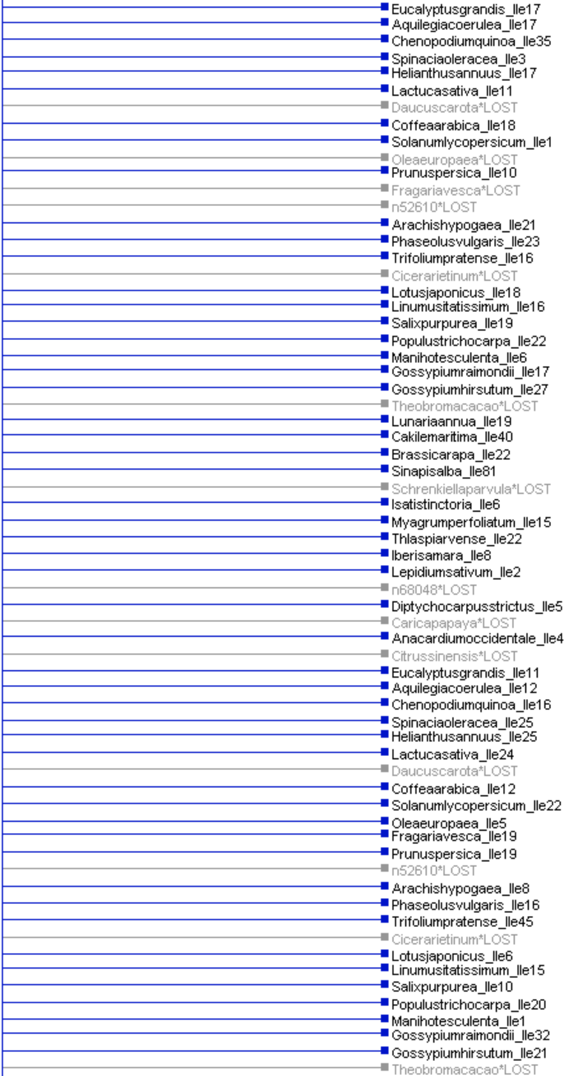

Eucalyptusgrandis_lle17
Aquilegiacoerulea_lle17
Chenopodiumquinoa_lle35
Spinaciaoleracea_lle3
Helianthusannuus_lle17
Lactucasativa_lle11
Daucuscarota*LOST
Coffeaarabica_lle18
Solanumlycopersicum_lle1
Oleaeuropaea*LOST
Prunuspersica_lle10
Fragariavesca*LOST
n52610*LOST
Arachishypogaea_lle21
Phaseolusvulgaris_lle23
Trifoliumpratense_lle16
Cicerarietinum*LOST
Lotusjaponicus_lle18
Linumusitatissimum_lle16
Salixpurpurea_lle19
Populustrichocarpa_lle22
Manihotesculenta_lle6
Gossypiumraimondii_lle17
Gossypiumhirsutum_lle27
Theobromacacao*LOST
Lunariaannua_lle19
Cakilemaritima_lle40
Brassicarapa_lle22
Sinapisalba_lle81
Schrenkiellaparvula*LOST
Isatistinctoria_lle6
Myagrumperfoliatum_lle15
Thlaspiarvense_lle22
Iberisamara_lle8
Lepidiumsativum_lle2
n68048*LOST
Diptychocarpusstrictus_lle5
Caricapapaya*LOST
Anacardiumoccidentale_lle4
Citrussinensis*LOST
Eucalyptusgrandis_lle11
Aquilegiacoerulea_lle12
Chenopodiumquinoa_lle16
Spinaciaoleracea_lle25
Helianthusannuus_lle25
Lactucasativa_lle24
Daucuscarota*LOST
Coffeaarabica_lle12
Solanumlycopersicum_lle22
Oleaeuropaea_lle5
Fragariavesca_lle19
Prunuspersica_lle19
n52610*LOST
Arachishypogaea_lle8
Phaseolusvulgaris_lle16
Trifoliumpratense_lle45
Cicerarietinum*LOST
Lotusjaponicus_lle6
Linumusitatissimum_lle15
Salixpurpurea_lle10
Populustrichocarpa_lle20
Manihotesculenta_lle1
Gossypiumraimondii_lle32
Gossypiumhirsutum_lle21
Theobromacacao*LOST

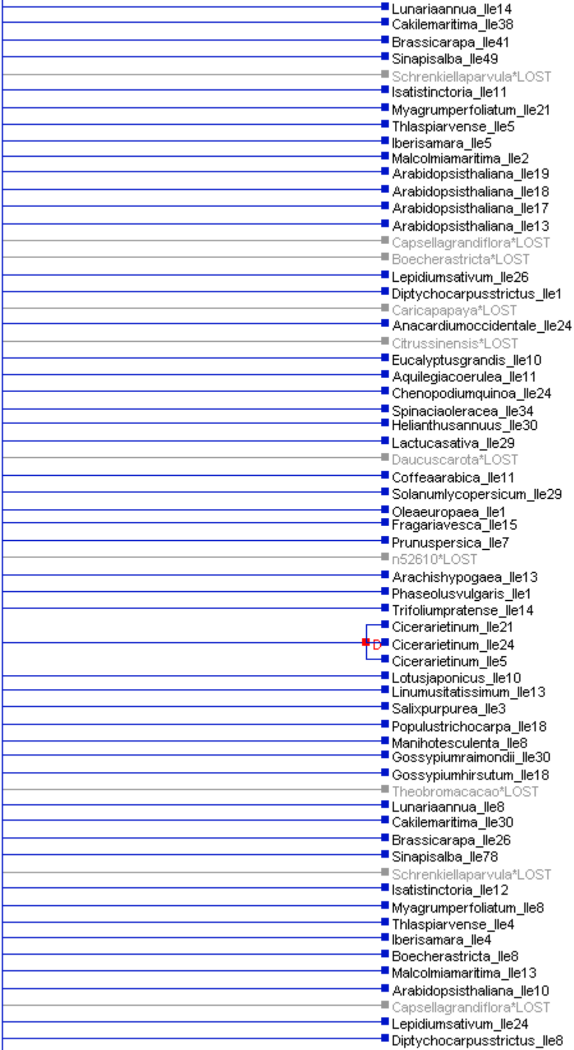
Lunariaannua_Ile14
Cakilemaritima_Ile38
Brassicarapa_Ile41
Sinapisalba_Ile49
Schrenkiellaparvula*LOST
Isatistinctoria_Ile11
Myagrumperfoliatum_Ile21
Thlaspiarvense_Ile5
Iberisamara_Ile5
Malcolmiamaritima_Ile2
Arabidopsisthaliana_Ile19
Arabidopsisthaliana_Ile18
Arabidopsisthaliana_Ile17
Arabidopsisthaliana_Ile13
Capsellagrandiflora*LOST
Boecherastricta*LOST
Lepidiumsativum_Ile26
Diptychocarpusstrictus_Ile1
Caricapapaya*LOST
Anacardiumoccidentale_Ile24
Citrussinensis*LOST
Eucalyptusgrandis_Ile10
Aquilegiacoerulea_Ile11
Chenopodiumquinoa_Ile24
Spinaciaoleracea_Ile34
Helianthusannuus_Ile30
Lactucasativa_Ile29
Daucuscarota*LOST
Coffeaarabica_Ile11
Solanumlycopersicum_Ile29
Oleaeuropaea_Ile1
Fragariavesca_Ile15
Prunuspersica_Ile7
n52610*LOST
Arachishypogaea_Ile13
Phaseolusvulgaris_Ile1
Trifoliumpratense_Ile14
Cicerarietinum_Ile21
D
Cicerarietinum_Ile24
Cicerarietinum_Ile5
Lotusjaponicus_Ile10
Linumusitatissimum_Ile13
Salixpurpurea_Ile3
Populustrichocarpa_Ile18
Manihotesculenta_Ile8
Gossypiumraimondii_Ile30
Gossypiumhirsutum_Ile18
Theobromacacao*LOST
Lunariaannua_Ile8
Cakilemaritima_Ile30
Brassicarapa_Ile26
Sinapisalba_Ile78
Schrenkiellaparvula*LOST
Isatistinctoria_Ile12
Myagrumperfoliatum_Ile8
Thlaspiarvense_Ile4
Iberisamara_Ile4
Boecherastricta_Ile8
Malcolmiamaritima_Ile13
Arabidopsisthaliana_Ile10
Capsellagrandiflora*LOST
Lepidiumsativum_Ile24
Diptychocarpusstrictus_Ile8

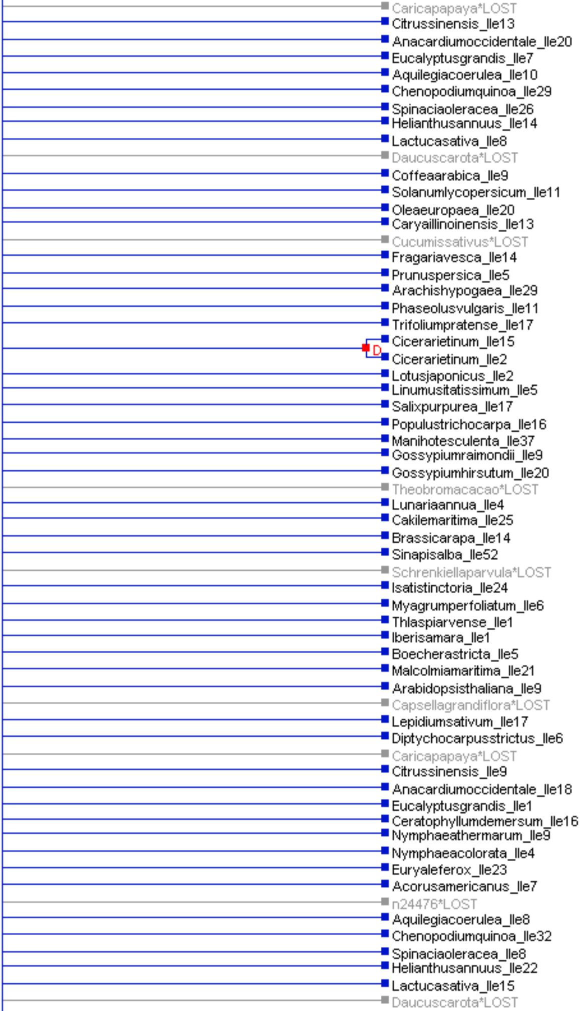
Caricapapaya*LOST
Citrussinensis_lle13
Anacardiumoccidentale_lle20
Eucalyptusgrandis_lle7
Aquilegiacoerulea_lle10
Chenopodiumquinoa_lle29
Spinaciaoleracea_lle26
Helianthusannuus_lle14
Lactucasativa_lle8
Daucuscarota*LOST
Coffeaarabica_lle9
Solanumlycopersicum_lle11
Oleaeuropaea_lle20
Caryaillinoinensis_lle13
Cucumissativus*LOST
Fragariavesca_lle14
Prunuspersica_lle5
Arachishypogaea_lle29
Phaseolusvulgaris_lle11
Trifoliumpratense_lle17
Cicerarietinum_lle15
D
Cicerarietinum_lle2
Lotusjaponicus_lle2
Linumusitatissimum_lle5
Salixpurpurea_lle17
Populustrichocarpa_lle16
Manihotesculenta_lle37
Gossypiumraimondii_lle9
Gossypiumhirsutum_lle20
Theobromacacao*LOST
Lunariaannua_lle4
Cakilemaritima_lle25
Brassicarapa_lle14
Sinapisalba_lle52
Schrenkiellaparvula*LOST
Isatistinctoria_lle24
Myagrumperfoliatum_lle6
Thlaspiarvense_lle1
Iberisamara_lle1
Boecherastricta_lle5
Malcolmiamaritima_lle21
Arabidopsisthaliana_lle9
Capsellagrandiflora*LOST
Lepidiumsativum_lle17
Diptychocarpusstrictus_lle6
Caricapapaya*LOST
Citrussinensis_lle9
Anacardiumoccidentale_lle18
Eucalyptusgrandis_lle1
Ceratophyllumdemersum_lle16
Nymphaeathermarum_lle9
Nymphaeacolorata_lle4
Euryaleferox_lle23
Acorusamericanus_lle7
n24476*LOST
Aquilegiacoerulea_lle8
Chenopodiumquinoa_lle32
Spinaciaoleracea_lle8
Helianthusannuus_lle22
Lactucasativa_lle15
Daucuscarota*LOST

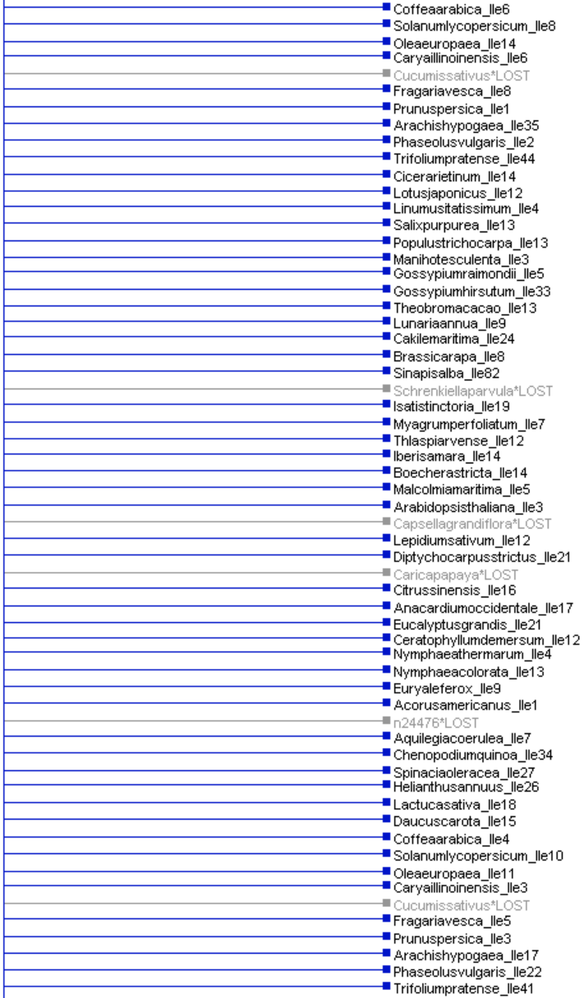
Coffeaarabica_lle6
Solanumlycopersicum_lle8
Oleaeuropaea_lle14
Caryaillinoinensis_lle6
Cucumissativus*LOST
Fragariavesca_lle8
Prunuspersica_lle1
Arachishypogaea_lle35
Phaseolusvulgaris_lle2
Trifoliumpratense_lle44
Cicerarietinum_lle14
Lotusjaponicus_lle12
Linumusitatissimum_lle4
Salixpurpurea_lle13
Populustrichocarpa_lle13
Manihotesculenta_lle3
Gossypiumraimondii_lle5
Gossypiumhirsutum_lle33
Theobromacacao_lle13
Lunariaannua_lle9
Cakilemaritima_lle24
Brassicarapa_lle8
Sinapisalba_lle82
Schrenkiellaparvula*LOST
Isatistinctoria_lle19
Myagrumperfoliatum_lle7
Thlaspiarvense_lle12
Iberisamara_lle14
Boecherastricta_lle14
Malcolmiamaritima_lle5
Arabidopsisthaliana_lle3
Capsellagrandiflora*LOST
Lepidiumsativum_lle12
Diptychocarpusstrictus_lle21
Caricapapaya*LOST
Citrussinensis_lle16
Anacardiumoccidentale_lle17
Eucalyptusgrandis_lle21
Ceratophyllumdemersum_lle12
Nymphaeathermarum_lle4
Nymphaeacolorata_lle13
Euryaleferox_lle9
Acorusamericanus_lle1
n24476*LOST
Aquilegiacoerulea_lle7
Chenopodiumquinoa_lle34
Spinaciaoleracea_lle27
Helianthusannuus_lle26
Lactucasativa_lle18
Daucuscarota_lle15
Coffeaarabica_lle4
Solanumlycopersicum_lle10
Oleaeuropaea_lle11
Caryaillinoinensis_lle3
Cucumissativus*LOST
Fragariavesca_lle5
Prunuspersica_lle3
Arachishypogaea_lle17
Phaseolusvulgaris_lle22
Trifoliumpratense_lle41

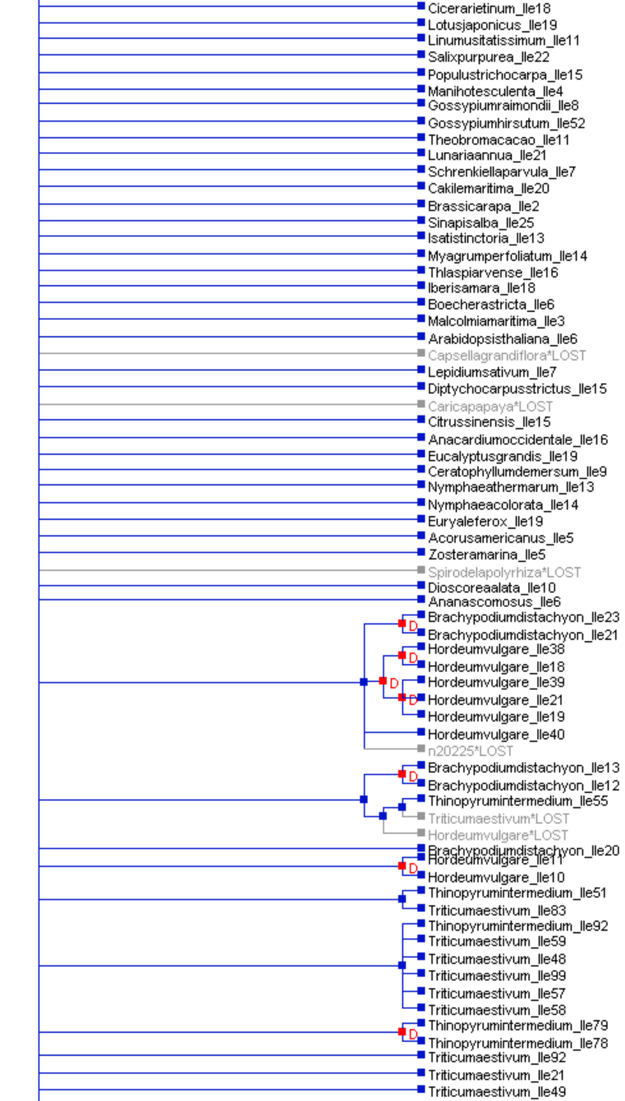
Cicerarietinum_lle18
Lotusjaponicus_lle19
Linumusitatissimum_lle11
Salixpurpurea_lle22
Populustrichocarpa_lle15
Manihotesculenta_lle4
Gossypiumraimondii_lle8
Gossypiumhirsutum_lle52
Theobromacacao_lle11
Lunariaannua_lle21
Schrenkiellaparvula_lle7
Cakilemaritima_lle20
Brassicarapa_lle2
Sinapisalba_lle25
Isatistinctoria_lle13
Myagrumperfoliatum_lle14
Thlaspiarvense_lle16
Iberisamara_lle18
Boecherastricta_lle6
Malcolmiamaritima_lle3
Arabidopsisthaliana_lle6
Capsellagrandiflora*LOST
Lepidiumsativum_lle7
Diptychocarpusstrictus_lle15
Caricapapaya*LOST
Citrussinensis_lle15
Anacardiumoccidentale_lle16
Eucalyptusgrandis_lle19
Ceratophyllumdemersum_lle9
Nymphaeathermarum_lle13
Nymphaeacolorata_lle14
Euryaleferox_lle19
Acorusamericanus_lle5
Zosteramarina_lle5
Spirodelapolyrhiza*LOST
Dioscoreaalata_lle10
Ananascomosus_lle6
Brachypodiumdistachyon_lle23
D
Brachypodiumdistachyon_lle21
Hordeumvulgare_lle38
D
Hordeumvulgare_lle18
D
Hordeumvulgare_lle39
D
Hordeumvulgare_lle21
Hordeumvulgare_lle19
Hordeumvulgare_lle40
n20225*LOST
Brachypodiumdistachyon_lle13
D
Brachypodiumdistachyon_lle12
Thinopyrumintermedium_lle55
Triticumaestivum*LOST
Hordeumvulgare*LOST
Brachypodiumdistachyon_lle20
Hordeumvulgare_lle11
D
Hordeumvulgare_lle10
Thinopyrumintermedium_lle51
Triticumaestivum_lle83
Thinopyrumintermedium_lle92
Triticumaestivum_lle59
Triticumaestivum_lle48
Triticumaestivum_lle99
Triticumaestivum_lle57
Triticumaestivum_lle58
Thinopyrumintermedium_lle79
D
Thinopyrumintermedium_lle78
Triticumaestivum_lle92
Triticumaestivum_lle21
Triticumaestivum_lle49

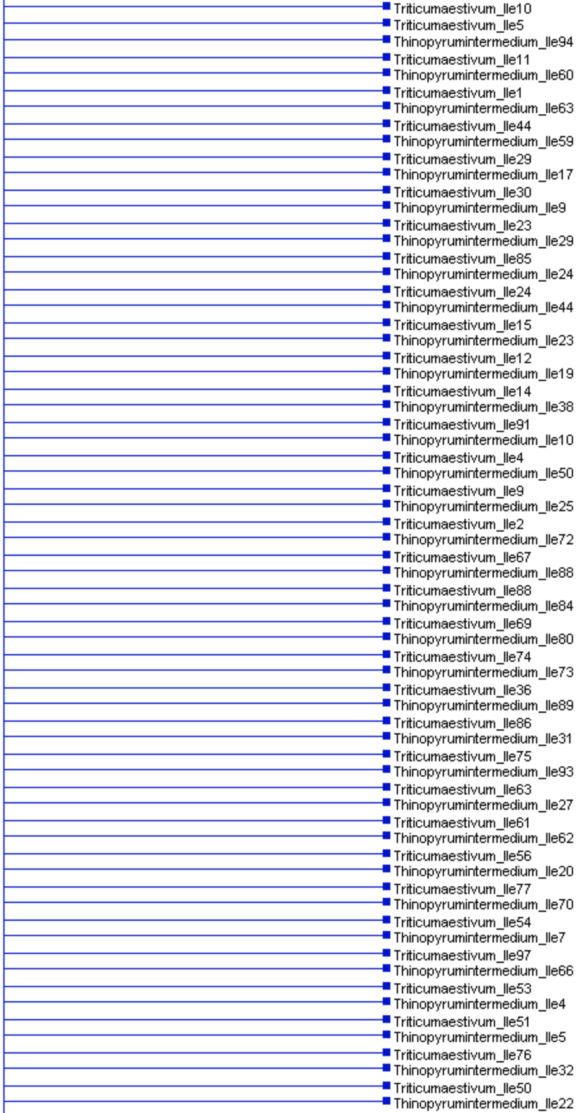
Triticumaestivum_Ile10
Triticumaestivum_Ile5
Thinopyrumintermedium_Ile94
Triticumaestivum_Ile11
Thinopyrumintermedium_Ile60
Triticumaestivum_Ile1
Thinopyrumintermedium_Ile63
Triticumaestivum_Ile44
Thinopyrumintermedium_Ile59
Triticumaestivum_Ile29
Thinopyrumintermedium_Ile17
Triticumaestivum_Ile30
Thinopyrumintermedium_Ile9
Triticumaestivum_Ile23
Thinopyrumintermedium_Ile29
Triticumaestivum_Ile85
Thinopyrumintermedium_Ile24
Triticumaestivum_Ile24
Thinopyrumintermedium_Ile44
Triticumaestivum_Ile15
Thinopyrumintermedium_Ile23
Triticumaestivum_Ile12
Thinopyrumintermedium_Ile19
Triticumaestivum_Ile14
Thinopyrumintermedium_Ile38
Triticumaestivum_Ile91
Thinopyrumintermedium_Ile10
Triticumaestivum_Ile4
Thinopyrumintermedium_Ile50
Triticumaestivum_Ile9
Thinopyrumintermedium_Ile25
Triticumaestivum_Ile2
Thinopyrumintermedium_Ile72
Triticumaestivum_Ile67
Thinopyrumintermedium_Ile88
Triticumaestivum_Ile88
Thinopyrumintermedium_Ile84
Triticumaestivum_Ile69
Thinopyrumintermedium_Ile80
Triticumaestivum_Ile74
Thinopyrumintermedium_Ile73
Triticumaestivum_Ile36
Thinopyrumintermedium_Ile89
Triticumaestivum_Ile86
Thinopyrumintermedium_Ile31
Triticumaestivum_Ile75
Thinopyrumintermedium_Ile93
Triticumaestivum_Ile63
Thinopyrumintermedium_Ile27
Triticumaestivum_Ile61
Thinopyrumintermedium_Ile62
Triticumaestivum_Ile56
Thinopyrumintermedium_Ile20
Triticumaestivum_Ile77
Thinopyrumintermedium_Ile70
Triticumaestivum_Ile54
Thinopyrumintermedium_Ile7
Triticumaestivum_Ile97
Thinopyrumintermedium_Ile66
Triticumaestivum_Ile53
Thinopyrumintermedium_Ile4
Triticumaestivum_Ile51
Thinopyrumintermedium_Ile5
Triticumaestivum_Ile76
Thinopyrumintermedium_Ile32
Triticumaestivum_Ile50
Thinopyrumintermedium_Ile22

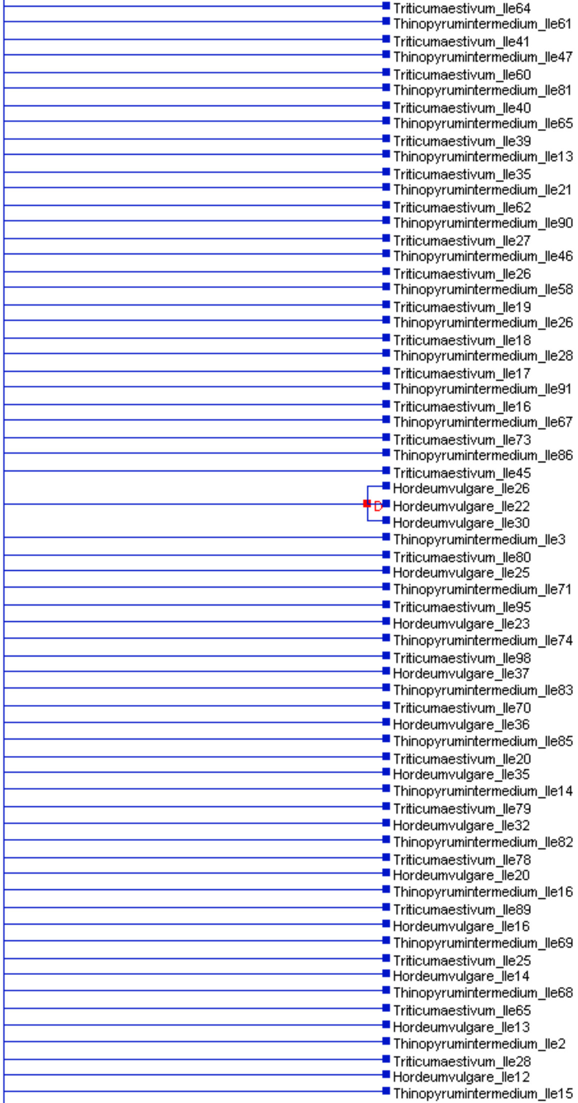

Triticumaestivum_lle64
Thinopyrumintermedium_lle61
Triticumaestivum_lle41
Thinopyrumintermedium_lle47
Triticumaestivum_lle60
Thinopyrumintermedium_lle81
Triticumaestivum_lle40
Thinopyrumintermedium_lle65
Triticumaestivum_lle39
Thinopyrumintermedium_lle13
Triticumaestivum_lle35
Thinopyrumintermedium_lle21
Triticumaestivum_lle62
Thinopyrumintermedium_lle90
Triticumaestivum_lle27
Thinopyrumintermedium_lle46
Triticumaestivum_lle26
Thinopyrumintermedium_lle58
Triticumaestivum_lle19
Thinopyrumintermedium_lle26
Triticumaestivum_lle18
Thinopyrumintermedium_lle28
Triticumaestivum_lle17
Thinopyrumintermedium_lle91
Triticumaestivum_lle16
Thinopyrumintermedium_lle67
Triticumaestivum_lle73
Thinopyrumintermedium_lle86
Triticumaestivum_lle45
Hordeumvulgare_lle26
D
Hordeumvulgare_lle22
Hordeumvulgare_lle30
Thinopyrumintermedium_lle3
Triticumaestivum_lle80
Hordeumvulgare_lle25
Thinopyrumintermedium_lle71
Triticumaestivum_lle95
Hordeumvulgare_lle23
Thinopyrumintermedium_lle74
Triticumaestivum_lle98
Hordeumvulgare_lle37
Thinopyrumintermedium_lle83
Triticumaestivum_lle70
Hordeumvulgare_lle36
Thinopyrumintermedium_lle85
Triticumaestivum_lle20
Hordeumvulgare_lle35
Thinopyrumintermedium_lle14
Triticumaestivum_lle79
Hordeumvulgare_lle32
Thinopyrumintermedium_lle82
Triticumaestivum_lle78
Hordeumvulgare_lle20
Thinopyrumintermedium_lle16
Triticumaestivum_lle89
Hordeumvulgare_lle16
Thinopyrumintermedium_lle69
Triticumaestivum_lle25
Hordeumvulgare_lle14
Thinopyrumintermedium_lle68
Triticumaestivum_lle65
Hordeumvulgare_lle13
Thinopyrumintermedium_lle2
Triticumaestivum_lle28
Hordeumvulgare_lle12
Thinopyrumintermedium_lle15

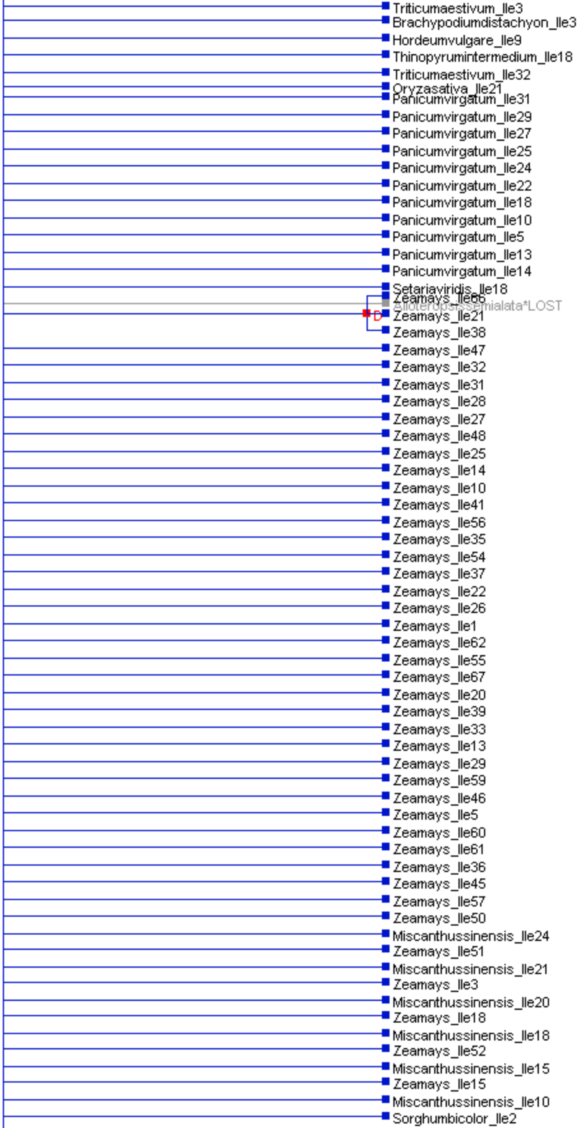

Triticumaestivum_Ile3
Brachypodiumdistachyon_Ile3
Hordeumvulgare_Ile9
Thinopyrumintermedium_Ile18
Triticumaestivum_Ile32
Oryzasativa_Ile21
Panicumvirgatum_Ile31
Panicumvirgatum_Ile29
Panicumvirgatum_Ile27
Panicumvirgatum_Ile25
Panicumvirgatum_Ile24
Panicumvirgatum_Ile22
Panicumvirgatum_Ile18
Panicumvirgatum_Ile10
Panicumvirgatum_Ile5
Panicumvirgatum_Ile13
Panicumvirgatum_Ile14
Setariaviridis_Ile18
Zeamays_Ile66
Alloteropsissemialata*LOST
D
Zeamays_Ile21
Zeamays_Ile38
Zeamays_Ile47
Zeamays_Ile32
Zeamays_Ile31
Zeamays_Ile28
Zeamays_Ile27
Zeamays_Ile48
Zeamays_Ile25
Zeamays_Ile14
Zeamays_Ile10
Zeamays_Ile41
Zeamays_Ile56
Zeamays_Ile35
Zeamays_Ile54
Zeamays_Ile37
Zeamays_Ile22
Zeamays_Ile26
Zeamays_Ile1
Zeamays_Ile62
Zeamays_Ile55
Zeamays_Ile67
Zeamays_Ile20
Zeamays_Ile39
Zeamays_Ile33
Zeamays_Ile13
Zeamays_Ile29
Zeamays_Ile59
Zeamays_Ile46
Zeamays_Ile5
Zeamays_Ile60
Zeamays_Ile61
Zeamays_Ile36
Zeamays_Ile45
Zeamays_Ile57
Zeamays_Ile50
Miscanthussinensis_Ile24
Zeamays_Ile51
Miscanthussinensis_Ile21
Zeamays_Ile3
Miscanthussinensis_Ile20
Zeamays_Ile18
Miscanthussinensis_Ile18
Zeamays_Ile52
Miscanthussinensis_Ile15
Zeamays_Ile15
Miscanthussinensis_Ile10
Sorghumbicolor_Ile2

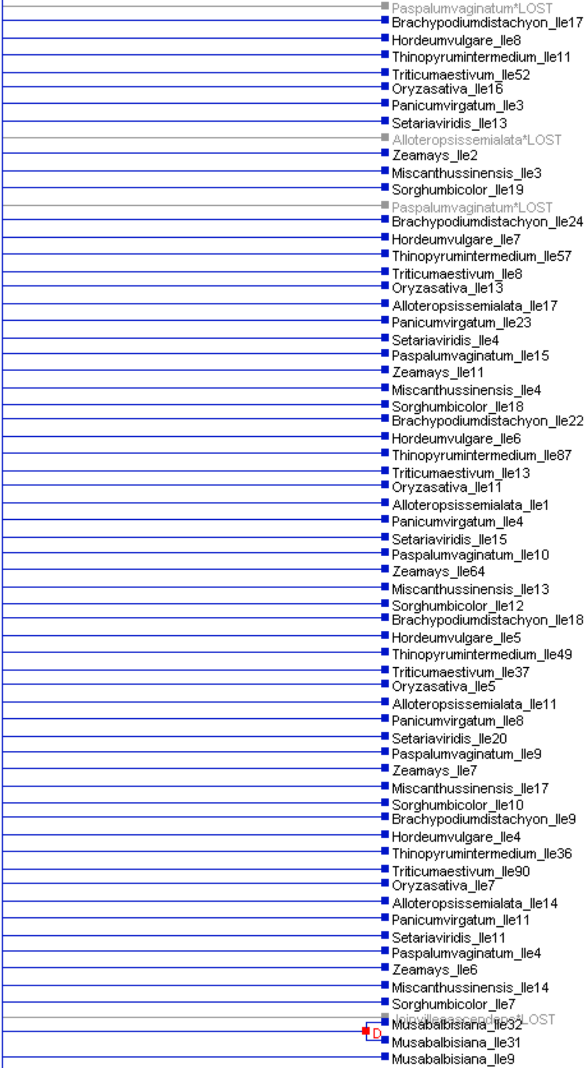
Paspalumvaginatum*LOST
Brachypodiumdistachyon_lle17
Hordeumvulgare_lle8
Thinopyrumintermedium_lle11
Triticumaestivum_lle52
Oryzasativa_lle16
Panicumvirgatum_lle3
Setariaviridis_lle13
Alloteropsissemialata*LOST
Zeamays_lle2
Miscanthussinensis_lle3
Sorghumbicolor_lle19
Paspalumvaginatum*LOST
Brachypodiumdistachyon_lle24
Hordeumvulgare_lle7
Thinopyrumintermedium_lle57
Triticumaestivum_lle8
Oryzasativa_lle13
Alloteropsissemialata_lle17
Panicumvirgatum_lle23
Setariaviridis_lle4
Paspalumvaginatum_lle15
Zeamays_lle11
Miscanthussinensis_lle4
Sorghumbicolor_lle18
Brachypodiumdistachyon_lle22
Hordeumvulgare_lle6
Thinopyrumintermedium_lle87
Triticumaestivum_lle13
Oryzasativa_lle11
Alloteropsissemialata_lle1
Panicumvirgatum_lle4
Setariaviridis_lle15
Paspalumvaginatum_lle10
Zeamays_lle64
Miscanthussinensis_lle13
Sorghumbicolor_lle12
Brachypodiumdistachyon_lle18
Hordeumvulgare_lle5
Thinopyrumintermedium_lle49
Triticumaestivum_lle37
Oryzasativa_lle5
Alloteropsissemialata_lle11
Panicumvirgatum_lle8
Setariaviridis_lle20
Paspalumvaginatum_lle9
Zeamays_lle7
Miscanthussinensis_lle17
Sorghumbicolor_lle10
Brachypodiumdistachyon_lle9
Hordeumvulgare_lle4
Thinopyrumintermedium_lle36
Triticumaestivum_lle90
Oryzasativa_lle7
Alloteropsissemialata_lle14
Panicumvirgatum_lle11
Setariaviridis_lle11
Paspalumvaginatum_lle4
Zeamays_lle6
Miscanthussinensis_lle14
Sorghumbicolor_lle7
Joinvilleaascendens*LOST
Musabalbisiana_lle32
D
Musabalbisiana_lle31
Musabalbisiana_lle9

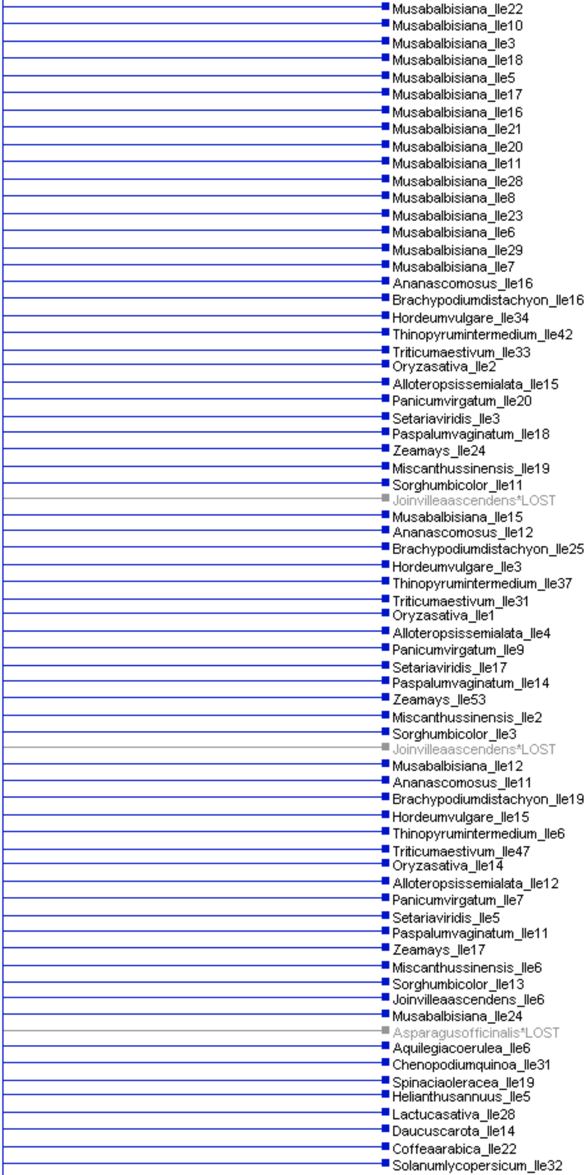

Musabalbisiana_lle22
Musabalbisiana_lle10
Musabalbisiana_lle3
Musabalbisiana_lle18
Musabalbisiana_lle5
Musabalbisiana_lle17
Musabalbisiana_lle16
Musabalbisiana_lle21
Musabalbisiana_lle20
Musabalbisiana_lle11
Musabalbisiana_lle28
Musabalbisiana_lle8
Musabalbisiana_lle23
Musabalbisiana_lle6
Musabalbisiana_lle29
Musabalbisiana_lle7
Ananascomosus_lle16
Brachypodiumdistachyon_lle16
Hordeumvulgare_lle34
Thinopyrumintermedium_lle42
Triticumaestivum_lle33
Oryzasativa_lle2
Alloteropsissemialata_lle15
Panicumvirgatum_lle20
Setariaviridis_lle3
Paspalumvaginatum_lle18
Zeamays_lle24
Miscanthussinensis_lle19
Sorghumbicolor_lle11
Joinvilleaascendens*LOST
Musabalbisiana_lle15
Ananascomosus_lle12
Brachypodiumdistachyon_lle25
Hordeumvulgare_lle3
Thinopyrumintermedium_lle37
Triticumaestivum_lle31
Oryzasativa_lle1
Alloteropsissemialata_lle4
Panicumvirgatum_lle9
Setariaviridis_lle17
Paspalumvaginatum_lle14
Zeamays_lle53
Miscanthussinensis_lle2
Sorghumbicolor_lle3
Joinvilleaascendens*LOST
Musabalbisiana_lle12
Ananascomosus_lle11
Brachypodiumdistachyon_lle19
Hordeumvulgare_lle15
Thinopyrumintermedium_lle6
Triticumaestivum_lle47
Oryzasativa_lle14
Alloteropsissemialata_lle12
Panicumvirgatum_lle7
Setariaviridis_lle5
Paspalumvaginatum_lle11
Zeamays_lle17
Miscanthussinensis_lle6
Sorghumbicolor_lle13
Joinvilleaascendens_lle6
Musabalbisiana_lle24
Asparagusofficinalis*LOST
Aquilegiacoerulea_lle6
Chenopodiumquinoa_lle31
Spinaciaoleracea_lle19
Helianthusannuus_lle5
Lactucasativa_lle28
Daucuscarota_lle14
Coffeaarabica_lle22
Solanumlycopersicum_lle32

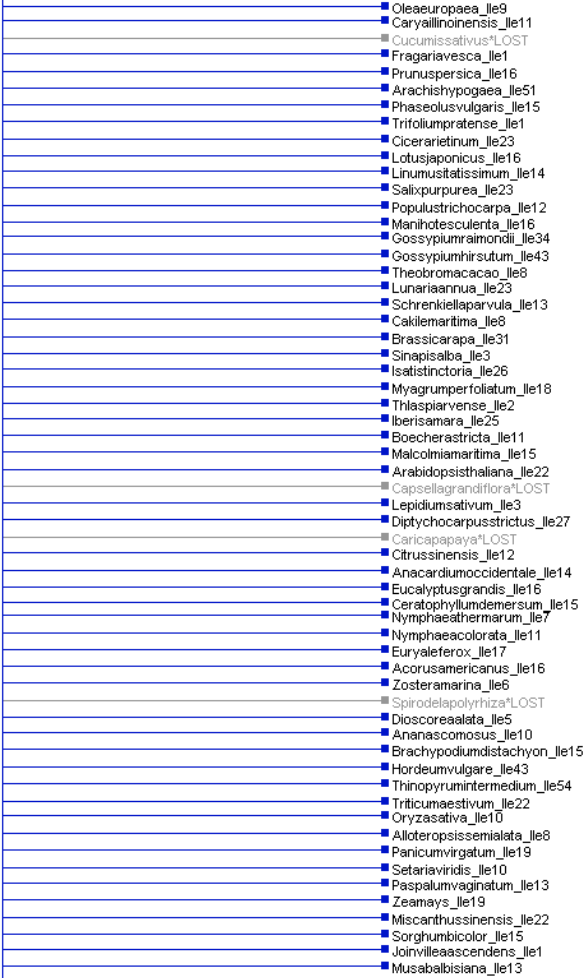
Oleaeuropaea_lle9
Caryaillinoinensis_lle11
Cucumissativus*LOST
Fragariavesca_lle1
Prunuspersica_lle16
Arachishypogaea_lle51
Phaseolusvulgaris_lle15
Trifoliumpratense_lle1
Cicerarietinum_lle23
Lotusjaponicus_lle16
Linumusitatissimum_lle14
Salixpurpurea_lle23
Populustrichocarpa_lle12
Manihotesculenta_lle16
Gossypiumraimondii_lle34
Gossypiumhirsutum_lle43
Theobromacacao_lle8
Lunariaannua_lle23
Schrenkiellaparvula_lle13
Cakilemaritima_lle8
Brassicarapa_lle31
Sinapisalba_lle3
Isatistinctoria_lle26
Myagrumperfoliatum_lle18
Thlaspiarvense_lle2
Iberisamara_lle25
Boecherastricta_lle11
Malcolmiamaritima_lle15
Arabidopsisthaliana_lle22
Capsellagrandiflora*LOST
Lepidiumsativum_lle3
Diptychocarpusstrictus_lle27
Caricapapaya*LOST
Citrussinensis_lle12
Anacardiumoccidentale_lle14
Eucalyptusgrandis_lle16
Ceratophyllumdemersum_lle15
Nymphaeathermarum_lle7
Nymphaeacolorata_lle11
Euryaleferox_lle17
Acorusamericanus_lle16
Zosteramarina_lle6
Spirodelapolyrhiza*LOST
Dioscoreaalata_lle5
Ananascomosus_lle10
Brachypodiumdistachyon_lle15
Hordeumvulgare_lle43
Thinopyrumintermedium_lle54
Triticumaestivum_lle22
Oryzasativa_lle10
Alloteropsissemialata_lle8
Panicumvirgatum_lle19
Setariaviridis_lle10
Paspalumvaginatum_lle13
Zeamays_lle19
Miscanthussinensis_lle22
Sorghumbicolor_lle15
Joinvilleaascendens_lle1
Musabalbisiana_lle13

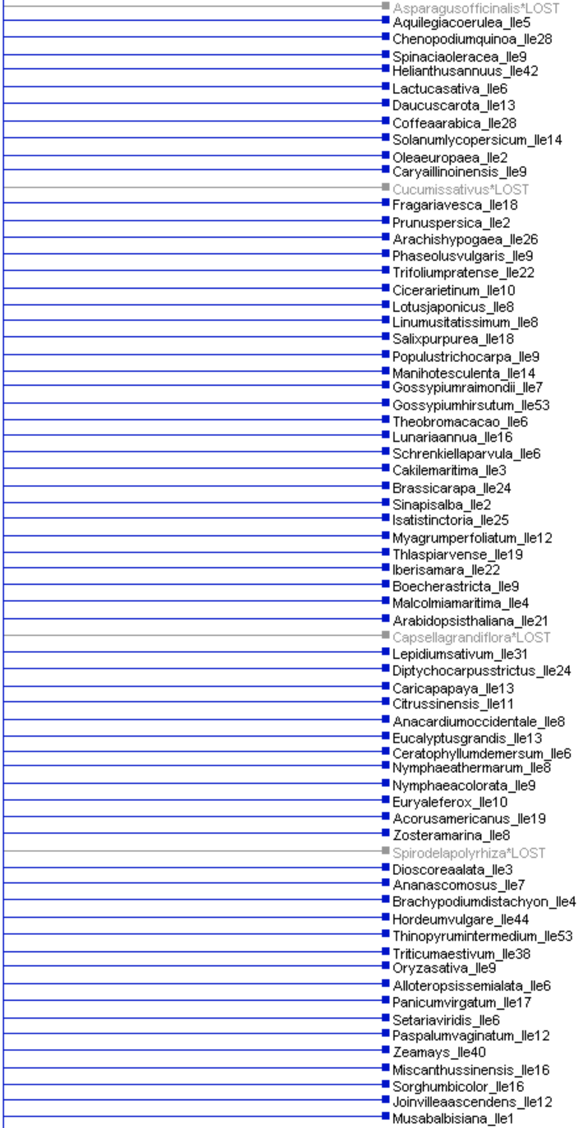

Asparagusofficinalis*LOST
Aquilegiacoerulea_Ile5
Chenopodiumquinoa_Ile28
Spinaciaoleracea_Ile9
Helianthusannuus_Ile42
Lactucasativa_Ile6
Daucuscarota_Ile13
Coffeaarabica_Ile28
Solanumlycopersicum_Ile14
Oleaeuropaea_Ile2
Caryaillinoinensis_Ile9
Cucumissativus*LOST
Fragariavesca_Ile18
Prunuspersica_Ile2
Arachishypogaea_Ile26
Phaseolusvulgaris_Ile9
Trifoliumpratense_Ile22
Cicerarietinum_Ile10
Lotusjaponicus_Ile8
Linumusitatissimum_Ile8
Salixpurpurea_Ile18
Populustrichocarpa_Ile9
Manihotesculenta_Ile14
Gossypiumraimondii_Ile7
Gossypiumhirsutum_Ile53
Theobromacacao_Ile6
Lunariaannua_Ile16
Schrenkiellaparvula_Ile6
Cakilemaritima_Ile3
Brassicarapa_Ile24
Sinapisalba_Ile2
Isatistinctoria_Ile25
Myagrumperfoliatum_Ile12
Thlaspiarvense_Ile19
Iberisamara_Ile22
Boecherastricta_Ile9
Malcolmiamaritima_Ile4
Arabidopsisthaliana_Ile21
Capsellagrandiflora*LOST
Lepidiumsativum_Ile31
Diptychocarpusstrictus_Ile24
Caricapapaya_Ile13
Citrussinensis_Ile11
Anacardiumoccidentale_Ile8
Eucalyptusgrandis_Ile13
Ceratophyllumdemersum_Ile6
Nymphaeathermarum_Ile8
Nymphaeacolorata_Ile9
Euryaleferox_Ile10
Acorusamericanus_Ile19
Zosteramarina_Ile8
Spirodelapolyrhiza*LOST
Dioscoreaalata_Ile3
Ananascomosus_Ile7
Brachypodiumdistachyon_Ile4
Hordeumvulgare_Ile44
Thinopyrumintermedium_Ile53
Triticumaestivum_Ile38
Oryzasativa_Ile9
Alloteropsissemialata_Ile6
Panicumvirgatum_Ile17
Setariaviridis_Ile6
Paspalumvaginatum_Ile12
Zeamays_Ile40
Miscanthussinensis_Ile16
Sorghumbicolor_Ile16
Joinvilleaascendens_Ile12
Musabalbisiana_Ile1

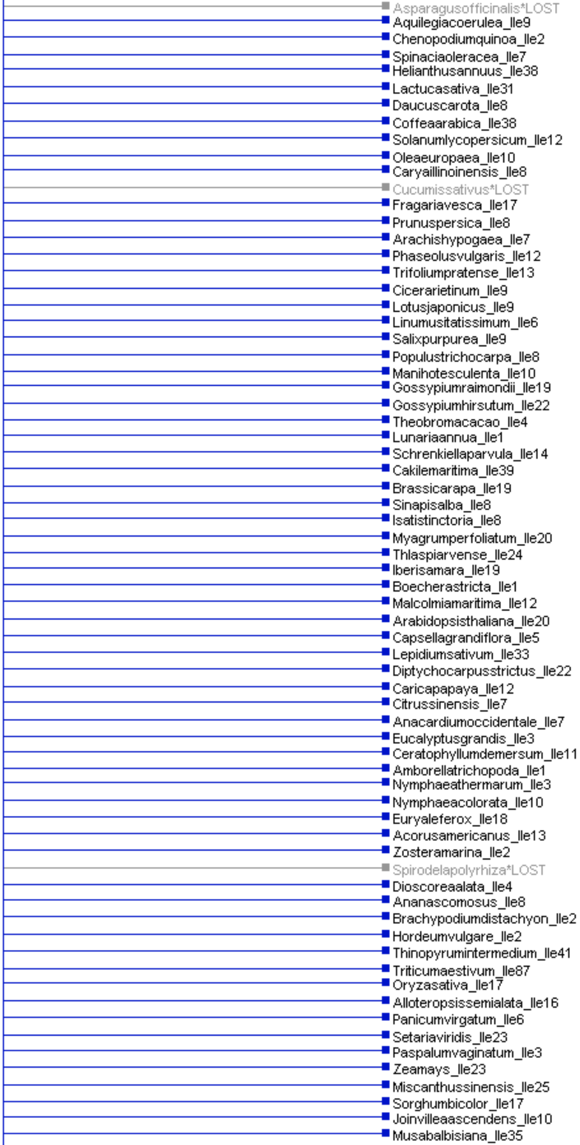
Asparagusofficinalis*LOST
Aquilegiacoerulea_Ile9
Chenopodiumquinoa_Ile2
Spinaciaoleracea_Ile7
Helianthusannuus_Ile38
Lactucasativa_Ile31
Daucuscarota_Ile8
Coffeaarabica_Ile38
Solanumlycopersicum_Ile12
Oleaeuropaea_Ile10
Caryaillinoinensis_Ile8
Cucumissativus*LOST
Fragariavesca_Ile17
Prunuspersica_Ile8
Arachishypogaea_Ile7
Phaseolusvulgaris_Ile12
Trifoliumpratense_Ile13
Cicerarietinum_Ile9
Lotusjaponicus_Ile9
Linumusitatissimum_Ile6
Salixpurpurea_Ile9
Populustrichocarpa_Ile8
Manihotesculenta_Ile10
Gossypiumraimondii_Ile19
Gossypiumhirsutum_Ile22
Theobromacacao_Ile4
Lunariaannua_Ile1
Schrenkiellaparvula_Ile14
Cakilemaritima_Ile39
Brassicarapa_Ile19
Sinapisalba_Ile8
Isatistinctoria_Ile8
Myagrumperfoliatum_Ile20
Thlaspiarvense_Ile24
Iberisamara_Ile19
Boecherastricta_Ile1
Malcolmiamaritima_Ile12
Arabidopsisthaliana_Ile20
Capsellagrandiflora_Ile5
Lepidiumsativum_Ile33
Diptychocarpusstrictus_Ile22
Caricapapaya_Ile12
Citrussinensis_Ile7
Anacardiumoccidentale_Ile7
Eucalyptusgrandis_Ile3
Ceratophyllumdemersum_Ile11
Amborellatrichopoda_Ile1
Nymphaeathermarum_Ile3
Nymphaeacolorata_Ile10
Euryaleferox_Ile18
Acorusamericanus_Ile13
Zosteramarina_Ile2
Spirodelapolyrhiza*LOST
Dioscoreaalata_Ile4
Ananascomosus_Ile8
Brachypodiumdistachyon_Ile2
Hordeumvulgare_Ile2
Thinopyrumintermedium_Ile41
Triticumaestivum_Ile87
Oryzasativa_Ile17
Alloteropsissemialata_Ile16
Panicumvirgatum_Ile6
Setariaviridis_Ile23
Paspalumvaginatum_Ile3
Zeamays_Ile23
Miscanthussinensis_Ile25
Sorghumbicolor_Ile17
Joinvilleaascendens_Ile10
Musabalbisiana_Ile35

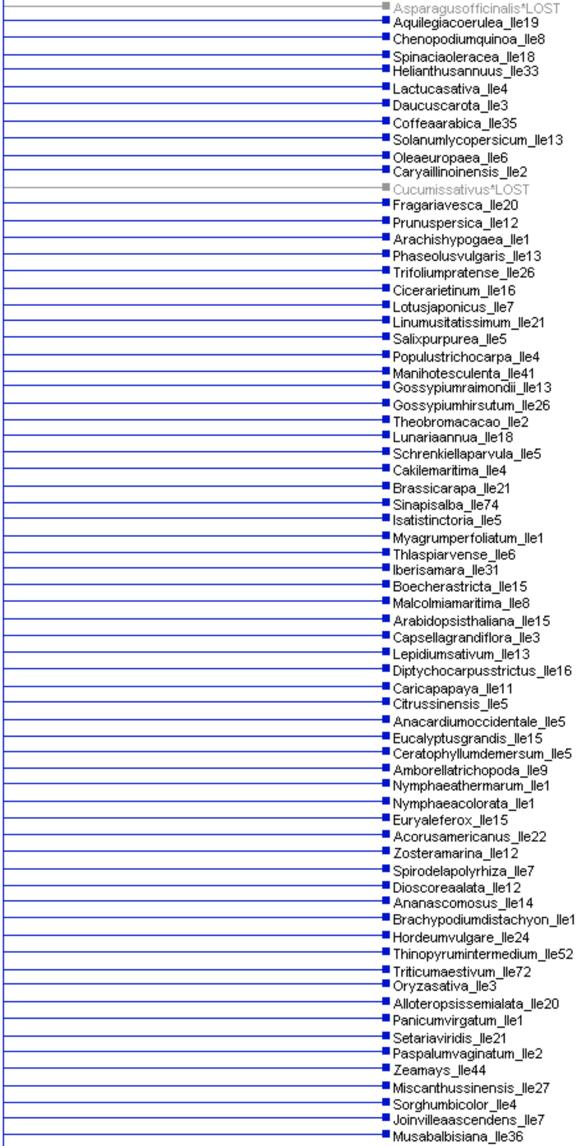
Asparagusofficinalis*LOST
Aquilegiacoerulea_lle19
Chenopodiumquinoa_lle8
Spinaciaoleracea_lle18
Helianthusannuus_lle33
Lactucasativa_lle4
Daucuscarota_lle3
Coffeaarabica_lle35
Solanumlycopersicum_lle13
Oleaeuropaea_lle6
Caryaillinoinensis_lle2
Cucumissativus*LOST
Fragariavesca_lle20
Prunuspersica_lle12
Arachishypogaea_lle1
Phaseolusvulgaris_lle13
Trifoliumpratense_lle26
Cicerarietinum_lle16
Lotusjaponicus_lle7
Linumusitatissimum_lle21
Salixpurpurea_lle5
Populustrichocarpa_lle4
Manihotesculenta_lle41
Gossypiumraimondii_lle13
Gossypiumhirsutum_lle26
Theobromacacao_lle2
Lunariaannua_lle18
Schrenkiellaparvula_lle5
Cakilemaritima_lle4
Brassicarapa_lle21
Sinapisalba_lle74
Isatistinctoria_lle5
Myagrumperfoliatum_lle1
Thlaspiarvense_lle6
Iberisamara_lle31
Boecherastricta_lle15
Malcolmiamaritima_lle8
Arabidopsisthaliana_lle15
Capsellagrandiflora_lle3
Lepidiumsativum_lle13
Diptychocarpusstrictus_lle16
Caricapapaya_lle11
Citrussinensis_lle5
Anacardiumoccidentale_lle5
Eucalyptusgrandis_lle15
Ceratophyllumdemersum_lle5
Amborellatrichopoda_lle9
Nymphaeathermarum_lle1
Nymphaeacolorata_lle1
Euryaleferox_lle15
Acorusamericanus_lle22
Zosteramarina_lle12
Spirodelapolyrhiza_lle7
Dioscoreaalata_lle12
Ananascomosus_lle14
Brachypodiumdistachyon_lle1
Hordeumvulgare_lle24
Thinopyrumintermedium_lle52
Triticumaestivum_lle72
Oryzasativa_lle3
Alloteropsissemialata_lle20
Panicumvirgatum_lle1
Setariaviridis_lle21
Paspalumvaginatum_lle2
Zeamays_lle44
Miscanthussinensis_lle27
Sorghumbicolor_lle4
Joinvilleaascendens_lle7
Musabalbisiana_lle36

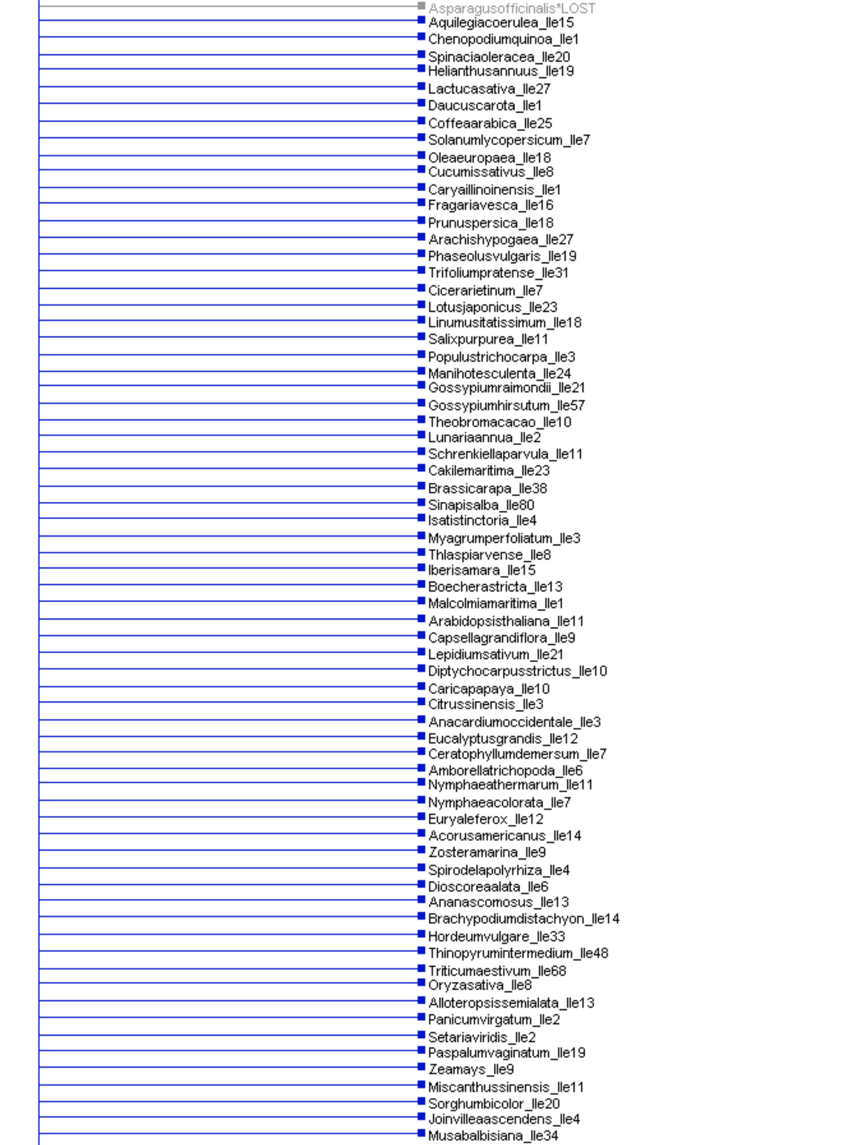
Asparagusofficinalis*LOST
Aquilegiacoerulea_Ile15
Chenopodiumquinoa_Ile1
Spinaciaoleracea_Ile20
Helianthusannuus_Ile19
Lactucasativa_Ile27
Daucuscarota_Ile1
Coffeaarabica_Ile25
Solanumlycopersicum_Ile7
Oleaeuropaea_Ile18
Cucumissativus_Ile8
Caryaillinoinensis_Ile1
Fragariavesca_Ile16
Prunuspersica_Ile18
Arachishypogaea_Ile27
Phaseolusvulgaris_Ile19
Trifoliumpratense_Ile31
Cicerarietinum_Ile7
Lotusjaponicus_Ile23
Linumusitatissimum_Ile18
Salixpurpurea_Ile11
Populustrichocarpa_Ile3
Manihotesculenta_Ile24
Gossypiumraimondii_Ile21
Gossypiumhirsutum_Ile57
Theobromacacao_Ile10
Lunariaannua_Ile2
Schrenkiellaparvula_Ile11
Cakilemaritima_Ile23
Brassicarapa_Ile38
Sinapisalba_Ile80
Isatistinctoria_Ile4
Myagrumperfoliatum_Ile3
Thlaspiarvense_Ile8
Iberisamara_Ile15
Boecherastricta_Ile13
Malcolmiamaritima_Ile1
Arabidopsisthaliana_Ile11
Capsellagrandiflora_Ile9
Lepidiumsativum_Ile21
Diptychocarpusstrictus_Ile10
Caricapapaya_Ile10
Citrussinensis_Ile3
Anacardiumoccidentale_Ile3
Eucalyptusgrandis_Ile12
Ceratophyllumdemersum_Ile7
Amborellatrichopoda_Ile6
Nymphaeathermarum_Ile11
Nymphaeacolorata_Ile7
Euryaleferox_Ile12
Acorusamericanus_Ile14
Zosteramarina_Ile9
Spirodelapolyrhiza_Ile4
Dioscoreaalata_Ile6
Ananascomosus_Ile13
Brachypodiumdistachyon_Ile14
Hordeumvulgare_Ile33
Thinopyrumintermedium_Ile48
Triticumaestivum_Ile68
Oryzasativa_Ile8
Alloteropsissemialata_Ile13
Panicumvirgatum_Ile2
Setariaviridis_Ile2
Paspalumvaginatum_Ile19
Zeamays_Ile9
Miscanthussinensis_Ile11
Sorghumbicolor_Ile20
Joinvilleaascendens_Ile4
Musabalbisiana_Ile34

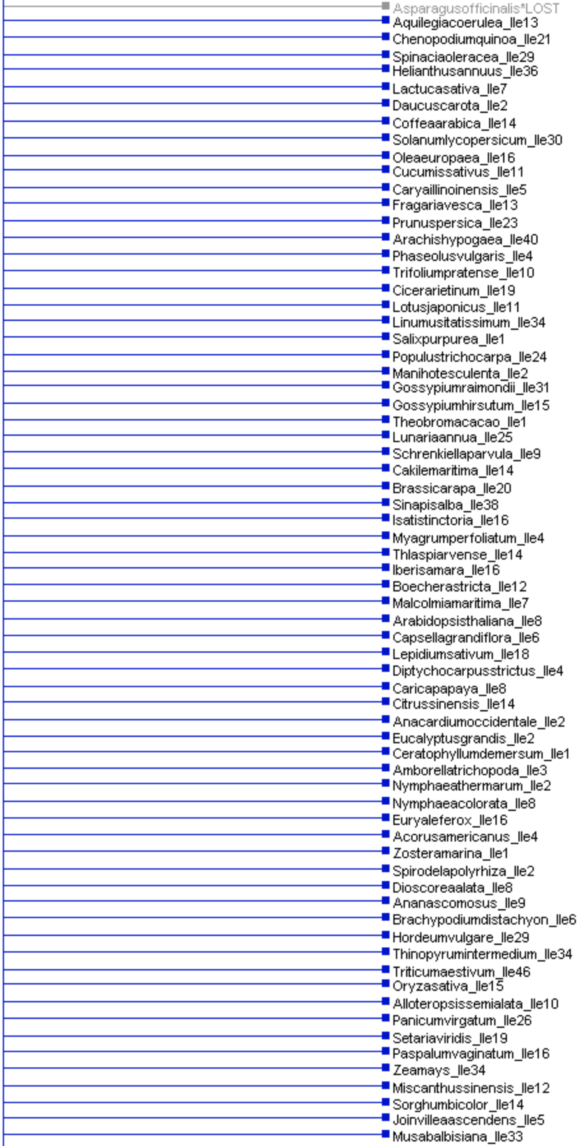
Asparagusofficinalis*LOST
Aquilegiacoerulea_Ile13
Chenopodiumquinoa_Ile21
Spinaciaoleracea_Ile29
Helianthusannuus_Ile36
Lactucasativa_Ile7
Daucuscarota_Ile2
Coffeaarabica_Ile14
Solanumlycopersicum_Ile30
Oleaeuropaea_Ile16
Cucumissativus_Ile11
Caryaillinoinensis_Ile5
Fragariavesca_Ile13
Prunuspersica_Ile23
Arachishypogaea_Ile40
Phaseolusvulgaris_Ile4
Trifoliumpratense_Ile10
Cicerarietinum_Ile19
Lotusjaponicus_Ile11
Linumusitatissimum_Ile34
Salixpurpurea_Ile1
Populustrichocarpa_Ile24
Manihotesculenta_Ile2
Gossypiumraimondii_Ile31
Gossypiumhirsutum_Ile15
Theobromacacao_Ile1
Lunariaannua_Ile25
Schrenkiellaparvula_Ile9
Cakilemaritima_Ile14
Brassicarapa_Ile20
Sinapisalba_Ile38
Isatistinctoria_Ile16
Myagrumperfoliatum_Ile4
Thlaspiarvense_Ile14
Iberisamara_Ile16
Boecherastricta_Ile12
Malcolmiamaritima_Ile7
Arabidopsisthaliana_Ile8
Capsellagrandiflora_Ile6
Lepidiumsativum_Ile18
Diptychocarpusstrictus_Ile4
Caricapapaya_Ile8
Citrussinensis_Ile14
Anacardiumoccidentale_Ile2
Eucalyptusgrandis_Ile2
Ceratophyllumdemersum_Ile1
Amborellatrichopoda_Ile3
Nymphaeathermarum_Ile2
Nymphaeacolorata_Ile8
Euryaleferox_Ile16
Acorusamericanus_Ile4
Zosteramarina_Ile1
Spirodelapolyrhiza_Ile2
Dioscoreaalata_Ile8
Ananascomosus_Ile9
Brachypodiumdistachyon_Ile6
Hordeumvulgare_Ile29
Thinopyrumintermedium_Ile34
Triticumaestivum_Ile46
Oryzasativa_Ile15
Alloteropsissemialata_Ile10
Panicumvirgatum_Ile26
Setariaviridis_Ile19
Paspalumvaginatum_Ile16
Zeamays_Ile34
Miscanthussinensis_Ile12
Sorghumbicolor_Ile14
Joinvilleaascendens_Ile5
Musabalbisiana_Ile33

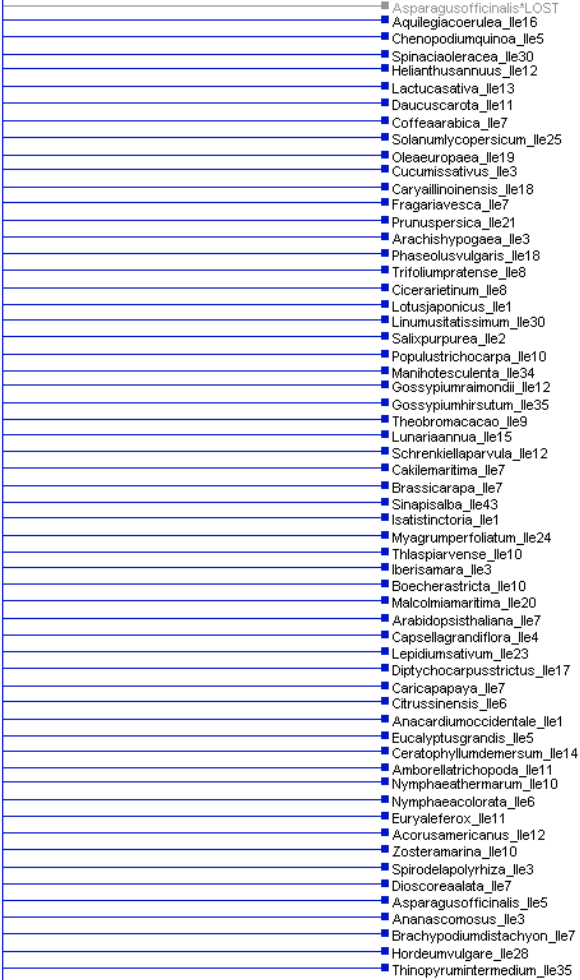
Asparagusofficinalis*LOST
Aquilegiacoerulea_Ile16
Chenopodiumquinoa_Ile5
Spinaciaoleracea_Ile30
Helianthusannuus_Ile12
Lactucasativa_Ile13
Daucuscarota_Ile11
Coffeaarabica_Ile7
Solanumlycopersicum_Ile25
Oleaeuropaea_Ile19
Cucumissativus_Ile3
Caryaillinoinensis_Ile18
Fragariavesca_Ile7
Prunuspersica_Ile21
Arachishypogaea_Ile3
Phaseolusvulgaris_Ile18
Trifoliumpratense_Ile8
Cicerarietinum_Ile8
Lotusjaponicus_Ile1
Linumusitatissimum_Ile30
Salixpurpurea_Ile2
Populustrichocarpa_Ile10
Manihotesculenta_Ile34
Gossypiumraimondii_Ile12
Gossypiumhirsutum_Ile35
Theobromacacao_Ile9
Lunariaannua_Ile15
Schrenkiellaparvula_Ile12
Cakilemaritima_Ile7
Brassicarapa_Ile7
Sinapisalba_Ile43
Isatistinctoria_Ile1
Myagrumperfoliatum_Ile24
Thlaspiarvense_Ile10
Iberisamara_Ile3
Boecherastricta_Ile10
Malcolmiamaritima_Ile20
Arabidopsisthaliana_Ile7
Capsellagrandiflora_Ile4
Lepidiumsativum_Ile23
Diptychocarpusstrictus_Ile17
Caricapapaya_Ile7
Citrussinensis_Ile6
Anacardiumoccidentale_Ile1
Eucalyptusgrandis_Ile5
Ceratophyllumdemersum_Ile14
Amborellatrichopoda_Ile11
Nymphaeathermarum_Ile10
Nymphaeacolorata_Ile6
Euryaleferox_Ile11
Acorusamericanus_Ile12
Zosteramarina_Ile10
Spirodelapolyrhiza_Ile3
Dioscoreaalata_Ile7
Asparagusofficinalis_Ile5
Ananascomosus_Ile3
Brachypodiumdistachyon_Ile7
Hordeumvulgare_Ile28
Thinopyrumintermedium_Ile35

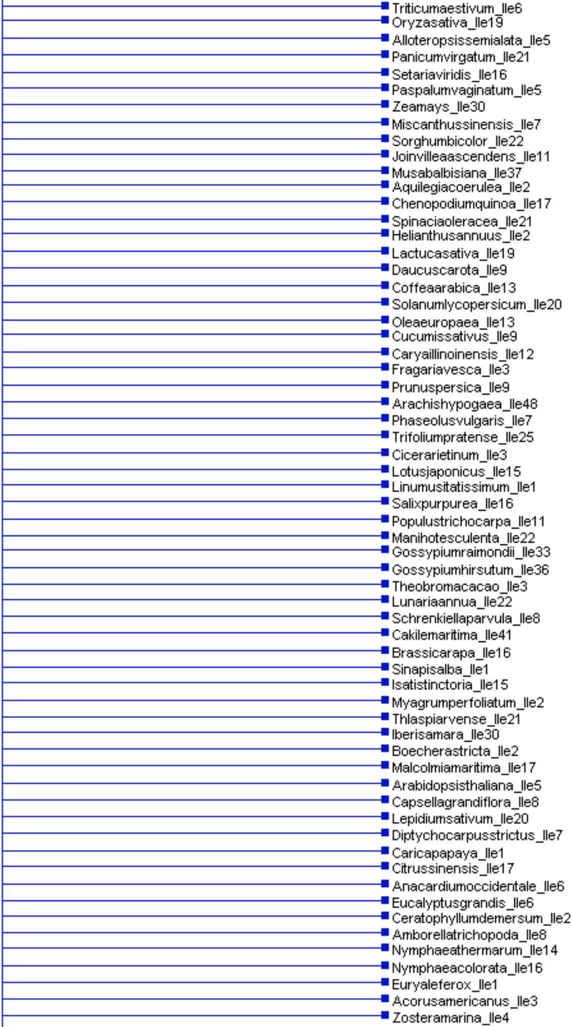
Triticumaestivum_Ile6
Oryzasativa_Ile19
Alloteropsissemialata_Ile5
Panicumvirgatum_Ile21
Setariaviridis_Ile16
Paspalumvaginatum_Ile5
Zeamays_Ile30
Miscanthussinensis_Ile7
Sorghumbicolor_Ile22
Joinvilleaascendens_Ile11
Musabalbisiana_Ile37
Aquilegiacoerulea_Ile2
Chenopodiumquinoa_Ile17
Spinaciaoleracea_Ile21
Helianthusannuus_Ile2
Lactucasativa_Ile19
Daucuscarota_Ile9
Coffeaarabica_Ile13
Solanumlycopersicum_Ile20
Oleaeuropaea_Ile13
Cucumissativus_Ile9
Caryaillinoinensis_Ile12
Fragariavesca_Ile3
Prunuspersica_Ile9
Arachishypogaea_Ile48
Phaseolusvulgaris_Ile7
Trifoliumpratense_Ile25
Cicerarietinum_Ile3
Lotusjaponicus_Ile15
Linumusitatissimum_Ile1
Salixpurpurea_Ile16
Populustrichocarpa_Ile11
Manihotesculenta_Ile22
Gossypiumraimondii_Ile33
Gossypiumhirsutum_Ile36
Theobromacacao_Ile3
Lunariaannua_Ile22
Schrenkiellaparvula_Ile8
Cakilemaritima_Ile41
Brassicarapa_Ile16
Sinapisalba_Ile1
Isatistinctoria_Ile15
Myagrumperfoliatum_Ile2
Thlaspiarvense_Ile21
Iberisamara_Ile30
Boecherastricta_Ile2
Malcolmiamaritima_Ile17
Arabidopsisthaliana_Ile5
Capsellagrandiflora_Ile8
Lepidiumsativum_Ile20
Diptychocarpusstrictus_Ile7
Caricapapaya_Ile1
Citrussinensis_Ile17
Anacardiumoccidentale_Ile6
Eucalyptusgrandis_Ile6
Ceratophyllumdemersum_Ile2
Amborellatrichopoda_Ile8
Nymphaeathermarum_Ile14
Nymphaeacolorata_Ile16
Euryaleferox_Ile1
Acorusamericanus_Ile3
Zosteramarina_Ile4

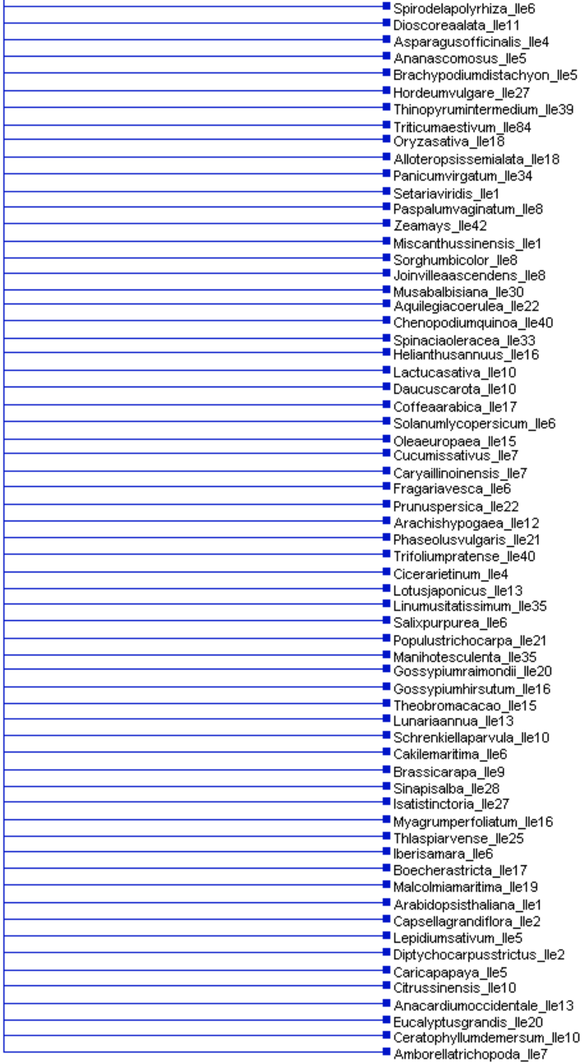

Image generated with Notung 2.9.1.5, on 05 22, 23
