# Supplementary material for: tRNA gene content, structure, and organization in the flowering plant lineage
Source: Front Plant Sci. 2024 Dec 23;15:1486612. doi: 10.3389/fpls.2024.1486612 (PMC11700998; doi:10.3389/fpls.2024.1486612)
Supplement: Supplementary file 7 [file DataSheet7.pdf]

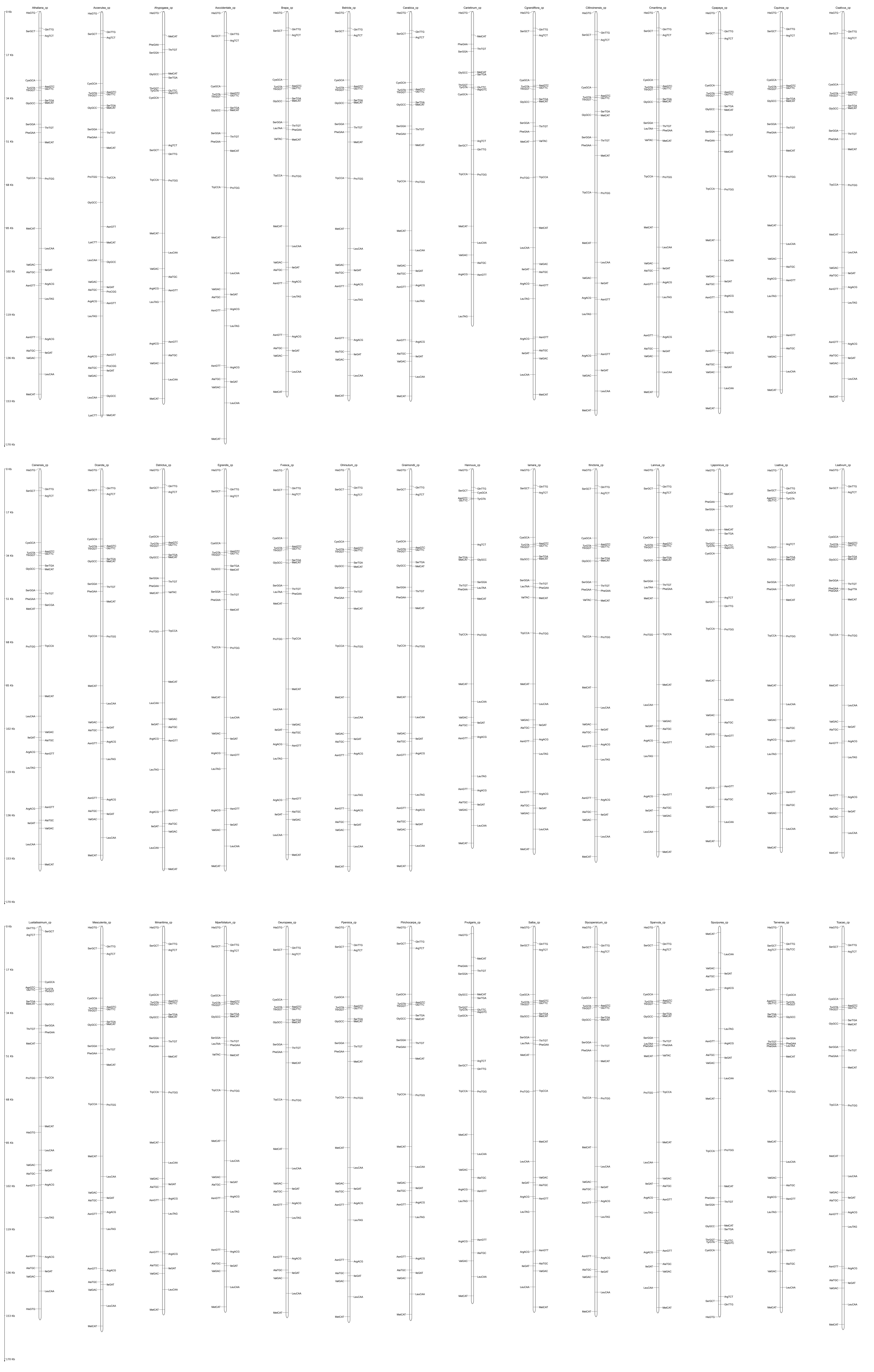

Athaliana_cp
Acoerulea_cp
Ahypogaea_cp
Aoccidentale_cp
Brapa_cp
Bstricta_cp
Carabica_cp
Carietinum_cp
Cgrandiflora_cp
Cillinoinensis_cp
Cmaritima_cp
Cpapaya_cp
Cquinoa_cp
Csativus_cp
Csinensis_cp
Dcarota_cp
Dstrictus_cp
Egrandis_cp
Fvesca_cp
Ghirsutum_cp
Gramondii_cp
Hannuus_cp
Iamara_cp
Itinctoria_cp
Lannua_cp
Ljaponicus_cp
Lsativa_cp
Lsativum_cp
Lusitatissimum_cp
Mesculenta_cp
Mmaritima_cp
Mperfoliatum_cp
Oeuropaea_cp
Ppersica_cp
Ptrichocarpa_cp
Pvulgaris_cp
Salba_cp
Slycopersicum_cp
Sparvula_cp
Spurpurea_cp
Tarvense_cp
Tcacao_cp
0 Kb
17 Kb
34 Kb
51 Kb
68 Kb
85 Kb
102 Kb
119 Kb
136 Kb
153 Kb
170 Kb
