# Supplementary material for: tRNA gene content, structure, and organization in the flowering plant lineage
Source: Front Plant Sci. 2024 Dec 23;15:1486612. doi: 10.3389/fpls.2024.1486612 (PMC11700998; doi:10.3389/fpls.2024.1486612)
Supplement: Supplementary file 8 [file DataSheet8.pdf]

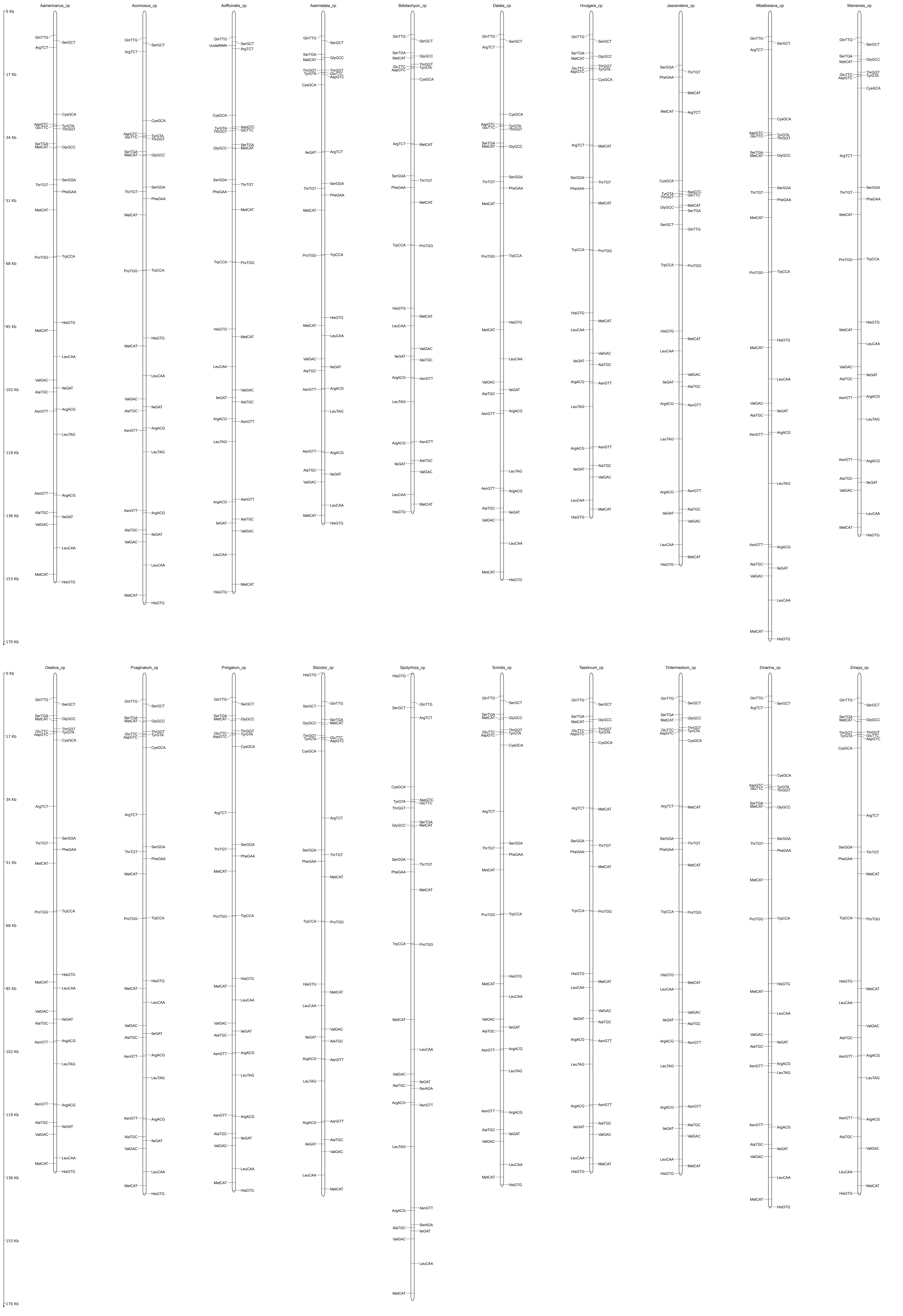
0 Kb
17 Kb
34 Kb
51 Kb
68 Kb
85 Kb
102 Kb
119 Kb
136 Kb
153 Kb
170 Kb
Aamericanus_cp
GlnTTG SerGCT ArgTCT CysGCA AspGTC TyrGTA GluTTC ThrGGT SerTGA MetCAT GlyGCC SerGGA ThrTGT PheGAA MetCAT ProTGG TrpCCA HisGTG MetCAT LeuCAA ValGAC IleGAT AlaTGC ArgACG AsnGTT LeuTAG AsnGTT ArgACG AlaTGC IleGAT ValGAC LeuCAA MetCAT HisGTG
Acomosus_cp
GlnTTG SerGCT ArgTCT CysGCA AspGTC TyrGTA GluTTC ThrGGT SerTGA MetCAT GlyGCC SerGGA ThrTGT PheGAA MetCAT ProTGG TrpCCA HisGTG MetCAT LeuCAA ValGAC IleGAT AlaTGC ArgACG AsnGTT LeuTAG AsnGTT ArgACG AlaTGC IleGAT ValGAC LeuCAA MetCAT HisGTG
Aofficinalis_cp
GlnTTG SerGCT UndetNNN ArgTCT CysGCA AspGTC TyrGTA GluTTC ThrGGT SerTGA GlyGCC MetCAT SerGGA ThrTGT PheGAA MetCAT TrpCCA ProTGG HisGTG MetCAT LeuCAA ValGAC IleGAT AlaTGC ArgACG AsnGTT LeuTAG AsnGTT ArgACG AlaTGC IleGAT ValGAC LeuCAA MetCAT HisGTG
Asemialata_cp
GlnTTG SerGCT SerTGA GlyGCC MetCAT ThrGGT ThrGGT TyrGTA GluTTC AspGTC CysGCA IleGAT ArgTCT SerGGA ThrTGT PheGAA MetCAT ProTGG TrpCCA HisGTG MetCAT LeuCAA ValGAC IleGAT AlaTGC AsnGTT ArgACG LeuTAG AsnGTT ArgACG AlaTGC IleGAT ValGAC LeuCAA MetCAT HisGTG
Bdistachyon_cp
GlnTTG SerGCT SerTGA GlyGCC MetCAT ThrGGT GluTTC TyrGTA AspGTC CysGCA ArgTCT MetCAT SerGGA ThrTGT PheGAA MetCAT TrpCCA ProTGG HisGTG MetCAT LeuCAA ValGAC IleGAT AlaTGC ArgACG AsnGTT LeuTAG AsnGTT ArgACG AlaTGC IleGAT ValGAC LeuCAA MetCAT HisGTG
Dalata_cp
GlnTTG SerGCT ArgTCT CysGCA AspGTC TyrGTA GluTTC ThrGGT SerTGA MetCAT GlyGCC SerGGA ThrTGT PheGAA MetCAT ProTGG TrpCCA HisGTG MetCAT LeuCAA ValGAC IleGAT AlaTGC ArgACG AsnGTT LeuTAG AsnGTT ArgACG AlaTGC IleGAT ValGAC LeuCAA MetCAT HisGTG
Hvulgare_cp
GlnTTG SerGCT SerTGA GlyGCC MetCAT ThrGGT GluTTC TyrGTA AspGTC CysGCA ArgTCT MetCAT SerGGA ThrTGT PheGAA MetCAT TrpCCA ProTGG HisGTG MetCAT LeuCAA ValGAC IleGAT AlaTGC ArgACG AsnGTT LeuTAG ArgACG AsnGTT AlaTGC IleGAT ValGAC LeuCAA MetCAT HisGTG
Jascendens_cp
SerGGA ThrTGT PheGAA MetCAT MetCAT ArgTCT CysGCA AspGTC TyrGTA GluTTC ThrGGT MetCAT GlyGCC SerTGA SerGCT GlnTTG TrpCCA ProTGG HisGTG MetCAT LeuCAA ValGAC IleGAT AlaTGC ArgACG AsnGTT LeuTAG ArgACG AsnGTT AlaTGC IleGAT ValGAC LeuCAA MetCAT HisGTG
Mbalbisiana_cp
GlnTTG SerGCT ArgTCT CysGCA AspGTC TyrGTA GluTTC ThrGGT SerTGA MetCAT GlyGCC SerGGA ThrTGT PheGAA MetCAT ProTGG TrpCCA HisGTG MetCAT LeuCAA ValGAC IleGAT AlaTGC ArgACG AsnGTT LeuTAG AsnGTT ArgACG AlaTGC IleGAT ValGAC LeuCAA MetCAT HisGTG
Msinensis_cp
GlnTTG SerGCT SerTGA GlyGCC MetCAT ThrGGT GluTTC TyrGTA AspGTC CysGCA ArgTCT SerGGA ThrTGT PheGAA MetCAT ProTGG TrpCCA HisGTG MetCAT LeuCAA ValGAC IleGAT AlaTGC AsnGTT ArgACG LeuTAG AsnGTT ArgACG AlaTGC IleGAT ValGAC LeuCAA MetCAT HisGTG
Osativa_cp
GlnTTG SerGCT SerTGA GlyGCC MetCAT ThrGGT GluTTC TyrGTA AspGTC CysGCA ArgTCT SerGGA ThrTGT PheGAA MetCAT ProTGG TrpCCA HisGTG MetCAT LeuCAA ValGAC IleGAT AlaTGC ArgACG AsnGTT LeuTAG AsnGTT ArgACG AlaTGC IleGAT ValGAC LeuCAA MetCAT HisGTG
Pvaginatum_cp
GlnTTG SerGCT SerTGA GlyGCC MetCAT ThrGGT GluTTC TyrGTA AspGTC CysGCA ArgTCT SerGGA ThrTGT PheGAA MetCAT ProTGG TrpCCA HisGTG MetCAT LeuCAA ValGAC IleGAT AlaTGC ArgACG AsnGTT LeuTAG AsnGTT ArgACG AlaTGC IleGAT ValGAC LeuCAA MetCAT HisGTG
Pvirgatum_cp
GlnTTG SerGCT SerTGA GlyGCC MetCAT ThrGGT GluTTC TyrGTA AspGTC CysGCA ArgTCT SerGGA ThrTGT PheGAA MetCAT ProTGG TrpCCA HisGTG MetCAT LeuCAA ValGAC IleGAT AlaTGC AsnGTT ArgACG LeuTAG AsnGTT ArgACG AlaTGC IleGAT ValGAC LeuCAA MetCAT HisGTG
Sbicolor_cp
HisGTG GlnTTG SerGCT SerTGA MetCAT GlyGCC ThrGGT TyrGTA GluTTC AspGTC CysGCA ArgTCT SerGGA ThrTGT PheGAA MetCAT TrpCCA ProTGG HisGTG MetCAT LeuCAA ValGAC IleGAT AlaTGC ArgACG AsnGTT LeuTAG ArgACG AsnGTT AlaTGC IleGAT ValGAC LeuCAA MetCAT
Spolyrhiza_cp
HisGTG SerGCT GlnTTG ArgTCT CysGCA TyrGTA AspGTC GluTTC ThrGGT SerTGA GlyGCC MetCAT SerGGA ThrTGT PheGAA MetCAT TrpCCA ProTGG MetCAT LeuCAA ValGAC IleGAT AlaTGC SerAGA ArgACG AsnGTT LeuTAG AsnGTT ArgACG SerAGA AlaTGC IleGAT ValGAC LeuCAA MetCAT
Sviridis_cp
GlnTTG SerGCT SerTGA GlyGCC MetCAT ThrGGT GluTTC TyrGTA AspGTC CysGCA ArgTCT SerGGA ThrTGT PheGAA MetCAT ProTGG TrpCCA HisGTG MetCAT LeuCAA ValGAC IleGAT AlaTGC AsnGTT ArgACG LeuTAG AsnGTT ArgACG AlaTGC IleGAT ValGAC LeuCAA MetCAT HisGTG
Taestivum_cp
GlnTTG SerGCT SerTGA GlyGCC MetCAT ThrGGT GluTTC TyrGTA AspGTC CysGCA ArgTCT MetCAT SerGGA ThrTGT PheGAA MetCAT TrpCCA ProTGG HisGTG MetCAT LeuCAA ValGAC IleGAT AlaTGC ArgACG AsnGTT LeuTAG ArgACG AsnGTT AlaTGC IleGAT ValGAC LeuCAA MetCAT HisGTG
Tintermedium_cp
GlnTTG SerGCT SerTGA GlyGCC MetCAT ThrGGT GluTTC TyrGTA AspGTC CysGCA ArgTCT MetCAT SerGGA ThrTGT PheGAA MetCAT TrpCCA ProTGG HisGTG MetCAT LeuCAA ValGAC IleGAT AlaTGC ArgACG AsnGTT LeuTAG ArgACG AsnGTT AlaTGC IleGAT ValGAC LeuCAA MetCAT HisGTG
Zmarina_cp
GlnTTG SerGCT ArgTCT CysGCA AspGTC TyrGTA GluTTC ThrGGT SerTGA MetCAT GlyGCC SerGGA ThrTGT PheGAA MetCAT ProTGG TrpCCA HisGTG MetCAT LeuCAA ValGAC IleGAT AlaTGC ArgACG AsnGTT LeuTAG AsnGTT ArgACG AlaTGC IleGAT ValGAC LeuCAA MetCAT HisGTG
Zmays_cp
GlnTTG SerGCT SerTGA GlyGCC MetCAT ThrGGT TyrGTA GluTTC AspGTC CysGCA ArgTCT SerGGA ThrTGT PheGAA MetCAT TrpCCA ProTGG HisGTG MetCAT LeuCAA ValGAC AlaTGC AsnGTT ArgACG LeuTAG AsnGTT ArgACG AlaTGC ValGAC LeuCAA MetCAT HisGTG
